# Supplementary material for: Evidence for existence of an apoptosis‐inducing BH3‐only protein, sayonara, in Drosophila
Source: EMBO J. 2023 Feb 2;42(8):e110454. doi: 10.15252/embj.2021110454 (PMC10107002; doi:10.15252/embj.2021110454)
Supplement: Supplementary file 1 — Expanded View Figures PDF [file EMBJ-42-e110454-s006.pdf]

Expanded View Figures

Figure EV1. Alignment of noncanonical BH3-only proteins and a structure of CG14044.

A Alignment of Beclin in humans, chicken, xenopus, zebrafish, and fly demonstrates that the BH3 motif (the red rectangle is the core sequence of the BH3 motif; the pink rectangle is the sequence surrounding the core BH3 motif) is not conserved in the fly. An * (asterisk) indicates positions which have a single, perfectly conserved residue. (A:) (colon) indicates conservation between groups of strongly similar properties. (A.) (period) indicates conservation between groups of weakly similar properties.

B Alignment of Bnip3 in humans, chicken, xenopus, zebrafish, and fly demonstrates that the BH3 motif (the red rectangle is the core sequence of the BH3 motif; the pink rectangle is the sequence surrounding the core BH3 motif) is not conserved in the fly.

C The BH3 motif (LAYNLGVIGDARK) of CG14044 exists in the interhelix region of the protein structure, which was predicted by alphafold 2.

D A representative image of overlayed adult wings: black, + (control); magenta, Synr WT; blue, Synr ΔBH3.

E Amino acid sequence of the coiled-coil domain substitution mutant. All the hydrophobic and charged amino acids were changed to glycine.

F HA-tagged Synr was expressed in the wing pouch. The BH3 deletion or BH3 amino acid change does not reduce the Synr expression levels compared with Synr WT. Two independent pictures of each condition are shown.

Data information: Scale bars, 500 μm in (D) and 50 μm in (F).
Source data are available online for this figure.

© 2023 The Authors

© 2023 The Authors

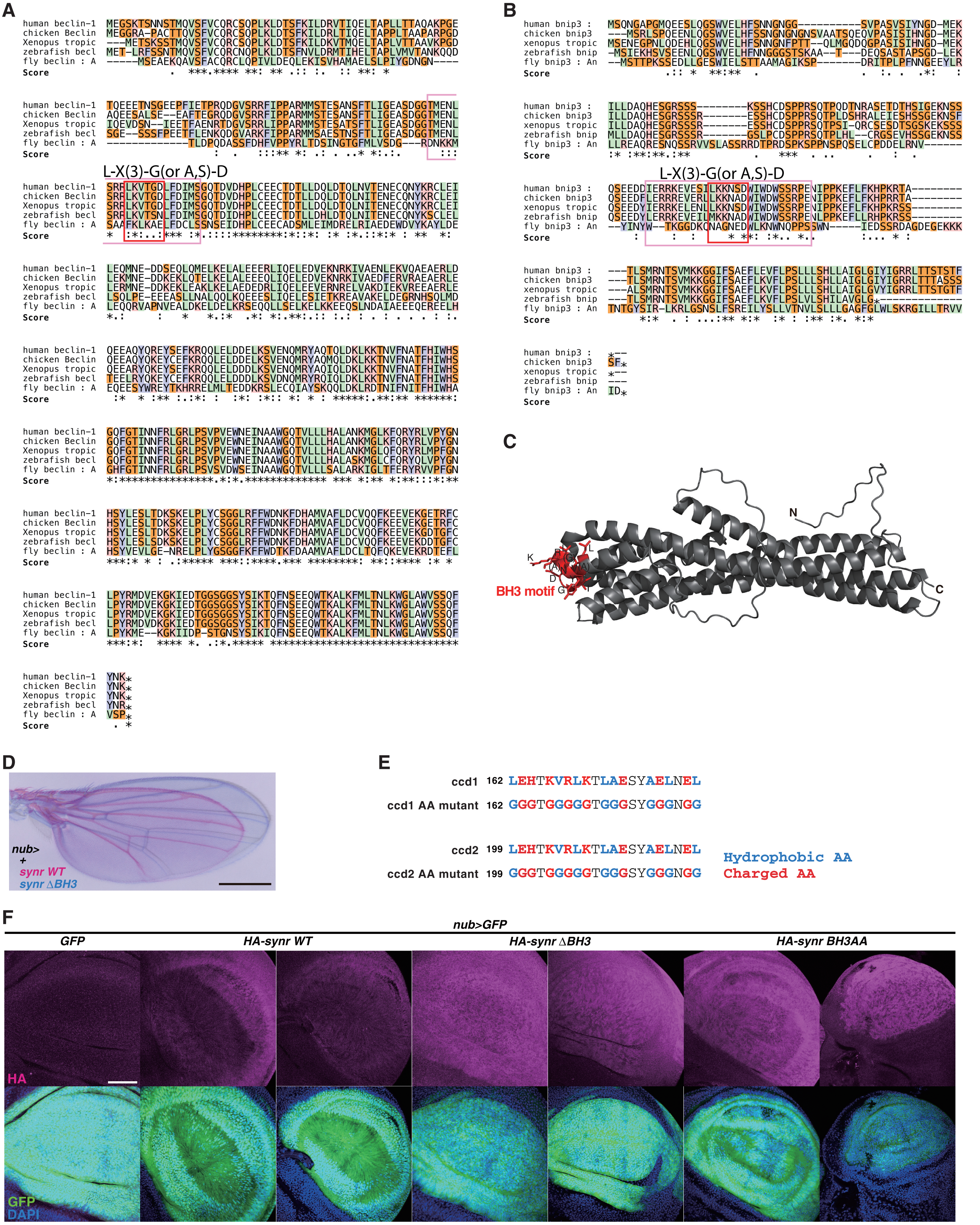

Figure EV1.

 © 2023 The Authors

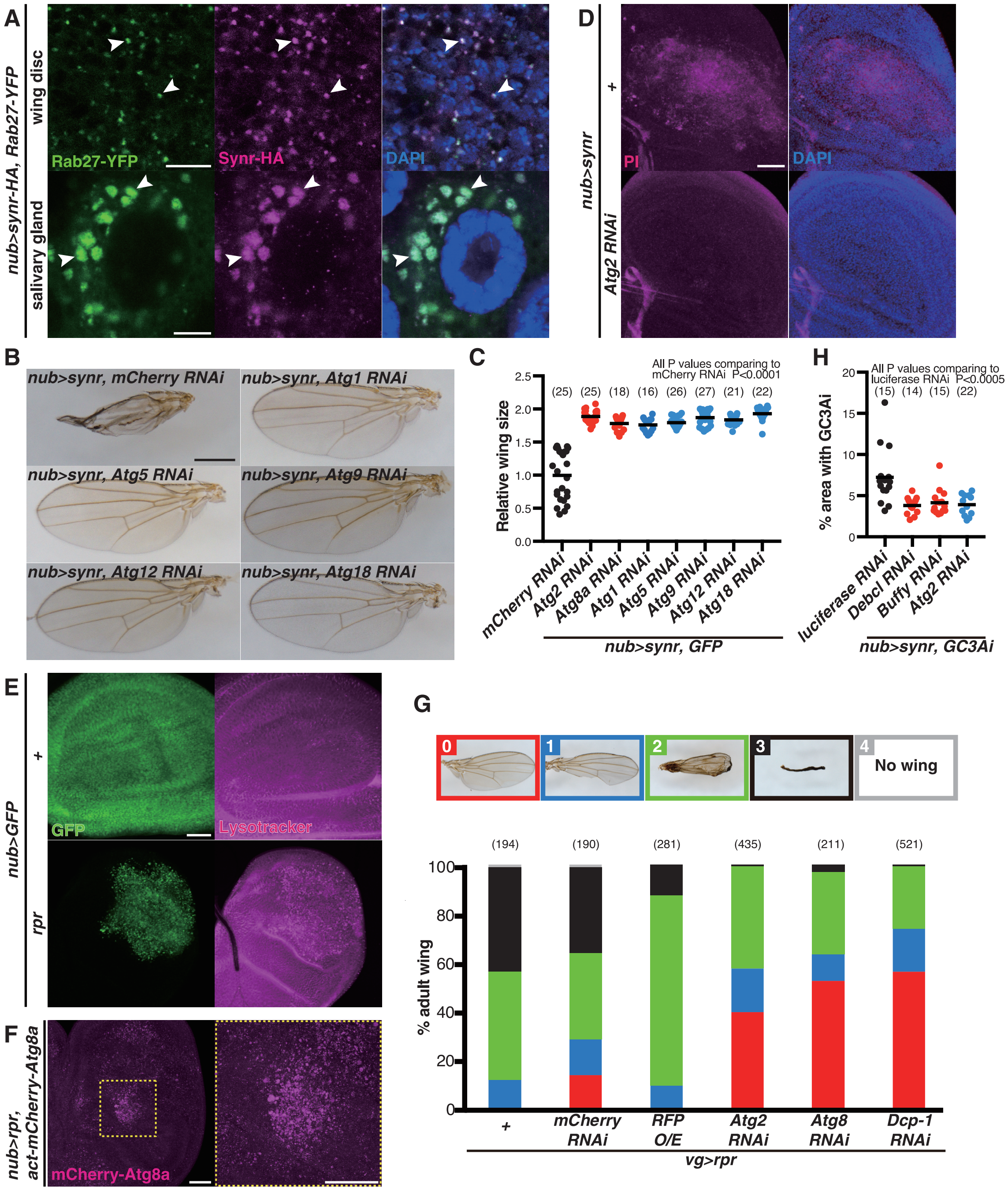

Figure EV2. Synr-induced autophagy.

A Synr is colocalized with Rab27 (arrowheads) in the wing disc and the salivary gland at 112 h AEL.
B The Synr-induced wing structural defect is suppressed by autophagy inhibition.
C Quantification of the wing size with a combination of Synr expression and autophagy inhibition.
D *Atg2* knockdown suppresses Synr-induced cell death, which was detected by propidium iodide (PI).
E A DIAP1 inhibitor, reaper induces autolysosome accumulation in the wing disc.
F Reaper also induces autophagosome accumulation in the wing disc. The yellow-dotted square region is magnified in the right picture.
G Reaper-induced wing defects are suppressed by autophagy inhibition. Wing phenotypes were divided into five classes based on the severity as indicated in pictures.
H Knockdown of *Debcl*, *Buffy*, or *Atg2* significantly suppresses caspase activation compared with the control RNAi for luciferase.

Data information: Statistical significance was determined using one-way ANOVA with Dunnett's *post hoc* test. Scale bars, 10 μm in (A), 500 μm in (B), and 50 μm in (D–F).
Source data are available online for this figure.

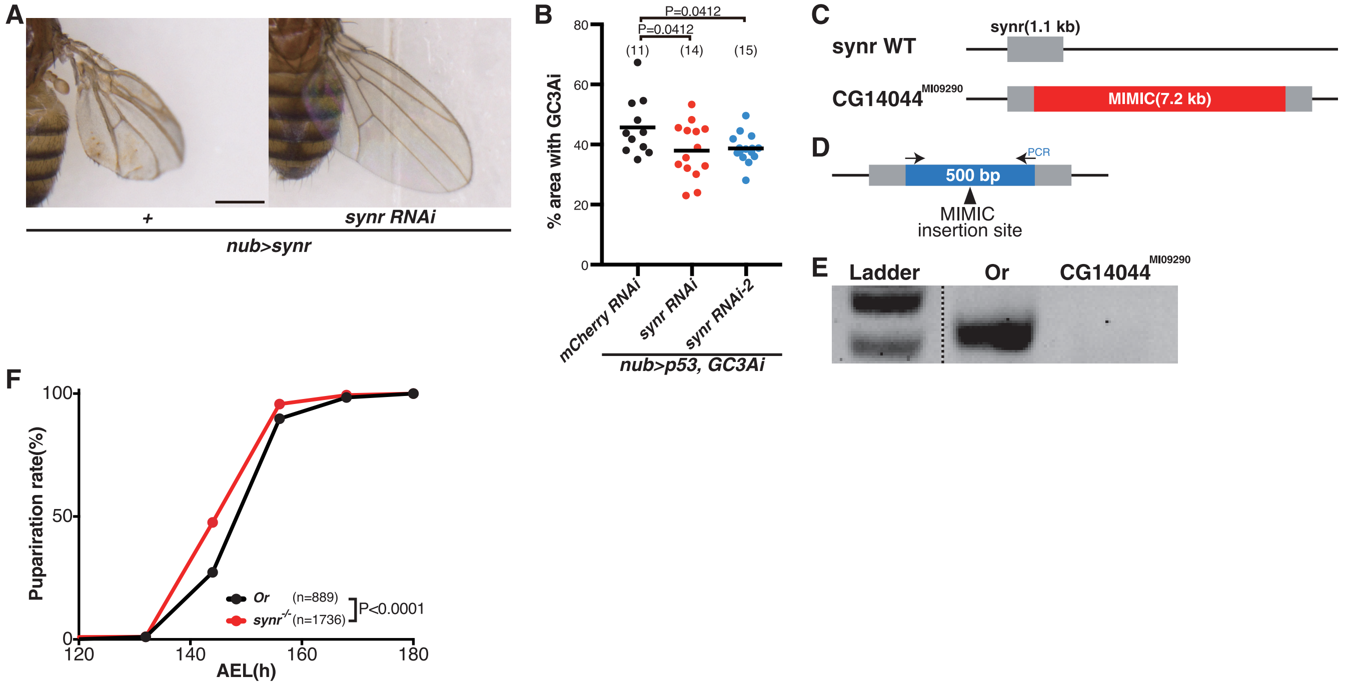

Figure EV3. Knockdown and knockout of *synr*.

A A newly generated *synr* RNAi line can suppress the effect of ectopic *synr* expression on wings.
B Two RNAis for *synr* can suppress p53-mediated caspase activation, which is detected by GC3Ai.
C Schematic of MiMIC (Minos-mediated integration cassette) insertion in *synr* (CGCG14044^{MI09290}).
D A design of primers to detect MiMIC insertion.
E Using the primers, the MiMIC insertion was confirmed by PCR. During extensive outcross of CGCG14044^{MI09290} to OregonR, MiMIC insertion was confirmed by PCR.
F The extensively outcrossed *synr* mutant demonstrates a slight, but reproducible developmental delay during development.

Data information: Statistical significance was determined using one-way ANOVA with Holm-Sidak's multiple comparisons test (B) and a log-rank (Mantel–Cox) test (F). Scale bar, 500 µm in (A).
Source data are available online for this figure.

© 2023 The Authors

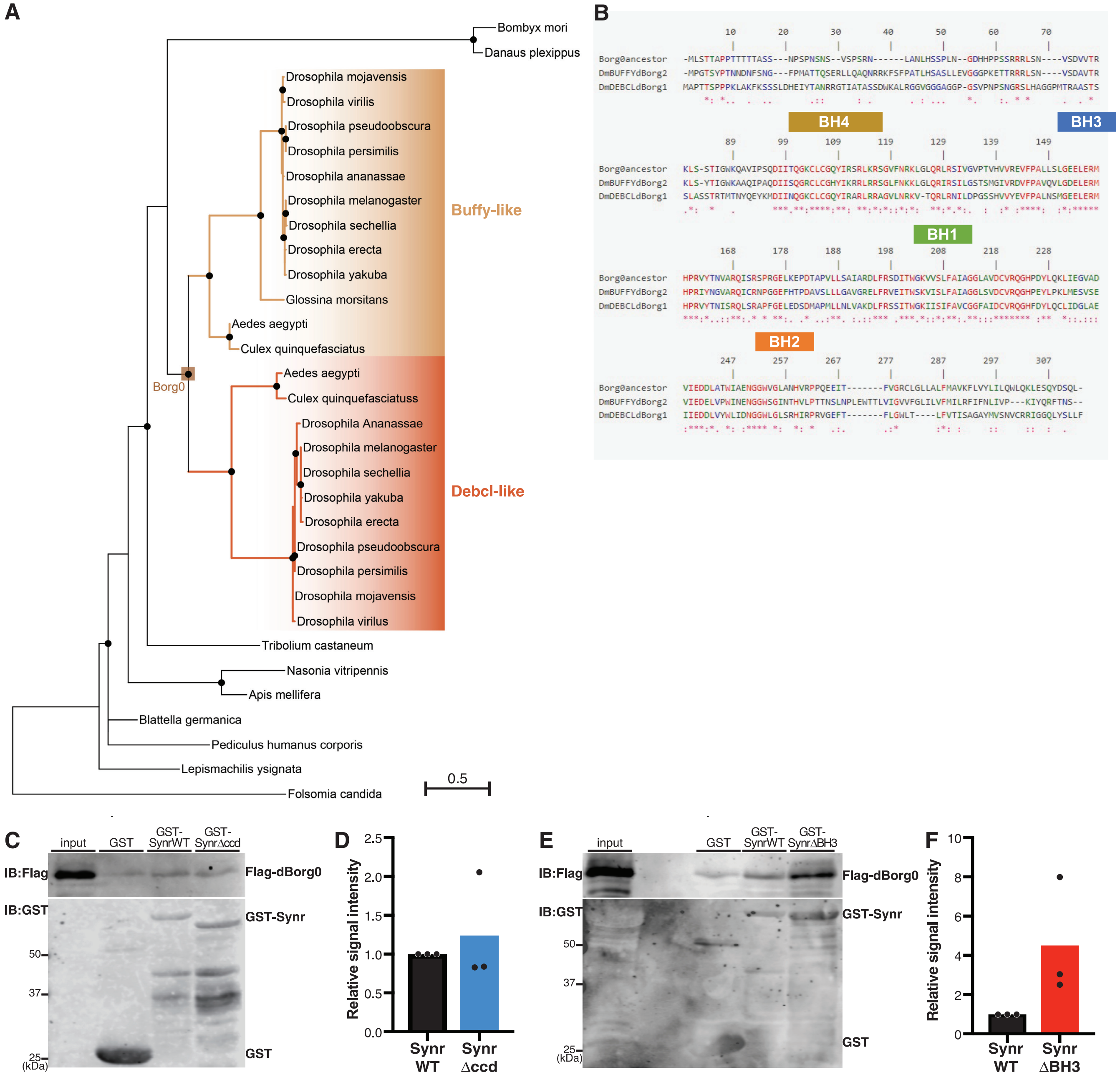

Figure EV4. Evolution of the Buffy–Debcl orthology group in insects.

A Phylogenetic subtree depicting the Buffy–Debcl duplication in Diptera. The Buffy–Debcl ancestral protein before duplication was named dBorg0, since Debcl and Buffy are also known as dBorg-1 and dBorg-2, respectively. The scale bar indicates 0.5 substitution per site.

B The amino acid sequence of dBorg0 was predicted based on ancestral protein reconstruction (see Materials and Methods). Multiple sequence alignment of *Drosophila* Debcl (also known as dBorg-1) and Buffy (dBorg-2) amino acid sequences with that of their putative ancestor dBorg0.

C–F Lysates from HEK293T cells that express Flag-dBorg0 were incubated with GST, GST-Synr WT, GST-Synr ΔBH3, or GST-Synr Δcoiled-coil mutant, which was bound to glutathione Sepharose. Synr WT, Synr BH3 mutant, and Synr coiled-coil domain mutant can pull down dBorg0 in a similar manner, indicating that the coiled-coil region and the BH3 motif are dispensable for Synr's binding to dBorg0, which is similar to Buffy and Debcl.

Source data are available online for this figure.

© 2023 The Authors
