# Supplementary figures and images for: Evidence for existence of an apoptosis‐inducing BH3‐only protein, sayonara, in Drosophila (part 1 of 2)
Source: EMBO J. 2023 Feb 2;42(8):e110454. doi: 10.15252/embj.2021110454 (PMC10107002; doi:10.15252/embj.2021110454)

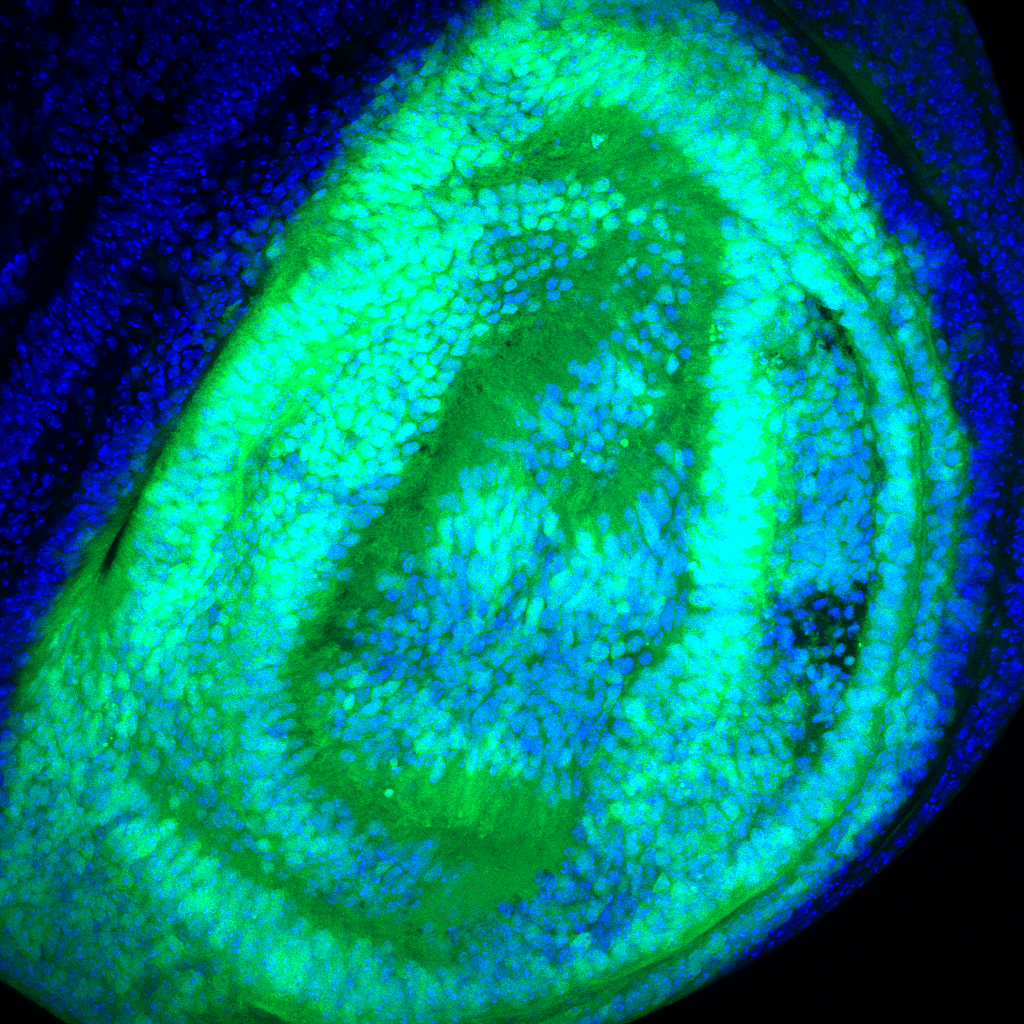

Supplement: Supplementary file 3 — Source Data for Expanded View [file EMBJ-42-e110454-s005.zip › FigEV/FigEV1/synr BH3AA/BH3 AA-Orthogonal Projection-04_c2+3.tif]

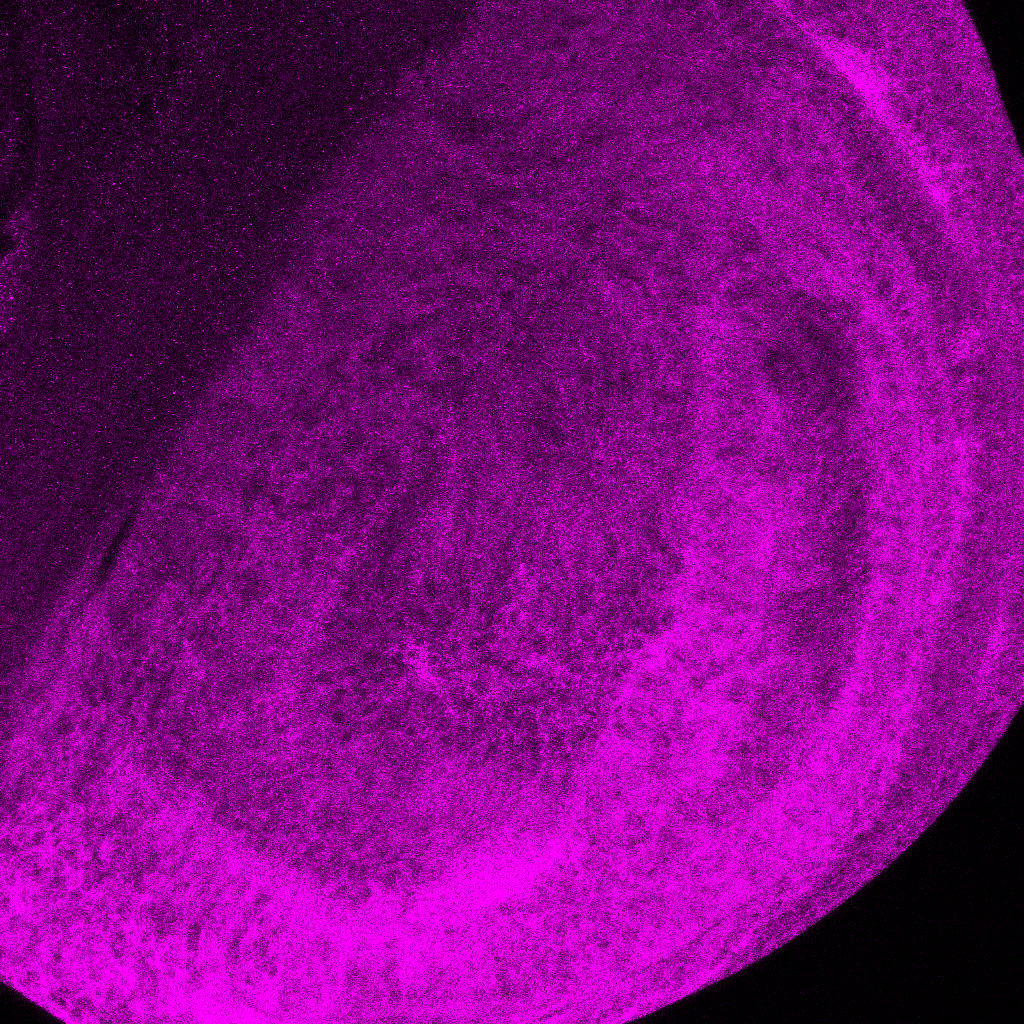

Supplement: Supplementary file 3 — Source Data for Expanded View [file EMBJ-42-e110454-s005.zip › FigEV/FigEV1/synr BH3AA/BH3 AA-Orthogonal Projection-04_c1.tif]

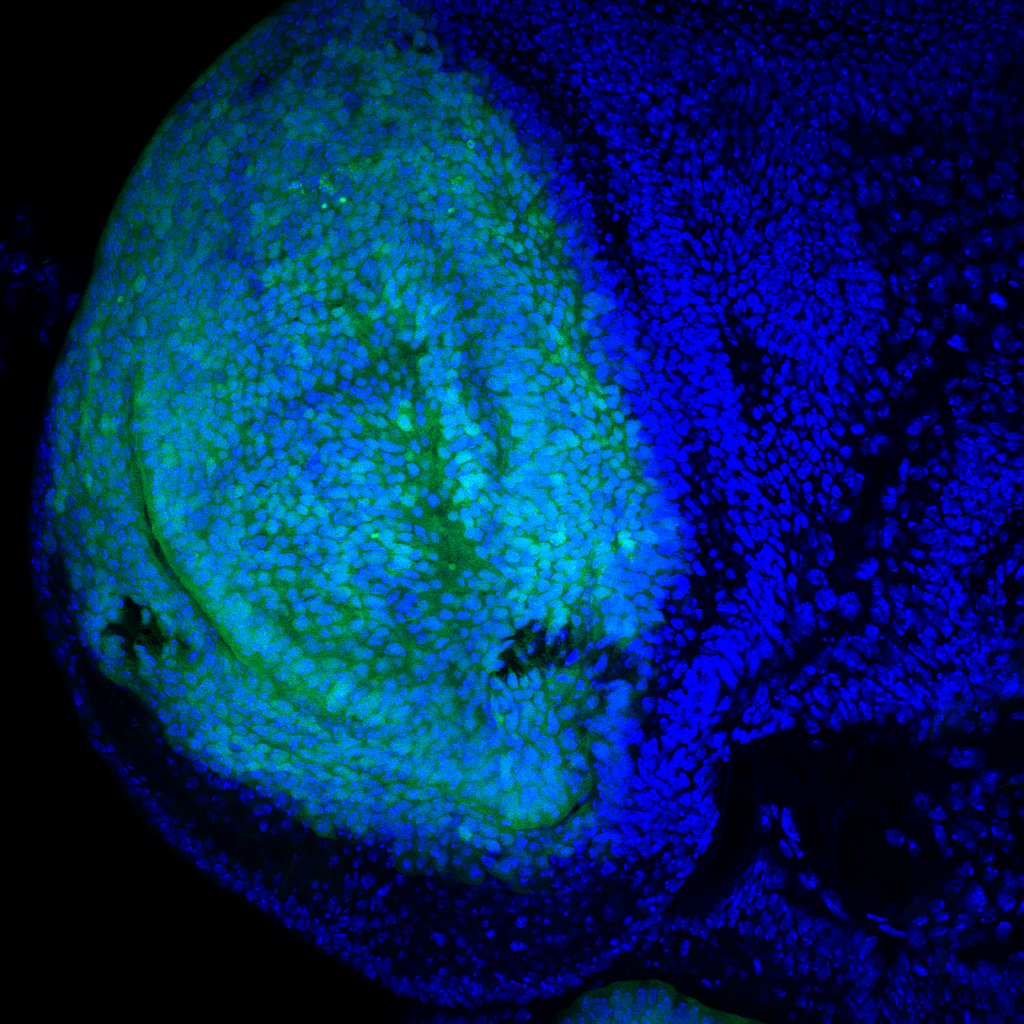

Supplement: Supplementary file 3 — Source Data for Expanded View [file EMBJ-42-e110454-s005.zip › FigEV/FigEV1/synr BH3AA/Experiment-7712-Orthogonal Projection-12_c2+3.tif]

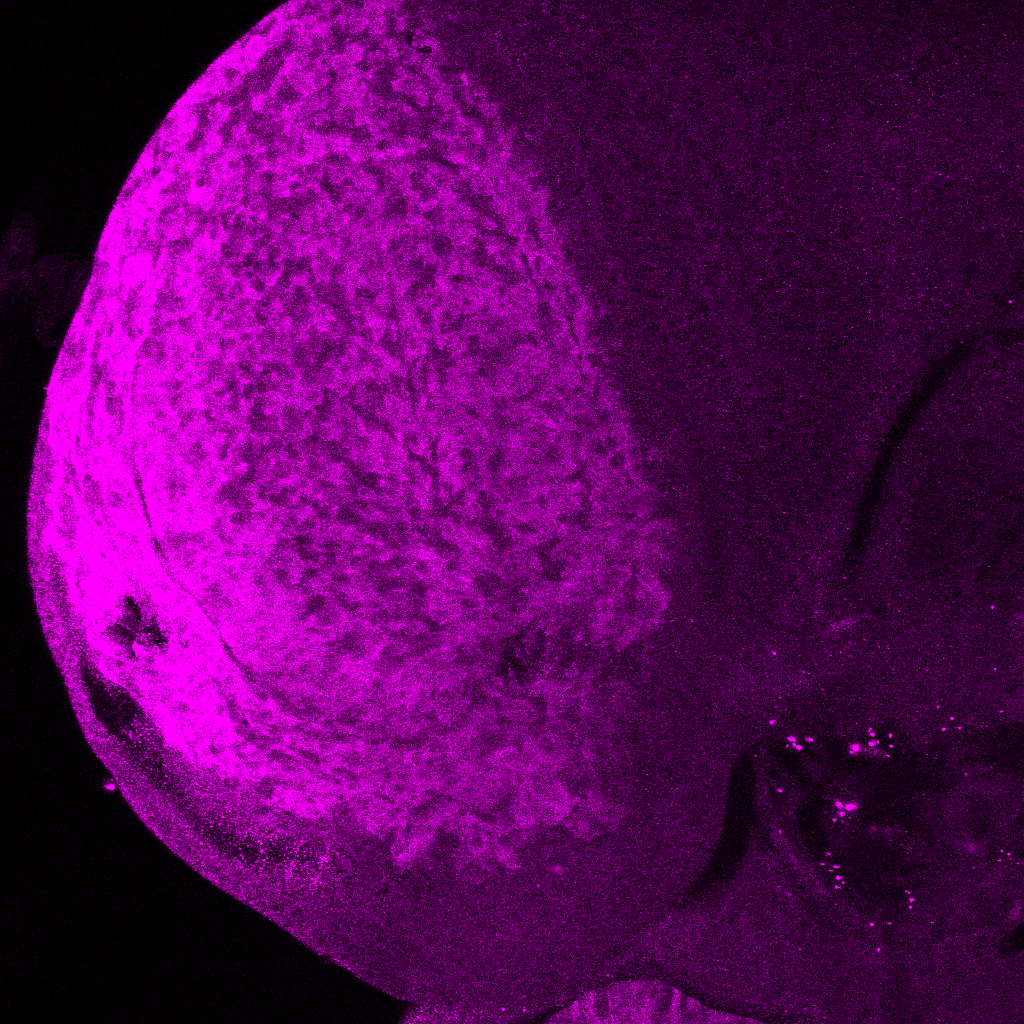

Supplement: Supplementary file 3 — Source Data for Expanded View [file EMBJ-42-e110454-s005.zip › FigEV/FigEV1/synr BH3AA/Experiment-7712-Orthogonal Projection-12_c1.tif]

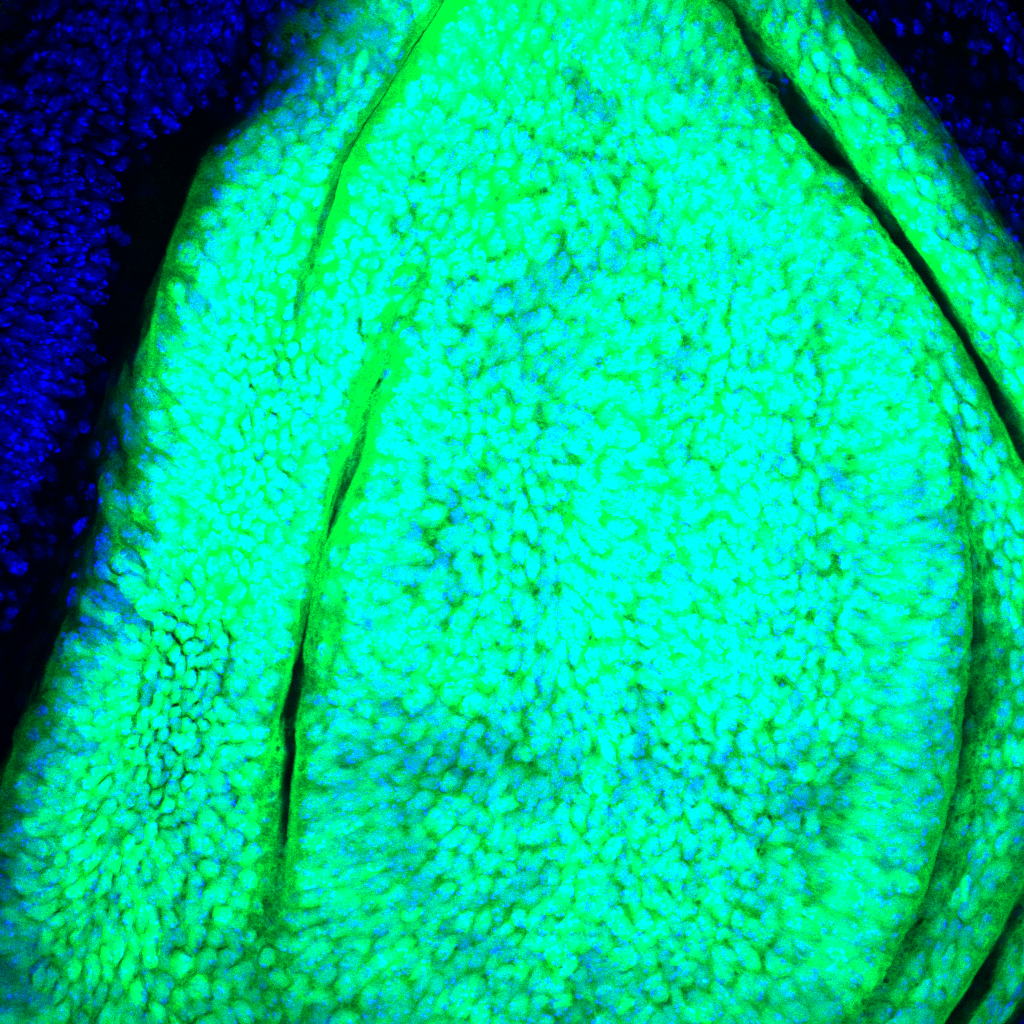

Supplement: Supplementary file 3 — Source Data for Expanded View [file EMBJ-42-e110454-s005.zip › FigEV/FigEV1/GFP/UAS-GFP-Orthogonal Projection-06_c2+3.tif]

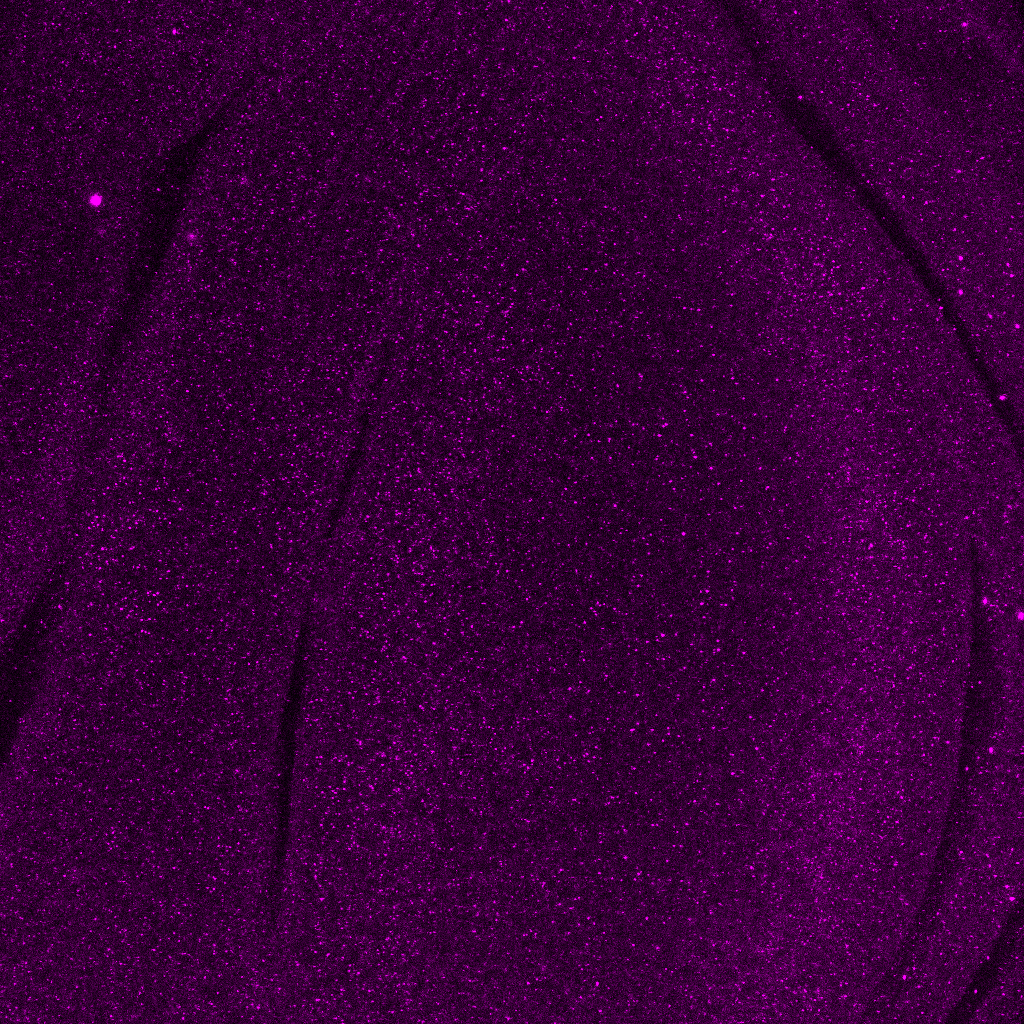

Supplement: Supplementary file 3 — Source Data for Expanded View [file EMBJ-42-e110454-s005.zip › FigEV/FigEV1/GFP/UAS-GFP-Orthogonal Projection-06_c1.tif]

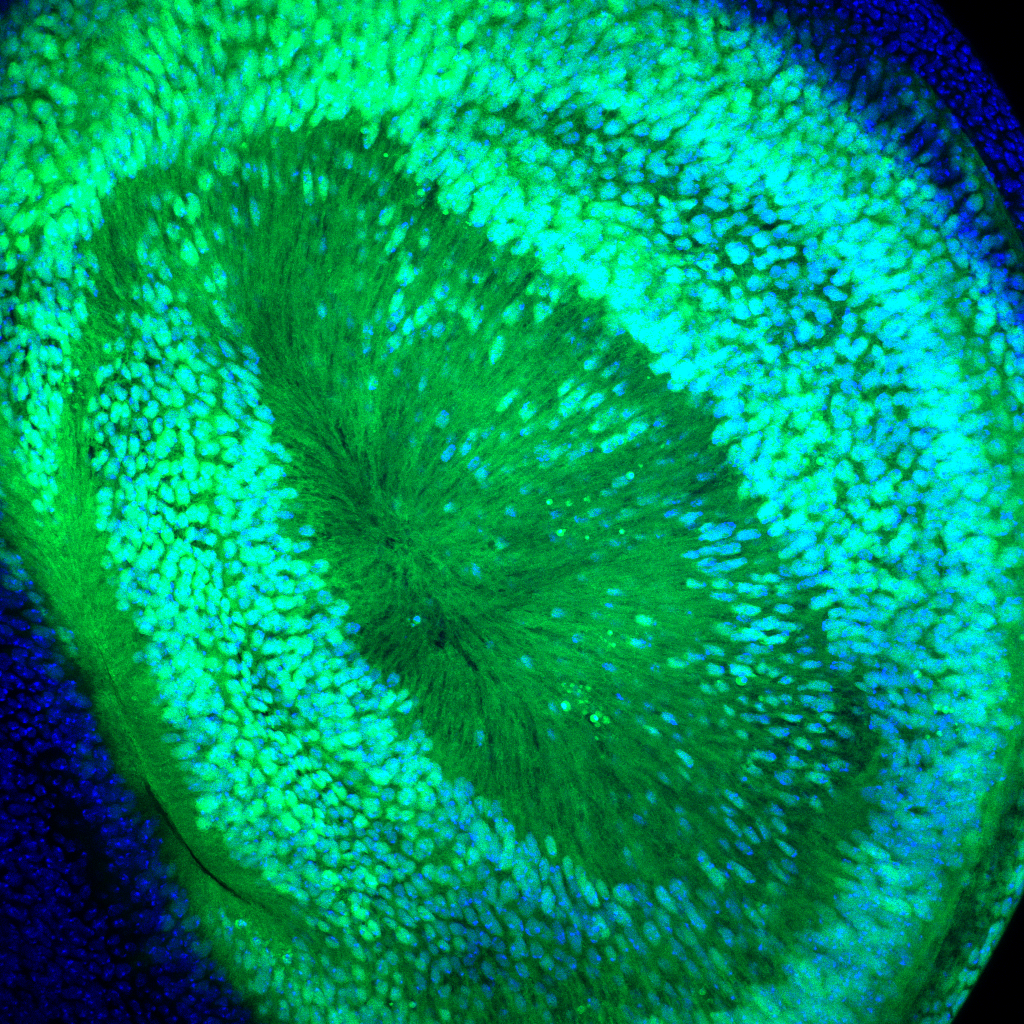

Supplement: Supplementary file 3 — Source Data for Expanded View [file EMBJ-42-e110454-s005.zip › FigEV/FigEV1/synr WT/WT-Orthogonal Projection-07_c2+3.tif]

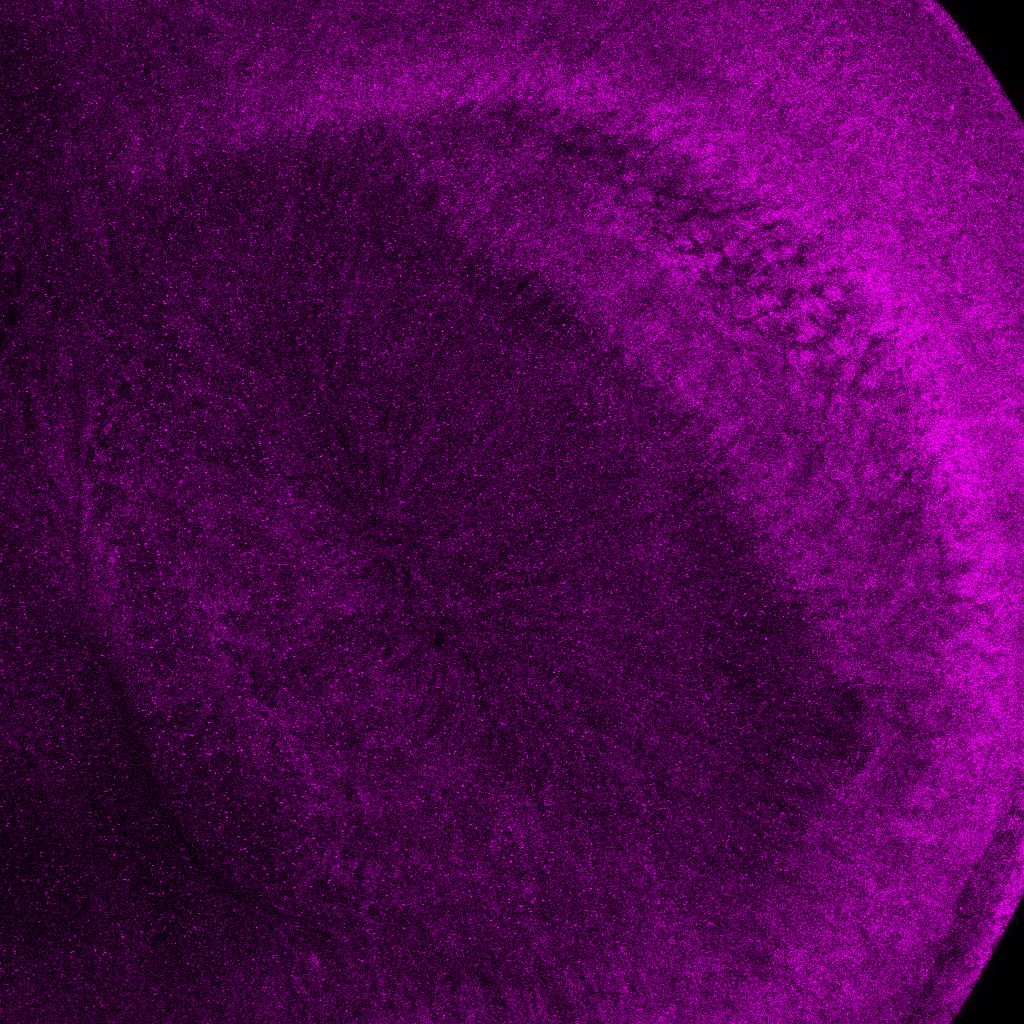

Supplement: Supplementary file 3 — Source Data for Expanded View [file EMBJ-42-e110454-s005.zip › FigEV/FigEV1/synr WT/WT-Orthogonal Projection-07_c1.tif]

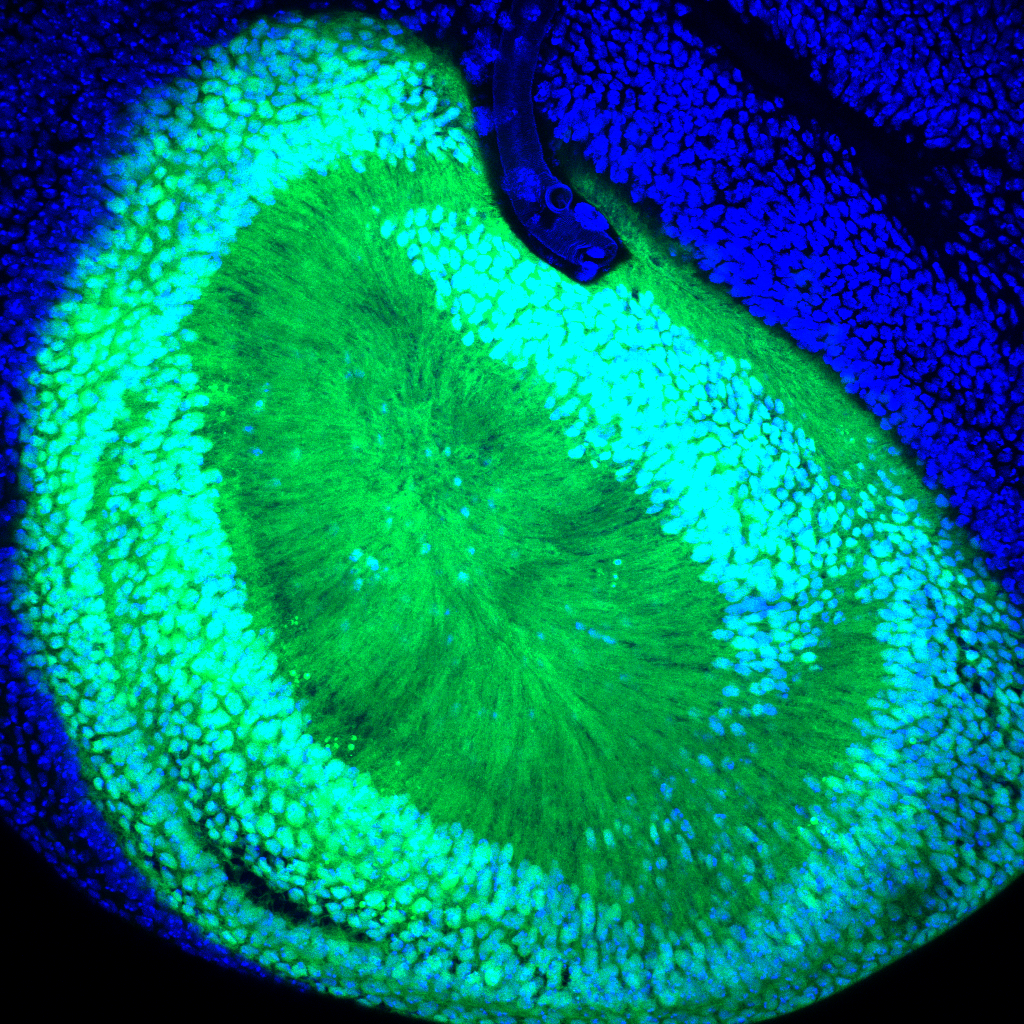

Supplement: Supplementary file 3 — Source Data for Expanded View [file EMBJ-42-e110454-s005.zip › FigEV/FigEV1/synr WT/Experiment-7701-Orthogonal Projection-WT09_c2+3.tif]

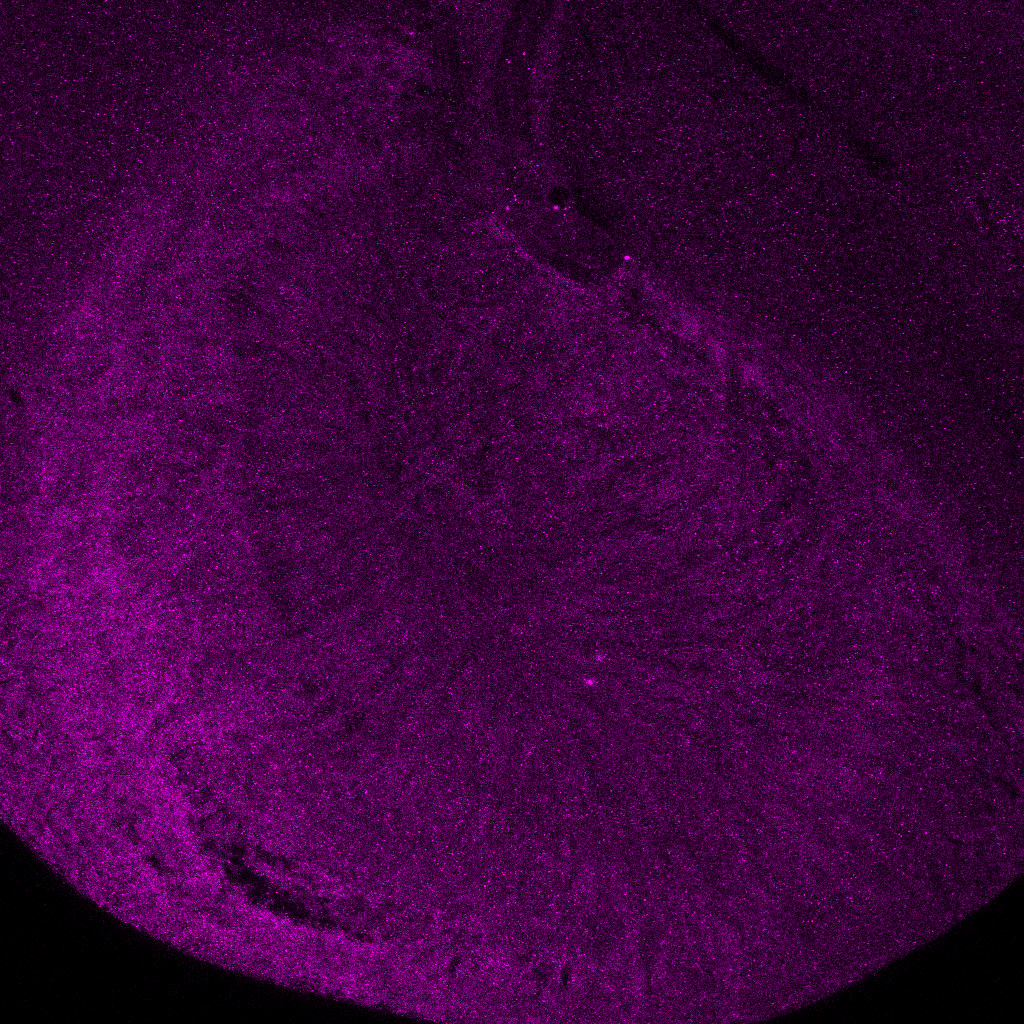

Supplement: Supplementary file 3 — Source Data for Expanded View [file EMBJ-42-e110454-s005.zip › FigEV/FigEV1/synr WT/Experiment-7701-Orthogonal Projection-WT09_c1.tif]

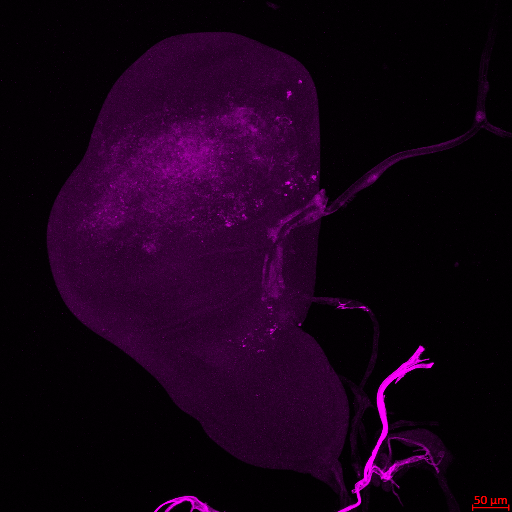

Supplement: Supplementary file 3 — Source Data for Expanded View [file EMBJ-42-e110454-s005.zip › FigEV/FigEV2/FigD/+-PI.tif]

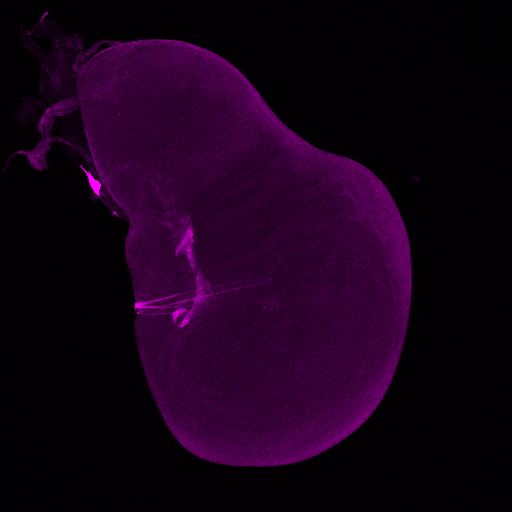

Supplement: Supplementary file 3 — Source Data for Expanded View [file EMBJ-42-e110454-s005.zip › FigEV/FigEV2/FigD/Atg2 RNAi-PI.tif]

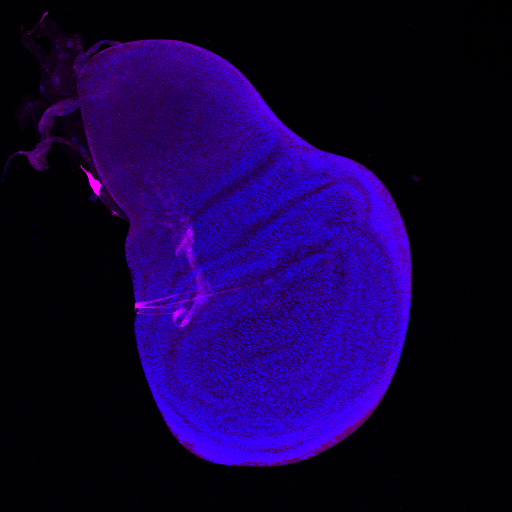

Supplement: Supplementary file 3 — Source Data for Expanded View [file EMBJ-42-e110454-s005.zip › FigEV/FigEV2/FigD/Atg2 RNAi-merge.tif]

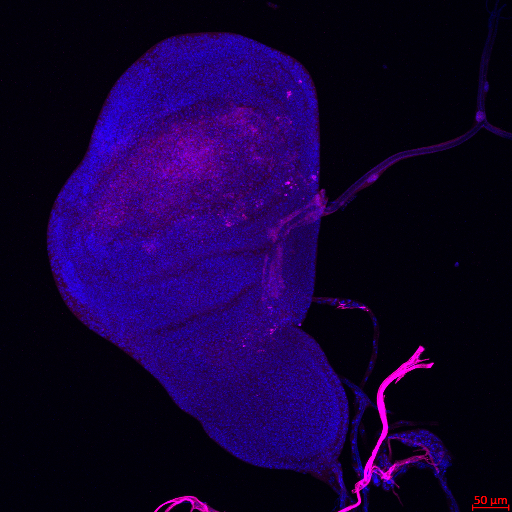

Supplement: Supplementary file 3 — Source Data for Expanded View [file EMBJ-42-e110454-s005.zip › FigEV/FigEV2/FigD/+-merge.tif]

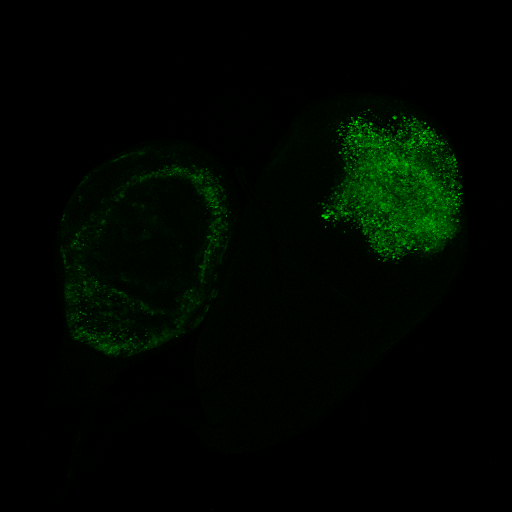

Supplement: Supplementary file 3 — Source Data for Expanded View [file EMBJ-42-e110454-s005.zip › FigEV/FigEV2/FigE/rpr-GFP.tif]

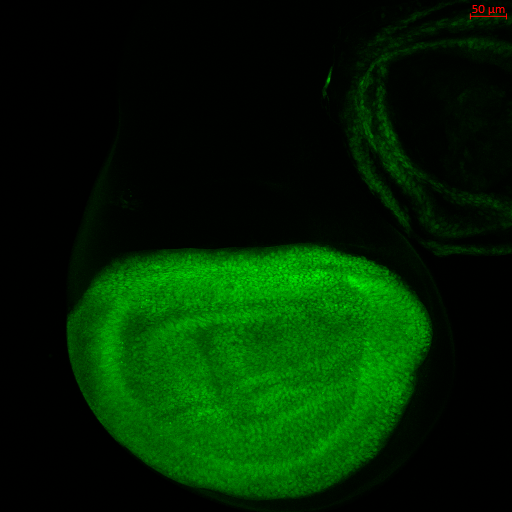

Supplement: Supplementary file 3 — Source Data for Expanded View [file EMBJ-42-e110454-s005.zip › FigEV/FigEV2/FigE/+-GFP.tif]

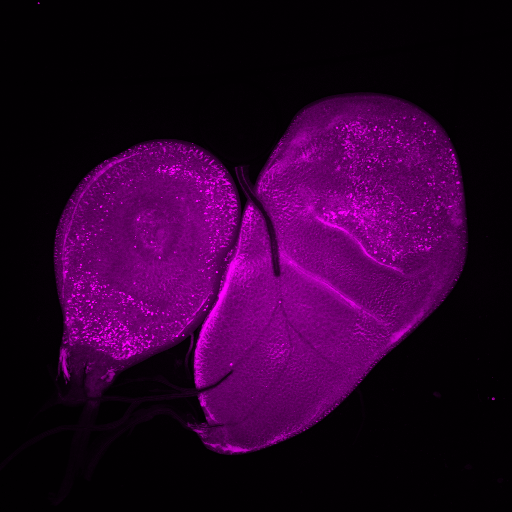

Supplement: Supplementary file 3 — Source Data for Expanded View [file EMBJ-42-e110454-s005.zip › FigEV/FigEV2/FigE/rpr-lysotracker.tif]

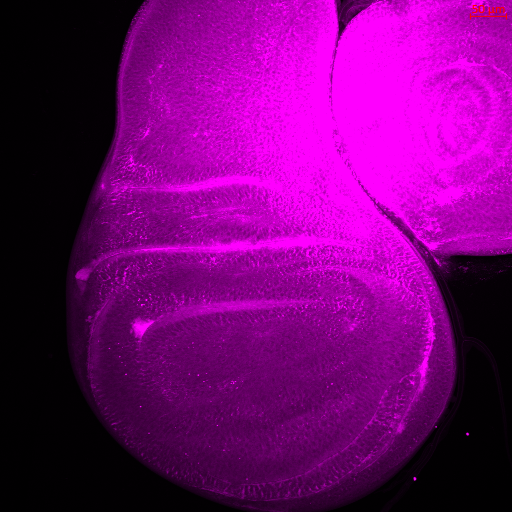

Supplement: Supplementary file 3 — Source Data for Expanded View [file EMBJ-42-e110454-s005.zip › FigEV/FigEV2/FigE/+-lysotracker.tif]

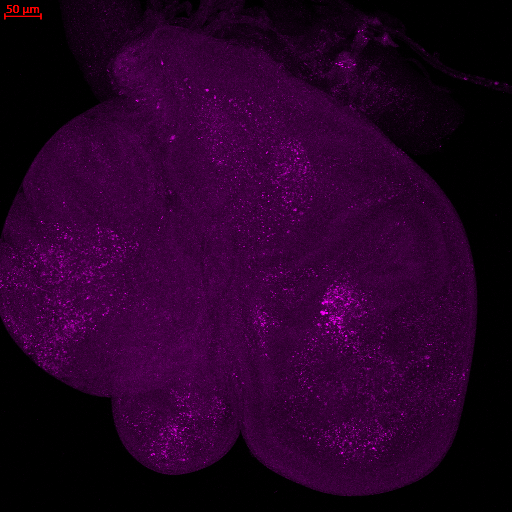

Supplement: Supplementary file 3 — Source Data for Expanded View [file EMBJ-42-e110454-s005.zip › FigEV/FigEV2/FigF/rpr-mCherry atg8a.tif]

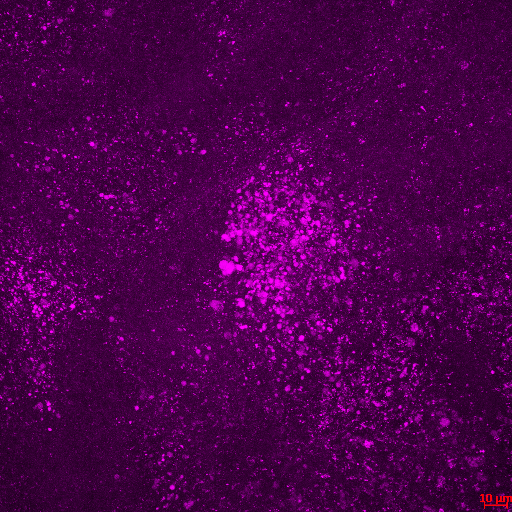

Supplement: Supplementary file 3 — Source Data for Expanded View [file EMBJ-42-e110454-s005.zip › FigEV/FigEV2/FigF/magnified.tif]

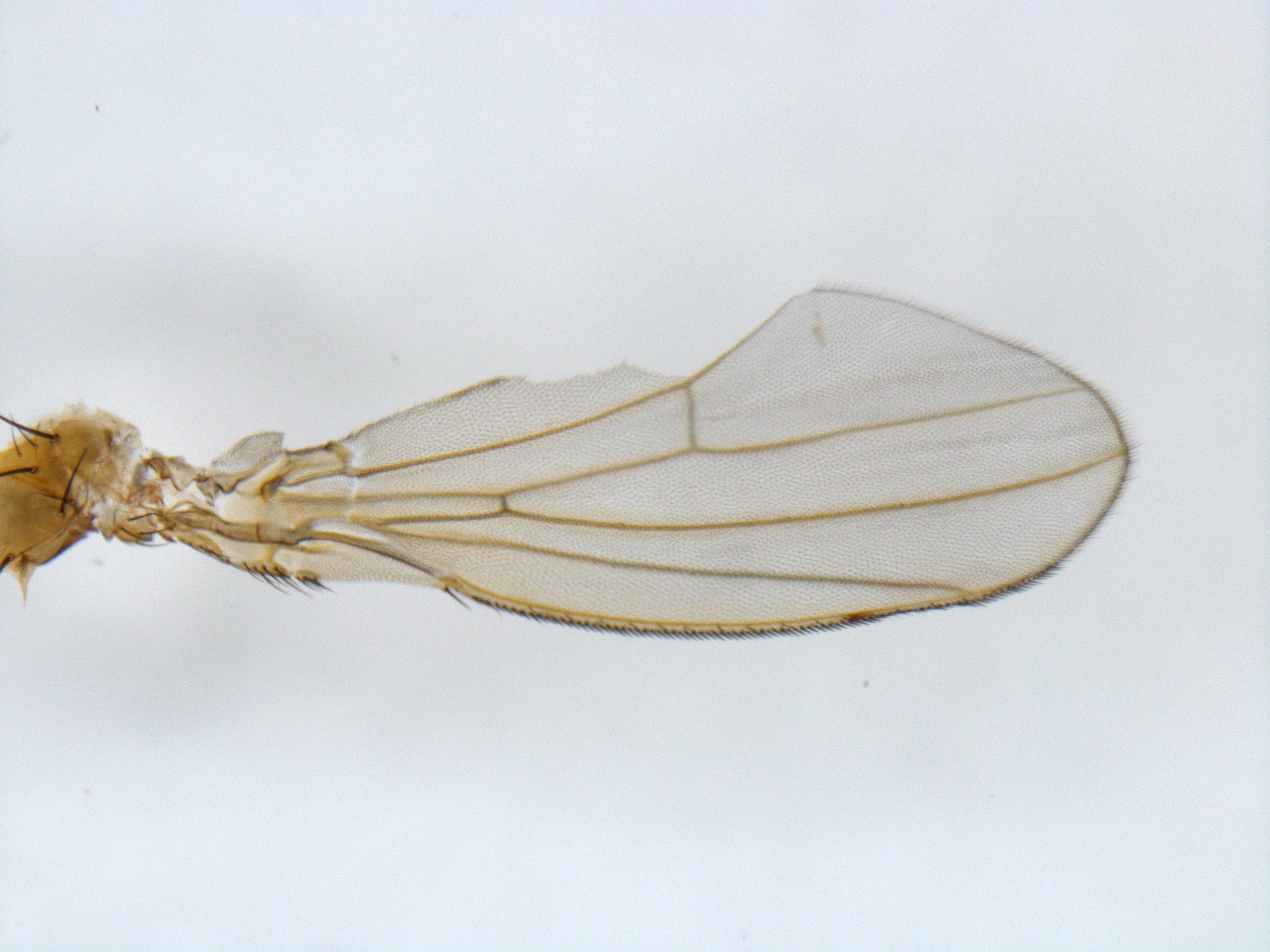

Supplement: Supplementary file 3 — Source Data for Expanded View [file EMBJ-42-e110454-s005.zip › FigEV/FigEV2/FigG/1.jpg]

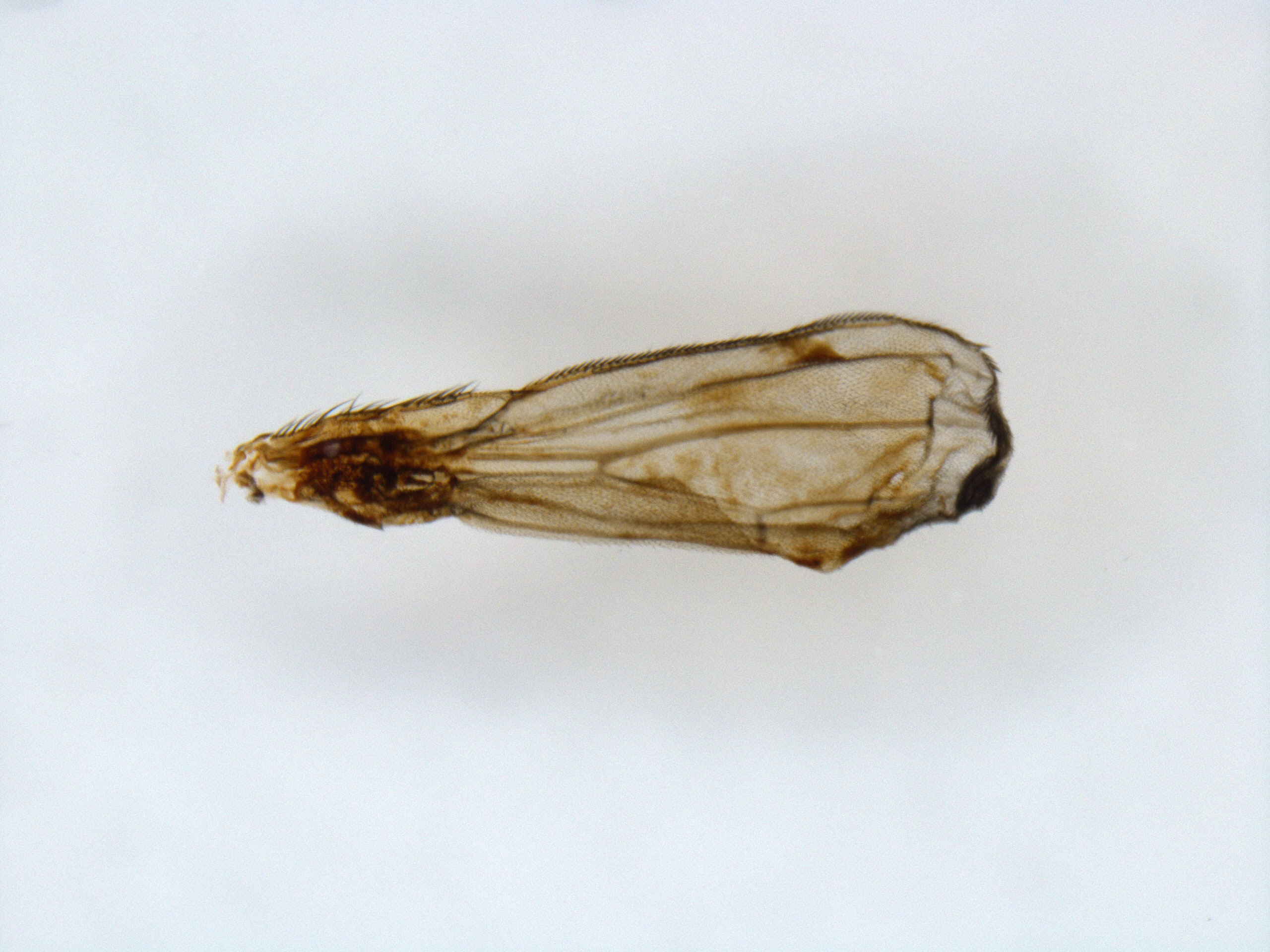

Supplement: Supplementary file 3 — Source Data for Expanded View [file EMBJ-42-e110454-s005.zip › FigEV/FigEV2/FigG/2.jpg]

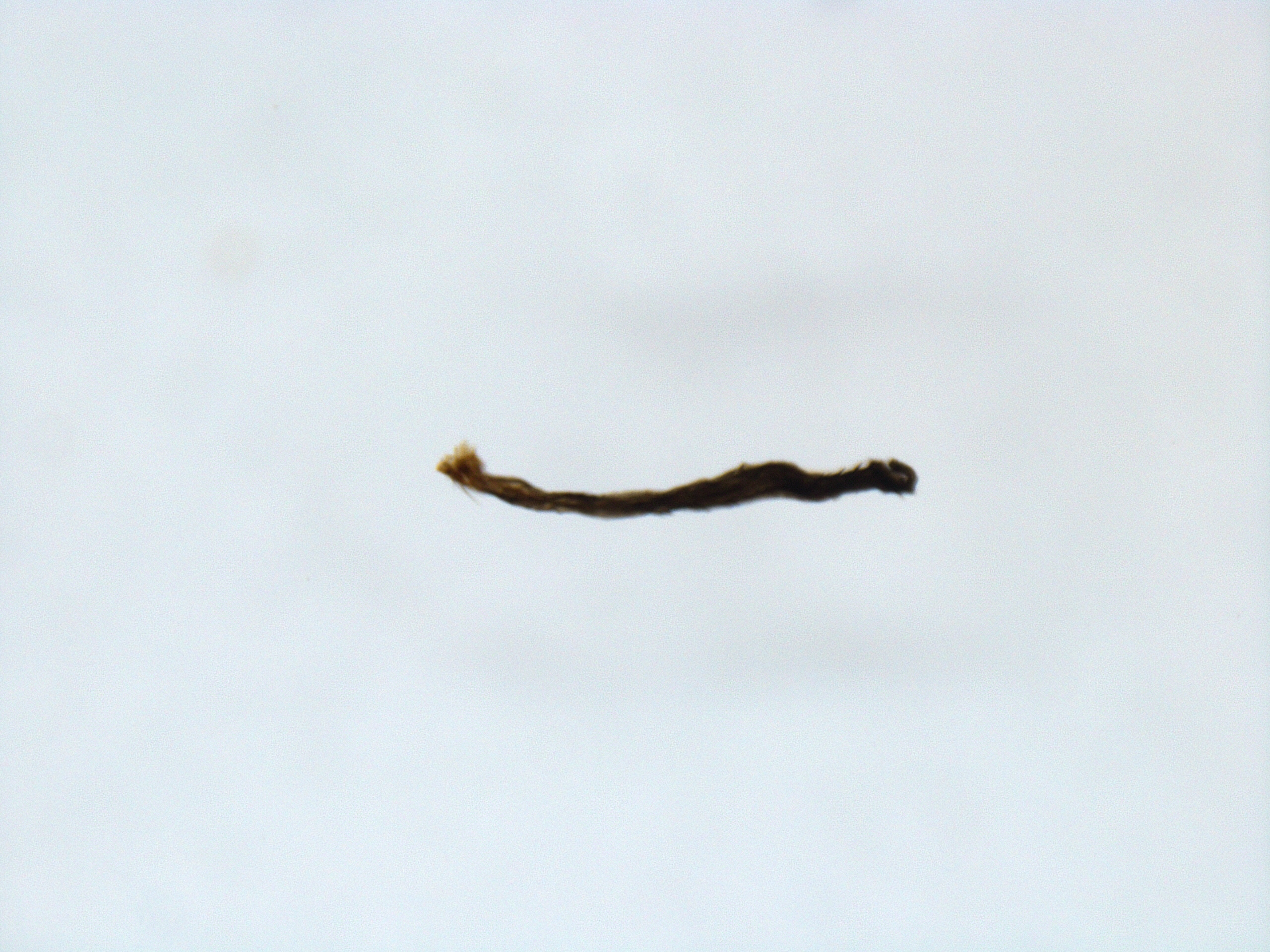

Supplement: Supplementary file 3 — Source Data for Expanded View [file EMBJ-42-e110454-s005.zip › FigEV/FigEV2/FigG/3.jpg]

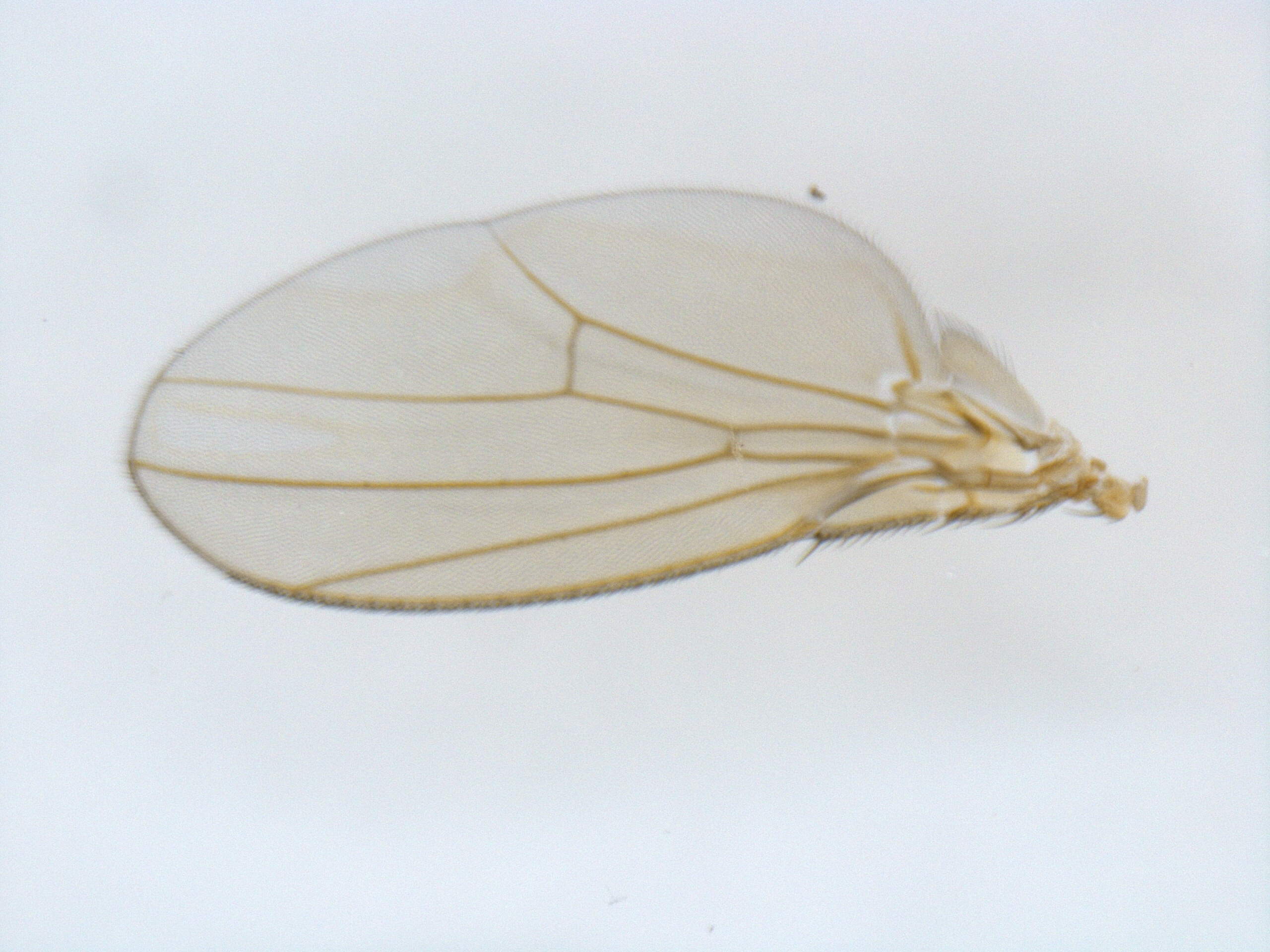

Supplement: Supplementary file 3 — Source Data for Expanded View [file EMBJ-42-e110454-s005.zip › FigEV/FigEV2/FigG/0.jpg]

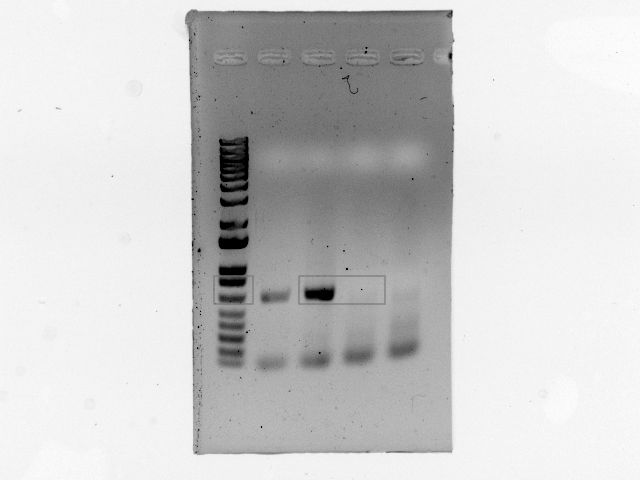

Supplement: Supplementary file 3 — Source Data for Expanded View [file EMBJ-42-e110454-s005.zip › FigEV/FigEV3/FigE/invert.tif]

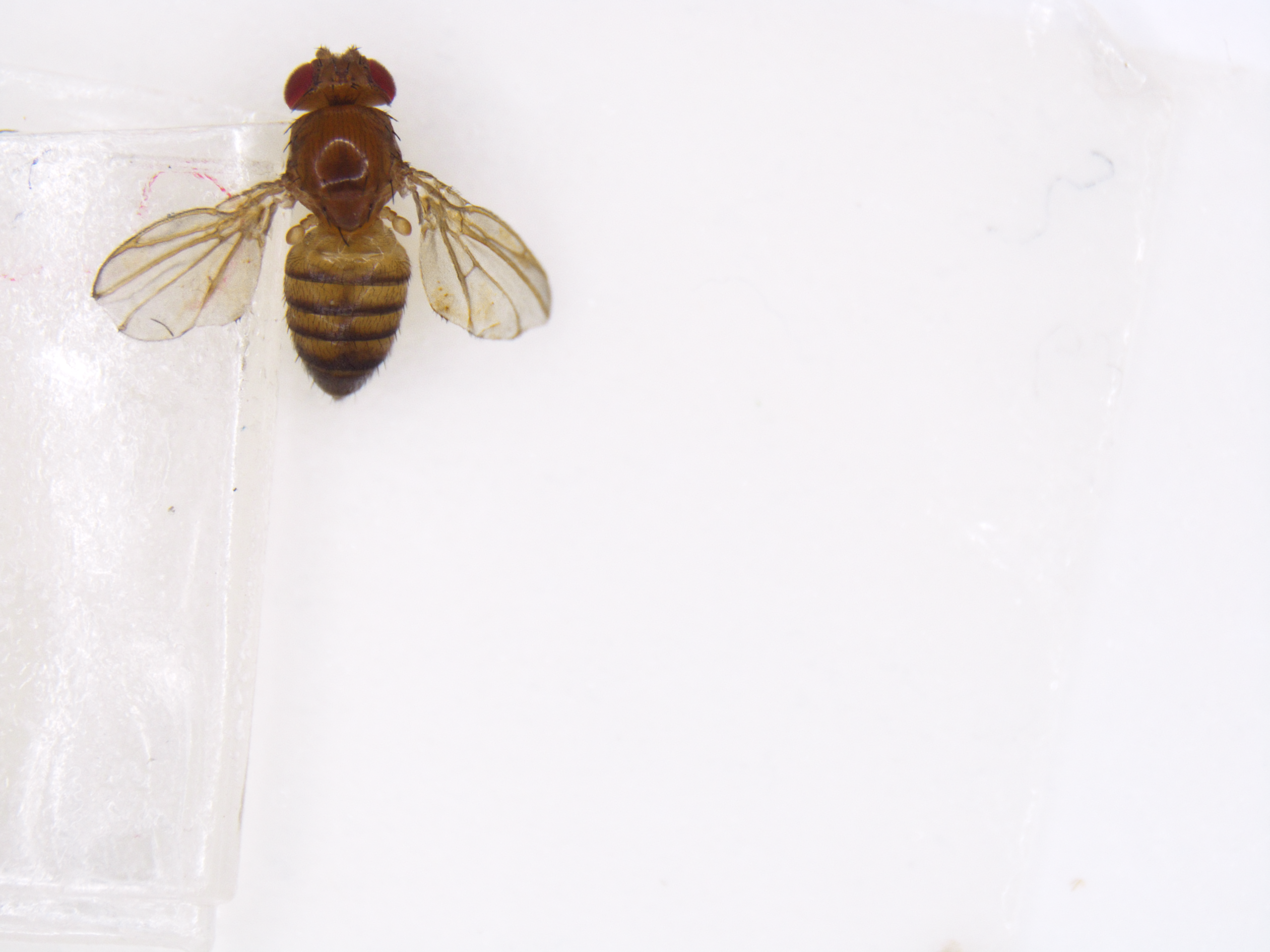

Supplement: Supplementary file 3 — Source Data for Expanded View [file EMBJ-42-e110454-s005.zip › FigEV/FigEV3/FigA/+.tif]

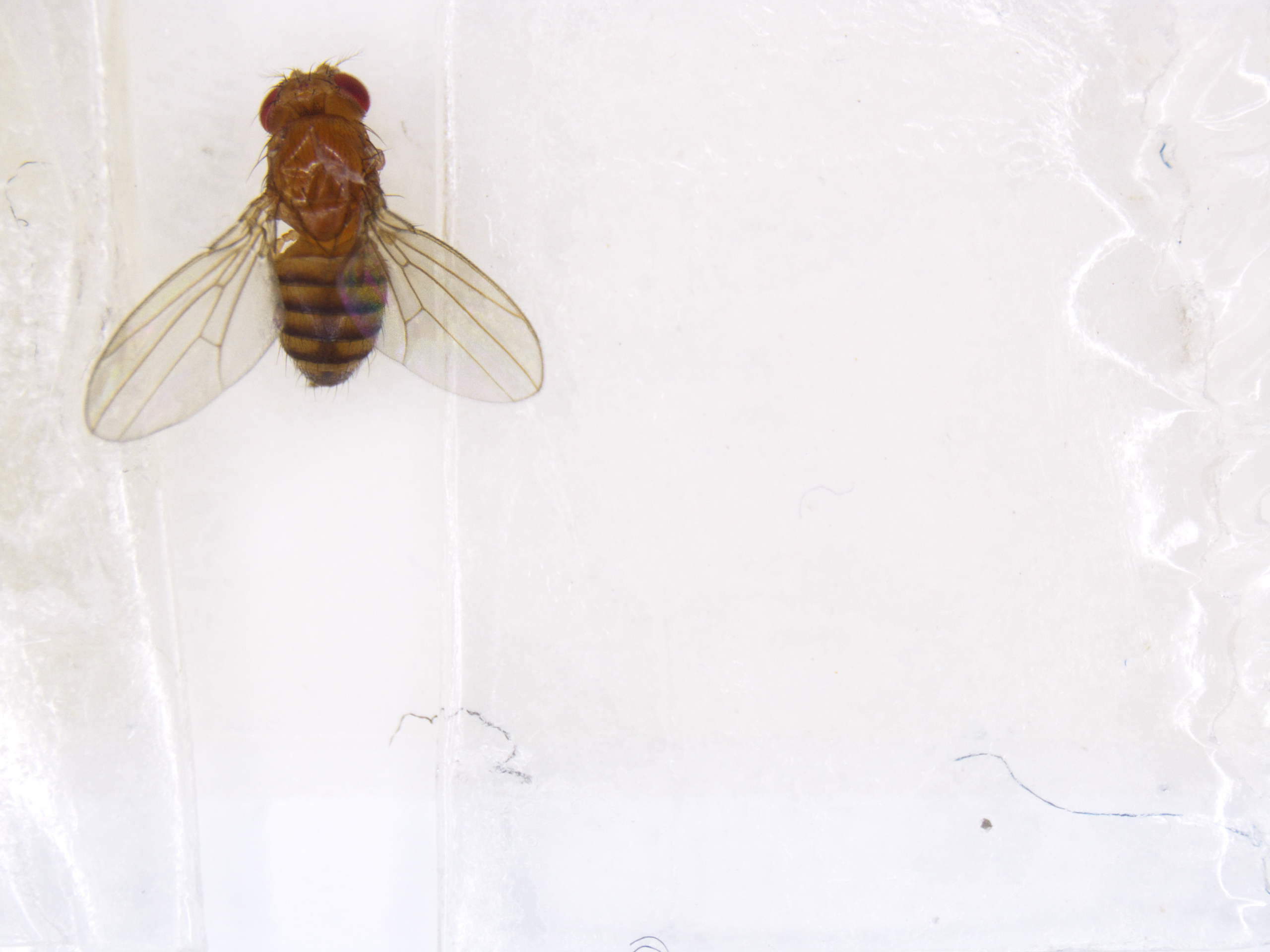

Supplement: Supplementary file 3 — Source Data for Expanded View [file EMBJ-42-e110454-s005.zip › FigEV/FigEV3/FigA/synr RNAi.tif]

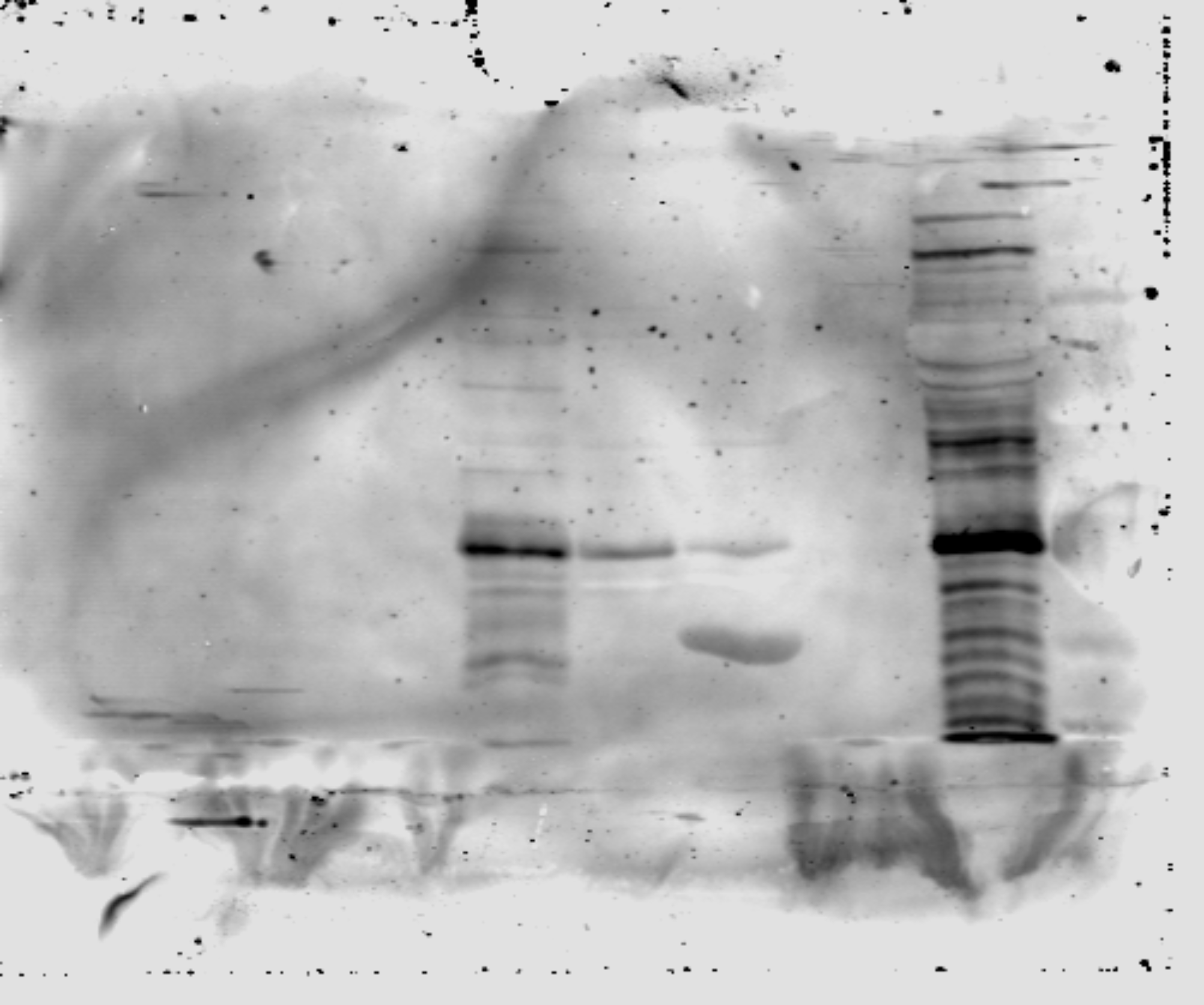

Supplement: Supplementary file 3 — Source Data for Expanded View [file EMBJ-42-e110454-s005.zip › FigEV/FigEV4/FigE/dborg0-3 800.tif]

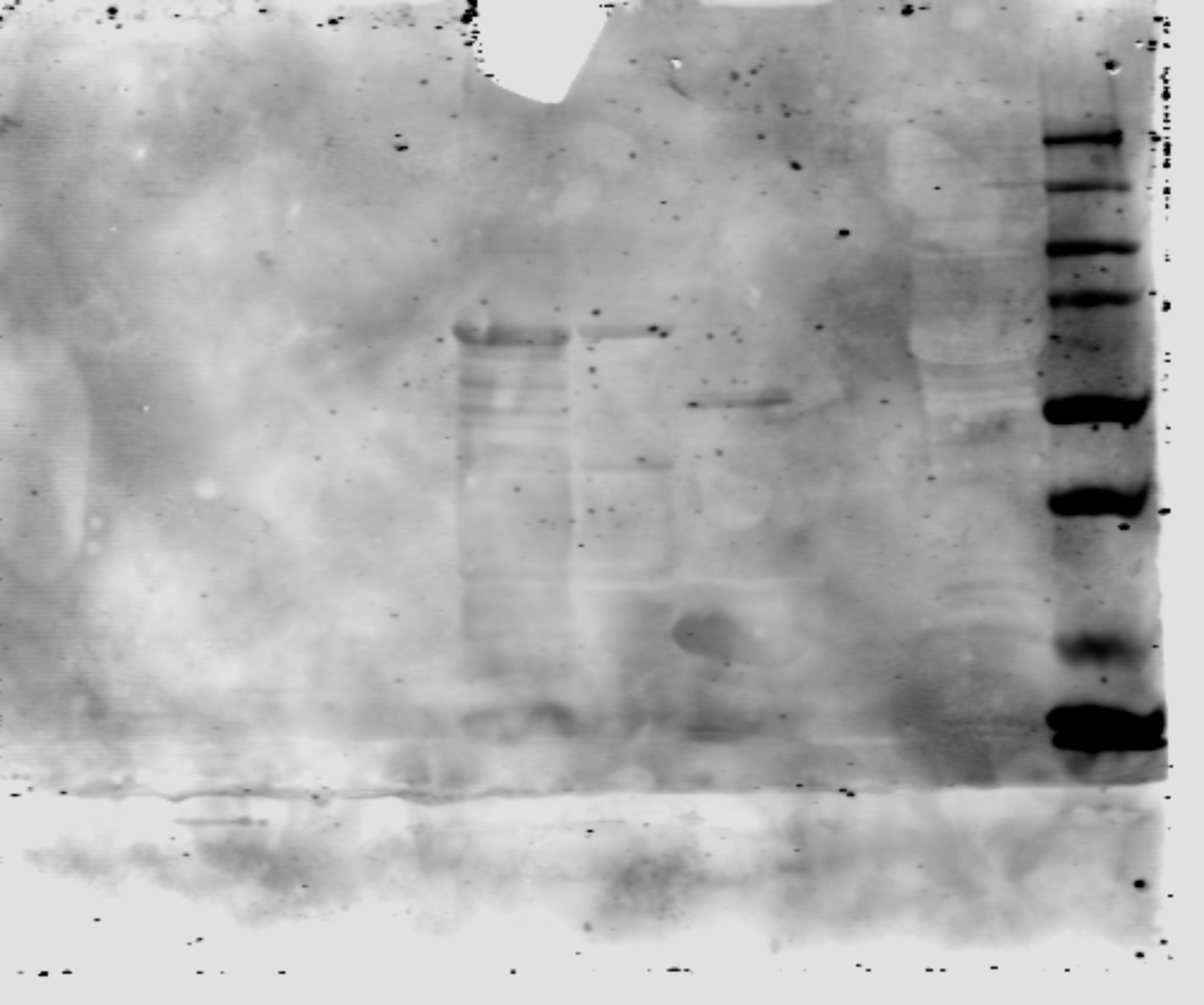

Supplement: Supplementary file 3 — Source Data for Expanded View [file EMBJ-42-e110454-s005.zip › FigEV/FigEV4/FigE/dborg0-3 700.tif]

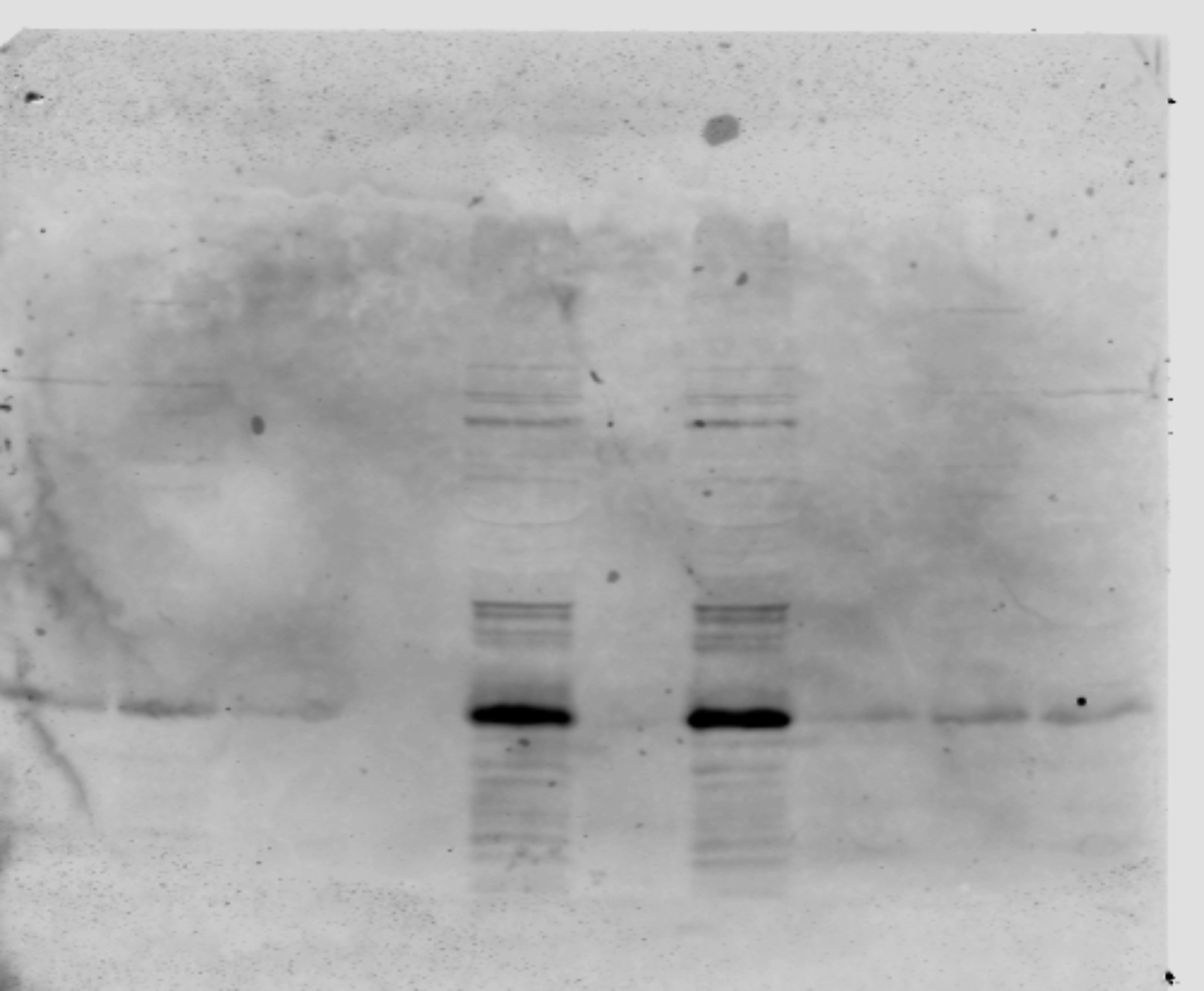

Supplement: Supplementary file 3 — Source Data for Expanded View [file EMBJ-42-e110454-s005.zip › FigEV/FigEV4/FigC/1_2-debcl.tif]

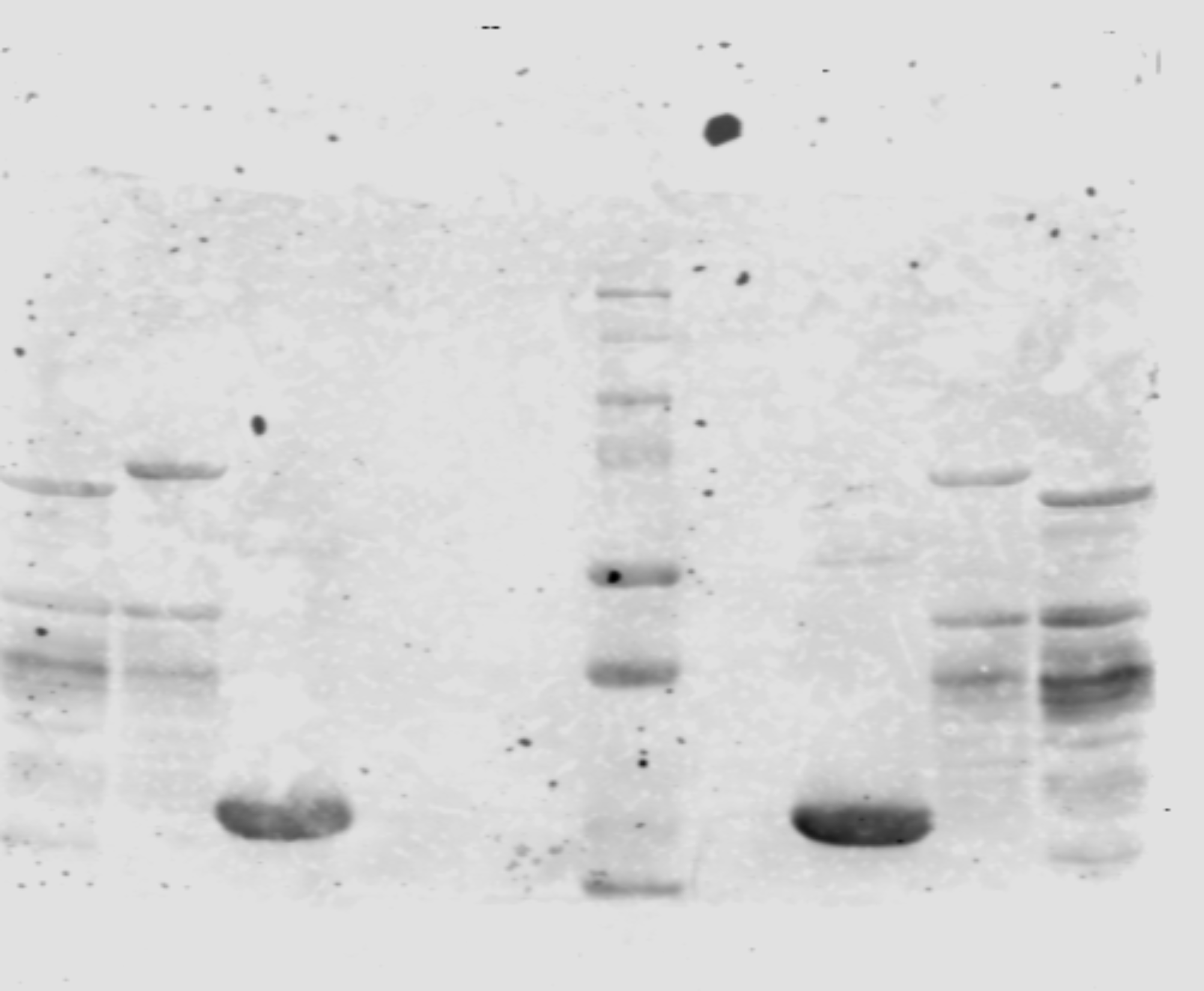

Supplement: Supplementary file 3 — Source Data for Expanded View [file EMBJ-42-e110454-s005.zip › FigEV/FigEV4/FigC/1_2-gst.tif]

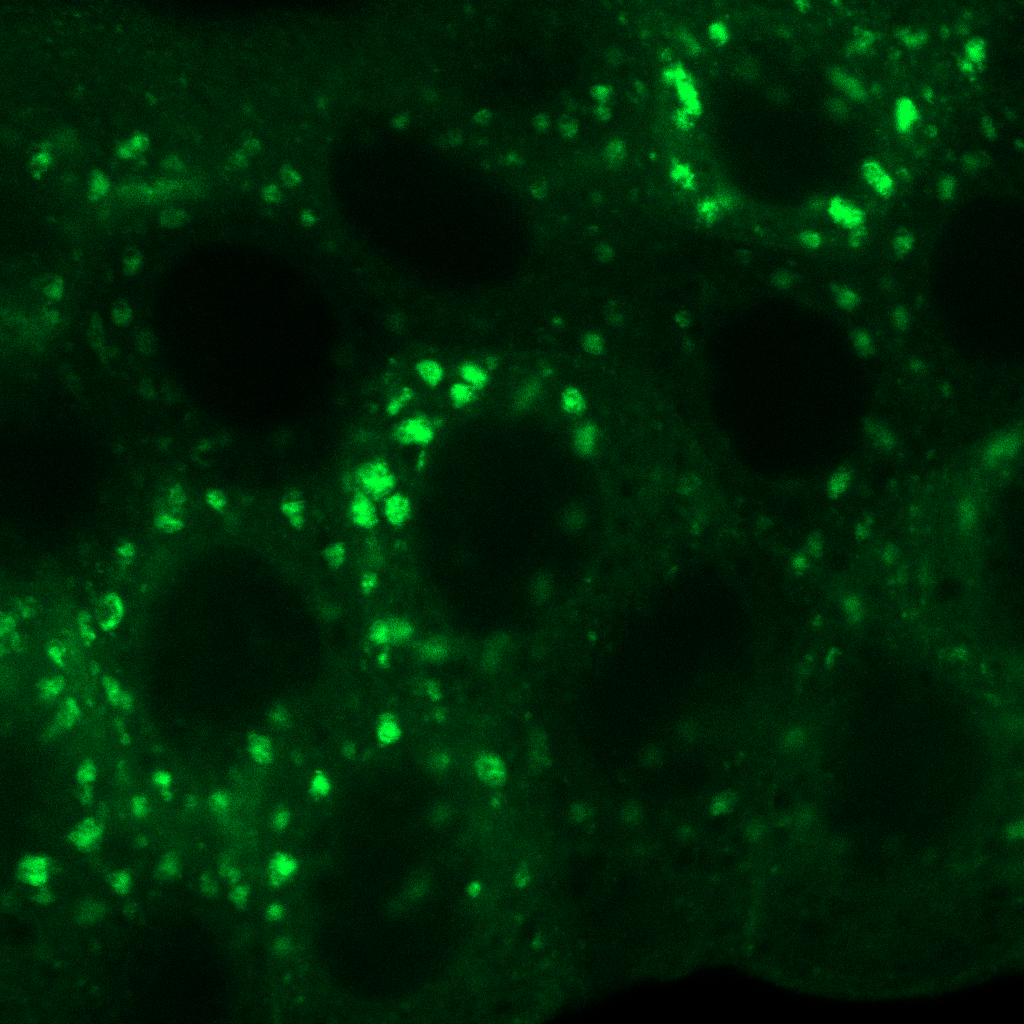

Supplement: Supplementary file 3 — Source Data for Expanded View [file EMBJ-42-e110454-s005.zip › FigEV/FigEV2/FigA/salivary gland/rab27.tif]

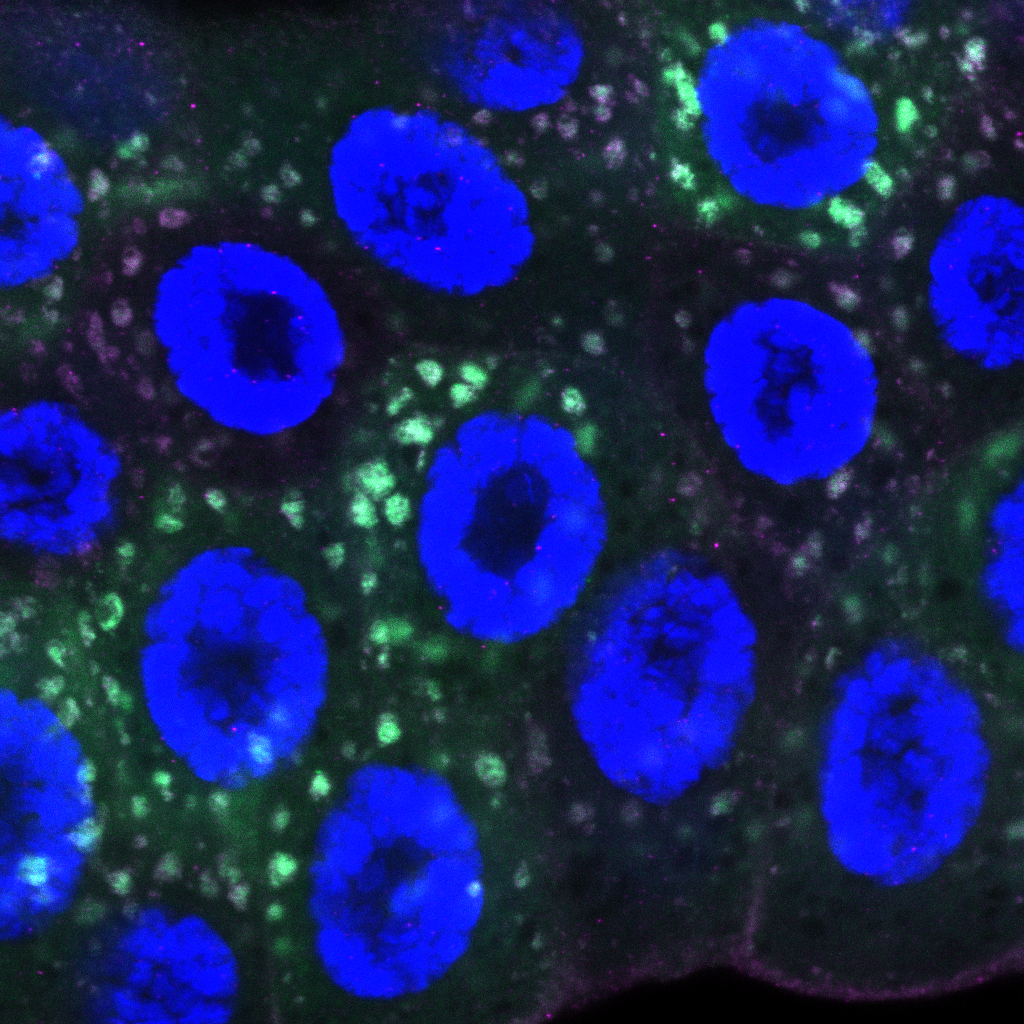

Supplement: Supplementary file 3 — Source Data for Expanded View [file EMBJ-42-e110454-s005.zip › FigEV/FigEV2/FigA/salivary gland/merge.tif]

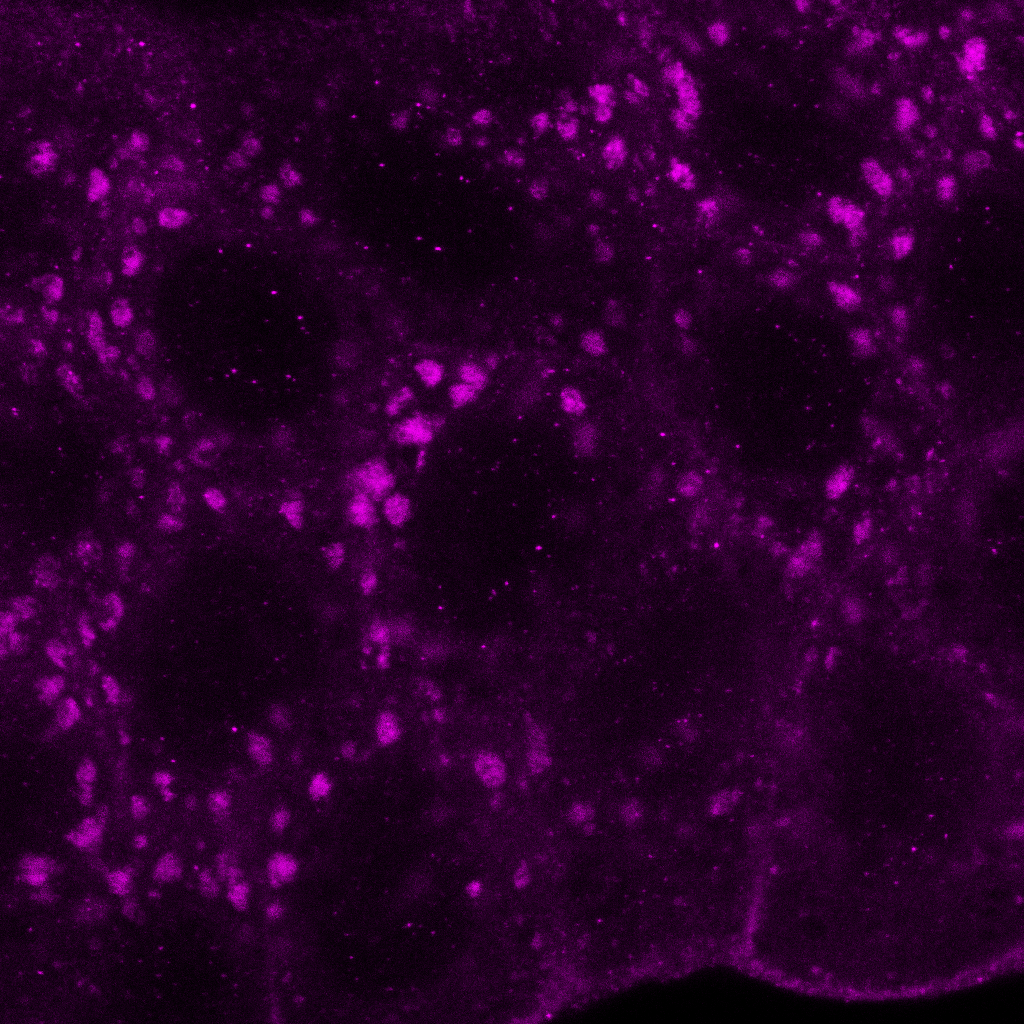

Supplement: Supplementary file 3 — Source Data for Expanded View [file EMBJ-42-e110454-s005.zip › FigEV/FigEV2/FigA/salivary gland/synr HA.tif]

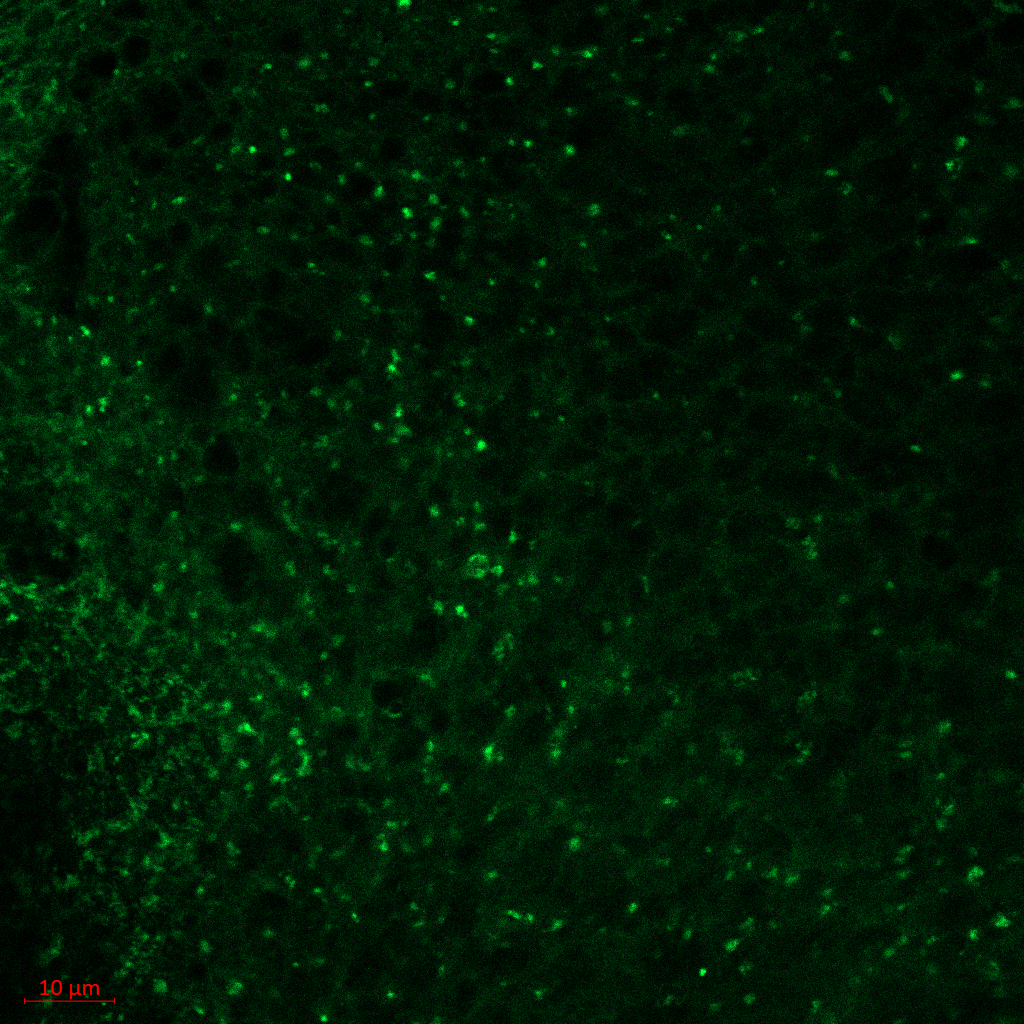

Supplement: Supplementary file 3 — Source Data for Expanded View [file EMBJ-42-e110454-s005.zip › FigEV/FigEV2/FigA/wing disc/rab27.tif]

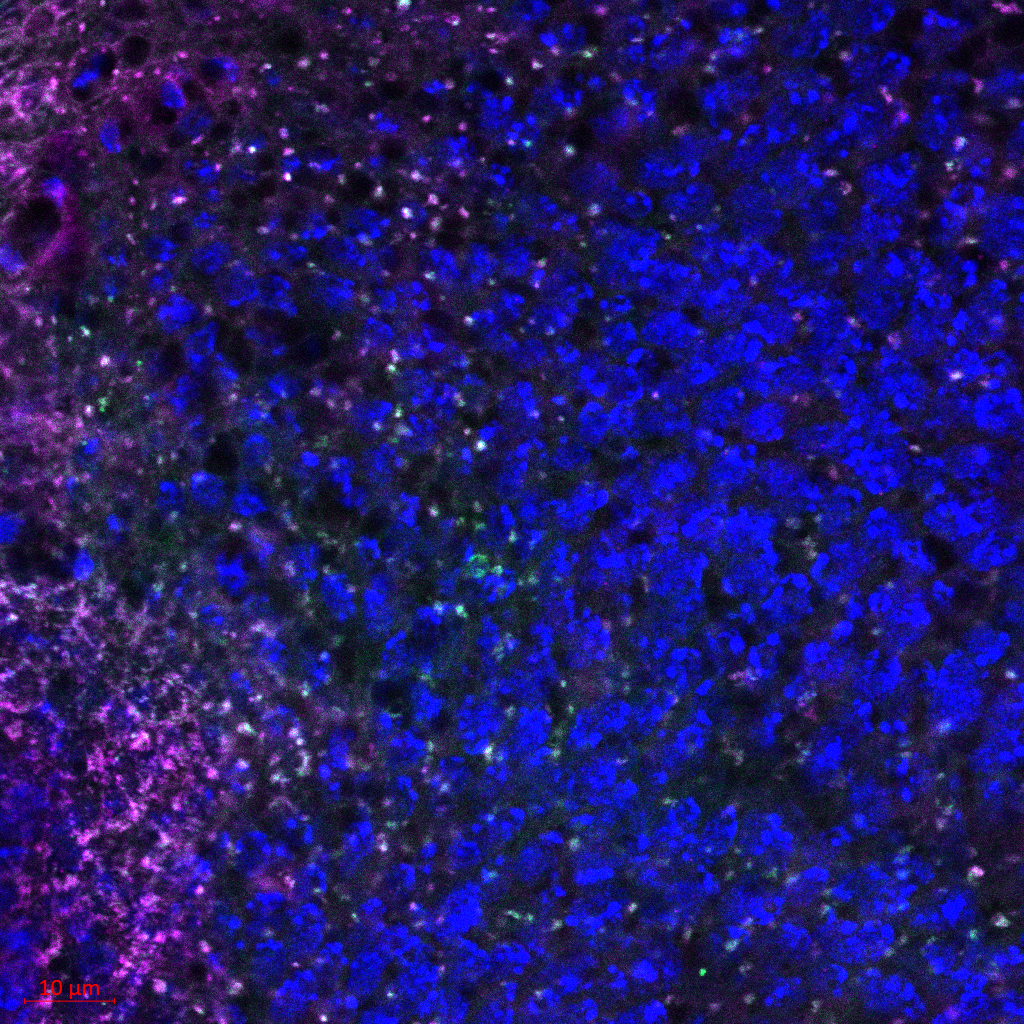

Supplement: Supplementary file 3 — Source Data for Expanded View [file EMBJ-42-e110454-s005.zip › FigEV/FigEV2/FigA/wing disc/merge.tif]

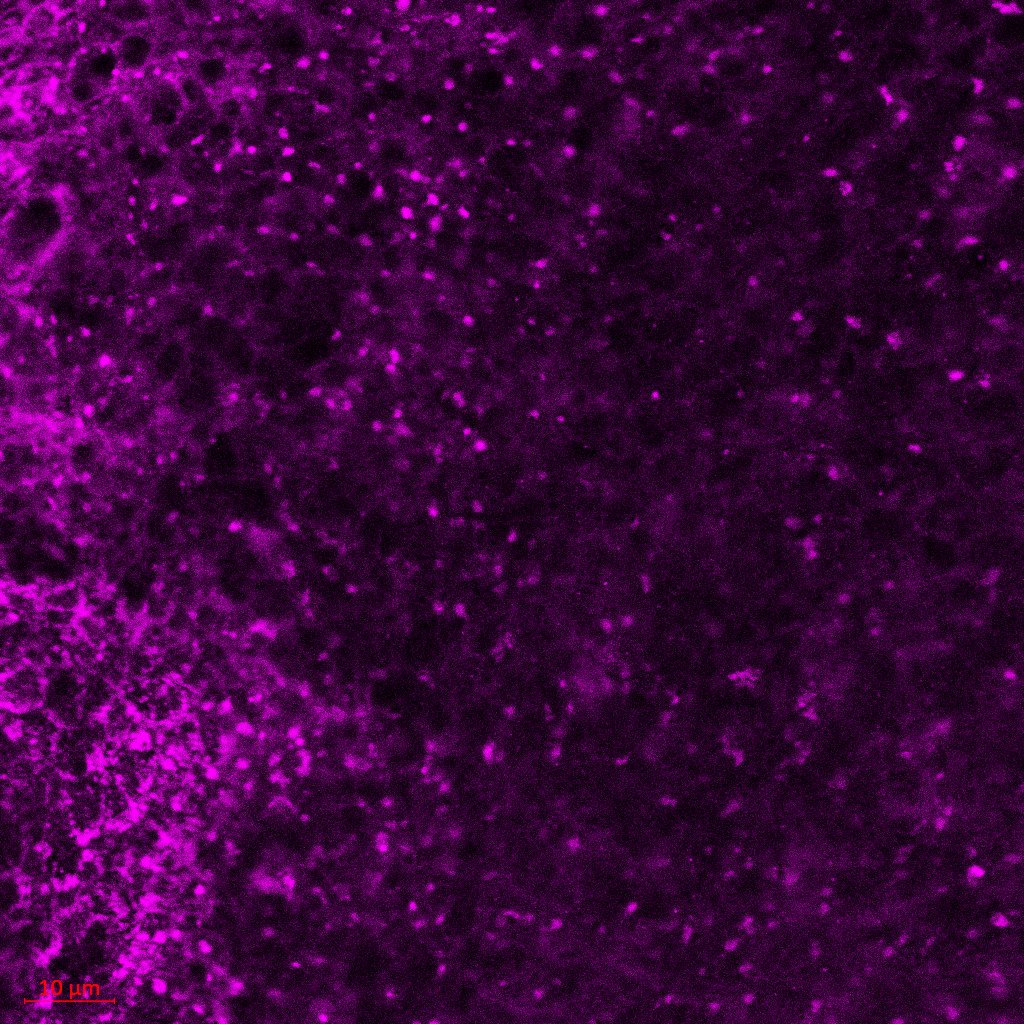

Supplement: Supplementary file 3 — Source Data for Expanded View [file EMBJ-42-e110454-s005.zip › FigEV/FigEV2/FigA/wing disc/synr HA.tif]

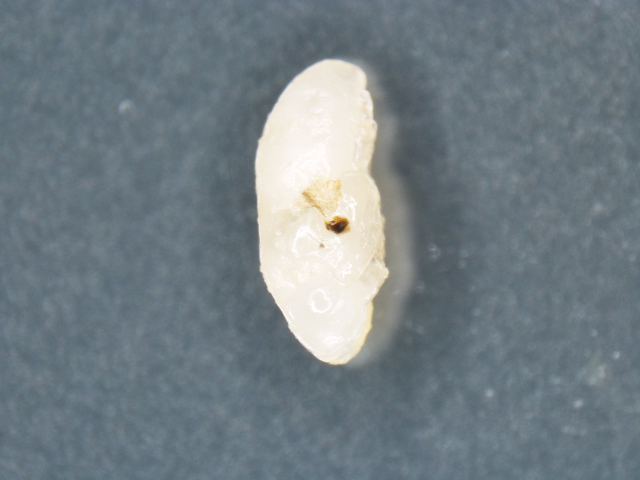

Supplement: Supplementary file 5 — Source Data for Figure 1 [file EMBJ-42-e110454-s003.zip › Fig1/Fig1-E/pupae/CG14044.tif]

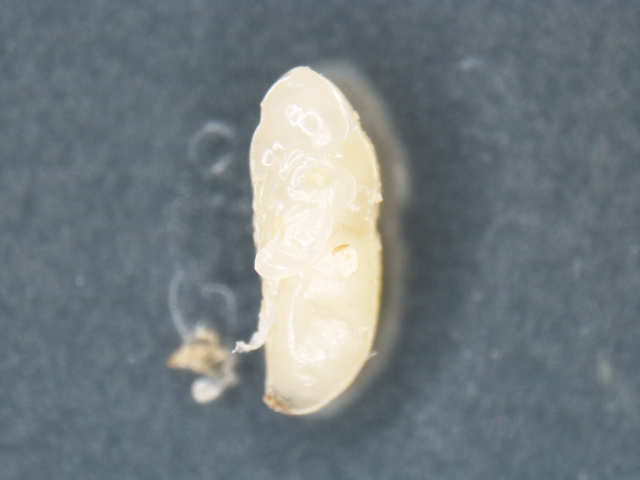

Supplement: Supplementary file 5 — Source Data for Figure 1 [file EMBJ-42-e110454-s003.zip › Fig1/Fig1-E/pupae/+.tif]

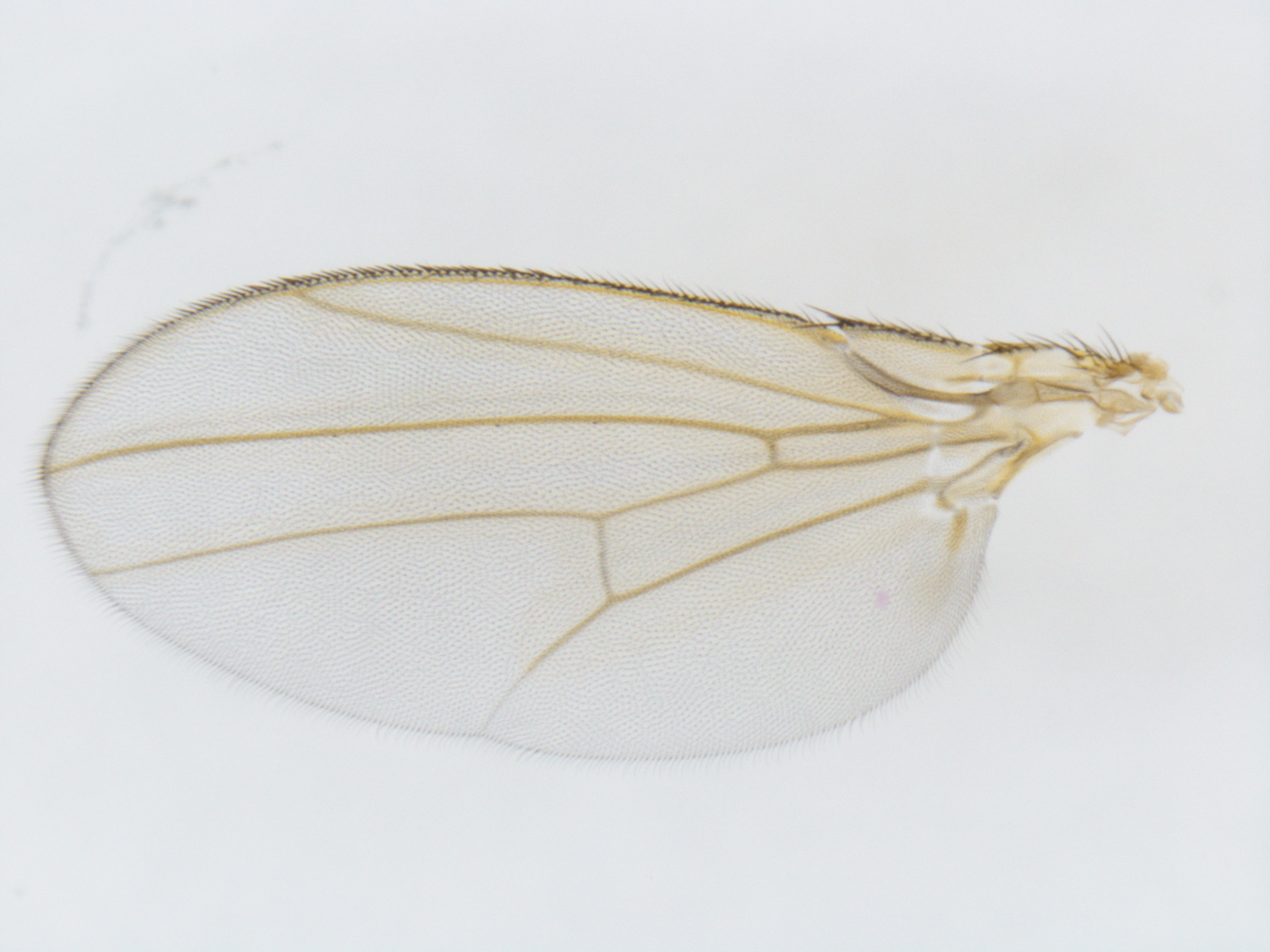

Supplement: Supplementary file 5 — Source Data for Figure 1 [file EMBJ-42-e110454-s003.zip › Fig1/Fig1-E/adult wing/+.tif]

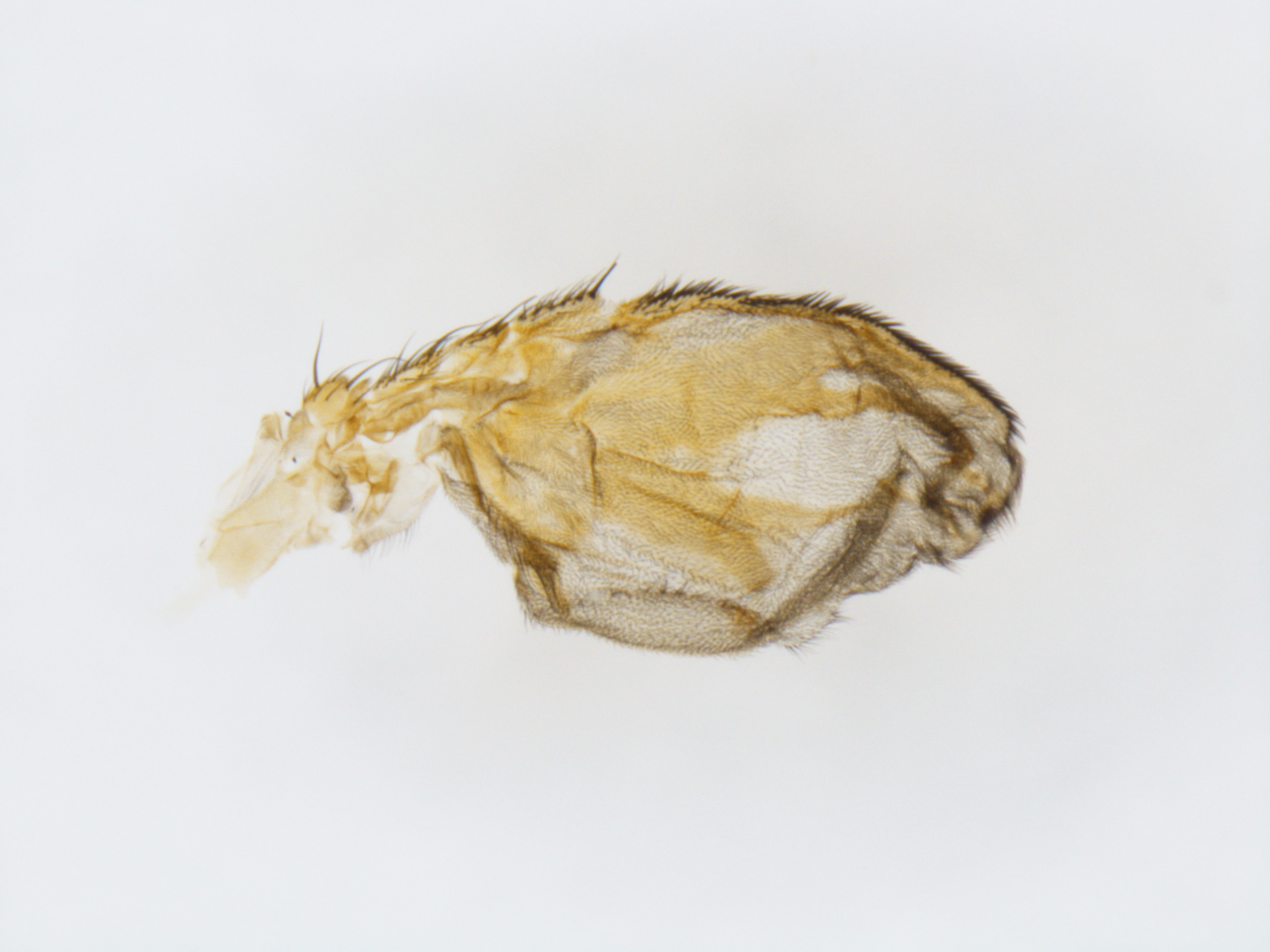

Supplement: Supplementary file 5 — Source Data for Figure 1 [file EMBJ-42-e110454-s003.zip › Fig1/Fig1-E/adult wing/CG14044.tif]

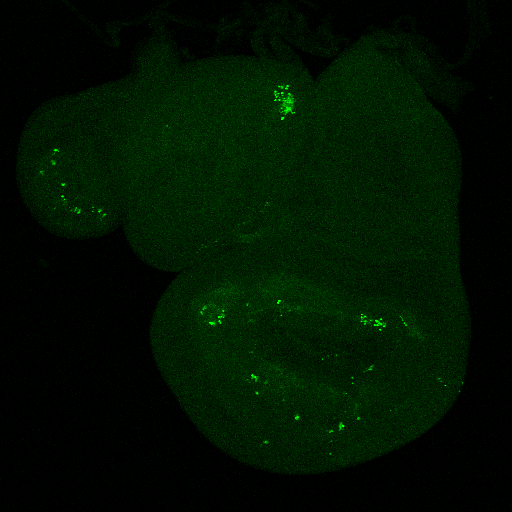

Supplement: Supplementary file 5 — Source Data for Figure 1 [file EMBJ-42-e110454-s003.zip › Fig1/Fig1-H/+/14-Orthogonal Projection-04_c2.tif]

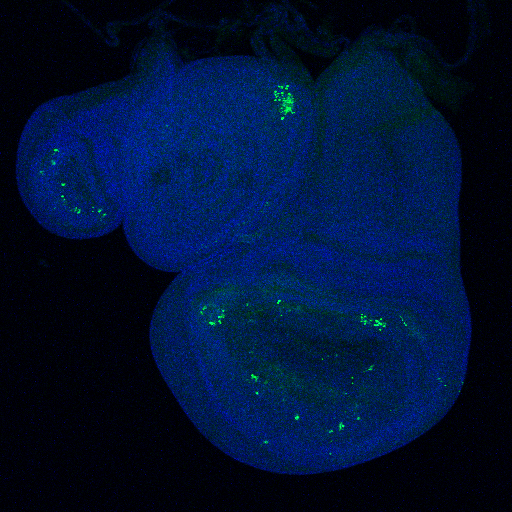

Supplement: Supplementary file 5 — Source Data for Figure 1 [file EMBJ-42-e110454-s003.zip › Fig1/Fig1-H/+/14-Orthogonal Projection-04_c1+2.tif]

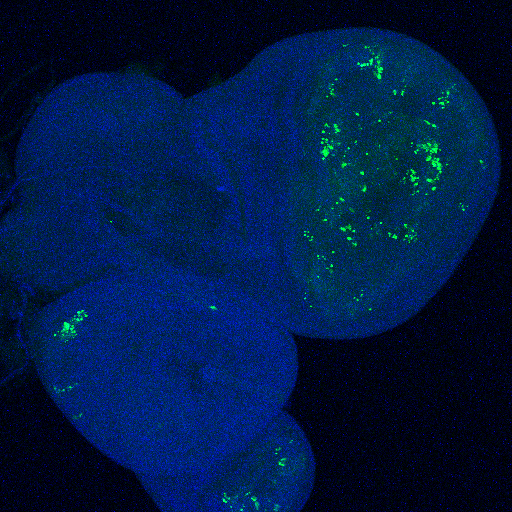

Supplement: Supplementary file 5 — Source Data for Figure 1 [file EMBJ-42-e110454-s003.zip › Fig1/Fig1-H/synr WT/7-Orthogonal Projection-02_c1+2.tif]

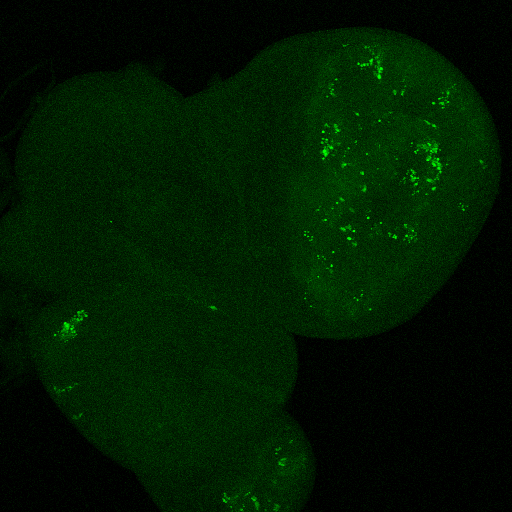

Supplement: Supplementary file 5 — Source Data for Figure 1 [file EMBJ-42-e110454-s003.zip › Fig1/Fig1-H/synr WT/7-Orthogonal Projection-02_c2.tif]

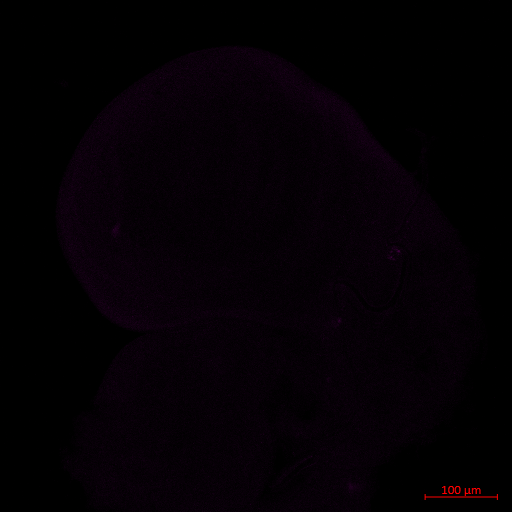

Supplement: Supplementary file 5 — Source Data for Figure 1 [file EMBJ-42-e110454-s003.zip › Fig1/Fig1-E/Dcp-1 staining/+/nub-GFP wing disc DCP1 1_c1.tif]

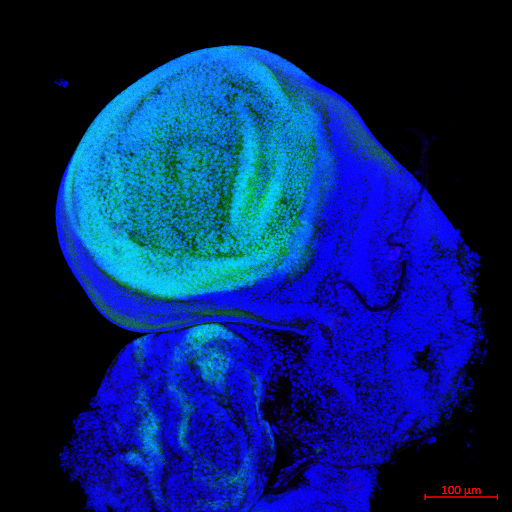

Supplement: Supplementary file 5 — Source Data for Figure 1 [file EMBJ-42-e110454-s003.zip › Fig1/Fig1-E/Dcp-1 staining/+/nub-GFP wing disc DCP1 1_c1+2+3.tif]

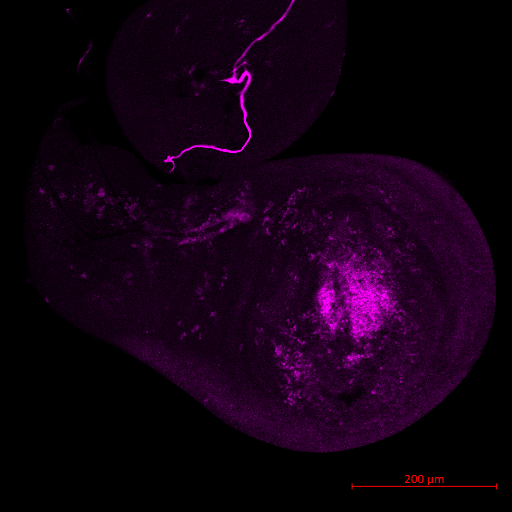

Supplement: Supplementary file 5 — Source Data for Figure 1 [file EMBJ-42-e110454-s003.zip › Fig1/Fig1-E/Dcp-1 staining/CG14044/nub-CG14044 wing disc DCP1 1_c1.tif]

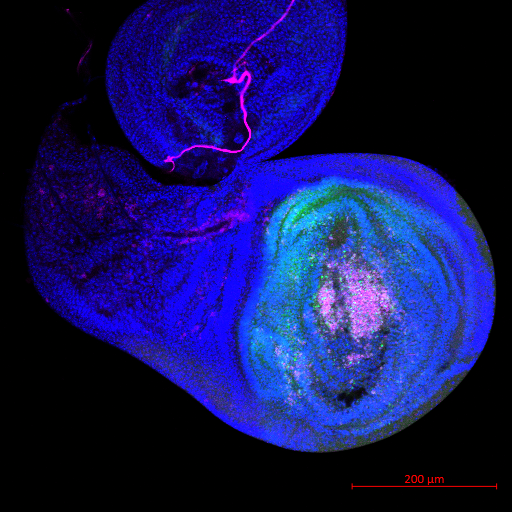

Supplement: Supplementary file 5 — Source Data for Figure 1 [file EMBJ-42-e110454-s003.zip › Fig1/Fig1-E/Dcp-1 staining/CG14044/nub-CG14044 wing disc DCP1 1_c1+2+3.tif]

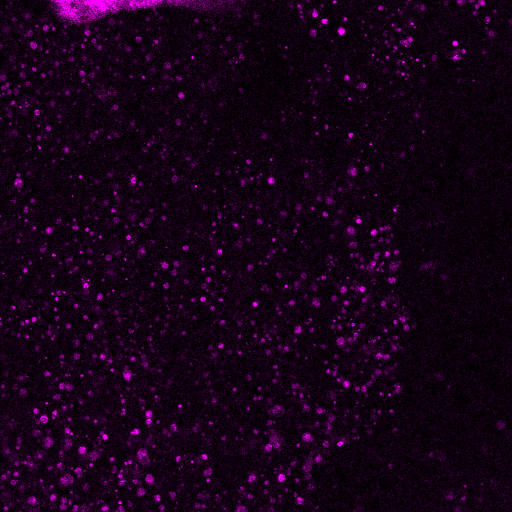

Supplement: Supplementary file 5 — Source Data for Figure 1 [file EMBJ-42-e110454-s003.zip › Fig1/Fig1-E/tunel/CG14044/CG14044-Orthogonal Projection-01_c1.tif]

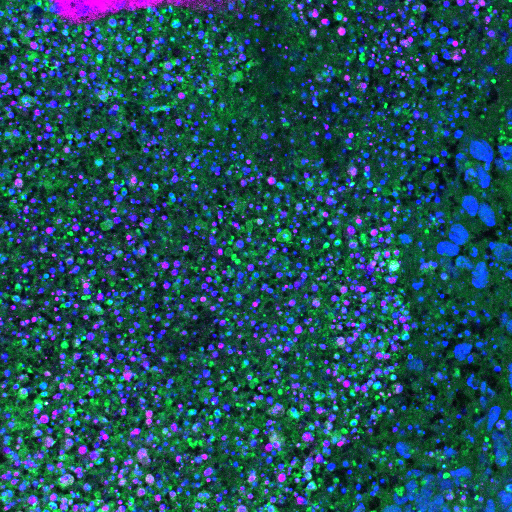

Supplement: Supplementary file 5 — Source Data for Figure 1 [file EMBJ-42-e110454-s003.zip › Fig1/Fig1-E/tunel/CG14044/CG14044-Orthogonal Projection-01_c1+2+3.tif]

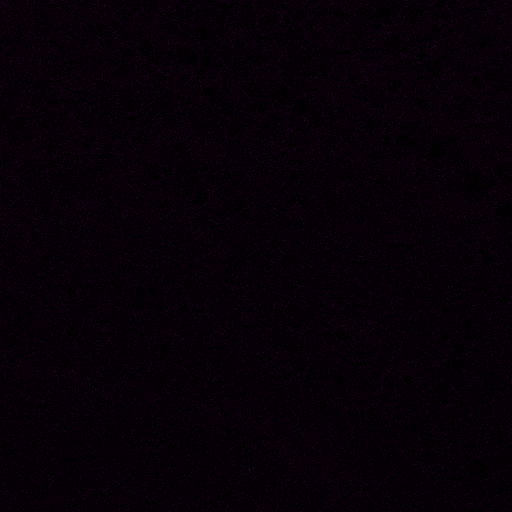

Supplement: Supplementary file 5 — Source Data for Figure 1 [file EMBJ-42-e110454-s003.zip › Fig1/Fig1-E/tunel/+/4-Orthogonal Projection-03_c1.tif]

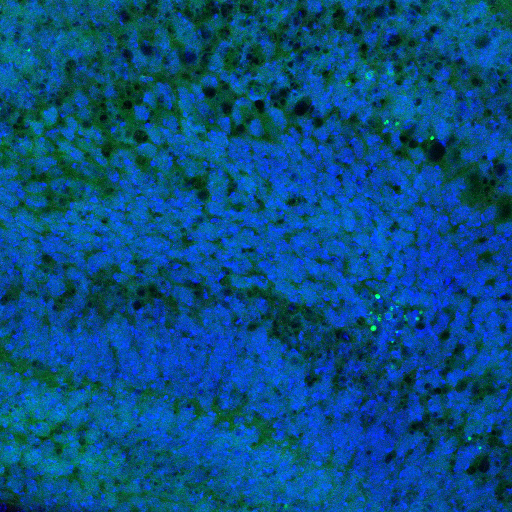

Supplement: Supplementary file 5 — Source Data for Figure 1 [file EMBJ-42-e110454-s003.zip › Fig1/Fig1-E/tunel/+/4-Orthogonal Projection-03_c1+2+3.tif]

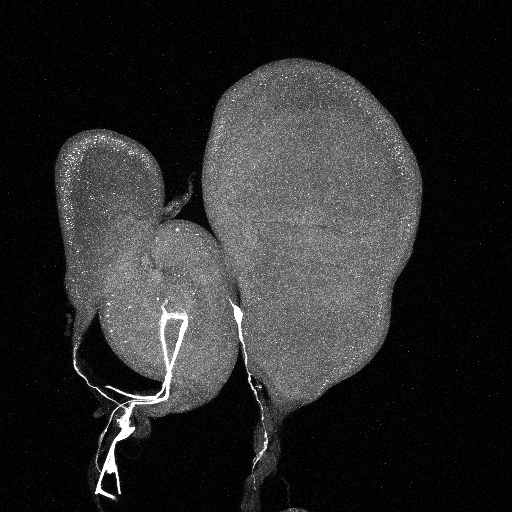

Supplement: Supplementary file 6 — Source Data for Figure 2 [file EMBJ-42-e110454-s001.zip › Fig2/FigF/debcl-Orthogonal Projection-08_c1.tif]

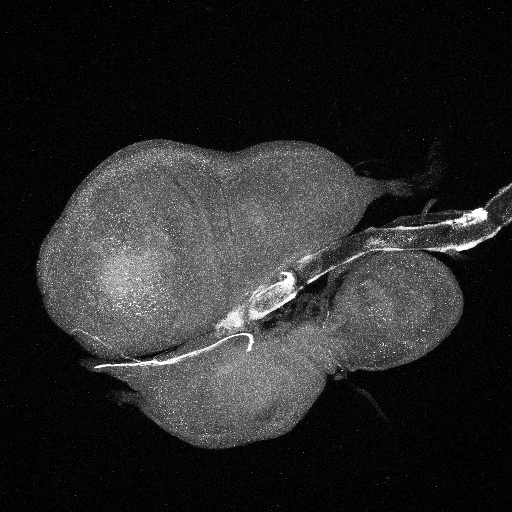

Supplement: Supplementary file 6 — Source Data for Figure 2 [file EMBJ-42-e110454-s001.zip › Fig2/FigF/OR-2-Orthogonal Projection-07_c1.tif]

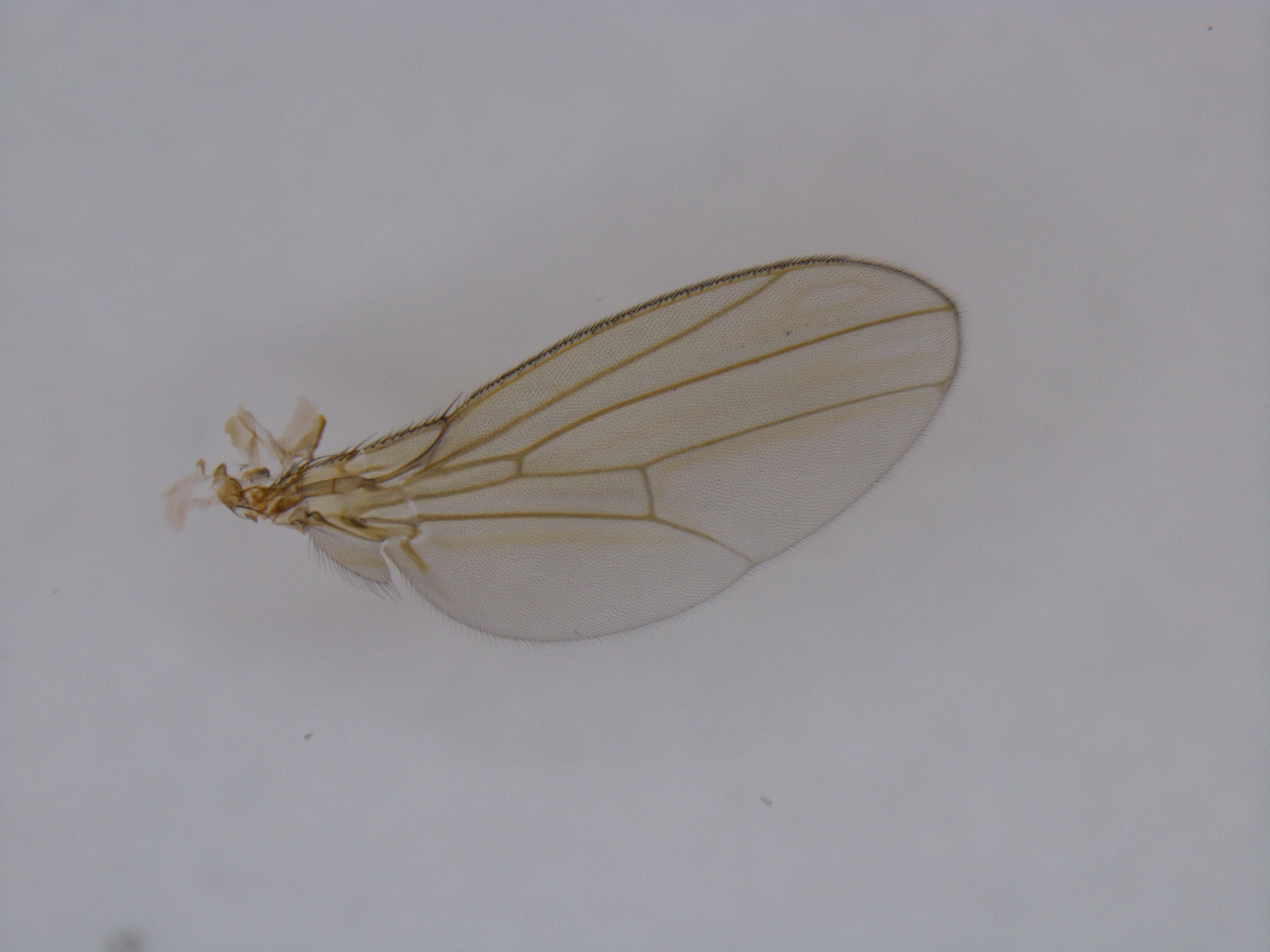

Supplement: Supplementary file 6 — Source Data for Figure 2 [file EMBJ-42-e110454-s001.zip › Fig2/FigA-D/buffy 29608.tif]

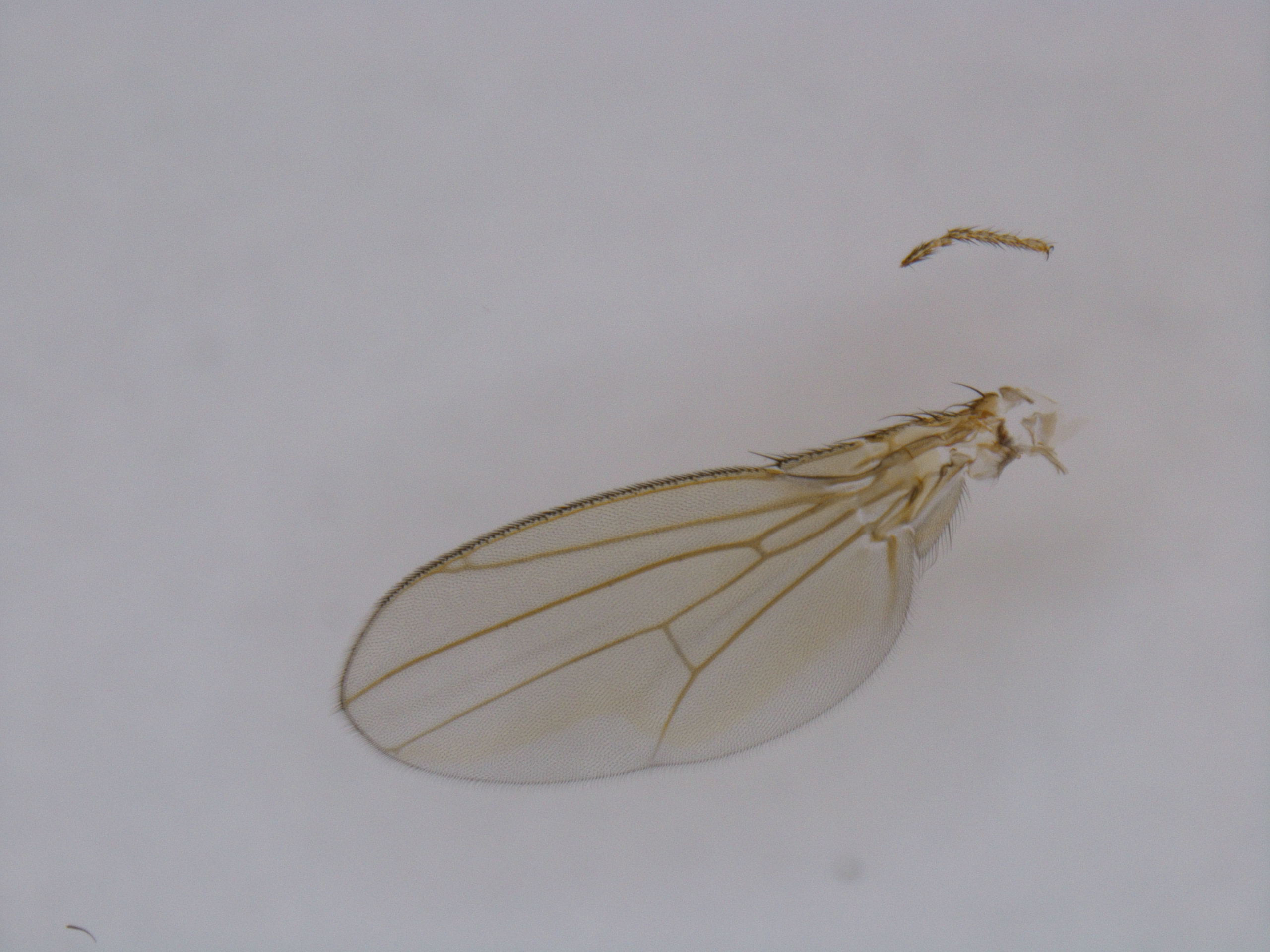

Supplement: Supplementary file 6 — Source Data for Figure 2 [file EMBJ-42-e110454-s001.zip › Fig2/FigA-D/dcp1 28909.tif]

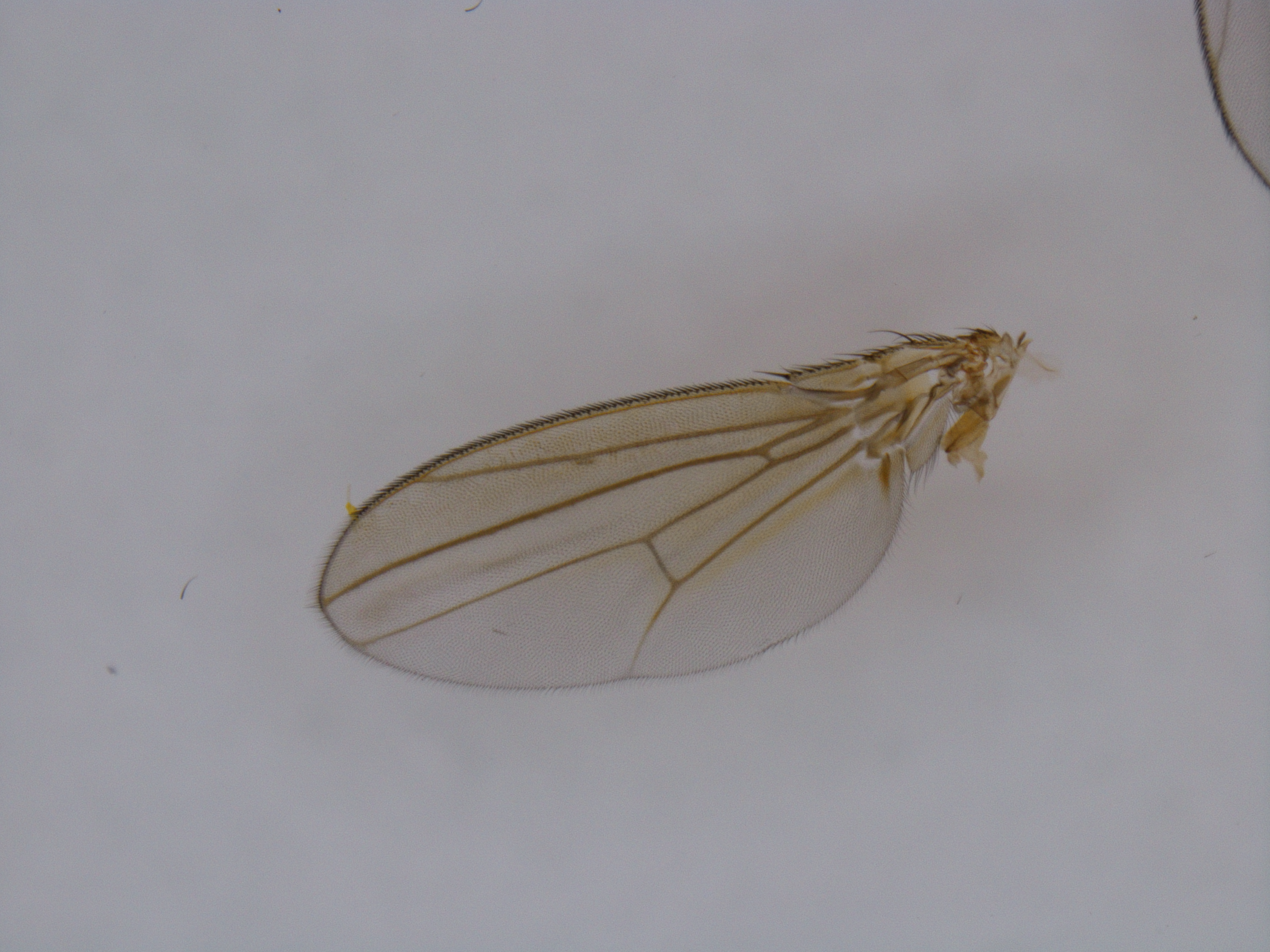

Supplement: Supplementary file 6 — Source Data for Figure 2 [file EMBJ-42-e110454-s001.zip › Fig2/FigA-D/debcl27083.tif]

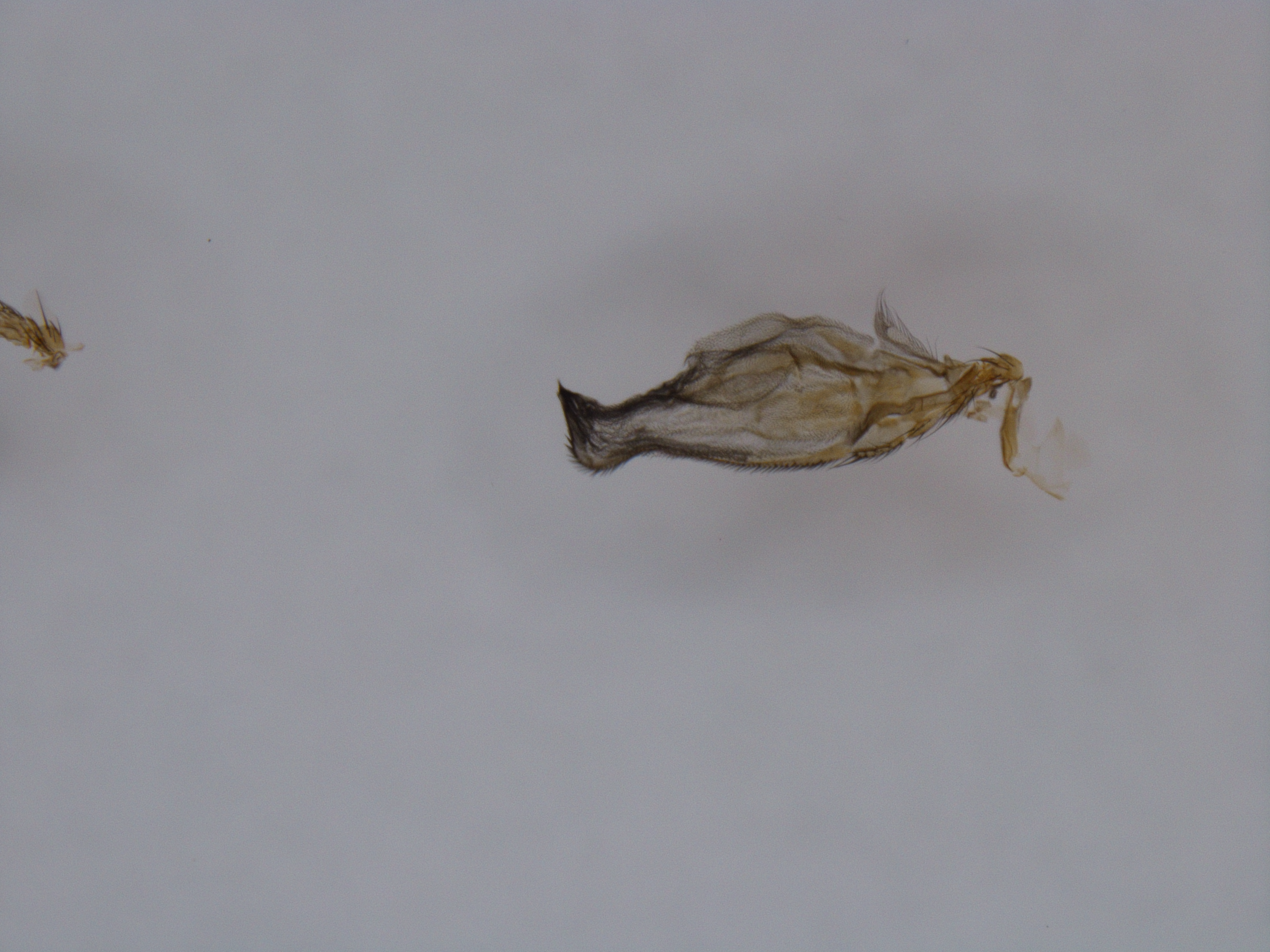

Supplement: Supplementary file 6 — Source Data for Figure 2 [file EMBJ-42-e110454-s001.zip › Fig2/FigA-D/mcherry.tif]

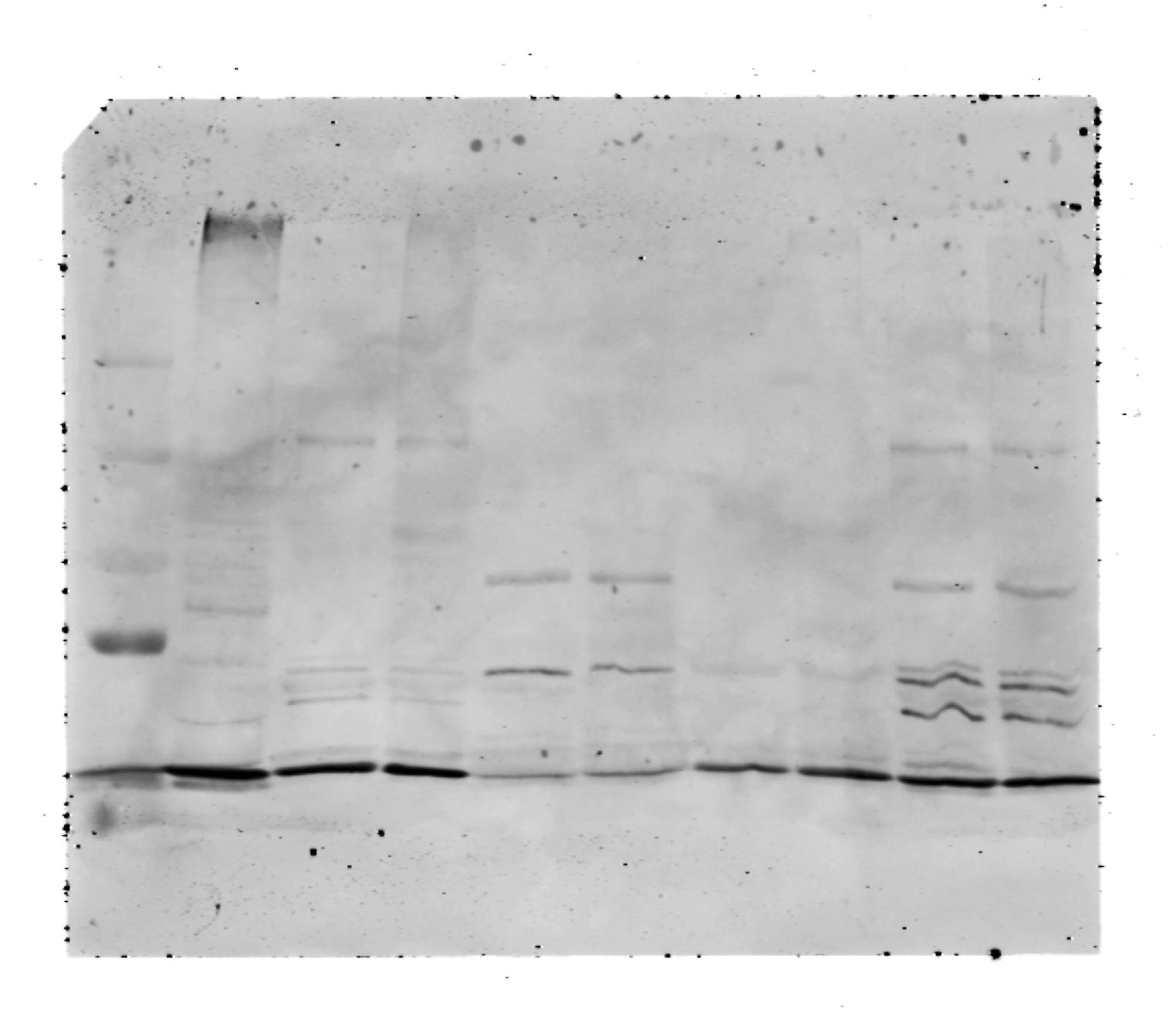

Supplement: Supplementary file 6 — Source Data for Figure 2 [file EMBJ-42-e110454-s001.zip › Fig2/FigO/141,340.tif]

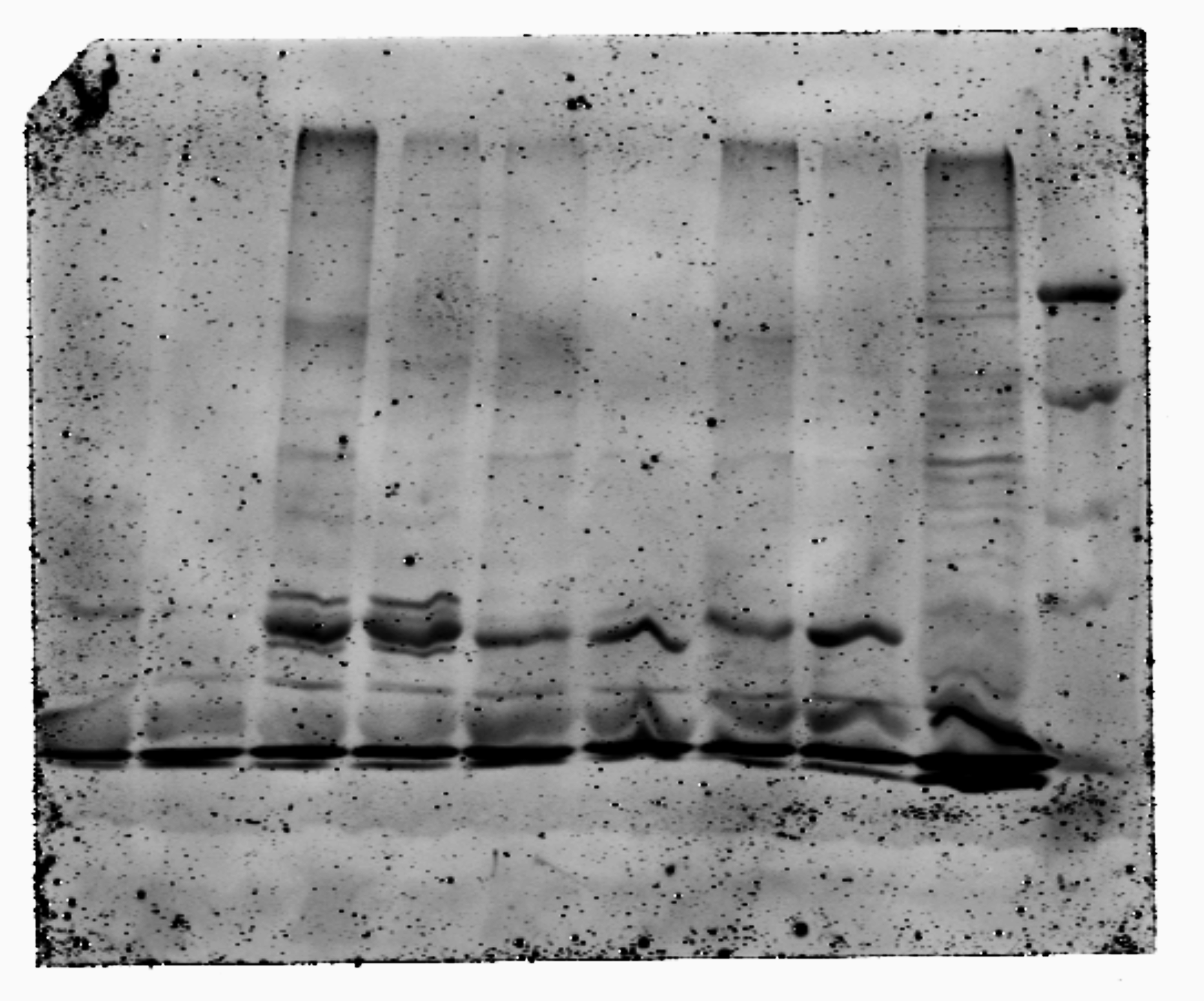

Supplement: Supplementary file 6 — Source Data for Figure 2 [file EMBJ-42-e110454-s001.zip › Fig2/FigO/124.tif]

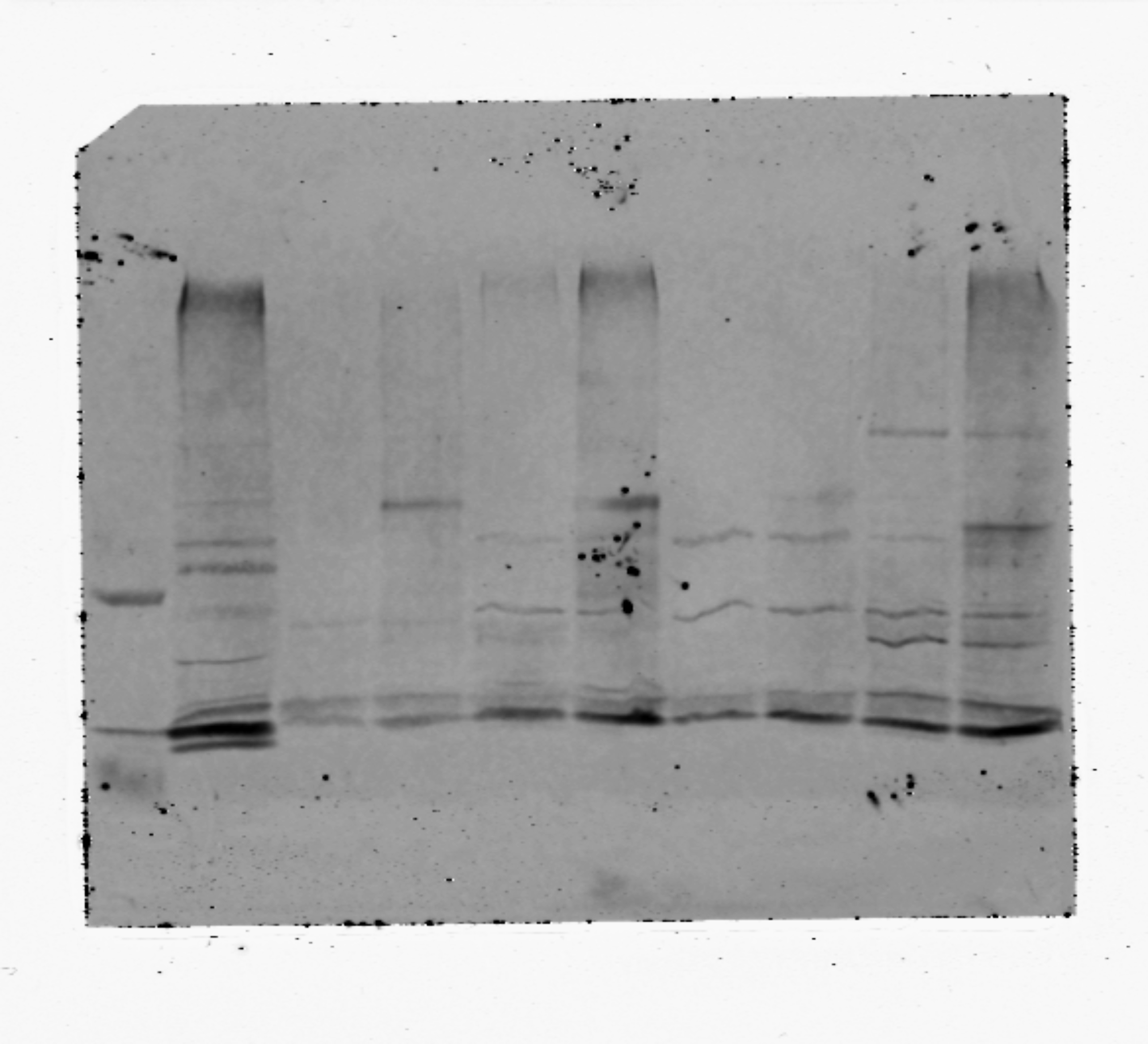

Supplement: Supplementary file 6 — Source Data for Figure 2 [file EMBJ-42-e110454-s001.zip › Fig2/FigP/141.tif]

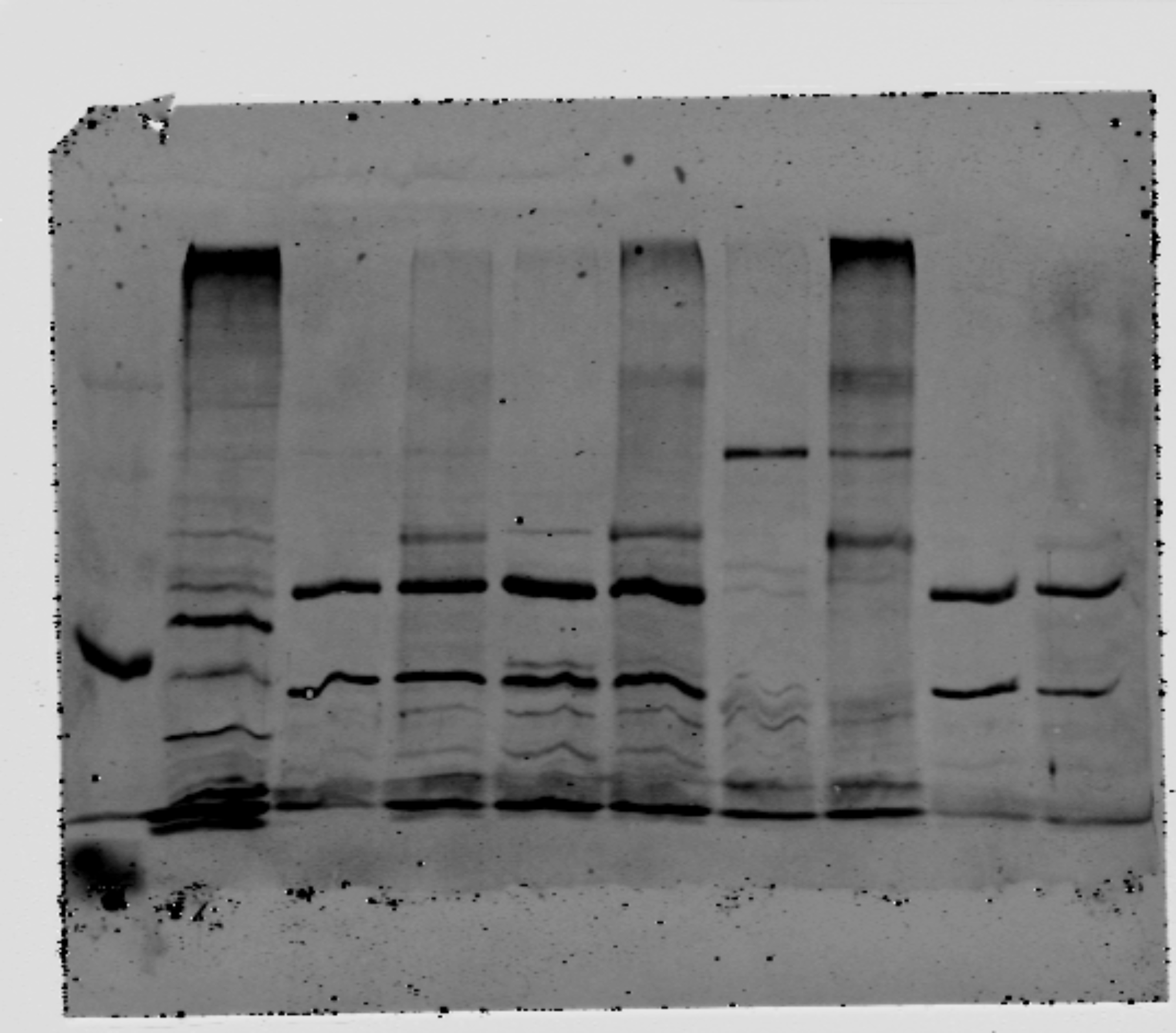

Supplement: Supplementary file 6 — Source Data for Figure 2 [file EMBJ-42-e110454-s001.zip › Fig2/FigP/124.tif]

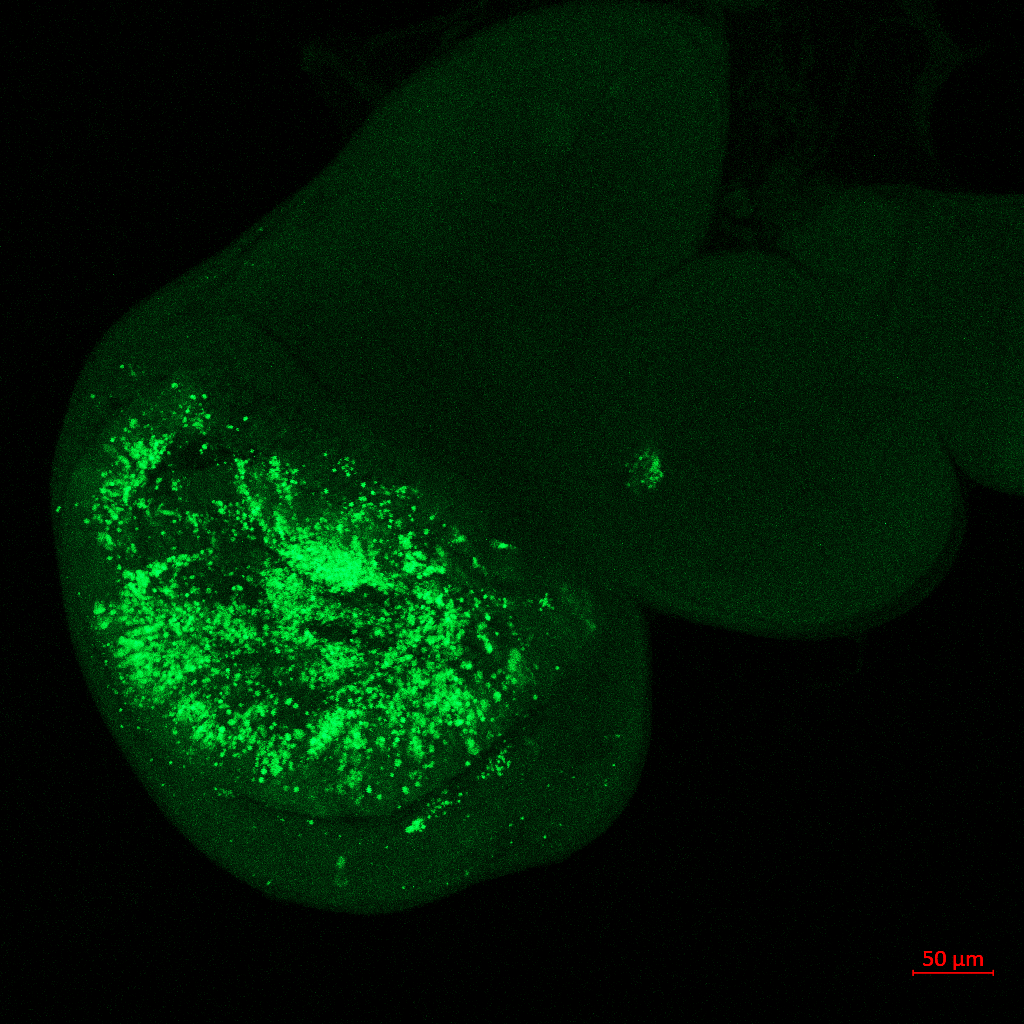

Supplement: Supplementary file 6 — Source Data for Figure 2 [file EMBJ-42-e110454-s001.zip › Fig2/FigG/luciferase RNAi.tif]

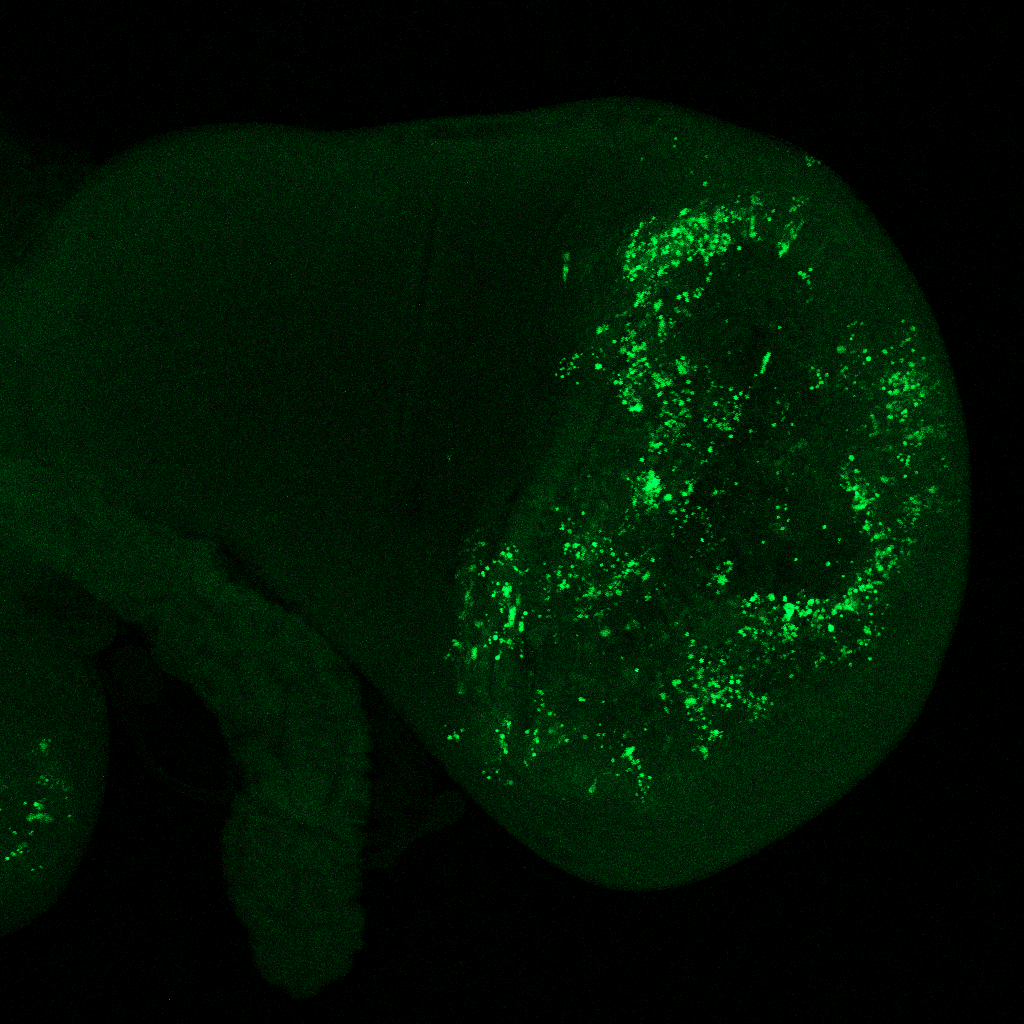

Supplement: Supplementary file 6 — Source Data for Figure 2 [file EMBJ-42-e110454-s001.zip › Fig2/FigG/Debcl RNAi.tif]

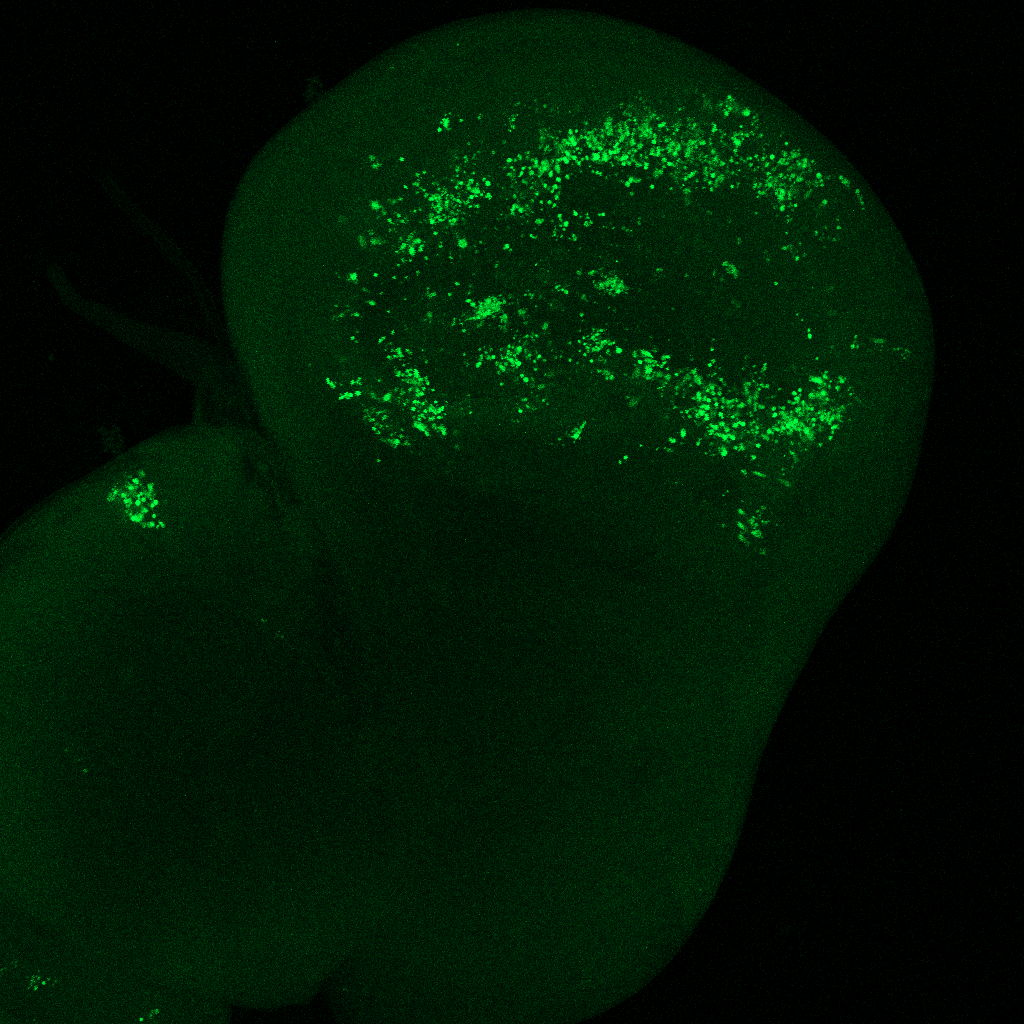

Supplement: Supplementary file 6 — Source Data for Figure 2 [file EMBJ-42-e110454-s001.zip › Fig2/FigG/BuffyRNAi.tif]

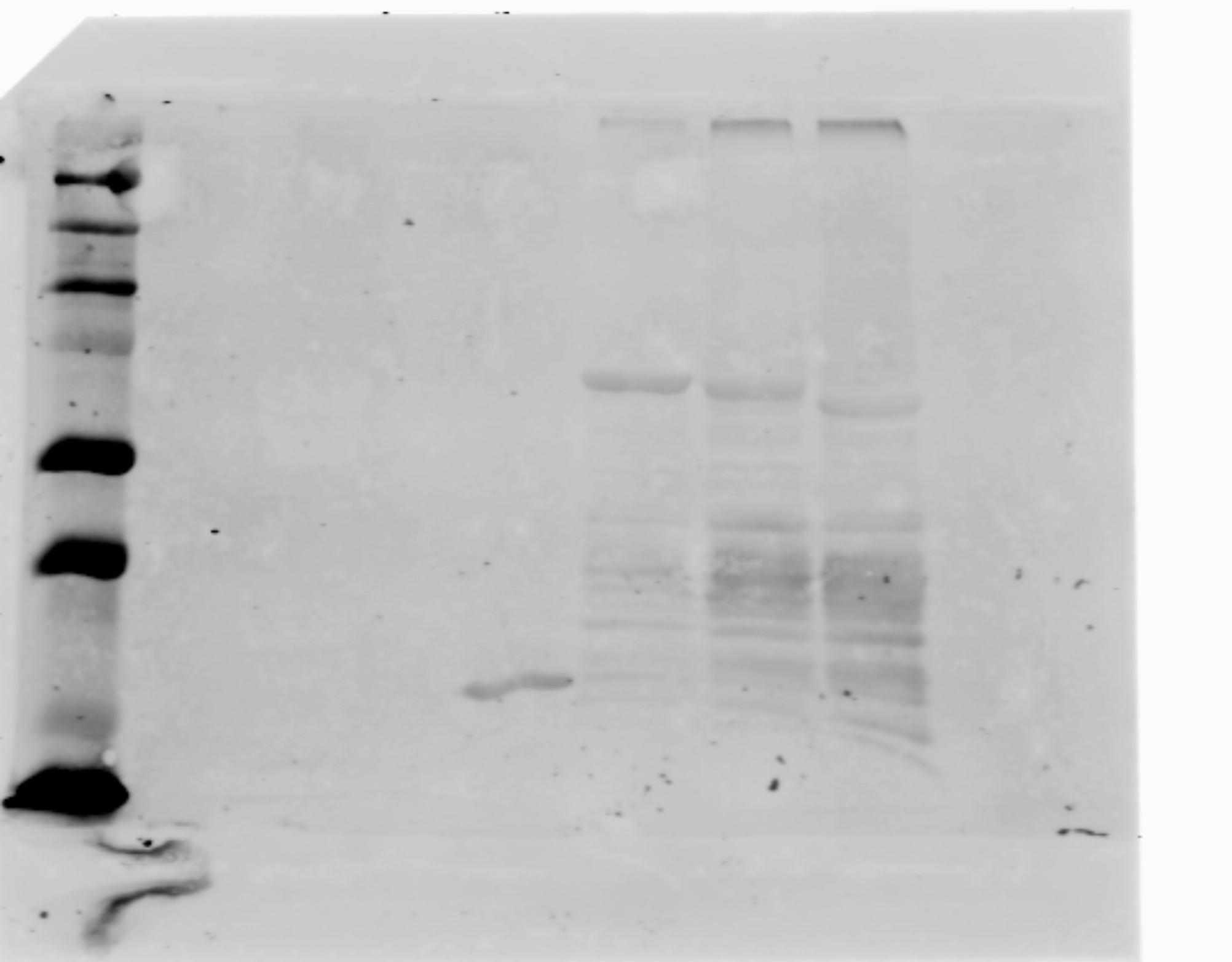

Supplement: Supplementary file 6 — Source Data for Figure 2 [file EMBJ-42-e110454-s001.zip › Fig2/FigH/debcl-GST.tif]

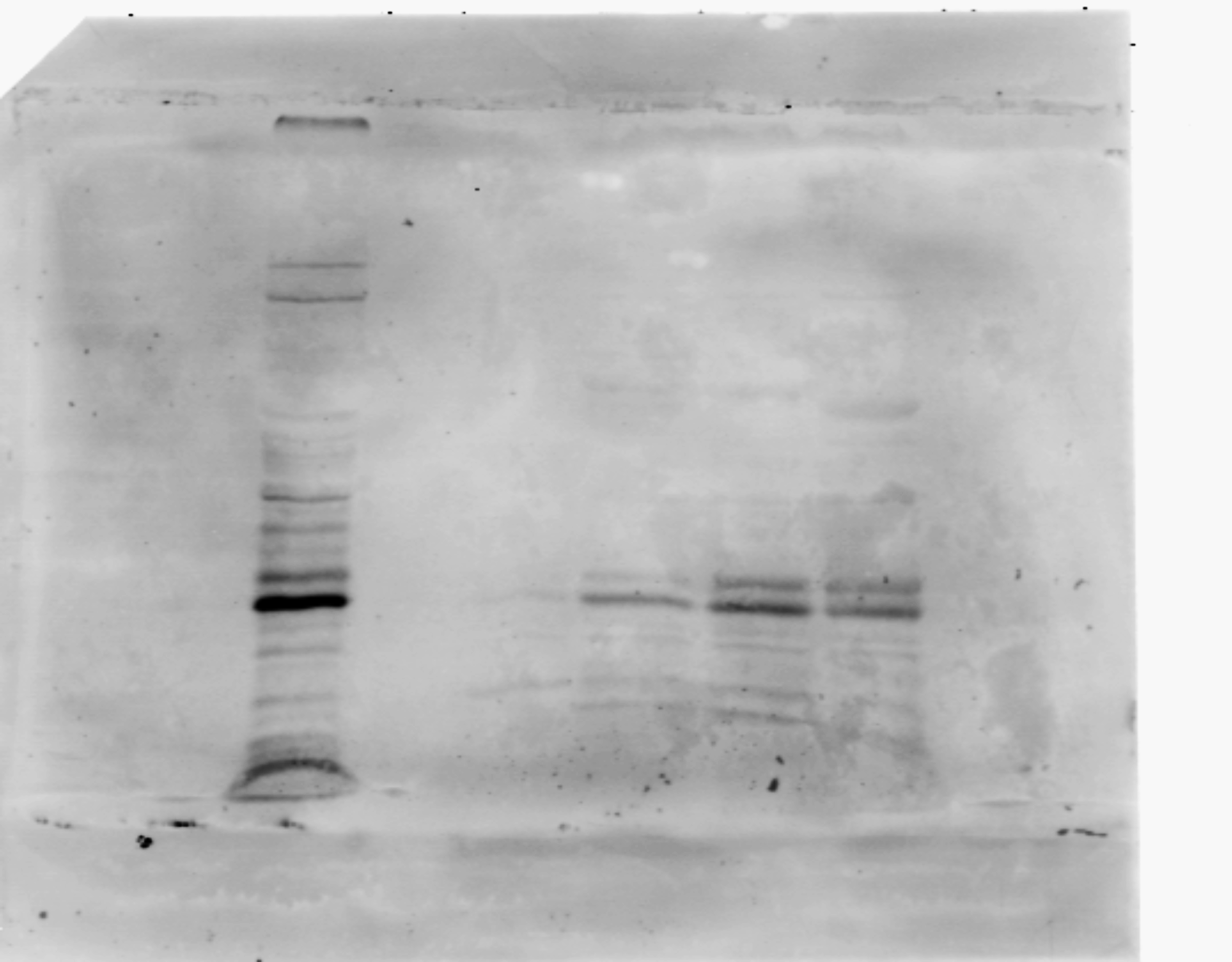

Supplement: Supplementary file 6 — Source Data for Figure 2 [file EMBJ-42-e110454-s001.zip › Fig2/FigH/debcl-flag.tif]

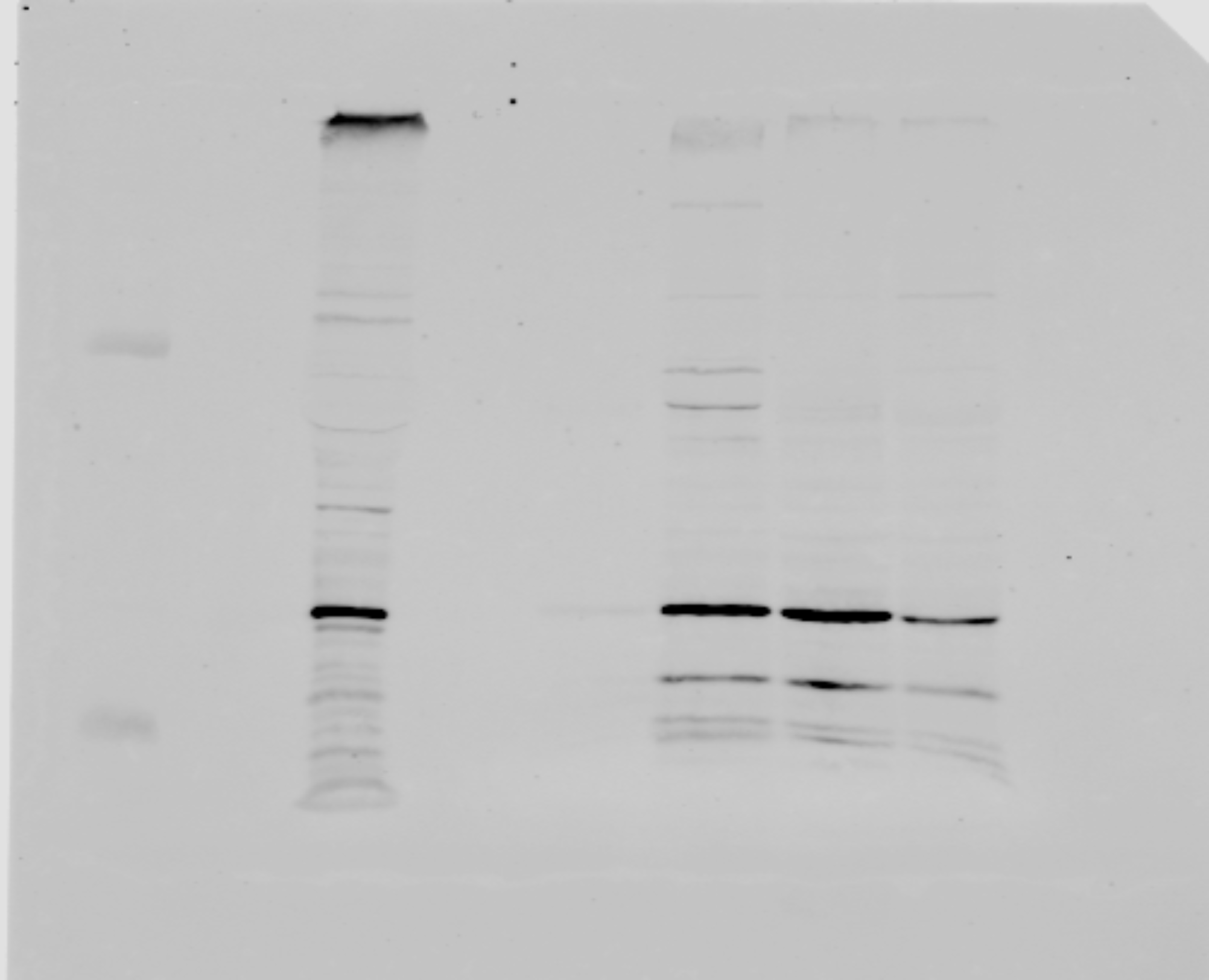

Supplement: Supplementary file 6 — Source Data for Figure 2 [file EMBJ-42-e110454-s001.zip › Fig2/FigI/buffy-flag.tif]

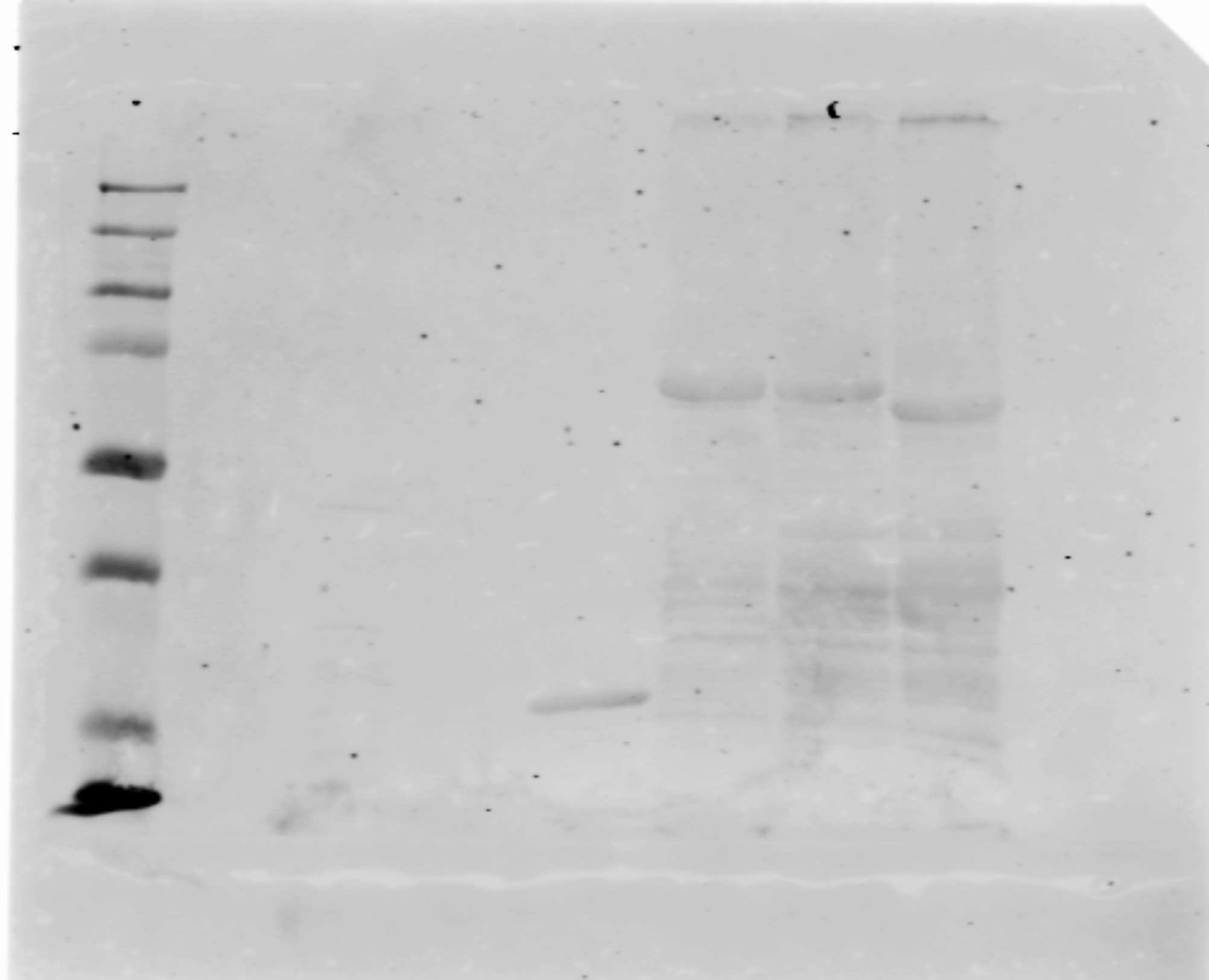

Supplement: Supplementary file 6 — Source Data for Figure 2 [file EMBJ-42-e110454-s001.zip › Fig2/FigI/buffy-GST.tif]

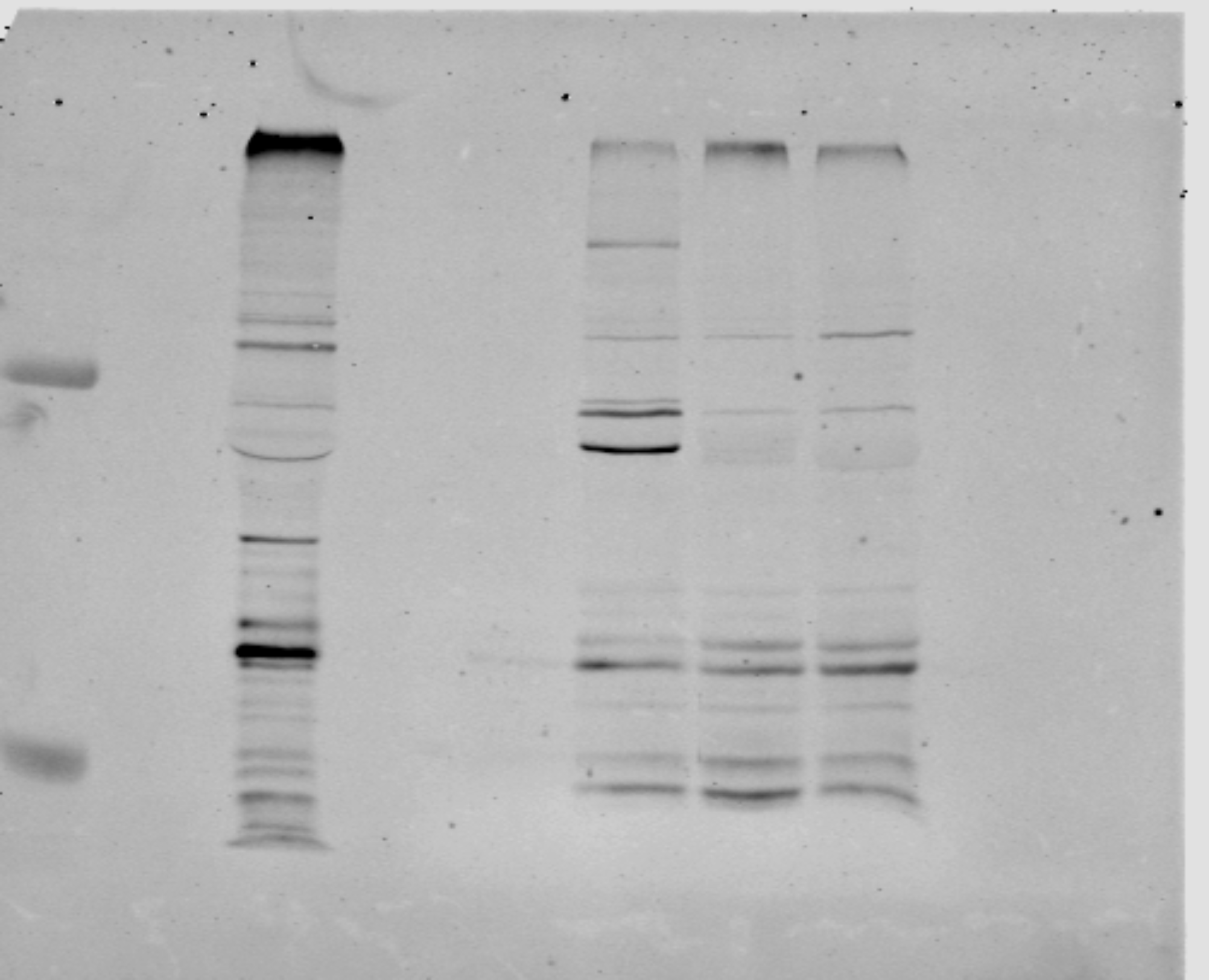

Supplement: Supplementary file 6 — Source Data for Figure 2 [file EMBJ-42-e110454-s001.zip › Fig2/FigJ/debcl-flag.tif]

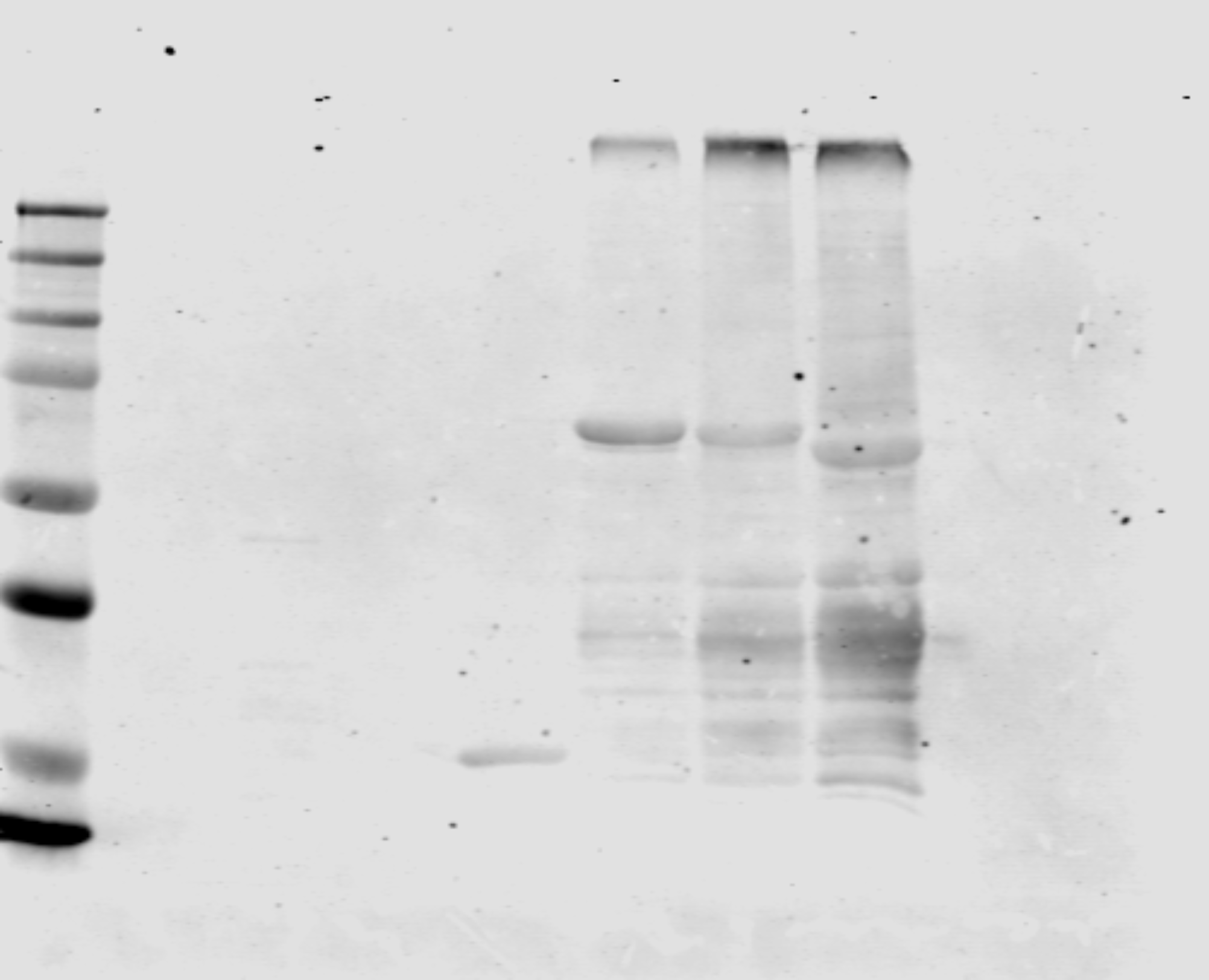

Supplement: Supplementary file 6 — Source Data for Figure 2 [file EMBJ-42-e110454-s001.zip › Fig2/FigJ/debcl-GST.tif]

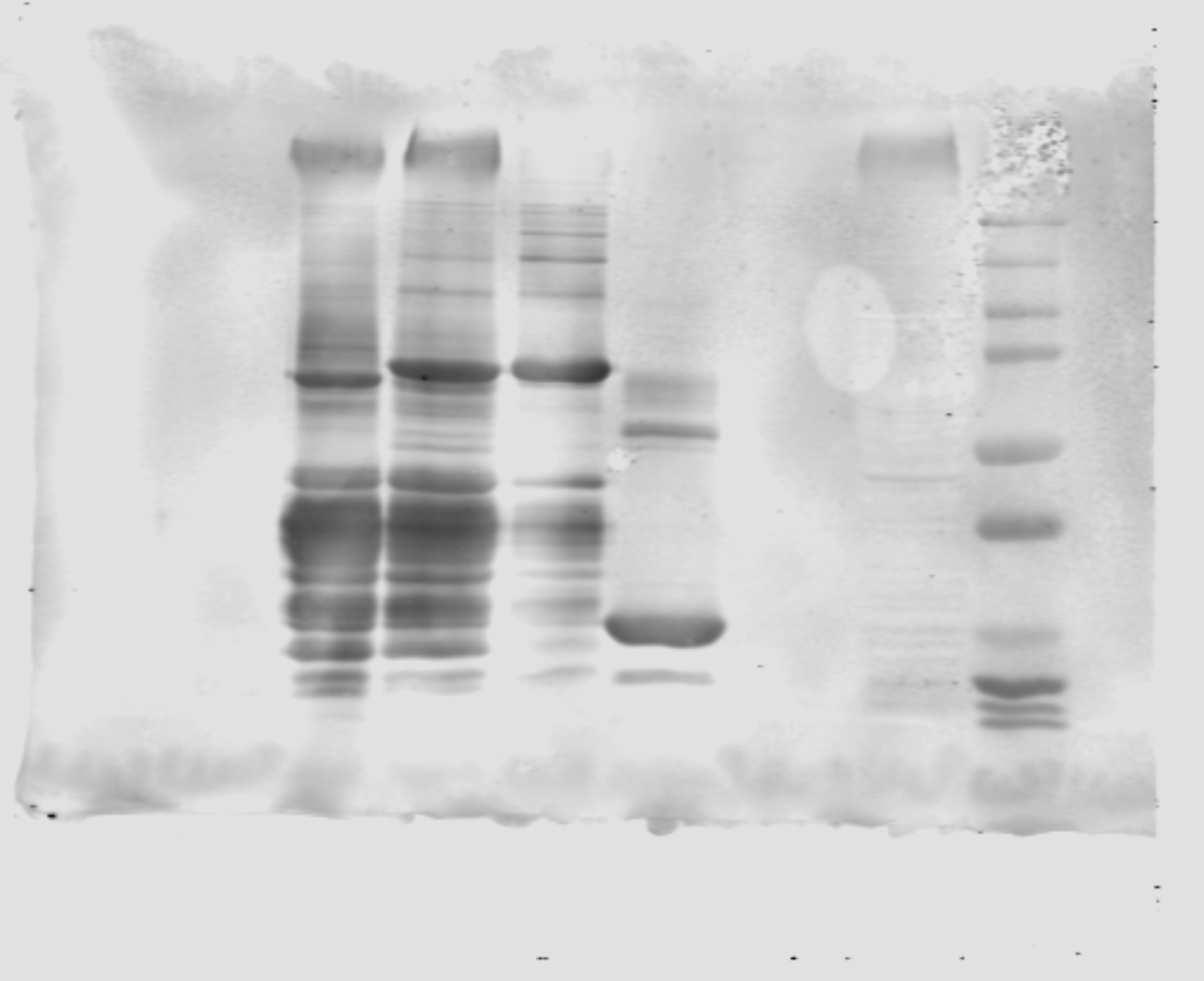

Supplement: Supplementary file 6 — Source Data for Figure 2 [file EMBJ-42-e110454-s001.zip › Fig2/FigL/2-synr.tif]

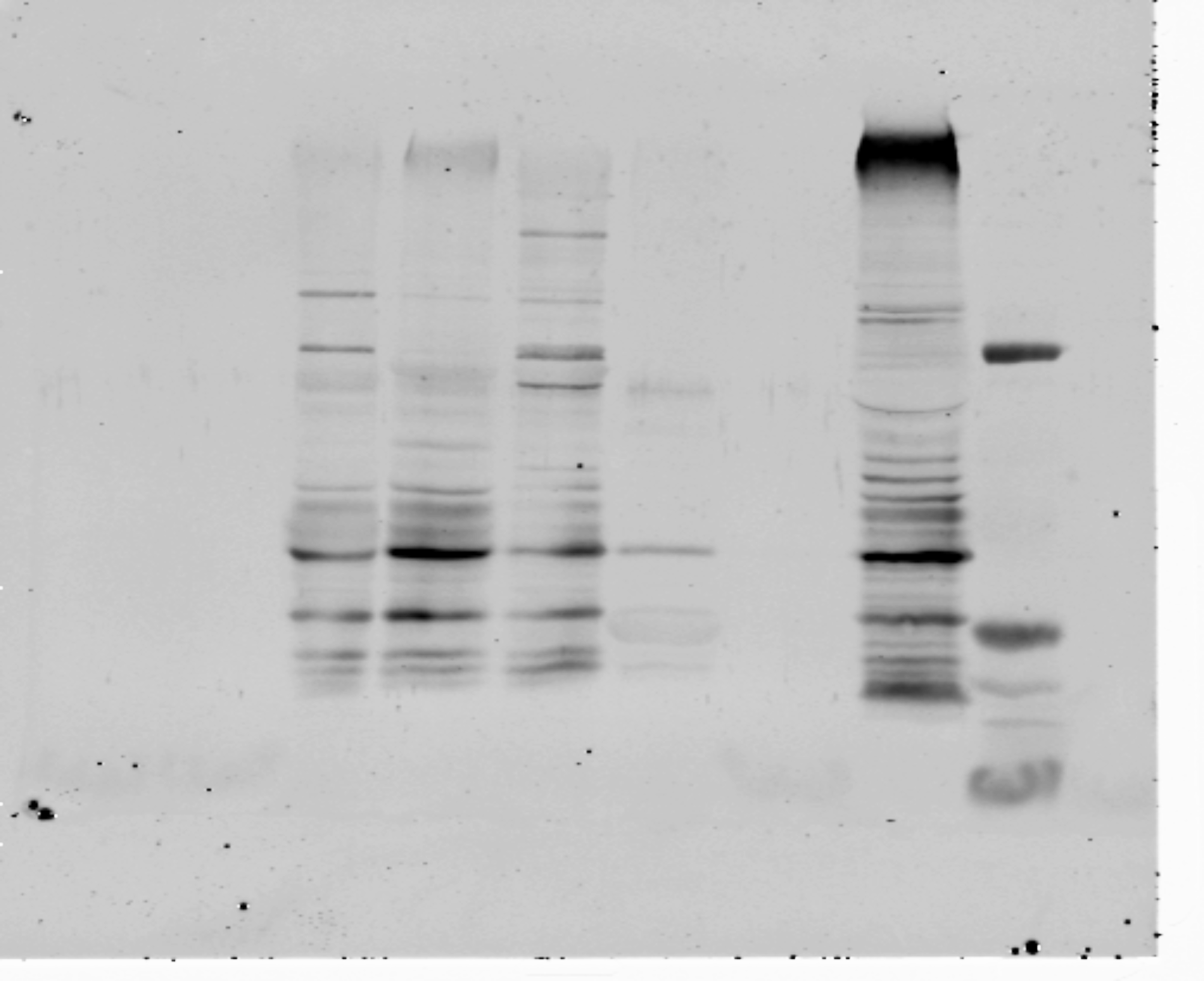

Supplement: Supplementary file 6 — Source Data for Figure 2 [file EMBJ-42-e110454-s001.zip › Fig2/FigL/2-B.tif]

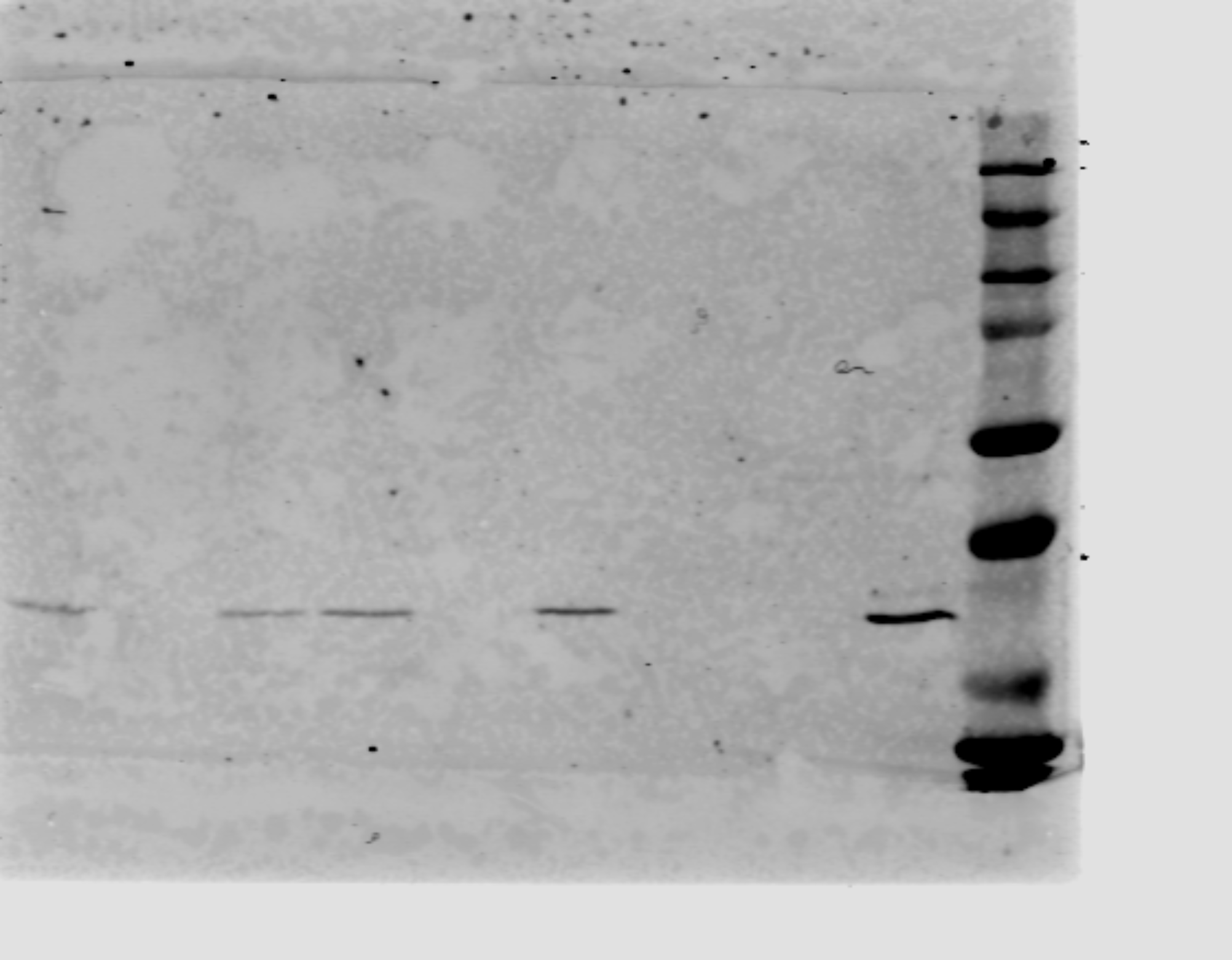

Supplement: Supplementary file 6 — Source Data for Figure 2 [file EMBJ-42-e110454-s001.zip › Fig2/FigQ/2-D.tif]

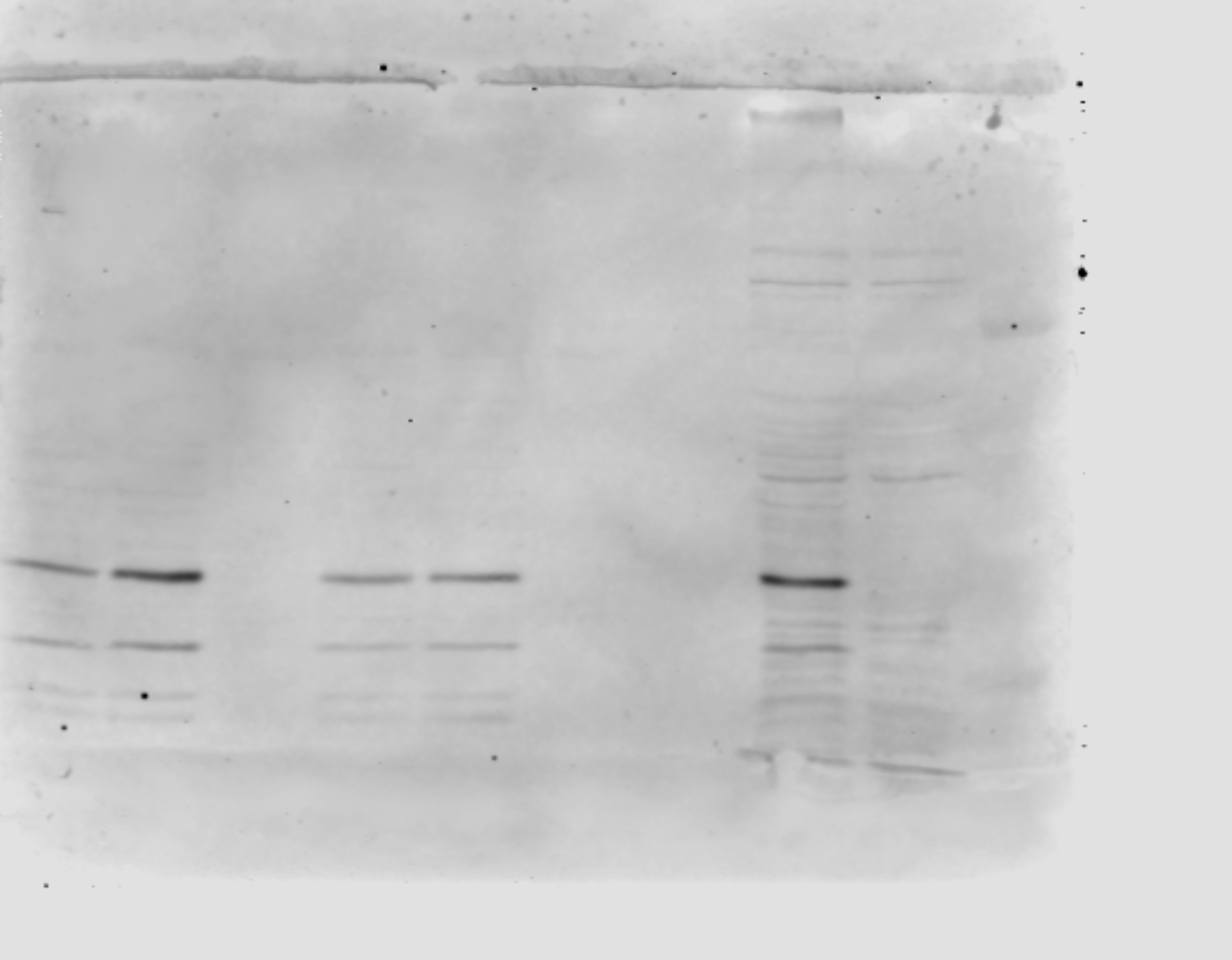

Supplement: Supplementary file 6 — Source Data for Figure 2 [file EMBJ-42-e110454-s001.zip › Fig2/FigQ/2-B.tif]

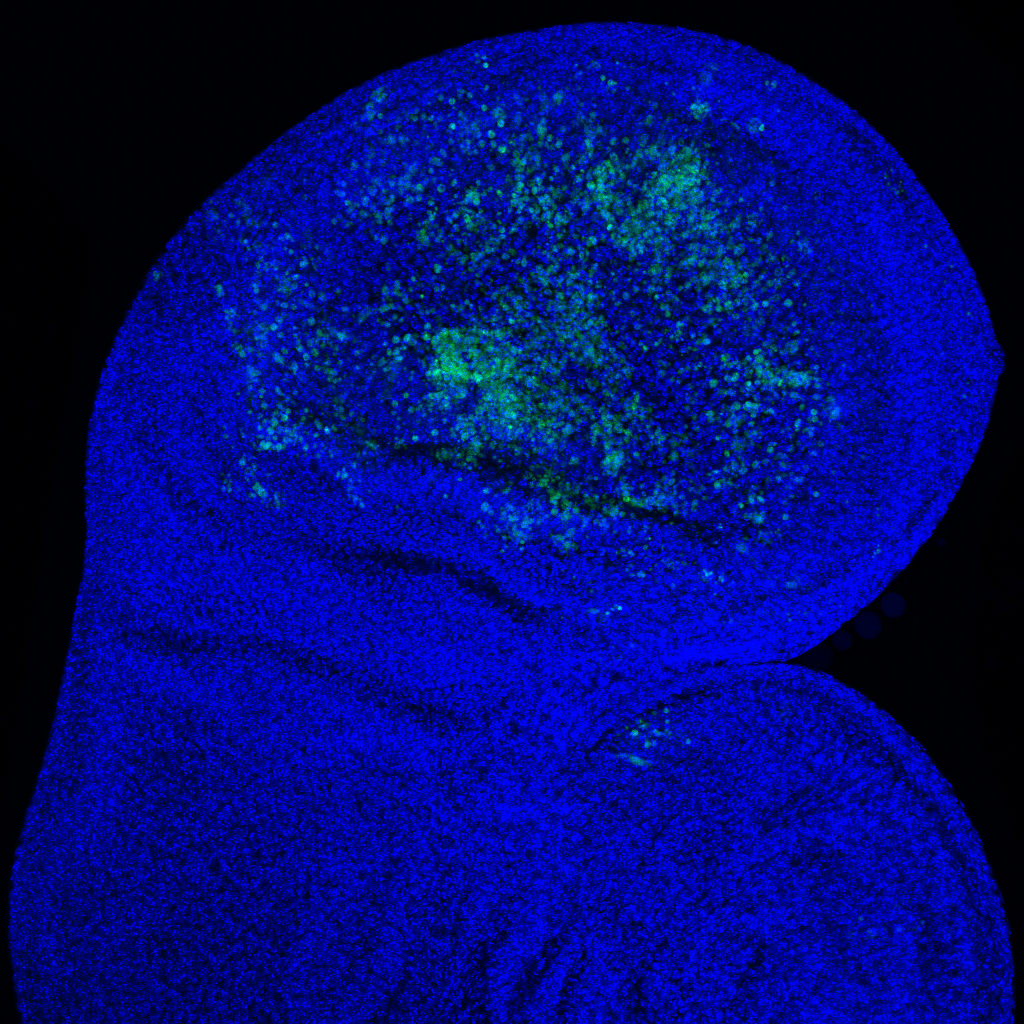

Supplement: Supplementary file 7 — Source Data for Figure 3 [file EMBJ-42-e110454-s007.zip › Fig3/FigH/Atg8a RNAi-merge.tif]

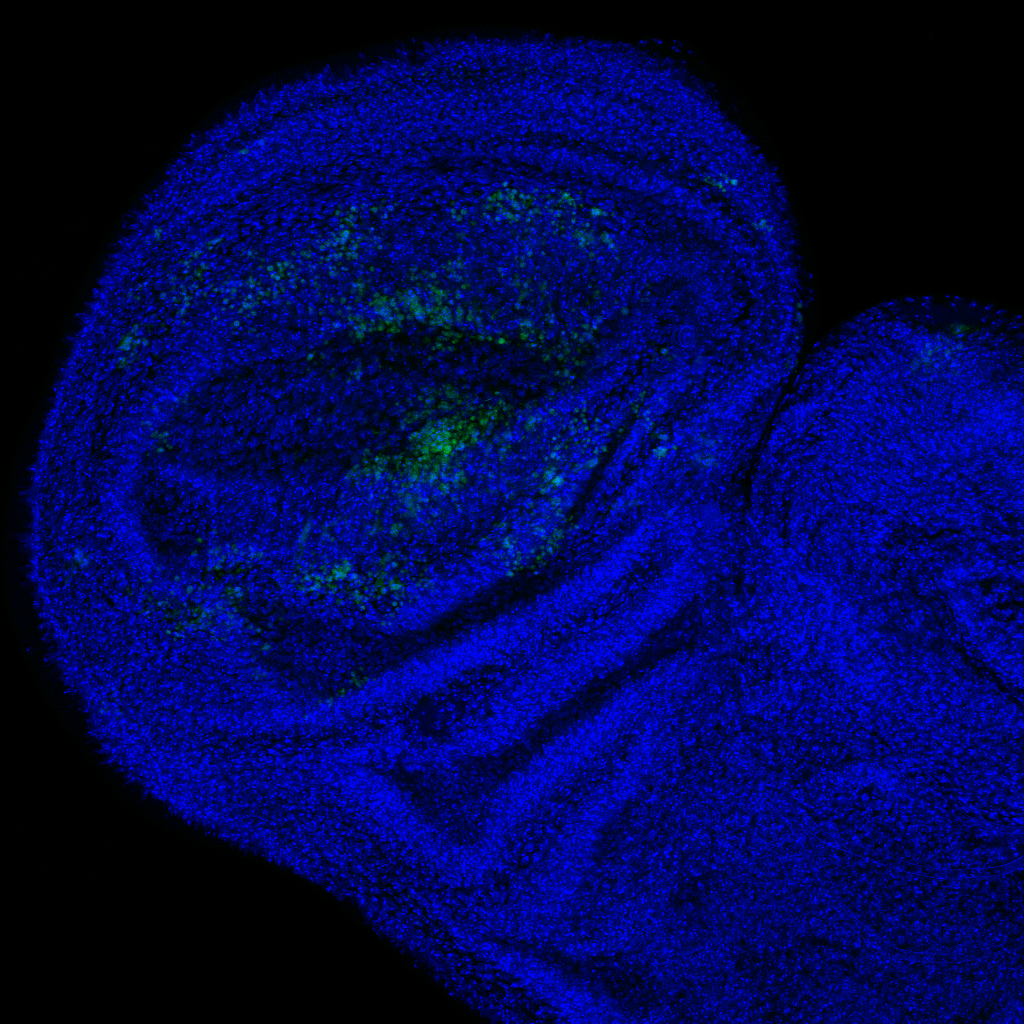

Supplement: Supplementary file 7 — Source Data for Figure 3 [file EMBJ-42-e110454-s007.zip › Fig3/FigH/Atg2 RNAi-merge.tif]

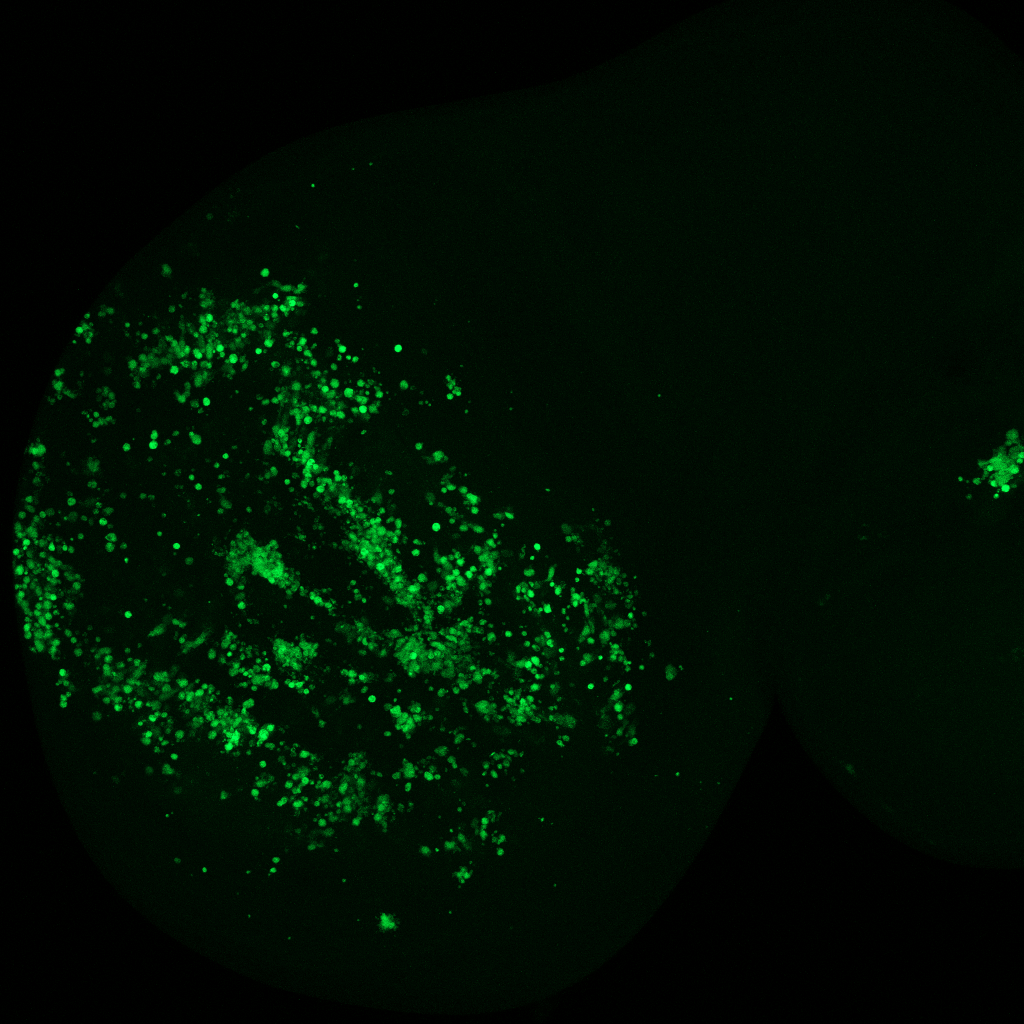

Supplement: Supplementary file 7 — Source Data for Figure 3 [file EMBJ-42-e110454-s007.zip › Fig3/FigH/Dcp-1 RNAi-GC3Ai.tif]

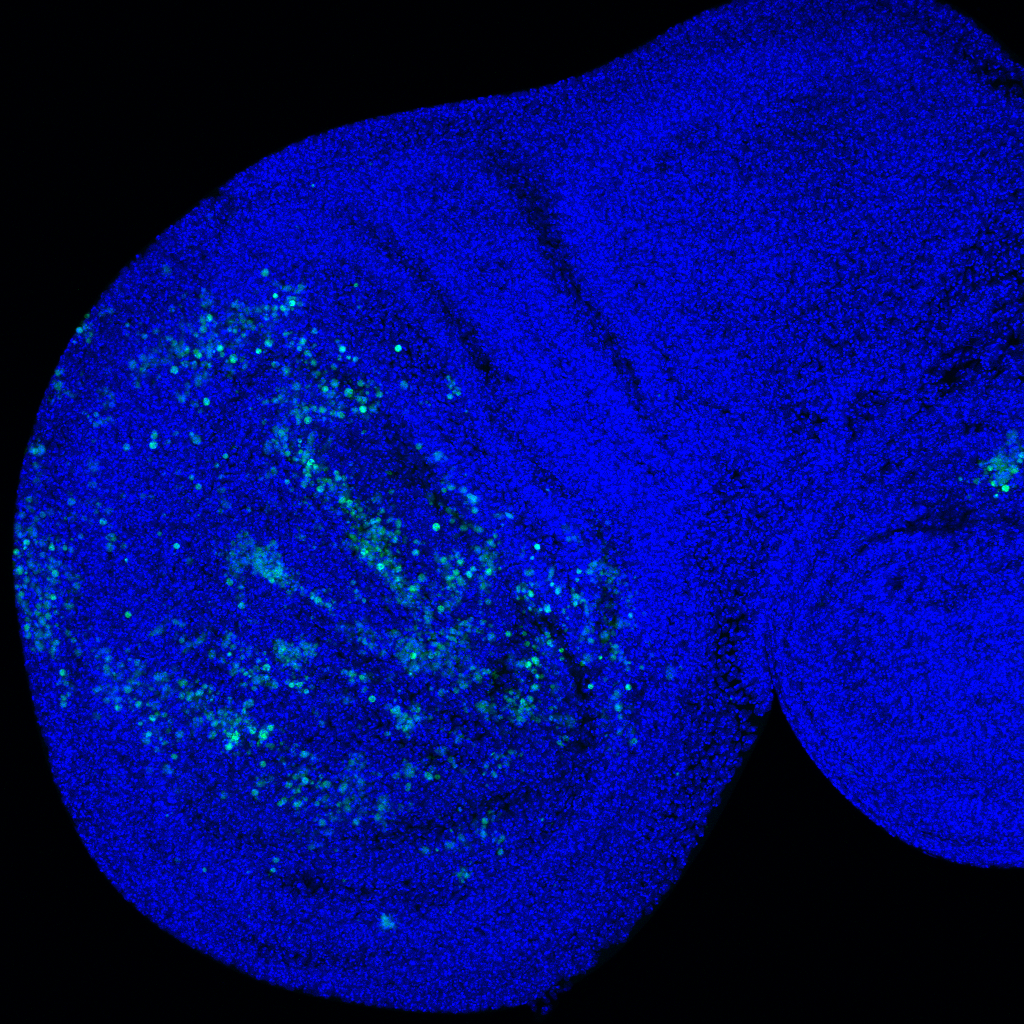

Supplement: Supplementary file 7 — Source Data for Figure 3 [file EMBJ-42-e110454-s007.zip › Fig3/FigH/Dcp-1 RNAi-merge.tif]

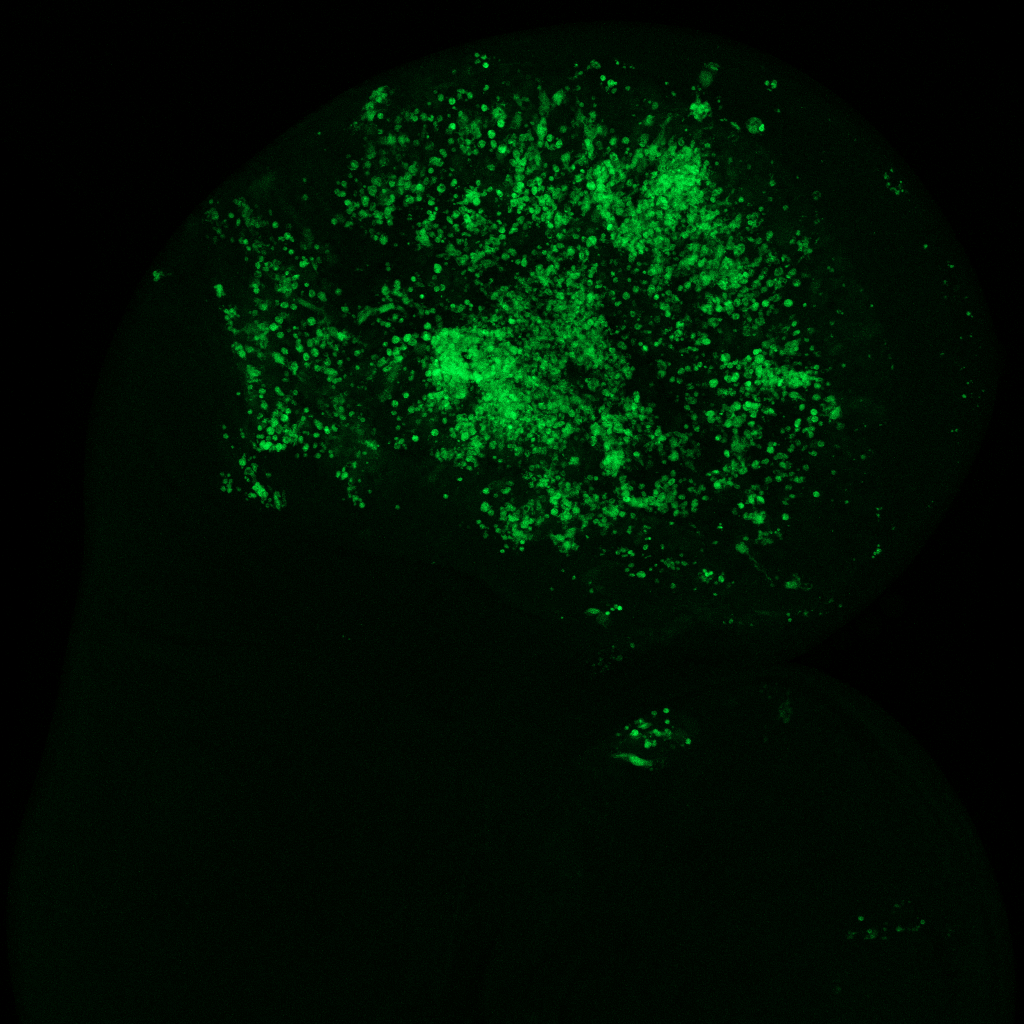

Supplement: Supplementary file 7 — Source Data for Figure 3 [file EMBJ-42-e110454-s007.zip › Fig3/FigH/Atg8a RNAi-GC3Ai.tif]

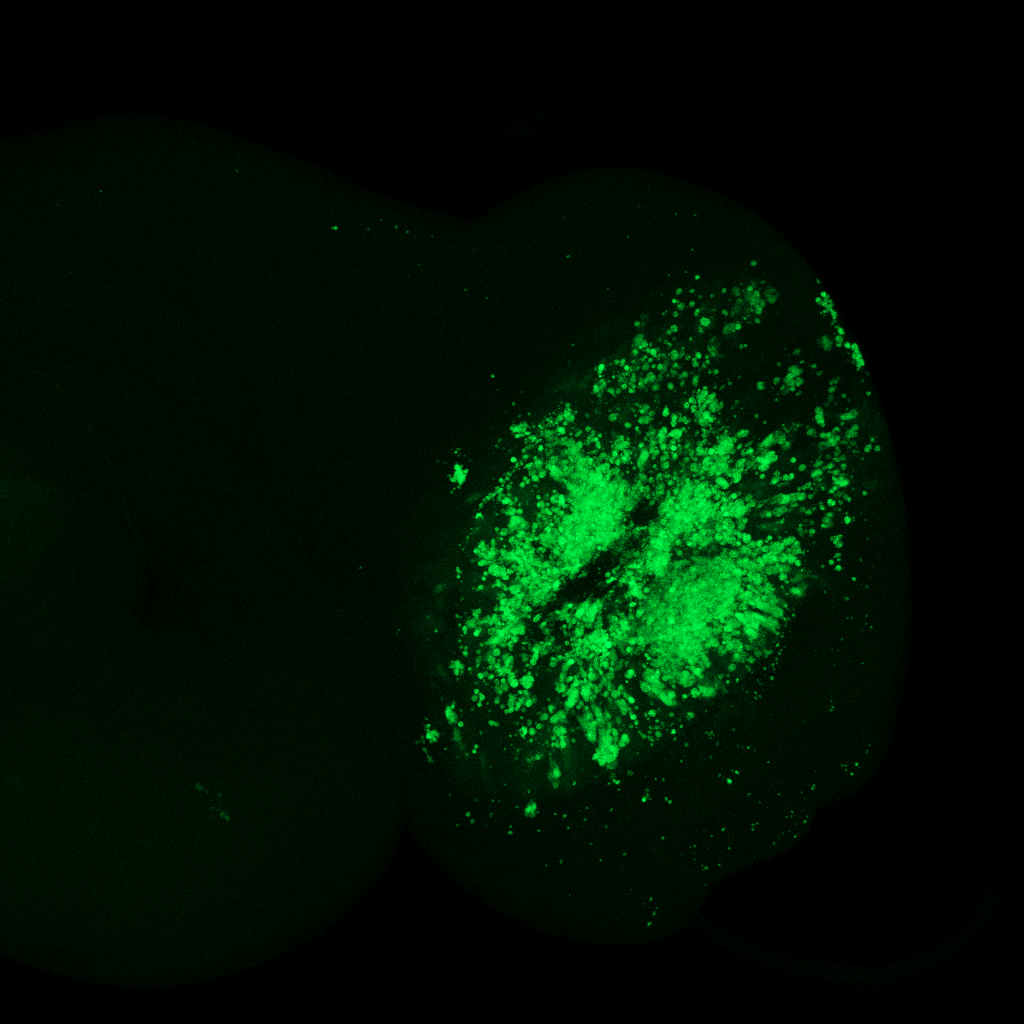

Supplement: Supplementary file 7 — Source Data for Figure 3 [file EMBJ-42-e110454-s007.zip › Fig3/FigH/w--GC3Ai.tif]

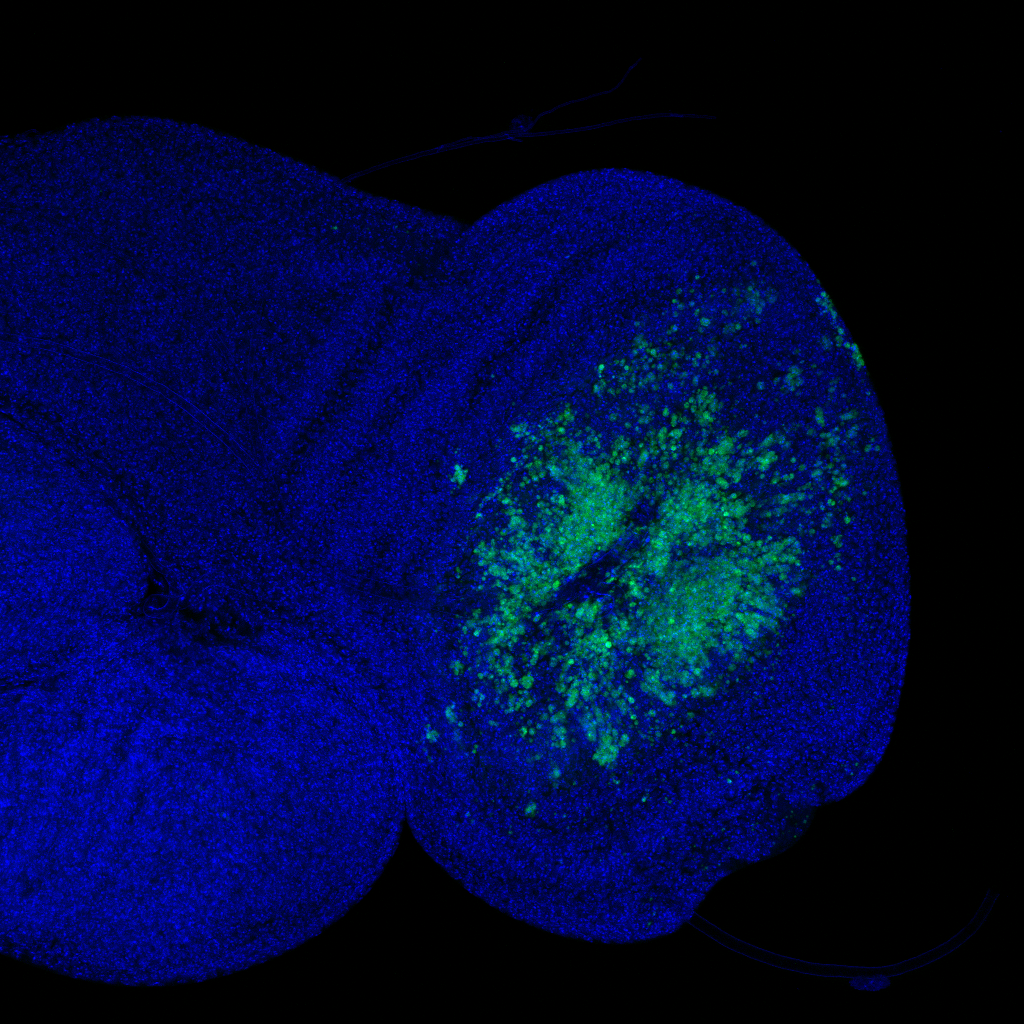

Supplement: Supplementary file 7 — Source Data for Figure 3 [file EMBJ-42-e110454-s007.zip › Fig3/FigH/w--merge.tif]

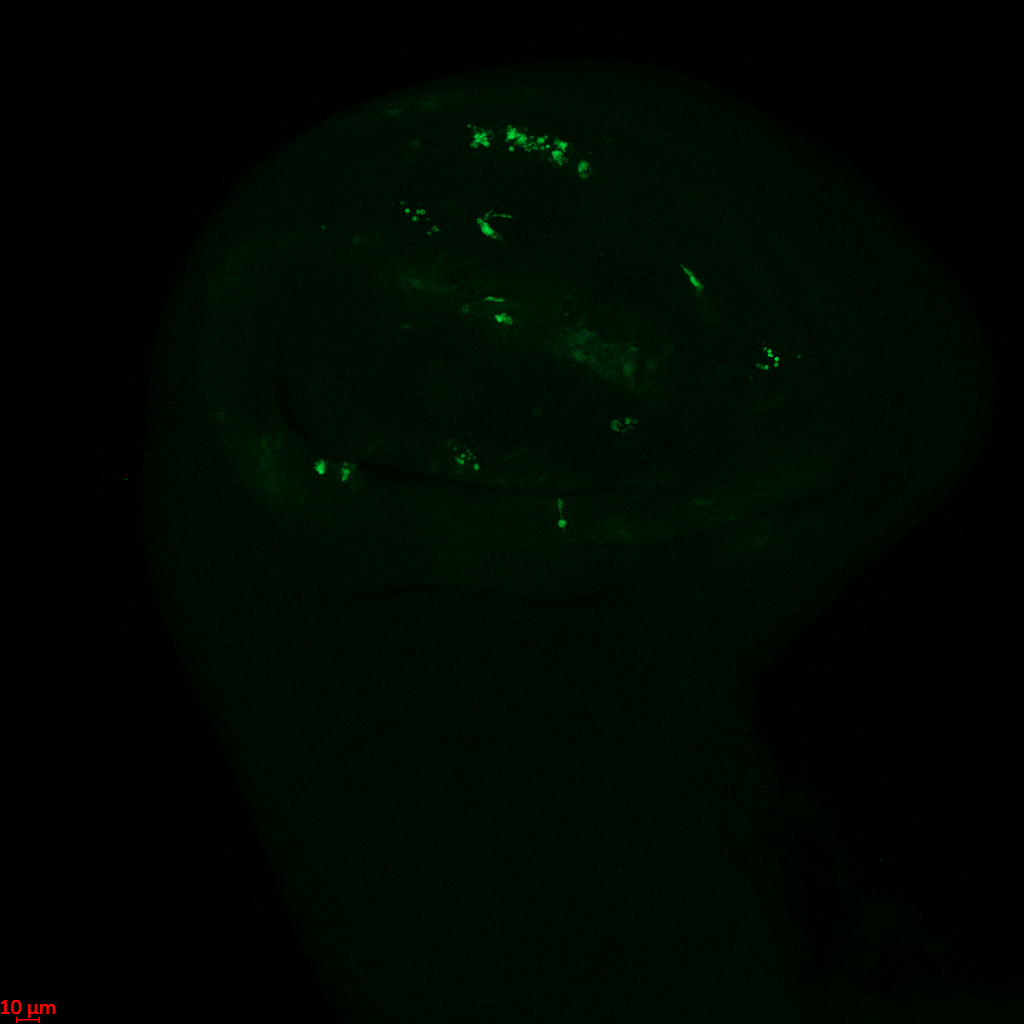

Supplement: Supplementary file 7 — Source Data for Figure 3 [file EMBJ-42-e110454-s007.zip › Fig3/FigH/synr RNAi-GC3Ai.tif]

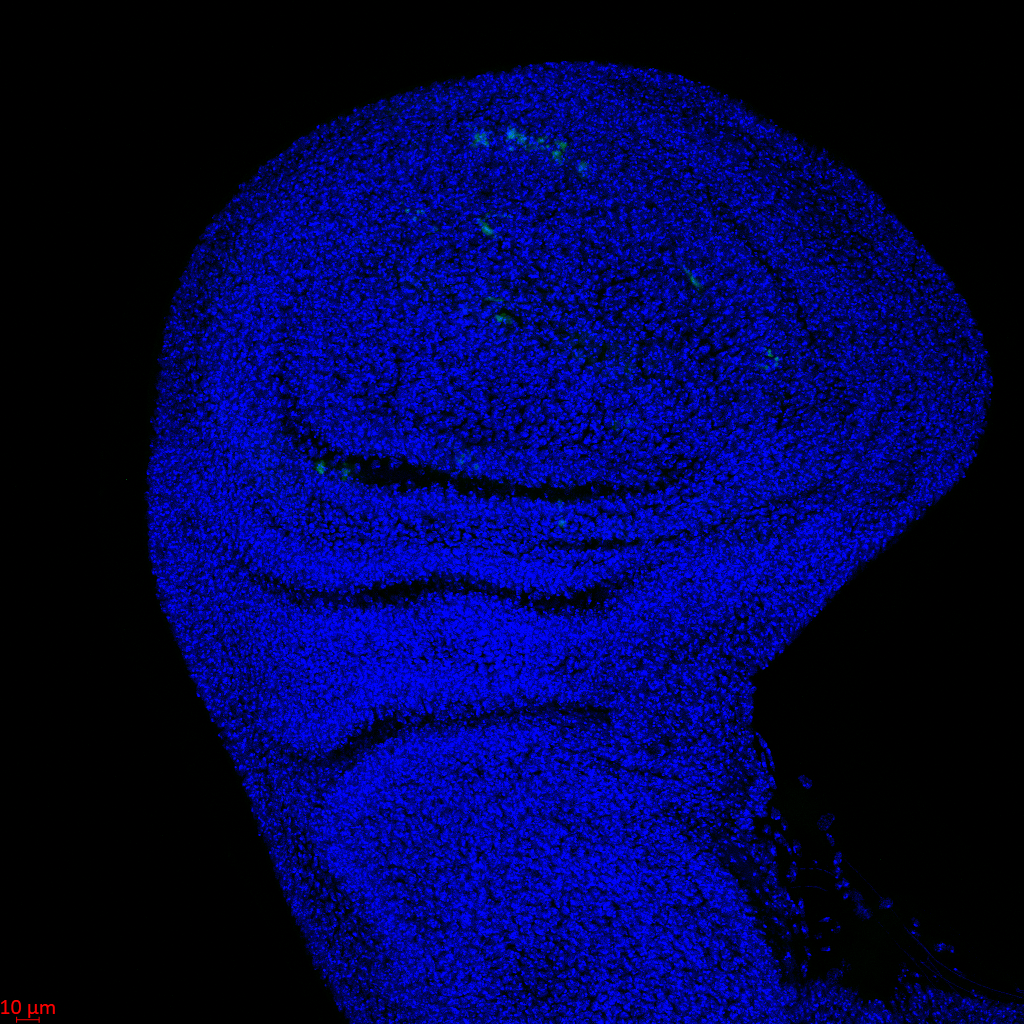

Supplement: Supplementary file 7 — Source Data for Figure 3 [file EMBJ-42-e110454-s007.zip › Fig3/FigH/synr RNAi-merge.tif]

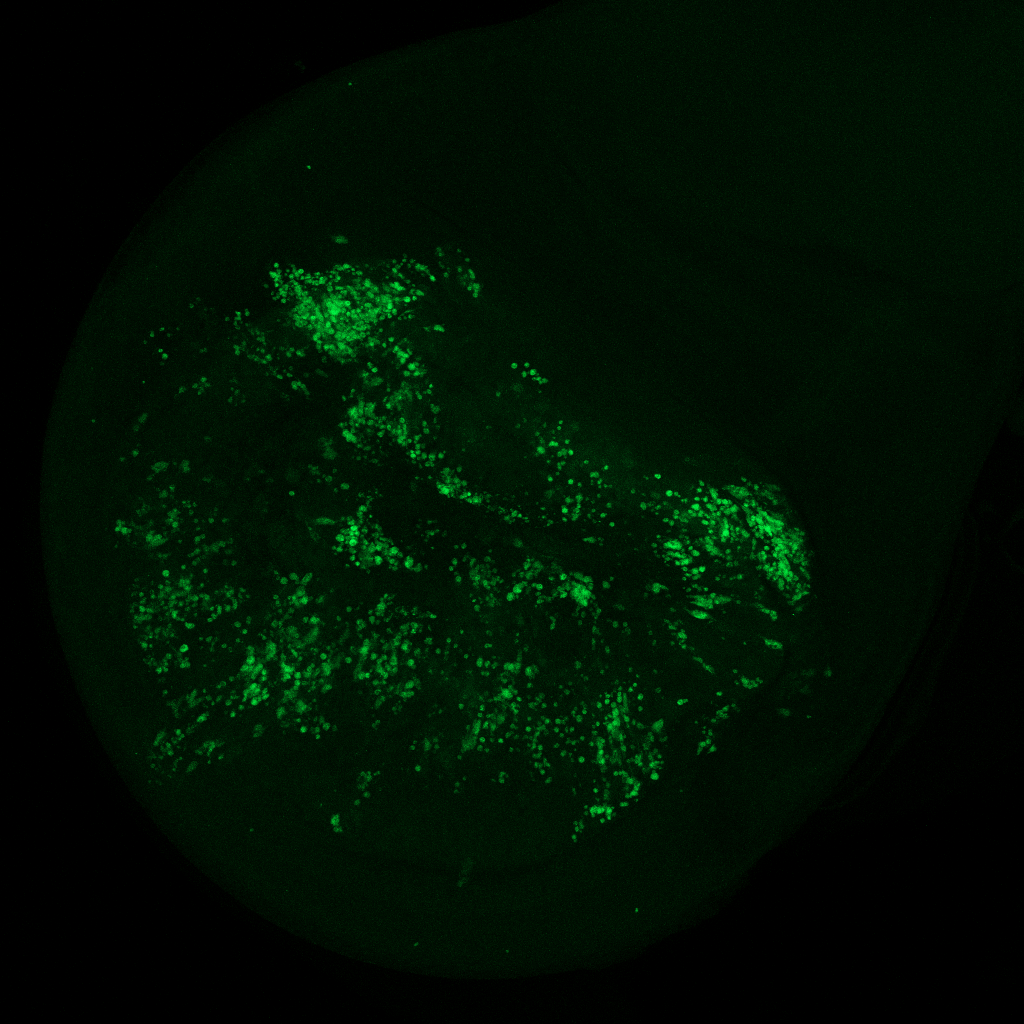

Supplement: Supplementary file 7 — Source Data for Figure 3 [file EMBJ-42-e110454-s007.zip › Fig3/FigH/Debcl RNAi-GC3Ai.tif]

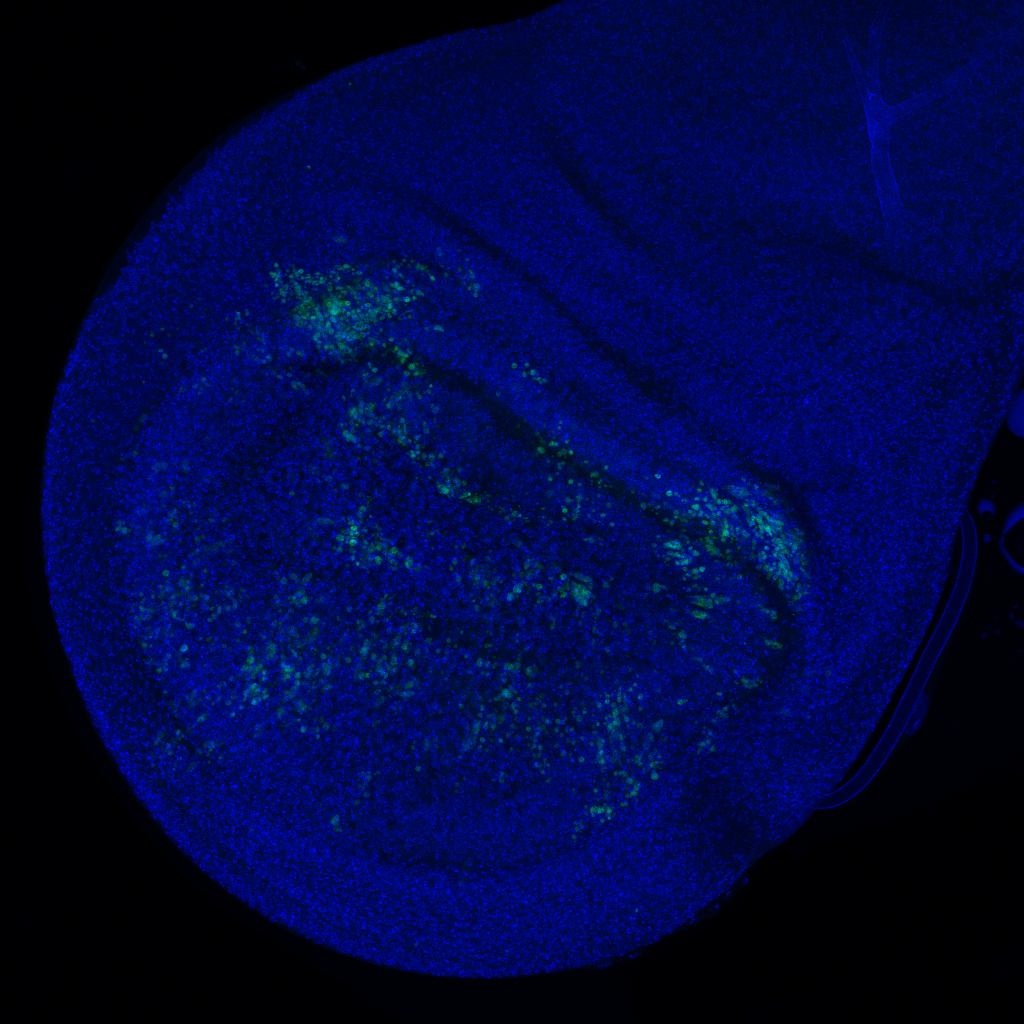

Supplement: Supplementary file 7 — Source Data for Figure 3 [file EMBJ-42-e110454-s007.zip › Fig3/FigH/Debcl RNAi-merge.tif]

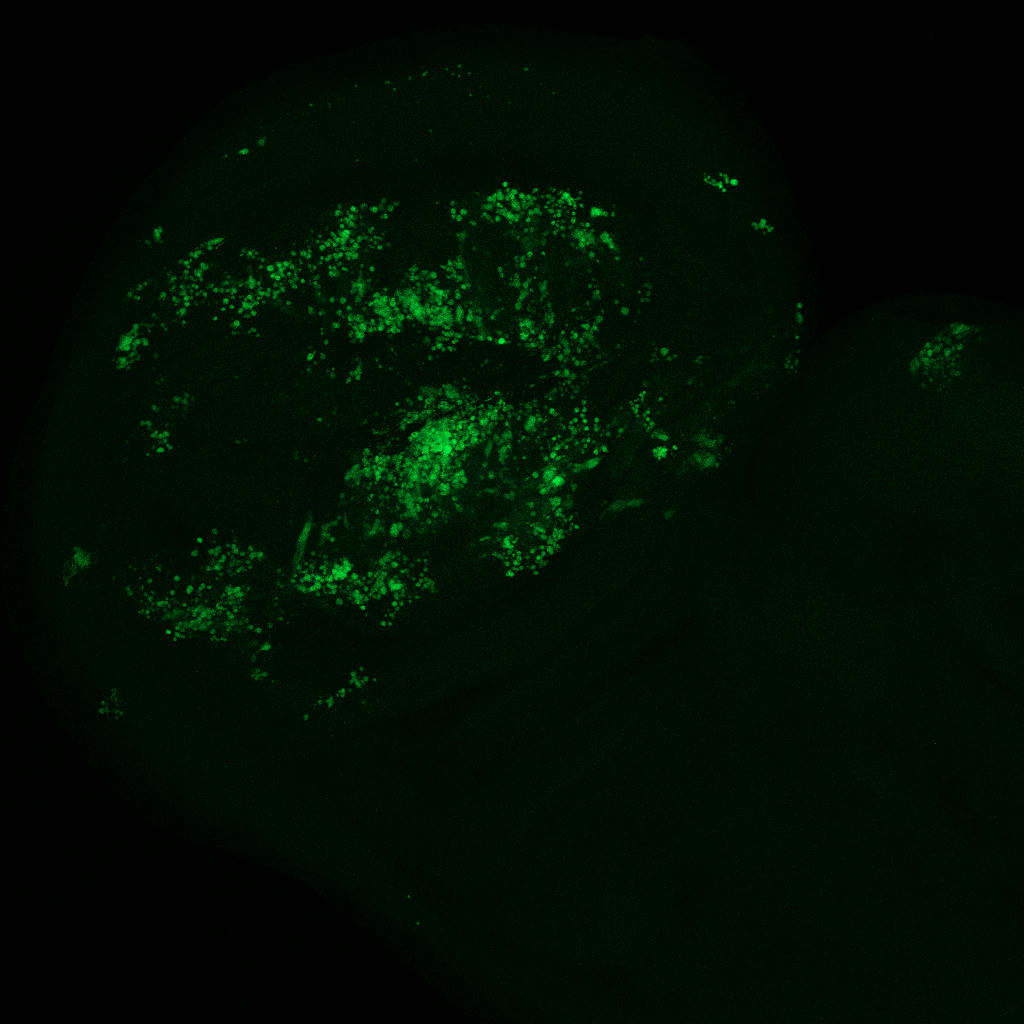

Supplement: Supplementary file 7 — Source Data for Figure 3 [file EMBJ-42-e110454-s007.zip › Fig3/FigH/Atg2 RNAi-GC3Ai.tif]

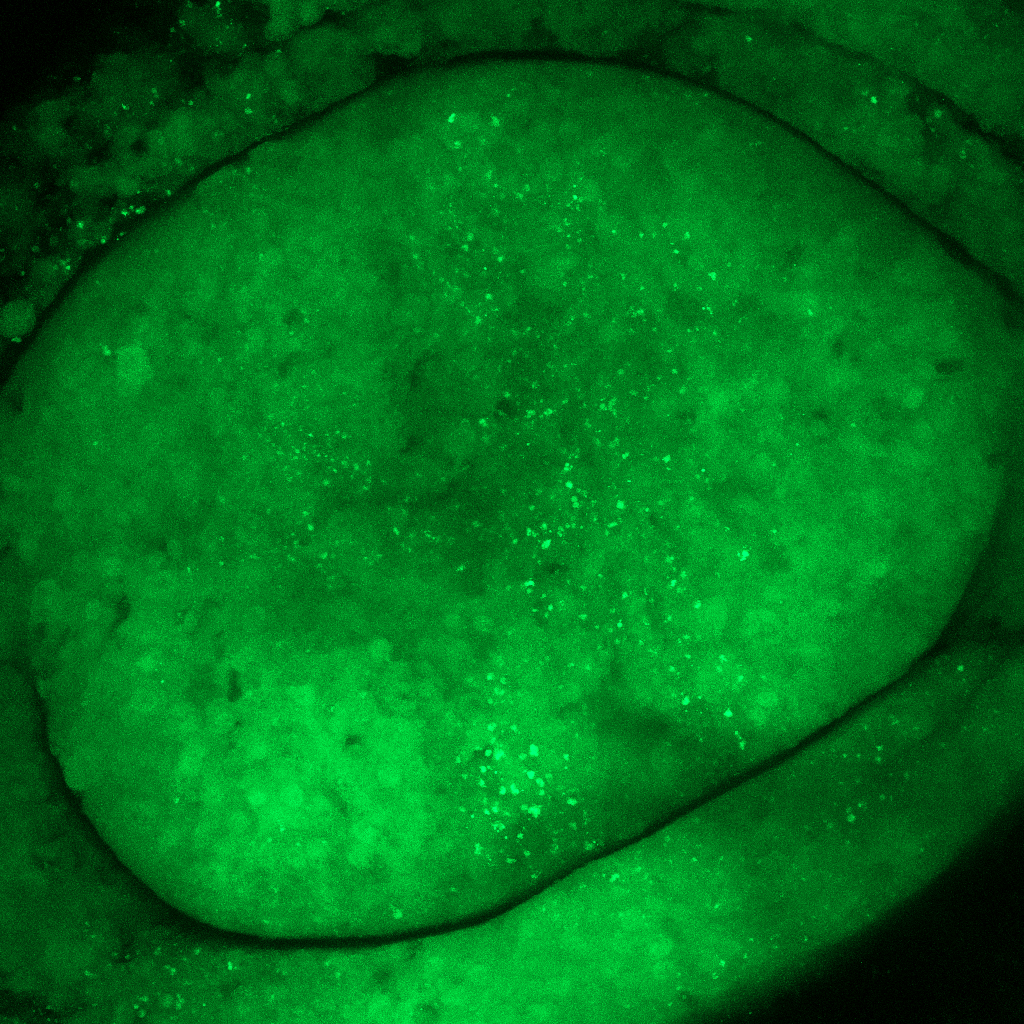

Supplement: Supplementary file 7 — Source Data for Figure 3 [file EMBJ-42-e110454-s007.zip › Fig3/FigJ/Dcp-1 RNAi-GFP.tif]

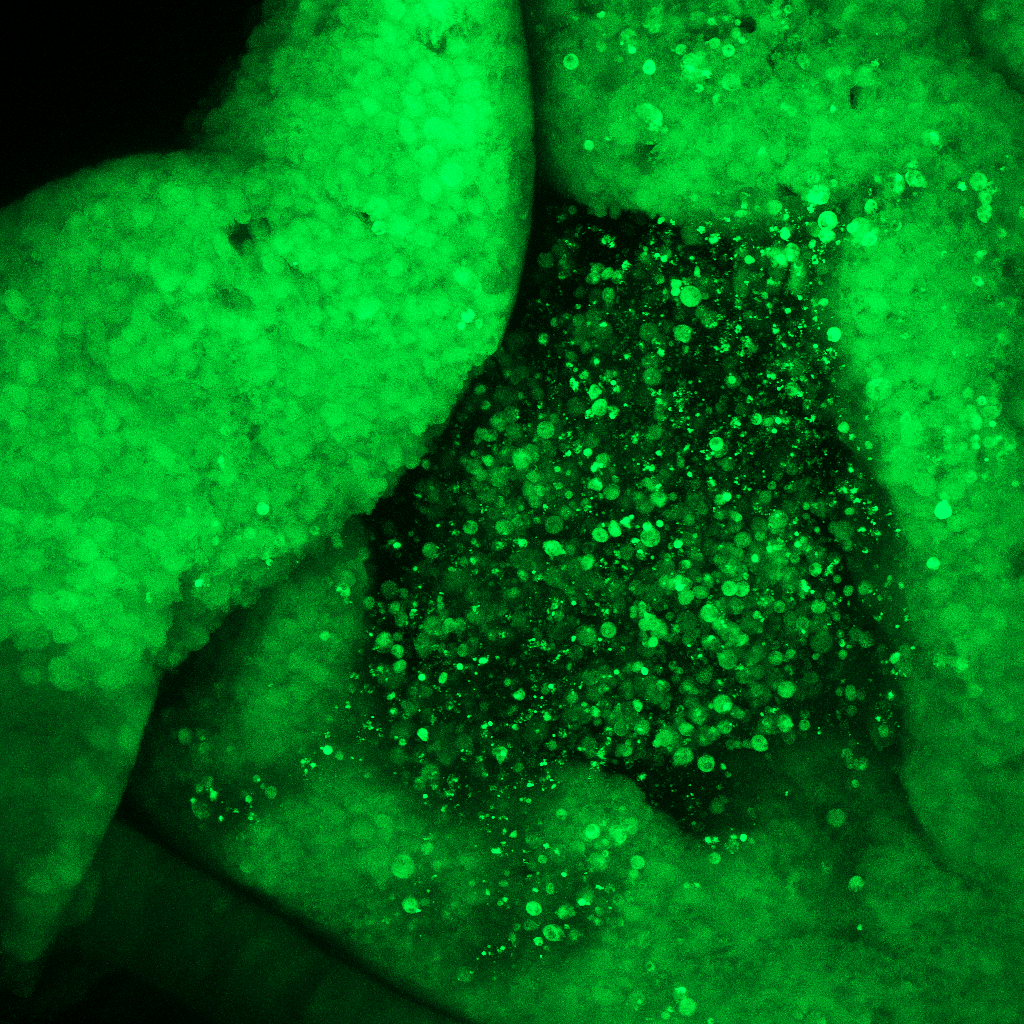

Supplement: Supplementary file 7 — Source Data for Figure 3 [file EMBJ-42-e110454-s007.zip › Fig3/FigJ/w-GFP.tif]

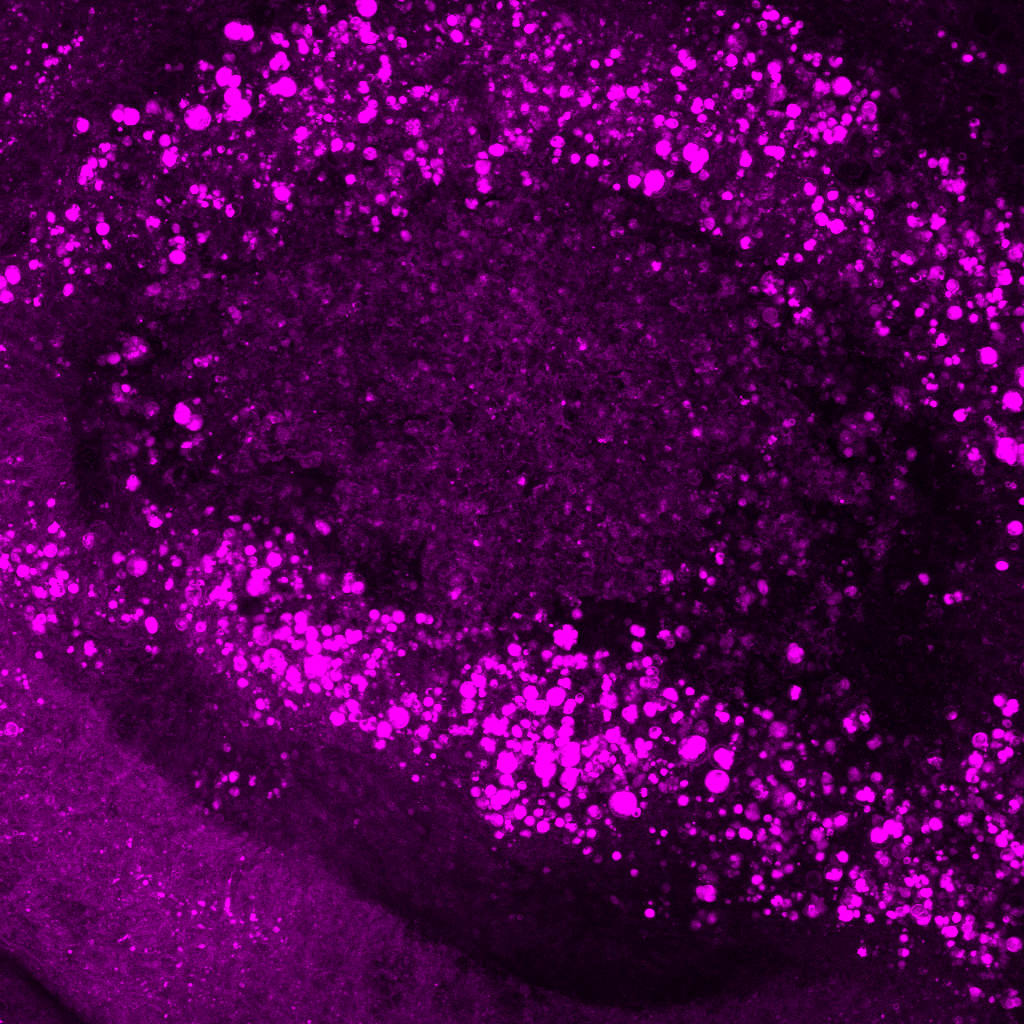

Supplement: Supplementary file 7 — Source Data for Figure 3 [file EMBJ-42-e110454-s007.zip › Fig3/FigJ/luciferase RNAi-Lysotracker.tif]

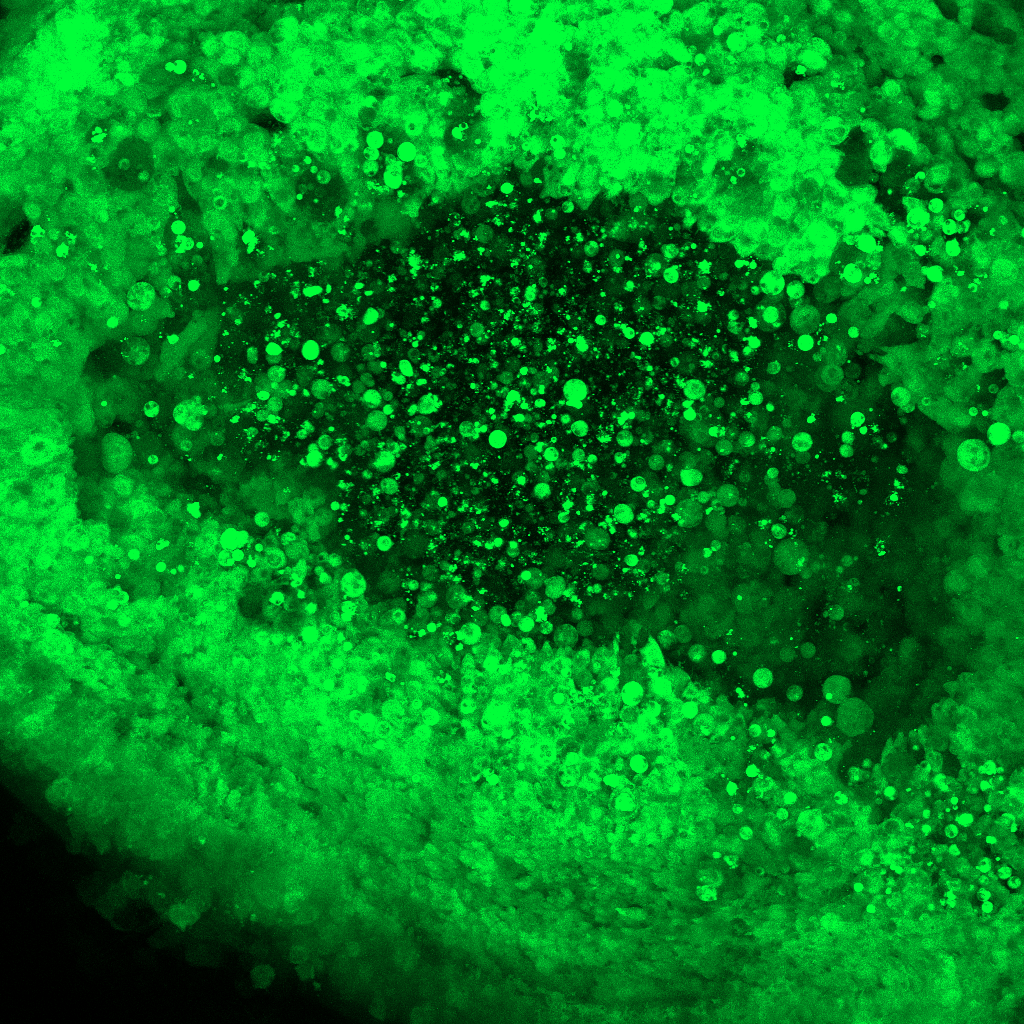

Supplement: Supplementary file 7 — Source Data for Figure 3 [file EMBJ-42-e110454-s007.zip › Fig3/FigJ/luciferase RNAi-GFP.tif]

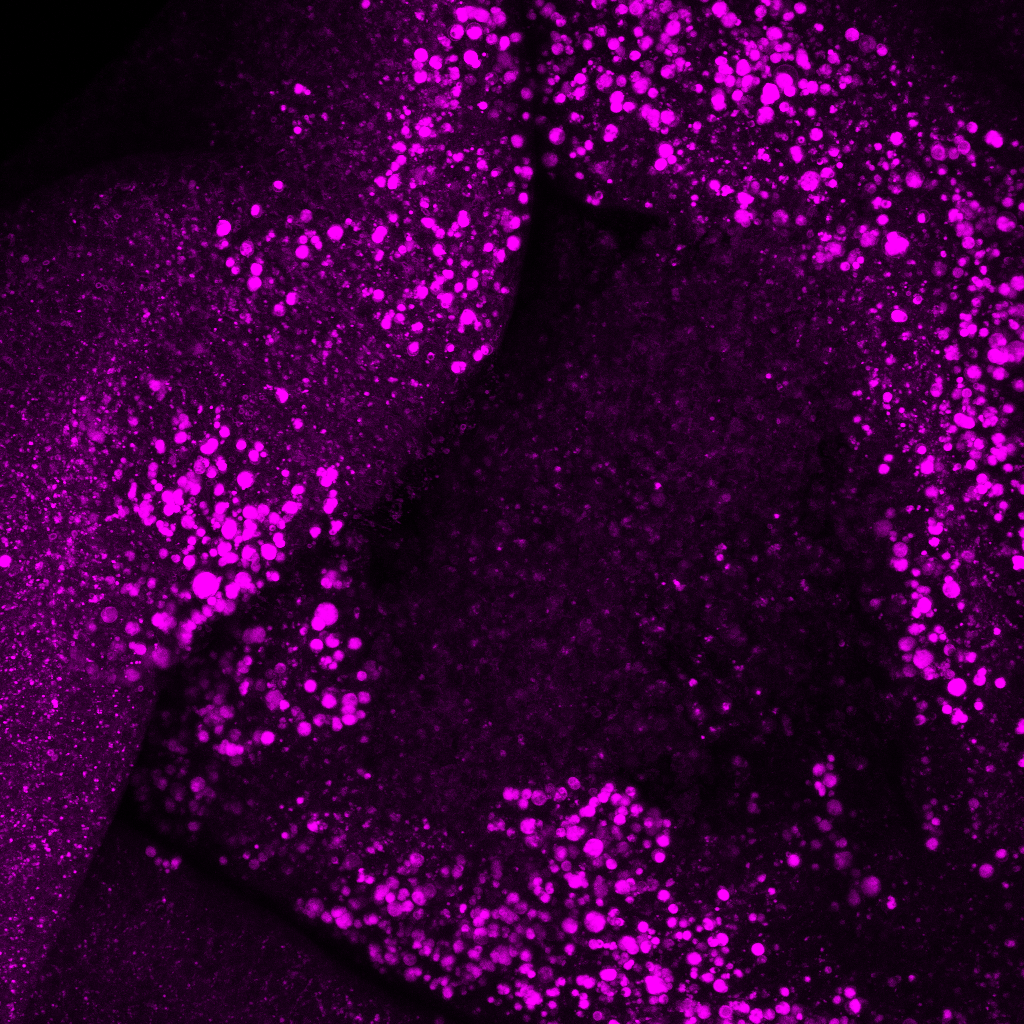

Supplement: Supplementary file 7 — Source Data for Figure 3 [file EMBJ-42-e110454-s007.zip › Fig3/FigJ/w-Lysotracker.tif]

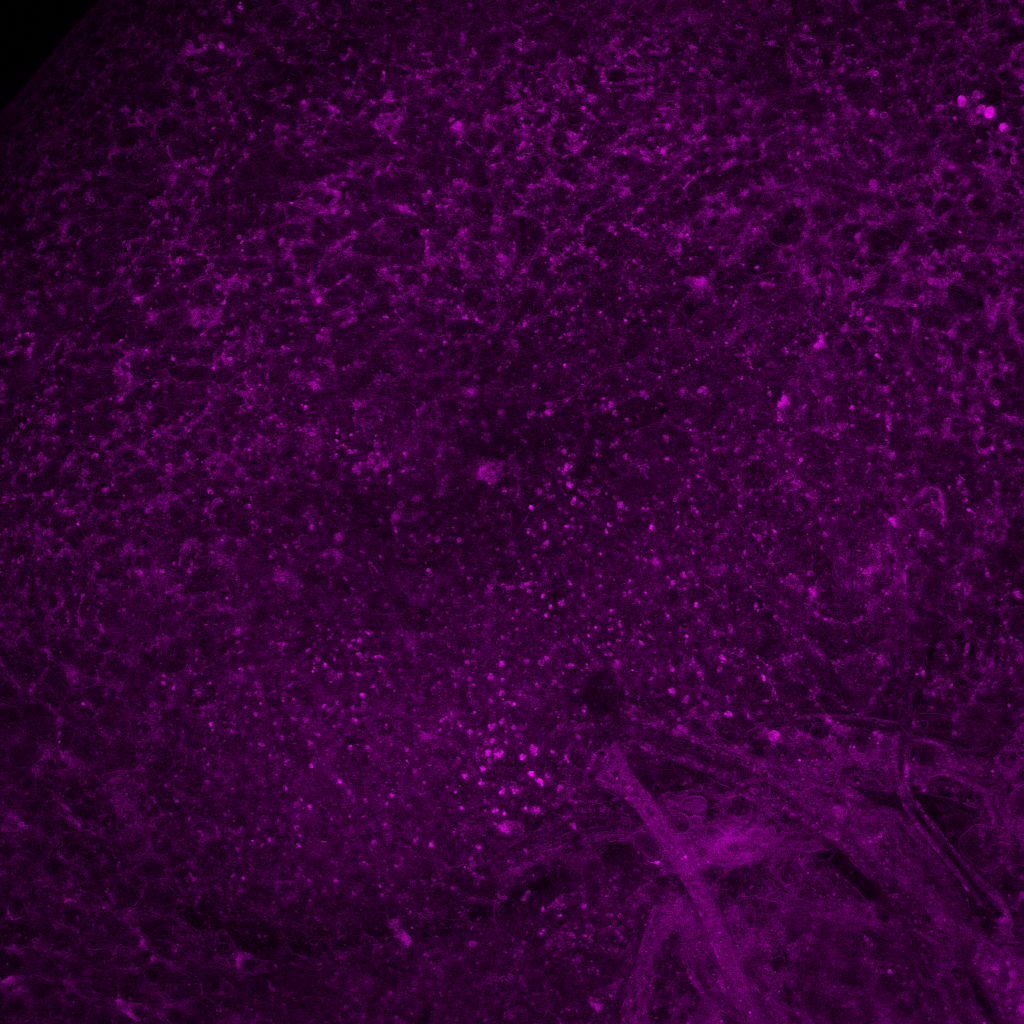

Supplement: Supplementary file 7 — Source Data for Figure 3 [file EMBJ-42-e110454-s007.zip › Fig3/FigJ/Dcp-1 RNAi-Lysotracker.tif]

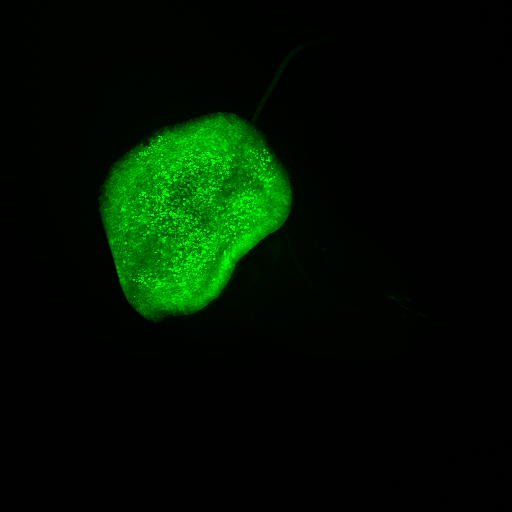

Supplement: Supplementary file 7 — Source Data for Figure 3 [file EMBJ-42-e110454-s007.zip › Fig3/FigA/synr-GFP.tif]

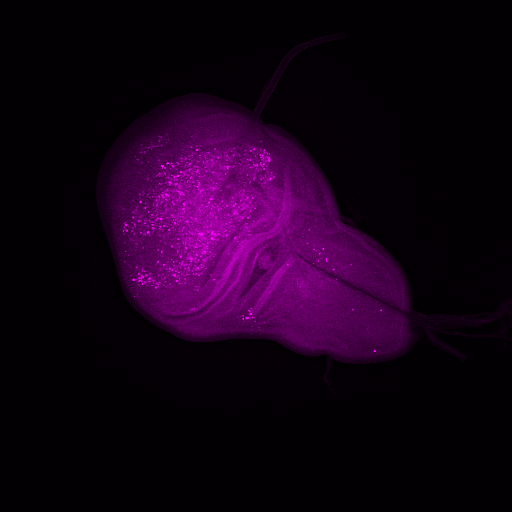

Supplement: Supplementary file 7 — Source Data for Figure 3 [file EMBJ-42-e110454-s007.zip › Fig3/FigA/synr-lysotracker.tif]

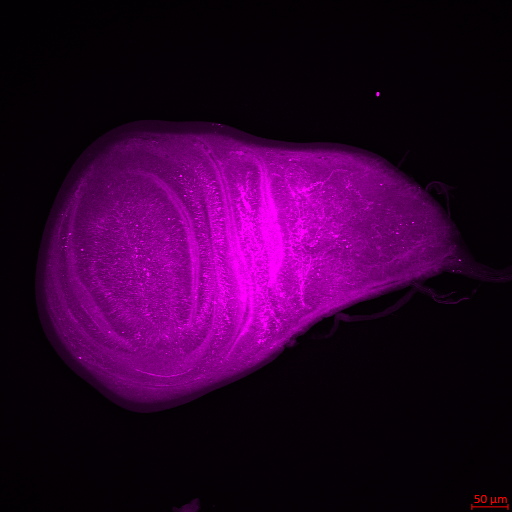

Supplement: Supplementary file 7 — Source Data for Figure 3 [file EMBJ-42-e110454-s007.zip › Fig3/FigA/+-lysotracker.tif]

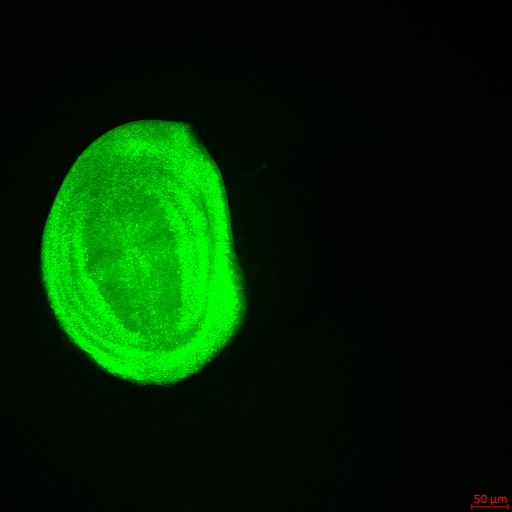

Supplement: Supplementary file 7 — Source Data for Figure 3 [file EMBJ-42-e110454-s007.zip › Fig3/FigA/+-GFP.tif]

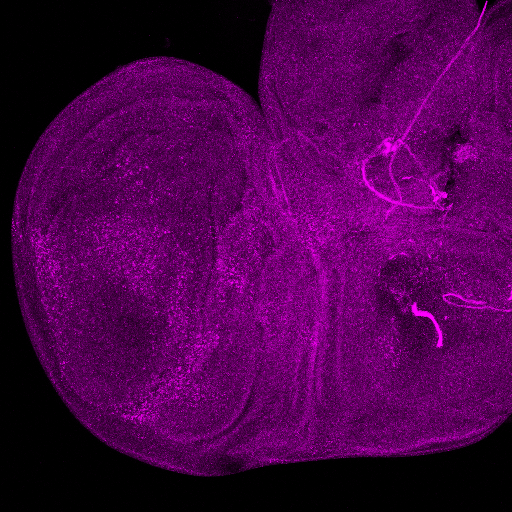

Supplement: Supplementary file 7 — Source Data for Figure 3 [file EMBJ-42-e110454-s007.zip › Fig3/FigB/synr-mCherry atg8a.tif]

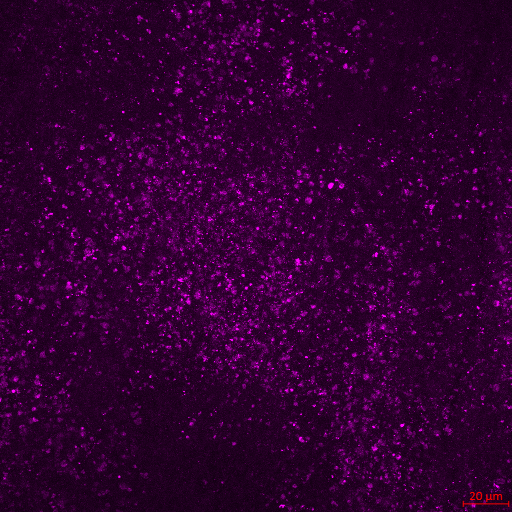

Supplement: Supplementary file 7 — Source Data for Figure 3 [file EMBJ-42-e110454-s007.zip › Fig3/FigB/magnified synr mCerry atg8a.tif]
