# Supplementary material for: Evidence for existence of an apoptosis‐inducing BH3‐only protein, sayonara, in Drosophila
Source: EMBO J. 2023 Feb 2;42(8):e110454. doi: 10.15252/embj.2021110454 (PMC10107002; doi:10.15252/embj.2021110454)
Supplement: Supplementary file 4 — PDF+ [file EMBJ-42-e110454-s004.pdf]

Article

SOURCE DATA | TRANSPARENT PROCESS | OPEN ACCESS

THE EMBO JOURNAL

Evidence for existence of an apoptosis-inducing BH3-only protein, *sayonara*, in *Drosophila*

Yuko Ikegawa[1,2], Christophe Combet[3], Mathieu Groussin[4,5,6], Vincent Navratil[7,8], Sabrina Safar-Remali[3], Takuya Shiota[9,10], Abdel Aouacheria[11] & Sa Kan Yoo[1,12,*]

Abstract

Cells need to sense stresses to initiate the execution of the dormant cell death program. Since the discovery of the first BH3-only protein Bad, BH3-only proteins have been recognized as indispensable stress sensors that induce apoptosis. BH3-only proteins have so far not been identified in *Drosophila* despite their importance in other organisms. Here, we identify the first *Drosophila* BH3-only protein and name it *sayonara*. Sayonara induces apoptosis in a BH3 motif-dependent manner and interacts genetically and biochemically with the BCL-2 homologous proteins, Buffy and Debcl. There is a positive feedback loop between Sayonara-mediated caspase activation and autophagy. The BH3 motif of *sayonara* phylogenetically appeared at the time of the ancestral gene duplication that led to the formation of *Buffy* and *Debcl* in the dipteran lineage. To our knowledge, this is the first identification of a bona fide BH3-only protein in *Drosophila*, thus providing a unique example of how cell death mechanisms can evolve both through time and across taxa.

Keywords apoptosis; BH3-only protein; caspase; cell death; *Drosophila*
Subject Categories Autophagy & Cell Death
DOI 10.15252/embj.2021110454 | Received 15 December 2021 | Revised 27 December 2022 | Accepted 28 December 2022 | Published online 2 February 2023
The EMBO Journal (2023) 42: e110454
See also: **K White** (April 2023)

Introduction

Apoptosis research blossomed from the finding of Ced3, a caspase, in *C. elegans* (Ellis & Horvitz, 1986; Igaki & Miura, 2004), followed by elucidation of the detailed molecular mechanisms of caspase activation in *C. elegans*, *Drosophila* and mammals (Budihardjo *et al*, 1999; Igaki & Miura, 2004; Riedl & Shi, 2004; Fan *et al*, 2005). In both mammals and *C. elegans*, BH3-only proteins work as important stress sensors that initiate events leading to caspase activation through BCL-2 proteins (Bouillet & Strasser, 2002; Giam *et al*, 2008; Lomonosova & Chinnadurai, 2008; Shamas-Din *et al*, 2011; Happo *et al*, 2012; Aouacheria *et al*, 2015; Doerflinger *et al*, 2015). Contrary to the situation of worms and mammals, it has been regarded over the last two decades that BH3-only proteins that induce caspase activation do not exist in *Drosophila* in spite of their importance in other organisms (Kuranaga & Miura, 2002; Quinn *et al*, 2003; Kumar & Cakouros, 2004; Denton *et al*, 2013; Banjara *et al*, 2020). For this reason, the current literature suggests that *Drosophila*, possibly all insects, might have evolved to possess a unique cell death program, which is primarily regulated by the inhibitor of apoptosis (IAP) family of proteins through their antagonists such as Reaper, Hid, and Grim (Goyal *et al*, 2000).

Since the initial finding of BH3-only proteins functioning as stress sensors that initiate caspase activation in mammals and *C. elegans* (Yang *et al*, 1995a; Conradt & Horvitz, 1998), the repertoire of BH3-only proteins expanded, now including structurally and functionally dissimilar proteins that have only distantly related BH3 sequences, which is widely denoted by the minimal hexameric sequence L-X(3)-G(or A, S)-D (Bouillet & Strasser, 2002; Giam *et al*, 2008; Lomonosova & Chinnadurai, 2008; Shamas-Din *et al*, 2011; Happo *et al*, 2012; Aouacheria *et al*, 2013, 2015; Doerflinger *et al*, 2015), and that belong to protein families with roles sometimes unrelated to cell death (Aouacheria *et al*, 2013, 2015). Interestingly, proteins such as Atg6 (Beclin) or BNIP3 were reported to contain a BH3 motif in humans and rodents (Aouacheria *et al*, 2005; Chinnadurai *et al*, 2008; Sinha & Levine, 2008), but the sequences of their respective homologs in *Drosophila* appear to be devoid of any recognizable BH3-like motif (Fig EV1A and B). Their functions are not known to be related to either caspase activation or apoptosis in flies. In this report, we focus on the classic type of BH3-only proteins that have a

1 Laboratory for Homeodynamics, RIKEN BDR, Kobe, Japan
2 Graduate School of Biostudies, Kyoto University, Kyoto, Japan
3 Centre de Recherche en Cancérologie de Lyon, UMR Inserm U1052, CNRS 5286, Université Claude Bernard Lyon 1, Centre Léon Bérard, Lyon, France
4 Laboratoire de Biométrie et Biologie Evolutive, Université de Lyon, Université Claude Bernard Lyon 1, CNRS, Villeurbanne, France
5 Department of Biological Engineering, Massachusetts Institute of Technology, Cambridge, MA, USA
6 Institute of Clinical Molecular Biology, Kiel University, Kiel, Germany
7 PRABI, Rhône-Alpes Bioinformatics Center, Université Lyon 1, Villeurbanne, France
8 UMS 3601, Institut Français de Bioinformatique, IFB-Core, Évry, France
9 Organization for Promotion of Tenure Track, University of Miyazaki, Miyazaki, Japan
10 Frontier Science Research Center, University of Miyazaki, Miyazaki, Japan
11 ISEM, Institut des Sciences de l'Evolution de Montpellier, UMR 5554, CNRS, IRD, EPHE, Université de Montpellier, Montpellier, France
12 Physiological Genetics Laboratory, RIKEN CPR, Kobe, Japan
*Corresponding author. Tel: +81 78 306 3150; E-mail: sakan.yoo@riken.jp

© 2023 The Authors. Published under the terms of the CC BY 4.0 license

typical BH3 sequence signature and that are able to activate caspases through BCL-2 family proteins.

Results

Discovery of a BH3-only protein, *sayonara*

We explored whether there is truly no fly BH3-only protein that activates caspase, as has been believed in the field over the last few decades. By performing *in silico* database analysis using BLAST searches at UniProt, we found a gene, CG14044 (Q9VMY2), whose product could be weakly aligned with the region of human BIK that includes the BH3 motif (Fig 1A). In fact, the region of this protein satisfies the definition of the BH3 motif. In addition to the BH3 motif, CG14044 has two coiled-coil domains but no other BH motif (Fig 1B). The BH3 motif exists in the interhelix region of the protein structure (Fig EV1C), which was predicted by alphafold 2 (Jumper *et al*, 2021).

CG14044 belongs to the Panther family PTHR21974, which is a relatively large, evolutionarily conserved family with its defining coiled-coil regions (http://www.pantherdb.org/panther/family.do?clsAccession=PTHR21974). Sequence and phylogenetic analyses indicate that the BH3 motif of CG14044 evolved within this protein group in the lineage leading to extant Diptera (Fig 1C). Because the BH3-only member BIK is found only in vertebrates (Rech de Laval *et al*, 2014), we interpret that the initial discovery of CG14044 using alignment with BIK was likely a fortuitous one and that the detected similarity to the BIK BH3 motif is the result of convergence or of a random event, that is, analogy rather than homology. This is consistent with the fact that most BH3-containing proteins are phylogenetically unrelated (Aouacheria *et al*, 2005, 2013) and with the view that BH3 motifs form a novel class of linear motifs (Aouacheria *et al*, 2015).

To investigate the function of CG14044, we first ectopically expressed CG14044 in the whole body of *Drosophila* using *act-Gal4* and *UAS-CG14044* (FlyORF F003800). This led to complete lethality (Fig 1D) during the larval stage. Next, we expressed CG14044 in the wing pouch to examine whether it can induce apoptosis. Expression of CG14044 induced caspase activation and DNA fragmentation in the wing disc, black melanization in the pupal wing and abnormal wings in adults (Fig 1E). In sum, CG14044 has a BH3 motif and induces caspase activation and cell death. We named this gene *sayonara* (*synr*), meaning "good bye" in Japanese.

BH3-only proteins classically induce caspase activation and apoptosis in a BH3 motif-dependent manner. To examine the importance of the BH3 motif of Synr, we generated new transgenic lines that have *UAS-synr WT* or *UAS-synr BH3 motif mutant*, which lacks the BH3 motif (Fig 1F), using the same insertion site *attp2*. Compared with the FlyORF stock, the newly generated *UAS-synr WT* induced a milder wing phenotype, reducing the wing size (Fig EV1D). The BH3 mutant did not induce the adult wing size reduction or caspase activation, which was detected with a caspase sensor, GC3Ai (Schott *et al*, 2017) (Figs 1G–I and EV1D). These data indicate that Synr induces caspase activation and apoptosis in a BH3 motif-dependent manner. Interestingly, in addition to the BH3 motif, the coiled-coil regions were also important for inducing the adult wing phenotype, as demonstrated by the phenotypes of coiled-coil domain (ccd) 1 and/or 2 deletion mutants (Fig 1J and K). We also made additional transgenics that have amino acid changes in either the BH3 motif or the coiled-coil domains rather than deletion of the regions. For the BH3 motif, we changed the four conserved hydrophobic amino acids to glutamate, as previously performed (Chen *et al*, 2005) (Fig 1L). For the coiled-coil domain formation, repetition of hydrophobic, hydrophilic, and charged amino acids is crucial (Mason & Arndt, 2004). Thus, we changed all the hydrophobic amino acids and charged amino acids to glycine (Fig EV1E). These mutations reversed the wing phenotype induced by *synr WT* (Fig 1M). To take further controls of the experimental setting, we also created transgenics with HA-tagged Synr. We confirmed that the BH3 mutations do not reduce the protein expression levels of Synr (Fig EV1F). These data indicate that the effect of the BH3 motif mutation is not due to reduced protein expression.

Synr interacts with Debcl and Buffy both genetically and biochemically

BH3-only proteins work through BCL-2 proteins (Bouillet & Strasser, 2002; Giam *et al*, 2008; Lomonosova & Chinnadurai, 2008; Shamas-Din *et al*, 2011; Happo *et al*, 2012; Aouacheria *et al*, 2015; Doerflinger *et al*, 2015). *Drosophila* has two BCL-2 proteins, Buffy and Debcl (Brachmann *et al*, 2000; Colussi *et al*, 2000; Igaki *et al*, 2000; Zhang *et al*, 2000; Quinn *et al*, 2003; Igaki & Miura, 2004; Doumanis *et al*, 2007). Debcl was originally identified as a proapoptotic BCL-2 protein (Brachmann *et al*, 2000; Colussi *et al*, 2000; Igaki *et al*, 2000; Zhang *et al*, 2000) whereas Buffy as an antiapoptotic BCL-2 protein (Quinn *et al*, 2003). Yet, later studies suggested that whether they behave as an anti or proapoptotic factor depends on cell types and physiological contexts (Igaki & Miura, 2004; Senoo-Matsuda *et al*, 2005; Doumanis *et al*, 2007; Clavier *et al*, 2015). We found that inhibition of either Buffy or Debcl suppresses the adult wing phenotype induced by Synr (Fig 2A–C and E). We also found that inhibition of the executioner caspase, Dcp-1, inhibits the Synr-induced wing phenotype (Fig 2D and E). Consistent with the adult wing results, inhibition of Debcl or Buffy suppressed Synr-mediated caspase activation, which was detected by cleavage of Dcp-1 and GC3Ai in the wing disc (Fig 2F and G). These genetic epistasis data demonstrate that *synr* and the BCL-2 family members *Debcl* and *Buffy*, genetically interact.

To examine whether they biochemically interact, we investigated biochemical binding of Synr to Debcl or Buffy. We performed GST pull-down assays using GST-Synr bound to glutathione Sepharose, and Buffy or Debcl, which was expressed in HEK293T cells. We found that Synr interacts with both BCL-2 proteins (Fig 2H and I). This strongly supports the idea to posit Synr as the caspase-activating BH3-only protein that interacts genetically and biochemically with BCL-2 proteins.

We then investigated which part of Synr is important for binding to Debcl or Buffy. In most BH3-only proteins, the BH3 motif is crucial for binding to BCL-2 proteins (Bouillet & Strasser, 2002; Giam *et al*, 2008; Lomonosova & Chinnadurai, 2008; Shamas-Din *et al*, 2011; Happo *et al*, 2012; Aouacheria *et al*, 2015; Doerflinger *et al*, 2015). Interestingly, deletion of either the BH3 motif or the coiled-coil domain did not affect Synr's binding to the BCL-2 proteins (Fig 2J–M). This indicates that Synr's physical interaction with Buffy or Debcl can occur without the BH3 motif. This seemed

 © 2023 The Authors

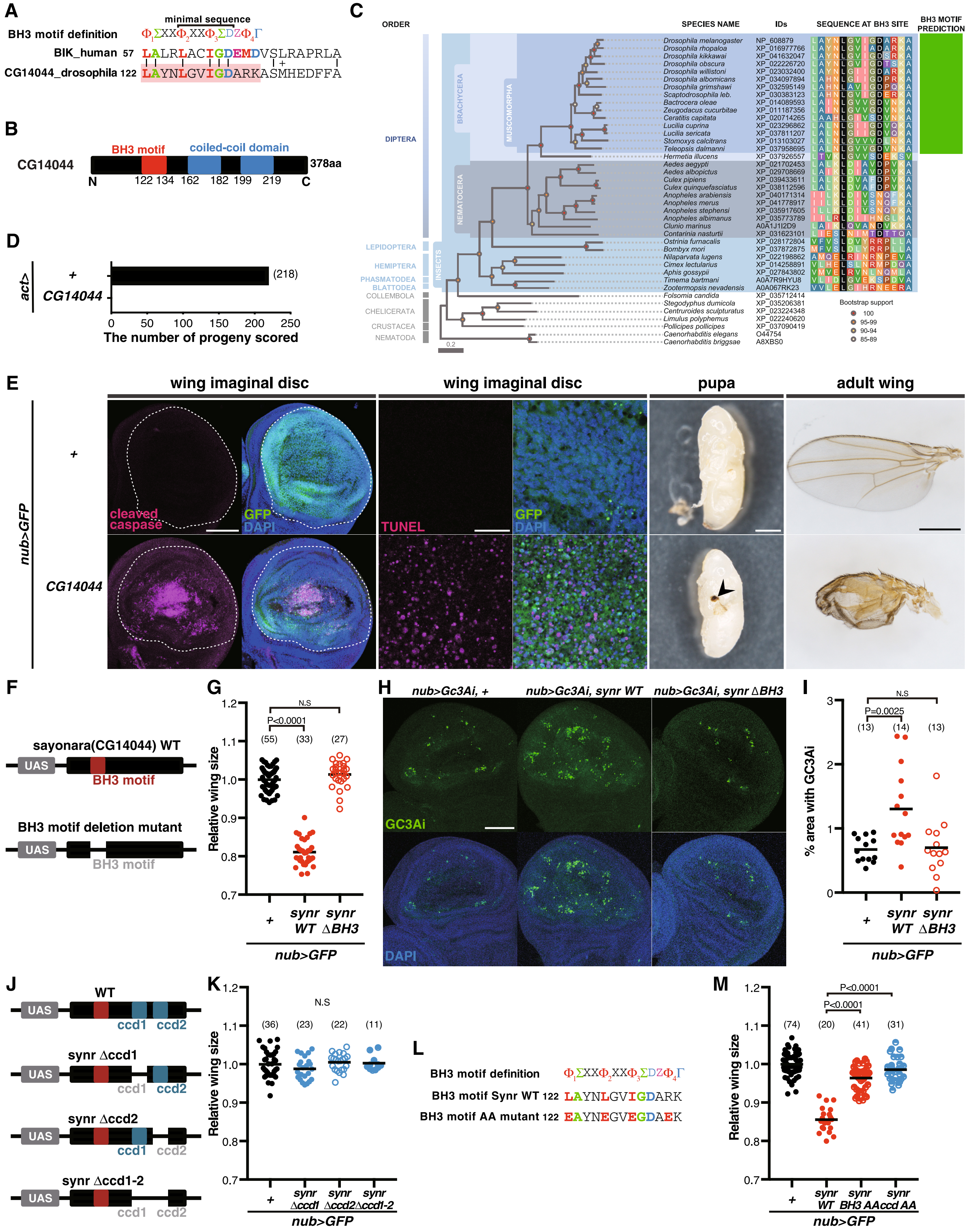
A
BH3 motif definition
minimal sequence
BIK_human 57 LALRLACIGDEMDVSLRAPRLA
CG14044_drosophila 122 LAYNLGVIGDARKASMHEDFFA
B
CG14044
BH3 motif
coiled-coil domain
378aa
N 122 134 162 182 199 219 C
C
ORDER
SPECIES NAME
IDs
SEQUENCE AT BH3 SITE
BH3 MOTIF PREDICTION
DIPTERA
LEPIDOPTERA
HEMIPTERA
PHASMATODEA
BLATTODEA
COLLEMBOLA
CHELICERATA
CRUSTACEA
NEMATODA
BRACHYCERA
MUSCOMORPHA
NEMATOCERA
INSECTS
Bootstrap support
D
act>
+
CG14044
(218)
The number of progeny scored
E
wing imaginal disc
wing imaginal disc
pupa
adult wing
nub>GFP
+
CG14044
cleaved caspase
GFP DAPI
TUNEL
GFP DAPI
F
sayonara(CG14044) WT
UAS
BH3 motif
BH3 motif deletion mutant
UAS
BH3 motif
G
Relative wing size
N.S
P<0.0001
(55) (33) (27)
+ synr WT synr ΔBH3
nub>GFP
H
nub>Gc3Ai, +
nub>Gc3Ai, synr WT
nub>Gc3Ai, synr ΔBH3
GC3Ai
DAPI
I
% area with GC3Ai
N.S
P=0.0025
(13) (14) (13)
+ synr WT synr ΔBH3
nub>GFP
J
WT
UAS
ccd1 ccd2
synr Δccd1
synr Δccd2
synr Δccd1-2
K
Relative wing size
N.S
(36) (23) (22) (11)
+ synr Δccd1 synr Δccd2 synr Δccd1-2
nub>GFP
L
BH3 motif definition
BH3 motif Synr WT 122 LAYNLGVIGDARK
BH3 motif AA mutant 122 EAYNEGVEGDAEK
M
Relative wing size
P<0.0001
P<0.0001
(74) (20) (41) (31)
+ synr WT synr BH3 AA synr ccd AA
nub>GFP

Figure 1.

Figure 1. Discovery of a BH3-only protein, *sayonara*.

A Alignment of human BIK and fly CG14044. A part of CG14044 can be aligned with a part of BIK that contains the BH3 motif. Φ, Σ, Z, and Γ indicate hydrophobic, small, acidic, and hydrophilic amino acid residues, respectively. The minimal sequence is L-X(3)-G(or A, S)-D.
B Schematic representation of CG14044.
C Neighbor-joining phylogenetic tree of Sayonara orthologs from representative ecdysozoan species. Colored circles indicate bootstrap support. Alignment of the region encompassing the L^1-X^2-X^3-X^4-X^5-D core pattern characteristic of most BH3 sequences is shown (with conserved leucine and aspartic acid residues in white on black background). The BCL2DB (Rech de Laval *et al*, 2014; Aouacheria *et al*, 2019) position-specific scoring matrix corresponding to the BH3 motif was used to scan the listed protein sequences for the presence of a BH3 motif (green box depicting positive hits). From this profile-based prediction, a BH3 motif can only be inferred in Sayonara sequences from the Brachyceran infraorder Muscomorpha.
D Ectopic expression of CG14044 with *act-Gal4* induces lethality during the larval stage.
E Ectopic expression of CG14044 in the wing pouch (GFP area indicated by the dotted line) induces caspase activation and DNA fragmentation (TUNEL) in the larval disc, melanization in the pupal wing (arrowhead), and a structural defect in the adult wing.
F Schematic representation of Sayonara WT and the mutant that lacks the BH3 motif.
G Deletion of the BH3 motif suppresses the effect of Synr on the wing size.
H Deletion of the BH3 motif suppresses Sayonara-induced caspase activation, which was detected by the caspase sensor GC3Ai.
I Quantification of GC3Ai-positive areas with Synr WT and the BH3 motif deletion mutant.
J Schematic representation of Sayonara WT and coiled-coil domain deletion mutants.
K Deletion of the coiled-coil domains suppresses the effect of Synr on the wing size.
L Amino acid sequence of the BH3 motif substitution mutant. The four well-conserved hydrophobic amino acids were changed to hydrophilic glutamic acid.
M Amino acid substitution at the BH3 motif or the coiled-coil domain suppresses the effect of Synr on the wing size.

Data information: Statistical significance was determined using one-way ANOVA with Dunnett's *post hoc* test. Scale bars, 100 μm in (E) (cleaved caspase) and (H), 20 μm in (E) (TUNEL) and 500 μm in (E) (pupae and adult wing).
Source data are available online for this figure.

at a glance contradictory from the genetic data that Synr's BH3 motif is indispensable for caspase activation (Fig 1F–M). We hypothesized that the BH3 motif may still interact with the BCL-2 proteins and play an important role albeit being dispensable for their gross interaction. To test this hypothesis, we assessed the interaction of Synr's BH3 motif and BCL-2 proteins by performing site-specific photo-crosslinking (Chin *et al*, 2002; Shiota *et al*, 2011). We incorporated p-benzoyl-L-phenylalanine (BPA) either in the BH3 motif (124Y) or in its proximity (141F) (Fig 2N). This experimentation enables assessment of the physical interaction between the BH3 motif and Buffy/Debcl. We found that UV-induced crosslinking occurs between the inserted BPA and Buffy/Debcl, demonstrating their physical interaction (Fig 2O and P).

Additionally, we found that Synr can pull down Debcl and Buffy simultaneously (Fig 2Q), suggesting a possibility that the three proteins make a complex. To further verify this possibility, we performed a sequential pull-down experiment by pulling down Flag-Buffy, followed by elution with the Flag peptide and second pull-down of Myc-Synr. This sequential pull-down experiment led to co-immunoprecipitation of HA-Debcl (Fig 2R). Based on these biochemical data and genetic data, we speculate that Synr makes a complex with Buffy and Debcl, where Synr's BH3 motif could exert a crucial effect on the protein complex's function. It is of note that Buffy and Debcl themselves interact with each other (Quinn *et al*, 2003), indicating that all the three proteins can interact with each other. The idea that Synr makes a complex with Debcl and Buffy is consistent with our genetic data that inhibition of either Debcl or Buffy is sufficient to suppress the synr-induced phenotype.

Feedback loop between apoptosis and autophagy

While we were characterizing the effects of synr expression, we unexpectedly found that Synr induces autophagy, which was detected by punctate accumulation of Lysotracker (Fig 3A) and mCherry-Atg8a (Fig 3B). Synr also colocalized with Atg8a and Rab27, which label autophagosomes (Nagy *et al*, 2015; Underwood *et al*, 2020), in the salivary gland and the wing disc (Figs 3C and EV2A). The relationship between autophagy and apoptosis is enigmatic (Gump & Thorburn, 2011; Marino *et al*, 2014; Bialik *et al*, 2018; Doherty & Baehrecke, 2018). Autophagy could occur as a byproduct of apoptosis or could affect the apoptotic process. While autophagy is in principle a mechanism for cell survival, it can induce caspase-dependent apoptosis (Scott *et al*, 2007; Mohseni *et al*, 2009; Nagata *et al*, 2019) as well as caspase-independent cell death, which is called autophagic cell death (Berry & Baehrecke, 2007; Bialik *et al*, 2018). Additionally, caspase can also induce autophagy (Hou *et al*, 2008). Which role autophagy plays depends on cellular contexts. Interestingly, inhibition of autophagy suppressed both the Synr-induced wing phenotype and cell death detected by propidium iodide and TUNEL (Figs 3D–G and EV2B–D), suggesting that Synr-induced autophagy is not just a byproduct of caspase-mediated apoptosis or counteractive for apoptosis, rather it has a positive contribution to the cell death phenotype. In addition to Synr-induced cell death, we also found that reaper (rpr), which activates apoptosis through DIAP1 inhibition (Goyal *et al*, 2000), induces autophagy (Fig EV2E and F) and that the rpr-induced phenotype in the adult wing is suppressed by autophagy inhibition (Fig EV2G).

We further clarified how autophagy regulates Synr-induced cell death. Autophagy inhibition suppressed caspase activation by Synr, in a similar manner to inhibition of Dcp-1 or Debcl (Figs 3H and I, and EV2H). On the contrary, Dcp-1 inhibition suppressed Synr-induced Lysotracker puncta formation, suggesting that caspase is necessary for Synr-induced autophagy (Fig 3J). These data indicate that there is a positive feedback loop between apoptosis and autophagy, activating each other at least in a context of Synr-induced cell death.

Endogenous function of Synr

Finally, we tested endogenous functions of *synr*. In vertebrates, BH3-only proteins often function downstream of p53 (Happo *et al*, 2012). We found that the p53-induced wing phenotype was well suppressed by *synr* RNAis that we newly generated (Figs 4A

 © 2023 The Authors

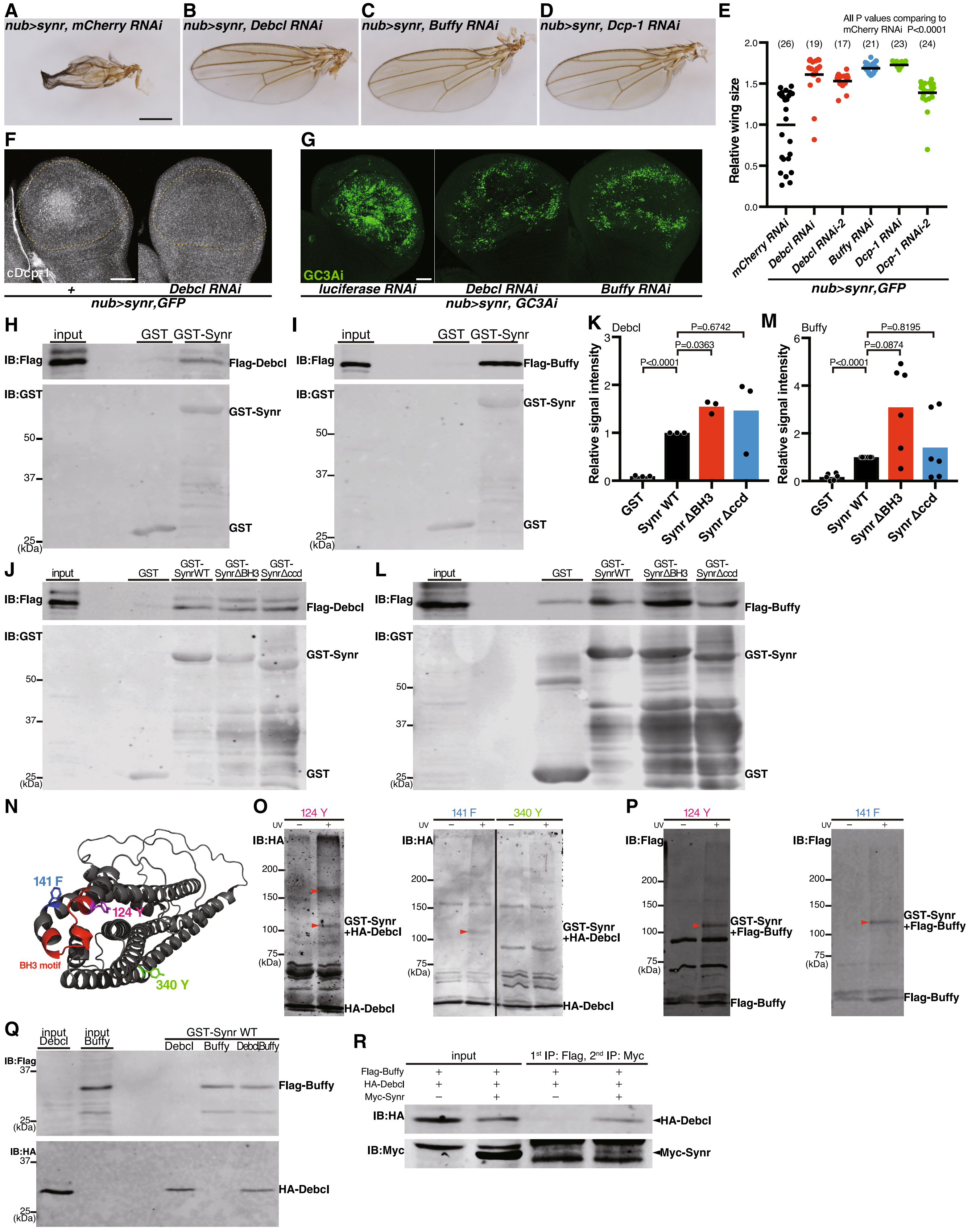
A nub>synr, mCherry RNAi
B nub>synr, Debcl RNAi
C nub>synr, Buffy RNAi
D nub>synr, Dcp-1 RNAi
E
All P values comparing to mCherry RNAi P<0.0001
Relative wing size
(26) (19) (17) (21) (23) (24)
mCherry RNAi
Debcl RNAi
Debcl RNAi-2
Buffy RNAi
Dcp-1 RNAi
Dcp-1 RNAi-2
nub>synr,GFP
F
cDcp-1
+
Debcl RNAi
nub>synr,GFP
G
GC3Ai
luciferase RNAi
Debcl RNAi
Buffy RNAi
nub>synr, GC3Ai
H
input GST GST-Synr
IB:Flag
Flag-Debcl
IB:GST
GST-Synr
GST
(kDa)
I
input GST GST-Synr
IB:Flag
Flag-Buffy
IB:GST
GST-Synr
GST
(kDa)
K
Debcl
Relative signal intensity
P<0.0001
P=0.0363
P=0.6742
GST
Synr WT
Synr ΔBH3
Synr Δccd
M
Buffy
Relative signal intensity
P<0.0001
P=0.0874
P=0.8195
GST
Synr WT
Synr ΔBH3
Synr Δccd
J
input GST GST-SynrWT GST-SynrΔBH3 GST-SynrΔccd
IB:Flag
Flag-Debcl
IB:GST
GST-Synr
GST
(kDa)
L
input GST GST-SynrWT GST-SynrΔBH3 GST-SynrΔccd
IB:Flag
Flag-Buffy
IB:GST
GST-Synr
GST
(kDa)
N
141 F
124 Y
BH3 motif
340 Y
O
124 Y
141 F
340 Y
UV
IB:HA
GST-Synr +HA-Debcl
HA-Debcl
(kDa)
P
124 Y
141 F
UV
IB:Flag
GST-Synr +Flag-Buffy
Flag-Buffy
(kDa)
Q
input Debcl
input Buffy
GST-Synr WT
Debcl Buffy Debcl Buffy
IB:Flag
Flag-Buffy
IB:HA
HA-Debcl
(kDa)
R
input
1st IP: Flag, 2nd IP: Myc
Flag-Buffy
HA-Debcl
Myc-Synr
IB:HA
HA-Debcl
IB:Myc
Myc-Synr

Figure 2.

Figure 2. Synr interacts with Debcl and Buffy both genetically and biochemically.

A–D The Synr-induced wing structural defect is suppressed by knockdown of *Debcl*, *Buffy* or *DCP1*.
E Quantification of the adult wing size with RNAis for apoptotic genes.
F Synr-induced caspase activation is suppressed by knockdown of *Debcl*.
G GC3Ai signals are suppressed by knockdown of *Debcl* or *Buffy* (quantification is shown in Fig EV2H).
H, I Lysates from HEK293T cells that express Flag-Debcl or Flag-Buffy were incubated with GST or GST-Synr bound to glutathione Sepharose. Flag-Debcl or Flag-Buffy is pulled down with GST-Synr but not with GST.
J Deletion of neither the coiled-coil domain nor the BH3 motif suppresses binding Flag-Debcl to GST-Synr.
K Quantification of the GST pull-down assay shown in (J).
L Deletion of neither the coiled-coil domain nor the BH3 motif suppresses binding of Flag-Buffy to GST-Synr.
M Quantification of the GST pull-down assay shown in (L).
N Amino acids that were replaced with BPA are shown. Tyrosine^{124} is within the BH3 motif and phenylalanine^{141} is in a close proximity to the BH3 motif. Tyrosine^{340} is for a negative control.
O Debcl can be photo-crosslinked with Synr at BPA that corresponds to Y^{124} and F^{141}. BPA at Y^{340} was used as a negative control. Red arrowheads indicate covalently crosslinked products.
P Buffy is also photo-crosslinked with Synr at BPA that corresponds to Y^{124} and F^{141}. Red arrowheads indicate covalently crosslinked products.
Q GST-Synr pulls down both Flag-Buffy and HA-Debcl simultaneously, suggesting a possibility of complex formation among Synr, Buffy and Debcl.
R HA-Debcl is detected after two series of immunoprecipitation. The first immunoprecipitation was performed for Flag-Buffy, followed by Flag peptide-mediated elution and secondary precipitation for Myc-Synr. HA-Debcl can be detected after this sequential immunoprecipitation.

Data information: Statistical significance was determined using one-way repeated measures ANOVA with Dunnett's *post hoc* test. Scale bars, 500 µm in (A) and 50 µm in (F) and (G).
Source data are available online for this figure.

and EV3A). *synr* RNAis also suppressed p53-induced caspase activation (Figs 4B and EV3B), indicating that endogenous *synr* regulates p53-mediated apoptosis.

We also examined more physiological functions of *synr*. Starvation induces caspase activation in the gut enterocytes (O'Brien *et al*, 2011). We found that *synr* inhibition suppressed starvation-induced caspase activation (Fig 4C). Interestingly, *synr* mutation or enterocyte-specific *synr* knockdown enhanced resistance to starvation (Figs 4D and E, and EV3C–E), supporting a physiological function of endogenous *synr*.

It is of note that the *synr* mutant was viable, suggesting that *synr* is not necessary for apoptosis during development, which is similar to mutants of *Buffy* or *Debcl* (Sevrioukov *et al*, 2007). However, we reproducibly found that the extensively outcrossed *synr* mutant develops slightly faster than control (Fig EV3F), suggesting that *synr* could play some role during development.

Discussion

Here, we report the original identification and characterization of the first BH3-only protein that induces caspase activation in *Drosophila*. This challenges the dogma that *Drosophila* is devoid of a BH3-only protein acting in cell death control.

One obvious question arising from our work is why a BH3-only protein was not found for over 20 years in *Drosophila*. First, *in silico* prediction of BH3-only proteins is notoriously difficult because of the degenerate (low complexity) nature of the BH3 motif signature (Aouacheria *et al*, 2013, 2015), thereby requiring experimental validation through laborious and time-consuming *in vitro* and *in vivo* assays. Another reason for missing the fly BH3-only protein for long could be related to the important roles DIAP1 and its regulators Rpr, Hid, and grim play in *Drosophila*, which probably led to underappreciation of the BCL-2 pathway.

Synr-induced caspase activation induces autophagy, which also requires caspase for its activation. Previous reports indicated that autophagy can induce caspase-dependent apoptosis (Scott *et al*, 2007; Mohseni *et al*, 2009; Nagata *et al*, 2019). On the contrary, caspases can also induce autophagy (Hou *et al*, 2008). We also observed that Rpr-mediated caspase activation induces autophagy (Fig EV2F–H). Our results integrate these observations in different systems and propose a feedback mechanism in a single setting (Fig 4F). We speculate that the feedback loop between caspase and autophagy could be of importance especially when caspase induction is relatively weak. Currently, we do not know how exactly caspase and autophagy components such as Atg proteins interact with each other. Discovering the detailed mechanistic basis of the coupling of apoptosis and autophagy is an important avenue for further study.

Contrary to the BCL-2 family of homologous proteins, which have additional BH motifs and share globular structures and common ancestry, most BH3-only proteins are phylogenetically unrelated (Aouacheria *et al*, 2005, 2013). Now defined as a particular class of short linear motifs (Aouacheria *et al*, 2015), BH3 motifs are expected to easily evolve *de novo* through convergent evolution or random incidence (homoplasy), and not exclusively through divergent evolution like BCL-2 family members (Aouacheria *et al*, 2015). Our discovery that the BH3 motif of Synr was a dipteran synapomorphy fits well into this paradigm. For example, although Cil-7 in *C. elegans* and Synr, both of which belong to the same protein family (PTHR21974), share similar coiled-coil domains in their C-terminal halves, their N-terminal regions greatly vary in length and amino acid sequence and Cil-7 does not have any recognizable BH3 motif.

Based on our genetic and biochemical data, we propose that Synr makes a complex with Debcl and Buffy, which is critical for caspase activation. In this model, both Debcl and Buffy function as proapoptotic BCL-2 proteins. This is reminiscent of the direct model rather than the indirect model in mammalian BH3-only proteins (Bouillet & Strasser, 2002; Giam *et al*, 2008; Lomonosova & Chinnadurai, 2008; Shamas-Din *et al*, 2011; Happo *et al*, 2012; Aouacheria *et al*, 2015; Doerflinger *et al*, 2015). One unique aspect of the BH3 motif of Synr is, in spite of its importance in Synr proapoptotic

 © 2023 The Authors

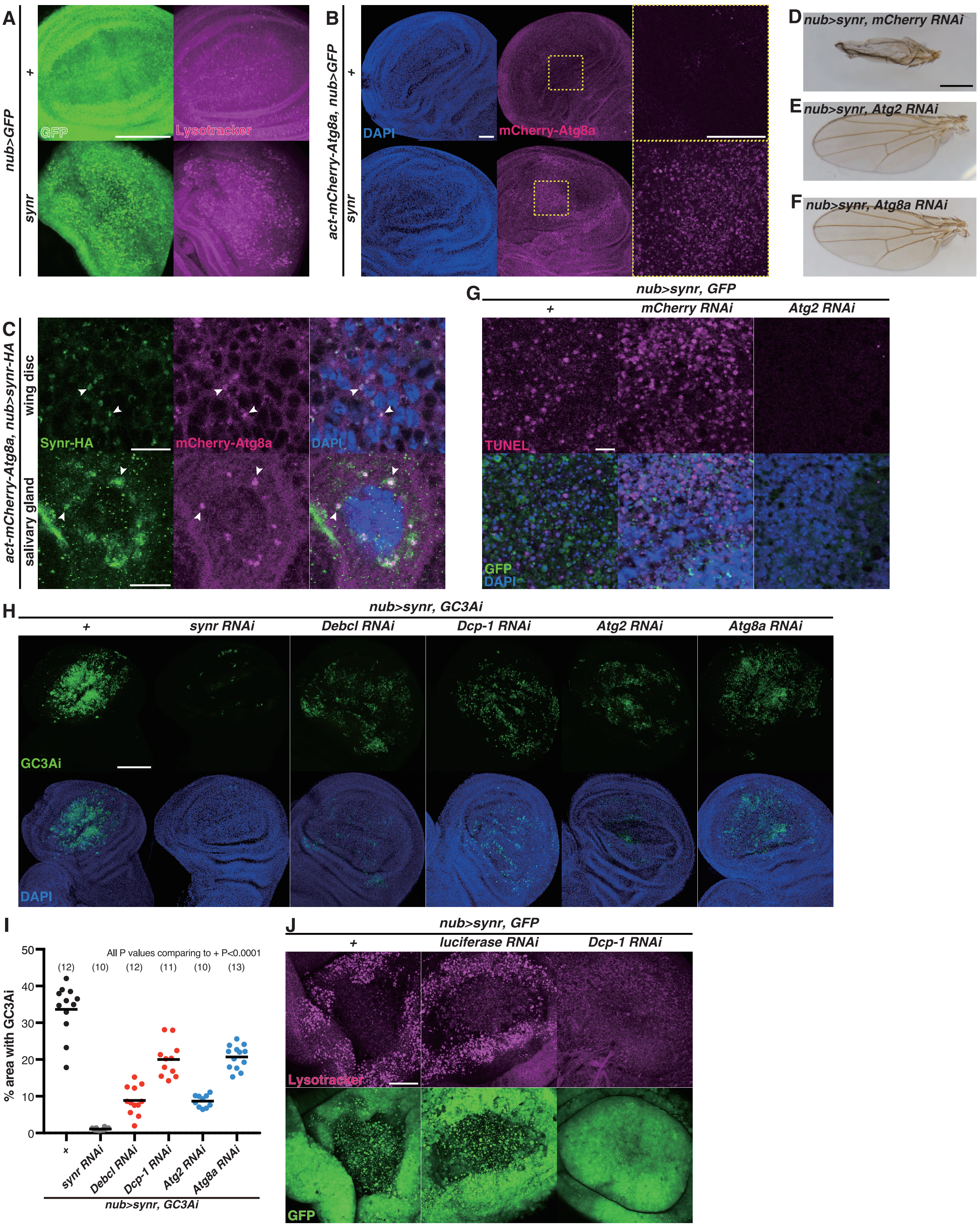
A
nub>GFP
+
synr
GFP
Lysotracker
B
act-mCherry-Atg8a, nub>GFP
DAPI
mCherry-Atg8a
D nub>synr, mCherry RNAi
E nub>synr, Atg2 RNAi
F nub>synr, Atg8a RNAi
C
act-mCherry-Atg8a, nub>synr-HA
wing disc
salivary gland
Synr-HA
mCherry-Atg8a
DAPI
G
nub>synr, GFP
+
mCherry RNAi
Atg2 RNAi
TUNEL
GFP
DAPI
H
nub>synr, GC3Ai
+
synr RNAi
Debcl RNAi
Dcp-1 RNAi
Atg2 RNAi
Atg8a RNAi
GC3Ai
DAPI
I
All P values comparing to + P<0.0001
(12) (10) (12) (11) (10) (13)
% area with GC3Ai
0
10
20
30
40
50
+
synr RNAi
Debcl RNAi
Dcp-1 RNAi
Atg2 RNAi
Atg8a RNAi
nub>synr, GC3Ai
J
nub>synr, GFP
+
luciferase RNAi
Dcp-1 RNAi
Lysotracker
GFP

Figure 3.

◀ **Figure 3. Feedback loop between apoptosis and autophagy.**

A Ectopic expression of Synr induces autolysosome accumulation, which was detected by Lysotracker, in the wing disc.
B Ectopic expression of Synr also induces autophagosome accumulation, which was detected by mCherry-Atg8a. The dotted square regions are magnified in the right pictures.
C Synr is colocalized with mCherry-Atg8a (arrowheads) in the wing disc and the salivary gland at 112 h AEL.
D–F Knockdown of *Atg2* or *Atg8a* suppresses the wing structural defect that is induced by Synr.
G *Atg2* knockdown suppresses Synr-induced cell death, which was detected by TUNEL staining.
H, I Synr-induced caspase activation is suppressed by inhibition of autophagy (*Atg2*, *Atg8a* RNAis) in a similar manner to apoptosis inhibition (*Debcl*, *Dcp-1* RNAis).
J Synr-induced autolysosome accumulation is suppressed by *Dcp-1* knockdown.

Data information: Statistical significance was determined using one-way ANOVA with Dunnett's *post hoc* test. Scale bars, 100 μm in (A) and (H), 50 μm in (B) and (J), 10 μm in (C) and (G), and 500 μm in (D).
Source data are available online for this figure.

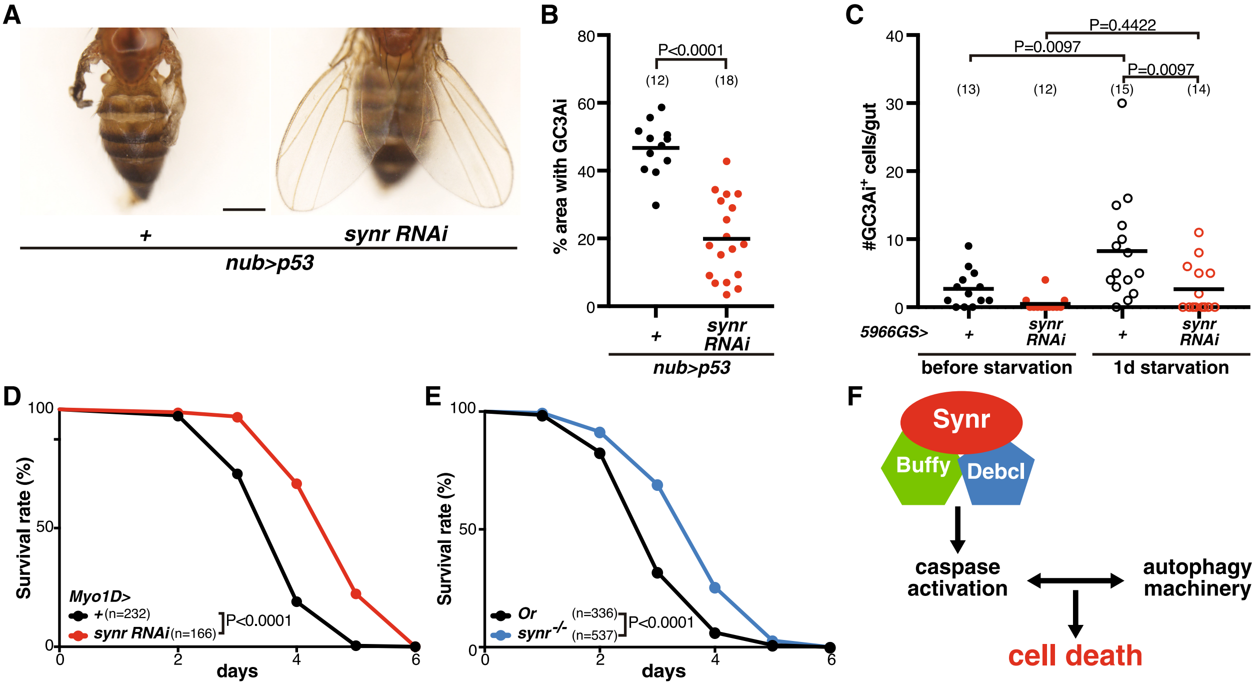

Figure 4. Endogenous function of Synr.

A Inhibition of Synr suppresses the wing defect that is induced by p53, a potent apoptosis inducer.
B Inhibition of Synr suppresses p53-induced caspase activation.
C Inhibition of Synr in the midgut enterocytes suppresses starvation-induced caspase activation in the midgut.
D Inhibition of Synr in the midgut enterocytes enhances resistance to starvation.
E The *synr* mutant also exhibits enhanced resistance to starvation.
F A proposed model of Synr-induced cell death. Synr, which functions together with Buffy and Debcl, induces initial caspase activation, which leads to feedback loop between apoptosis and autophagy.

Data information: Statistical significance was determined using a two-tailed unpaired *t*-test (B), one-way ANOVA with Holm-Sidak's multiple comparisons test (C), and a log-rank (Mantel–Cox) test (D, E). Scale bar, 500 μm in (A).
Source data are available online for this figure.

activity, being dispensable for Synr overall binding to Debcl/Buffy. At the same time, the BH3 motif binds to Debcl/Buffy, as demonstrated by the photo-crosslinking at the BH3 motif. We speculate that the local binding of Synr BH3 and Debcl/Buffy is critical for the function of the protein complex. Future protein structural analyses will reveal the molecular details by which Synr-BH3 motif exerts its function.

Our findings provide a unique insight into how a molecular machinery of apoptosis can be formed through possible coevolution of several genes. Our phylogenetic analyses suggest that a recognizable BH3 motif of *synr* appeared (Fig 1C) at the time (or soon afterwards) a gene duplication produced the pair of paralogous genes *Buffy* and *Debcl* in Diptera (Fig EV4A). We inferred and synthesized the sequence of the putative ancestor of Buffy/Debcl, which we named dBorg0, based on ancestral protein reconstruction (Fig EV4B). dBorg0 could biochemically bind to all of Synr WT, the coiled-coil deletion mutant and the BH3 deletion mutant (Fig EV4C–F), which is similar to Debcl and Buffy. Based on the

 © 2023 The Authors

evolutionary history of Synr, Debcl and Buffy and biochemical binding data (Figs 2H–R and EV4), we speculate the following evolutionary scenario. A BH3 motif evolved in the coiled-coil domain-containing protein in a subset of Diptera (that led to extant Muscomorpha or Brachycera), giving birth to *synr* with a BH3 motif. At a similar timing, in the lineage that led to modern Diptera, duplication of the ancestral BCL-2 homologous gene *dBorg0* led to the generation of the *Debcl* and *Buffy* pair of paralogous proteins. The two paralogs underwent functional divergence and subsequent specialization of their protein–protein interaction network, leading to functionalization of the apoptosis-inducing machinery complex Synr-Debcl-Buffy.

Materials and Methods

Drosophila husbandry

Flies were maintained as described previously (Yoo *et al*, 2016). The fly food was composed of 0.8% agar, 10% glucose, 4.5% corn flour, 3.72% dry yeast, 0.4% propionic acid, and 0.3% butyl p-hydroxybenzoate. All experiments were performed at 25°C except Figs 4A and B, and EV3B, which were conducted at 18°C.

Drosophila stocks

The fly stocks used in this study are listed in Dataset EV1. We also generated the following fly stocks by combining the listed stocks:
nub-gal4, *UAS-GFP*; *UAS-synr*

nub-gal4; *UAS-GC3Ai*

nub-gal4; *UAS-synr*

nub-gal4, *UAS-GFP*; *act-mCherry-Atg8a*

vg-gal4, *UAS-GFP*; *UAS-rpr*

nub-gal4; *UAS-p53*

5966GS-gal4; *UAS-GC3Ai*
$synr^{-/-}$ was made by extensive outcross of $CG14044^{MI09290}$ (50506, Bloomington Stock Center) to Oregon R. The MiMIC insertion was confirmed by PCR.

Immunofluorescence and imaging

Wing discs or adult midguts were immunostained as described previously (Nishida *et al*, 2021; Sasaki *et al*, 2021). We used the following antibodies and fluorescent dyes at the indicated dilutions: cleaved Dcp1 antibody (1:500; 9578, Cell Signaling Technology), HA antibody (1:500; 901513, BAB), DAPI (1:500; D9542, Sigma), and Alexa Fluor secondary antibodies (1:500; A11008 and A11036, Thermo Fisher). Fluorescence images were acquired with a confocal microscope (Zeiss LSM 780, 880, 900). To measure the wing size, wings were imaged with a stereo microscope (Nikon SMZ18). The wing size was quantified using Fiji. To quantify the percentage of GC3Ai-positive areas, images were binarized with a certain intensity threshold with Fiji, and the percentage was calculated to the area with gene expression, which was determined by the GC3Ai background signal and morphology.

DNA construction

For making transgenic flies, *synr* WT (FBpp0078629), *synr* BH3 motif deletion mutant, *synr* ccd deletion mutant, *synr* BH3 motif amino acid change mutant, or *synr* ccd amino acid change mutant was cloned into the pUASTattB vector. *synr* BH3 deletion mutant was made by overlap PCR to remove the BH3 motif (residues 122–131). *synr* coiled-coil domain mutants were also generated similarly. We removed residues 162–182 for *synr dcc1*, residues 199–219 for *synr dcc2*, and residues 162–219 for *synr dcc1-2*. Regarding AA substitution mutants, BH3 motif AA mutant was generated by overlap PCR. The ccd AA mutant was newly synthesized by GenScript.

The amino acid sequences of BH3 motif AA mutant and ccd AA mutant are the following:
BH3 motif AA mutant, EAYNEGVEAAAEK (122–134); ccd AA mutant, ccd1, GGGTGGGGGTGGGSYGGGNGG (162–182), ccd2, GTGGGGSGGGQGGTGGGTGGG (199–219)

For HA-tagged Synr, *synr WT*, *ΔBH3*, and *BH3AA* mutants were once inserted into the pCMV-HA-N vector (Takara #635690). Then, the tagged *synr* constructs were amplified by PCR and cloned into the pUASTattB vector. To knockdown *synr*, we generated shRNAi lines based on the Trip protocol by Jian-Quan Ni and Norbert Perrimon at Harvard Medical School (https://fgr.hms.harvard.edu/files/fly/files/2ndgenprotocol.pdf). The following oligos were annealed and cloned into the pVALIUM20 vector.

synr -RNAi:
ctagcagtTCGGCAAGTCCTAGCCGTCAAtagttatattcaagcataTTGACGGCTAGGACTTGCCGAgcg

aattcgcTCGGCAAGTCCTAGCCGTCAAtatgcttgaatataactaTTGACGGCTAGGACTTGCCGAactg

synr -RNAi-2:
ctagcagtAACGAGCTGTACGTAAAGCAAtagttatattcaagcataTTGCTTTACGTACAGCTCGTTgcg

aattcgcAACGAGCTGTACGTAAAGCAAtatgcttgaatataactaTTGCTTTACGTACAGCTCGTTactg

Transgenic flies were generated by BestGene.

For protein expression in *E. coli*, *synr WT*, *ΔBH3*, and *Δccd1-2* mutants were cloned into a pCold-GST DNA (Takara #3372) with an N-terminal GST tag.

For protein expression in HEK293T cells, both Buffy (FBpp0087182) and Debcl (FBpp0085443) were cloned into a Flag vector (a gift from Dr Nishimura), where the Flag tag was added to the N terminus. We also generated HA-Debcl with pCMV-HA-N vector (Takara #635690). Myc tag was also added at the N terminus of *synr* with pCMV-Myc vector (Takara #635689). Regarding the dBorg0 experiment, based on the amino acid sequence of dBorg0 (Fig EV4B), the following DNA sequence was synthesized with the human codon usage and cloned into the Flag vector (GenScript):

ATGCTGAGCACCACCGCCCCCCCCACCACCACCACCACCGCCAGCA
GCAACCCCAGCCCCAACAGCAACAGCGTGAGCCCCAGCAGAAACC
TGGCCAACCTGCACAGCAGCCCCCTGAACGGCGACCACCACCCCC
CCAGCAGCAGAAGAAGACTGAGCAACGTGAGCGACGTGGTGACC
AGAAAGCTGAGCAGCACCATCGGCTGGAAGCAGGCCGTGATCCCC
AGCCAGGACATCATCACCCAGGGCAAGTGCCTGTGCGGCCAGTA
CATCAGAAGCAGACTGAAGAGAAGCGGCGTGTTCAACAGAAAGC
TGGGCCTGCAGAGACTGAGAAGCATCGTGGGCGTGCCCACCGTG
CACGTGGTGAGAGAGGTGTTCCCCGCCCTGCTGAGCCTGGGCGAG
GAGCTGGAGAGAATGCACCCCAGAGTGTACACCAACGTGGCCAGA
CAGATCAGCAGAAGCCCCAGAGGCGAGCTGAAGGAGCCCGACACC
GCCCCCGTGCTGCTGAGCGCCATCGCCAGAGACCTGTTCAGAAGC
GACATCACCTGGGGCAAGGTGGTGAGCCTGTTCGCCATCGCCGGC
GGCCTGGCCGTGGACTGCGTGAGACAGGGCCACCCCGACTACCTG
CAGAAGCTGATCGAGGGCGTGGCCGACGTGATCGAGGACGACCTG
GCCACCTGGATCGCCGAGAACGGCGGCTGGGTGGGCCTGGCCAAC
CACGTGAGACCCCCCCAGGAGGAGATCACCTTCGTGGGCAGATGC
CTGGGCCTGCTGGCCCTGTTCATGGCCGTGAAGTTCCTGGTGTACC
TGATCCTGCAGTGGCTGCAGAAGCTGGAGAGCCAGTACGACAGCC
AGCTGTAA.

To generate *synr* mutant constructs for BPA photo-crosslinking assay, a codon of the target amino acids was changed to the amber codon TAG in the GST-synr plasmid by PCR with Primestar Mutagenesis Basal kit (Takara #R046A).

Protein preparation

GST proteins (GST only, GST-Synr, GST-Synr ΔBH3, and GST-Synr Δccd) were expressed in BL21 *E. coli* (Takara #9126) and purified by affinity for glutathione Sepharose (GE healthcare). After preculture for 4 h, we applied a cold shock according to the manufacturer's instruction and incubated in LB media with 0.1 mM IPTG at 15°C for 20 h. Cell pellets were isolated by centrifugation and lysed by sonication in the *E. coli* lysis buffer (PBS, 1% NP40, 250 μM PMSF). GST proteins were purified by affinity for glutathione Sepharose. After washing beads, lysates that contain Flag-tagged proteins or HA-tagged proteins were added. To make the lysates, HEK293T cells were lysed in the cell lysis buffer (pH 7.4 50 mM Hepes, 2% NP40, 150 mM NaCl, 5% Glycerol, 1 mM MgCl2, 1 mM MnCl2, 10 mM NaF, 1 mM Na3VO4, 10 μg/ml Leupeptin, 2 μg/ml Pepstatin, 0.1% Aprotinin, 1% Protease inhibitor cocktail (Roche, cOmplete, EDTA free), 0.1% Phosphatase inhibitor cocktail 2 (P5726, Sigma), 0.1% Phosphate inhibitor cocktail 3 (P0044, Sigma), 250 μM PMSF and 1 mM DTT), and cell lysates were cleaned by centrifugation. After washing the GST-bound beads with the cell lysis buffer, proteins were eluted by heating at 95°C for 5 min in the 2× sample buffer and they were separated by SDS–PAGE (8% SDS–PAGE gels).

Protein preparation for photo-crosslinking assay

Plasmids for GST proteins and pEVOL-pBpF (#31190), which includes tRNA synthetase/tRNA pair to incorporate pBPA into proteins, were co-transformed to pG-Tf2/BL21 *E. coli* (Takara #9124), and they were cultured LB medium with 1 mM p-benzoyl-L-phenylalanine and 0.1% L-arabinose. Then, cell lysis, GST purification, and incubation with lysates that contain Flag-tagged protein or HA-tagged protein were performed as described previously. Lysates were cleaned by centrifugation, and the GST-bound beads were exposed to UV for 15 min. Washing and elution were performed as described previously. Proteins were separated by SDS–PAGE (6% SDS–PAGE gels).

Sequential IP

All of Myc-Synr, HA-Debcl, and Flag-Buffy were expressed in HEK293T cells. Their lysates were incubated with the monoclonal mouse ANTI-FLAG M2 antibody (F1804, Sigma) and Protein A/G Magnetic Beads (#88802, Thermo Fisher) for 4 h. After washing beads, proteins were eluted with 200 μg/ml 3XFlag peptide and then elution was again incubated with new Protein A/G Magnetic Beads and Myc-tag mouse antibody (9B11, #2276, Cell Signaling Technology) O/N. After washing the beads with the cell lysis buffer, proteins were eluted by heating at 95°C for 5 min in the 2× sample buffer and they were separated by SDS–PAGE (8% SDS–PAGE gels).

Western blotting analysis

Proteins were separated on an SDS–PAGE gel and transferred to a nitrocellulose membrane (Bio-Rad Laboratories, Inc.). After blocking with 5% BSA, the membranes were immunoblotted with the following antibodies and fluorescent dyes at the indicated dilutions: GST rabbit antibody (1:1,000; 2622S, Cell Signaling Technology, or 1:1,000; 10000-0-AP, Proteintech), Monoclonal mouse ANTI-FLAG M2 antibody (1:1,000; F1804, Sigma), HA-Tag Rabbit antibody (1:1,000; 3724, Cell Signaling Technology), Anti-HA.11 Epitope Tag mouse antibody (1:1,000; 901513, BioLegend), Myc-tag mouse antibody (1:1,000, #2276, Cell Signaling Technology), IRDye® 680RD Goat anti-Rabbit IgG Secondary Antibody (1:10,000; 925-68071, LI-COR), and IRDye® 800CW Goat anti-Mouse IgG Secondary Antibody (1:10,000; 925-32210, LI-COR). Bands were visualized with a near-infrared fluorescence imaging scanner (LI-COR, Odyssey CLx). The signal intensity was measured with Image Studio from LI-COR.

Terminal deoxynucleotidyl transferase dUTP nick end labeling (TUNEL) assay

The TUNEL assay was performed using the ApopTag Red *in situ* apoptosis detection kit (Millipore) as described previously (Ciesielski *et al*, 2022). Wandering 3rd instar larvae were dissected in 1×PBS and fixed for 30 min in 1×PBS with 4% paraformaldehyde at room temperature (RT). After fixation, samples were washed with PBS 0.1% Triton-X100 and incubated in equilibration buffer (Apop Tag kit; Millipore) for 10 s. Then, samples were incubated in reaction buffer (TdT enzyme; ratio 7:3; Apop Tag kit) at 37°C for 1 h. The TdT reaction mix was replaced with stop buffer (diluted 1:34 in dH2O; Apop Tag kit) and incubated for 10 min at RT. Samples were then washed with PBS 0.1% Triton-X100 3 times and incubated with antidigoxigenin antibody solution (diluted 31:34 in blocking solution; ApopTag kit) overnight at 4°C. Samples were then washed with PBS 0.1% Triton-X100 3 times again and mounted on the slide glass.

Lysotracker staining

Wandering 3rd instar larvae were dissected in PBS and incubated with 50 μM LysoTracker Red DND-99 (L7528, Invitrogen) in PBS for

 © 2023 The Authors

5 min. After washing with PBS, samples were fixed with 4% PFA in PBS for 20 min. Then, samples were washed once with PBSTx and incubated with PBSTx containing DAPI (4 μg/ml) for 5 min. After washing with PBSTx, we observed the samples immediately with a confocal microscope.

PI staining

Wing discs in L3 larvae were dissected in PBS, incubated with Propidium Iodide (100 nM, 341-07881, Fujifilm) in PBS immediately after dissection, then fixed with 4% PFA in PBS, and washed in PBSTx.

Starvation assay

For both measuring the starvation resistance and caspase activation, 5–7-day-old virgin female flies were used. The starvation food contained only 1.5% agar. To measure starvation resistance, we counted the number of dead flies every day. For the analysis of caspase activation, we specifically induced GC3Ai expression in ECs with 5966-GS. We added 100 μM RU486 (M1732, TCI) to both the normal food and the starvation food.

Genotyping for CG14044^{MI09290}

To confirm CG14044^{MI09290} has a MIMIC insertion, we designed the primers to detect 500 bp surrounding the MIMIC insertion site. Since MIMIC is too large to amplify, the PCR does not work in CG14044^{MI09290}.

Quantification of developmental timing

To quantify developmental timing, we calculated the pupariation rate. Mated females were allowed to lay eggs on a grape agar plate for 8 h at 25°C. First, instar larvae were collected from the grape agar plate and placed into the normal food. Each vial contained 50 larvae. The number of new pupae of each genotype was recorded every 12 h. Pupariation rates were calculated as the number of pupariated flies divided by the total number of pupae at the end of each experiment.

Sayonara orthologue identification, alignment, and phylogenetic reconstruction

To infer the evolutionary history of Sayonara, we first queried Panther (16.0) to identify gene family members related to CG14044. We found that CG14044 belonged to Panther family PTHR21974. As our aim was to characterize the emergence of a BH3 motif in the Sayonara orthogroup rather than to build a comprehensive phylogeny with remote homologs, we restricted our analysis to nonredundant sequences from Ecdysozoa. Among 816 hits matching Panther family PTHR21974, we extracted 109 protein sequences from which 69 were selected for further analysis. Manual curation was performed to exclude identical, nearly identical or partial sequences as well as sequences from overrepresented taxa (e.g., *Drosophila*). BLAST searches against NCBI and UniProtKB databases were conducted one-by-one to find additional sequences in taxa of interest leading to a final dataset containing 41 representative sequences. Sequence E0W198 from *Pediculus humanus* (Psocodea) was unusual and excluded from subsequent analysis in that it has a significantly truncated C terminus. Sequences were aligned using MUSCLE, and the alignment was edited manually at ambiguous sites and at N- and C-terminal ends. A neighbor-joining tree was constructed using the BioNJ algorithm as implemented in SeaView (version 5) and Poisson distances (gaps included), with 1,000 bootstrap replicates. The tree was rooted using CIL-7 sequences from *Caenorhabditis*. The topology of our final tree is in accordance with previously published general phylogenies of insects and Diptera (Misof *et al*, 2014).

Profile-based methods for domain composition and BH3 motif prediction

Sayonara domain composition was assessed using HMM profile searches with the HMMER package (v3.0 March 2010). Briefly, we searched the UniProtKB 2021_03 database to retrieve homologs of fly CG14044 using the BLASTp algorithm, and the new sequences (obtained with E-value < 1e^{-6}, length ≥ 135 or 341 residues) were aligned using Clustal W 1.8 to construct two HMM profiles with *hmmbuild*. The full-length sequences (n = 52) of Sayonara (378 residues in *Drosophila melanogaster*) or the first 150 amino acids (present in n = 41 sequences) were used as inputs to build a first profile for the whole Sayonara orthogroup and a second profile specific for the N-terminal variable domain. These two profiles were searched against the UniProtKB 2021_03 sequence database by *hmmsearch*, resulting in 215 and 60 hits, respectively (E-value ≤ 0.001). Venn diagram analysis indicated that 60 sequences were common to both profiles, 155 sequences were exclusive to the first profile (full-length sequences), and no sequence was exclusive to the second profile (N-terminal domain). We predicted the presence or absence of BH3 motifs in the N-terminal domain of Sayonara orthologous sequences using evolutionary information provided by a position-specific scoring matrix (PSSM) profile. As previously described (Rech de Laval *et al*, 2014; Aouacheria *et al*, 2019), a PSSM profile of BH3 motifs was computed as part of an *ab initio* motif discovery procedure by running the *meme* program of the MEME software suite 4.9.0 using a set of 158 amino acid sequences of BCL-2 homologous proteins and BH3-only proteins. The PSSM was used to scan the set of 41 selected sequences for BH3 motif occurrences using the program *mast* from the MEME suite.

Characterization of the *Buffy–Debcl* duplication event

A total of 224 amino acid sequences (present in 201 different species) showing significative similarity to Drosophila *Buffy* and *Debcl* were collected from translated insect transcriptomes produced by the 1KITE (1K Insect Transcriptome Evolution) project (Misof *et al*, 2014). A profile HMM specific to both paralogous protein sequences was built and then used to screen the 1KITE transcriptome assemblies with the program hmmsearch (after translating the transcripts into all six possible reading frames). Only hits with a global e-value ≤ 10^{-5} and that were predicted to contain a BH motif (using Batch CD-search) were considered for subsequent analysis. Buffy and Debcl protein sequences were also searched against the nonredundant protein (NR) and Reference Sequence (RefSeq) databases using BLASTP, leading to an additional set of 39 sequences

(in 25 species). All 263 sequences were aligned with MUSCLE and trimmed with GBLOCKS. The phylogenetic tree was reconstructed using the UL3 amino acid substitution model with PhyML-Structure (Guindon & Gascuel, 2003; Le & Gascuel, 2010) and rooted using Collembola as the outgroup.

Ancestral sequence reconstruction and experimental resurrection

Ancestral sequence reconstruction (ASR) was carried out as described previously (Groussin *et al*, 2015), accounting for the uncertainty in the reconstruction of the gene tree and gene duplication and loss. We used Phyldog (Boussau *et al*, 2013) to reconcile the gene tree with the species tree. We used 18S ribosomal RNA sequences, when available in the NR database (32 sequences), to reconstruct the species tree. The reconciled species tree-aware gene tree was used as the guiding tree in Prank to realign Buffy–Debcl sequences. We used bppML (Dutheil & Boussau, 2008) to compare the capacity of different site-homogeneous and site-heterogenous models substitution models to fit the data and found that the site-heterogenous EX_EHO model was best at fitting the data according to the BIC criterion. We then performed ASR with the ML parameter estimates obtained with the EX_EHO model, using bppAncestor (Dutheil & Boussau, 2008) with the marginal ASR approach (Yang *et al*, 1995b). For a given site at a given internal node of the reconciled tree, the amino acid having the maximum posterior probability was inferred as the putative ancestral amino acid. Gap positions in ancestral sequences were introduced *a posteriori* based on inferences performed by Prank during sequence alignment. The putative Buffy–Debcl ancestor was named "Borg0" after dBorg-1 (one of the synonyms for Debcl) and dBorg-2 (Buffy).

Statistical analysis

Statistical tests used are indicated in the figure captions. Sample sizes were determined empirically based on the observed effects. Blinding was not applicable because the investigators who set up the experiments were the same with the ones doing sample collection and analyses. However, each experiment was associated with proper controls, and samples were collected and analyzed under identical conditions. All the statistical analyses were conducted in GraphPad Prism.

Data availability

This study includes no data deposited in external repositories.

Expanded View for this article is available online.

Acknowledgements
We thank Takashi Nishimura for the Flag vector, and Iswar Hariharan, Erina Kuranaga, Magali Suzanne and Eric Baehrecke, and TRiP at Harvard Medical School, the Bloomington Stock Center, the Kyoto DGGR Stock Center, and the Zurich ORFeome project for fly stocks. Some of the computational analyses were done using the computing facilities of the CC LBBE/PRABI-amsb. We thank Ralph Peters, Malte Petersen, Karen Meusemann, Xin Zhou, and Bernard Misof for sharing homologous sequences of Debcl and Buffy obtained from the 1 KITE project. We also thank Héloïse Philippon and Guy Perrière at PBIL/PRABI for their initial contribution. We thank the Yoo lab members, Fumiaki Obata, Yu-Chiun Wang, Takashi Nishimura, and Shigehiro Kuraku for their helpful comments on the manuscript. This work was supported by RIKEN Junior Research Associate Program to YI and AMED-PRIME (17939907), JSPS KAKENHI (JP16H06220, JP22H02807), and JST FOREST (JPMJFR216F) to SKY.

Author contributions
Yuko Ikegawa: Conceptualization; data curation; formal analysis; investigation; visualization; methodology; writing – review and editing. **Christophe Combet:** Data curation; software; formal analysis; investigation; visualization; methodology; writing – review and editing. **Mathieu Groussin:** Data curation; software; formal analysis; investigation; visualization; methodology; writing – review and editing. **Vincent Navratil:** Resources; data curation; methodology; writing – review and editing. **Sabrina Safar-Remali:** Investigation. **Takuya Shiota:** Methodology. **Abdel Aouacheria:** Data curation; formal analysis; supervision; investigation; visualization; methodology; writing – review and editing. **Sa Kan Yoo:** Conceptualization; formal analysis; supervision; funding acquisition; investigation; visualization; writing – original draft; project administration; writing – review and editing.

Disclosure and competing interests statement
The authors declare that they have no conflict of interest.

References

Aouacheria A, Brunet F, Gouy M (2005) Phylogenomics of life-or-death switches in multicellular animals: Bcl-2, BH3-only, and BNip families of apoptotic regulators. *Mol Biol Evol* 22: 2395 – 2416

Aouacheria A, Rech de Laval V, Combet C, Hardwick JM (2013) Evolution of Bcl-2 homology motifs: homology versus homoplasy. *Trends Cell Biol* 23: 103 – 111

Aouacheria A, Combet C, Tompa P, Hardwick JM (2015) Redefining the BH3 death domain as a 'short linear motif'. *Trends Biochem Sci* 40: 736 – 748

Aouacheria A, Navratil V, Combet C (2019) Database and bioinformatic analysis of BCL-2 family proteins and BH3-only proteins. *Methods Mol Biol* 1877: 23 – 43

Banjara S, Suraweera CD, Hinds MG, Kvansakul M (2020) The Bcl-2 family: ancient origins, conserved structures, and divergent mechanisms. *Biomolecules* 10: 128

Berry DL, Baehrecke EH (2007) Growth arrest and autophagy are required for salivary gland cell degradation in *Drosophila*. *Cell* 131: 1137 – 1148

Bialik S, Dasari SK, Kimchi A (2018) Autophagy-dependent cell death - where, how and why a cell eats itself to death. *J Cell Sci* 131: jcs215152

Bouillet P, Strasser A (2002) BH3-only proteins - evolutionarily conserved proapoptotic Bcl-2 family members essential for initiating programmed cell death. *J Cell Sci* 115: 1567 – 1574

Boussau B, Szollosi GJ, Duret L, Gouy M, Tannier E, Daubin V (2013) Genome-scale coestimation of species and gene trees. *Genome Res* 23: 323 – 330

Brachmann CB, Jassim OW, Wachsmuth BD, Cagan RL (2000) The *Drosophila* bcl-2 family member dBorg-1 functions in the apoptotic response to UV-irradiation. *Curr Biol* 10: 547 – 550

Budihardjo I, Oliver H, Lutter M, Luo X, Wang X (1999) Biochemical pathways of caspase activation during apoptosis. *Annu Rev Cell Dev Biol* 15: 269 – 290

Chen L, Willis SN, Wei A, Smith BJ, Fletcher JI, Hinds MG, Colman PM, Day CL, Adams JM, Huang DC (2005) Differential targeting of prosurvival Bcl-2

© 2023 The Authors

proteins by their BH3-only ligands allows complementary apoptotic function. *Mol Cell* 17: 393 – 403

Chin JW, Martin AB, King DS, Wang L, Schultz PG (2002) Addition of a photocrosslinking amino acid to the genetic code of *Escherichia coli*. *Proc Natl Acad Sci USA* 99: 11020 – 11024

Chinnadurai G, Vijayalingam S, Gibson SB (2008) BNIP3 subfamily BH3-only proteins: mitochondrial stress sensors in normal and pathological functions. *Oncogene* 27: S114 – S127

Ciesielski HM, Nishida H, Takano T, Fukuhara A, Otani T, Ikegawa Y, Okada M, Nishimura T, Furuse M, Yoo SK (2022) Erebosis, a new cell death mechanism during homeostatic turnover of gut enterocytes. *PLoS Biol* 20: e3001586

Clavier A, Ruby V, Rincheval-Arnold A, Mignotte B, Guenal I (2015) The *Drosophila* retinoblastoma protein, Rbf1, induces a Debcl- and Drp1-dependent mitochondrial apoptosis. *J Cell Sci* 128: 3239 – 3249

Colussi PA, Quinn LM, Huang DC, Coombe M, Read SH, Richardson H, Kumar S (2000) Debcl, a proapoptotic Bcl-2 homologue, is a component of the *Drosophila melanogaster* cell death machinery. *J Cell Biol* 148: 703 – 714

Conradt B, Horvitz HR (1998) The *C. elegans* protein EGL-1 is required for programmed cell death and interacts with the Bcl-2-like protein CED-9. *Cell* 93: 519 – 529

Denton D, Aung-Htut MT, Kumar S (2013) Developmentally programmed cell death in *Drosophila*. *Biochim Biophys Acta* 1833: 3499 – 3506

Doerflinger M, Glab JA, Puthalakath H (2015) BH3-only proteins: a 20-year stock-take. *FEBS J* 282: 1006 – 1016

Doherty J, Baehrecke EH (2018) Life, death and autophagy. *Nat Cell Biol* 20: 1110 – 1117

Doumanis J, Dorstyn L, Kumar S (2007) Molecular determinants of the subcellular localization of the *Drosophila* Bcl-2 homologues DEBCL and BUFFY. *Cell Death Differ* 14: 907 – 915

Dutheil J, Boussau B (2008) Non-homogeneous models of sequence evolution in the Bio++ suite of libraries and programs. *BMC Evol Biol* 8: 255

Ellis HM, Horvitz HR (1986) Genetic control of programmed cell death in the nematode *C. elegans*. *Cell* 44: 817 – 829

Fan TJ, Han LH, Cong RS, Liang J (2005) Caspase family proteases and apoptosis. *Acta Biochim Biophys Sin (Shanghai)* 37: 719 – 727

Giam M, Huang DC, Bouillet P (2008) BH3-only proteins and their roles in programmed cell death. *Oncogene* 27: S128 – S136

Goyal L, McCall K, Agapite J, Hartwieg E, Steller H (2000) Induction of apoptosis by *Drosophila* reaper, hid and grim through inhibition of IAP function. *EMBO J* 19: 589 – 597

Groussin M, Hobbs JK, Szollosi GJ, Gribaldo S, Arcus VL, Gouy M (2015) Toward more accurate ancestral protein genotype-phenotype reconstructions with the use of species tree-aware gene trees. *Mol Biol Evol* 32: 13 – 22

Guindon S, Gascuel O (2003) A simple, fast, and accurate algorithm to estimate large phylogenies by maximum likelihood. *Syst Biol* 52: 696 – 704

Gump JM, Thorburn A (2011) Autophagy and apoptosis: what is the connection? *Trends Cell Biol* 21: 387 – 392

Happo L, Strasser A, Cory S (2012) BH3-only proteins in apoptosis at a glance. *J Cell Sci* 125: 1081 – 1087

Hou YC, Chittaranjan S, Barbosa SG, McCall K, Gorski SM (2008) Effector caspase Dcp-1 and IAP protein Bruce regulate starvation-induced autophagy during *Drosophila melanogaster* oogenesis. *J Cell Biol* 182: 1127 – 1139

Igaki T, Miura M (2004) Role of Bcl-2 family members in invertebrates. *Biochim Biophys Acta* 1644: 73 – 81

Igaki T, Kanuka H, Inohara N, Sawamoto K, Nunez G, Okano H, Miura M (2000) Drob-1, a *Drosophila* member of the Bcl-2/CED-9 family that promotes cell death. *Proc Natl Acad Sci USA* 97: 662 – 667

Jumper J, Evans R, Pritzel A, Green T, Figurnov M, Ronneberger O, Tunyasuvunakool K, Bates R, Zidek A, Potapenko A *et al* (2021) Highly accurate protein structure prediction with AlphaFold. *Nature* 596: 583 – 589

Kumar S, Cakouros D (2004) Transcriptional control of the core cell-death machinery. *Trends Biochem Sci* 29: 193 – 199

Kuranaga E, Miura M (2002) Molecular genetic control of caspases and JNK-mediated neural cell death. *Arch Histol Cytol* 65: 291 – 300

Le SQ, Gascuel O (2010) Accounting for solvent accessibility and secondary structure in protein phylogenetics is clearly beneficial. *Syst Biol* 59: 277 – 287

Lomonosova E, Chinnadurai G (2008) BH3-only proteins in apoptosis and beyond: an overview. *Oncogene* 27: S2 – S19

Marino G, Niso-Santano M, Baehrecke EH, Kroemer G (2014) Self-consumption: the interplay of autophagy and apoptosis. *Nat Rev Mol Cell Biol* 15: 81 – 94

Mason JM, Arndt KM (2004) Coiled coil domains: stability, specificity, and biological implications. *Chembiochem* 5: 170 – 176

Misof B, Liu S, Meusemann K, Peters RS, Donath A, Mayer C, Frandsen PB, Ware J, Flouri T, Beutel RG *et al* (2014) Phylogenomics resolves the timing and pattern of insect evolution. *Science* 346: 763 – 767

Mohseni N, McMillan SC, Chaudhary R, Mok J, Reed BH (2009) Autophagy promotes caspase-dependent cell death during *Drosophila* development. *Autophagy* 5: 329 – 338

Nagata R, Nakamura M, Sanaki Y, Igaki T (2019) Cell competition is driven by autophagy. *Dev Cell* 51: 99 – 112

Nagy P, Varga A, Kovacs AL, Takats S, Juhasz G (2015) How and why to study autophagy in *Drosophila*: it's more than just a garbage chute. *Methods* 75: 151 – 161

Nishida H, Okada M, Yang L, Takano T, Tabata S, Soga T, Ho DM, Chung J, Minami Y, Yoo SK (2021) Methionine restriction breaks obligatory coupling of cell proliferation and death by an oncogene Src in *Drosophila*. *Elife* 10: e59809

O'Brien LE, Soliman SS, Li X, Bilder D (2011) Altered modes of stem cell division drive adaptive intestinal growth. *Cell* 147: 603 – 614

Quinn L, Coombe M, Mills K, Daish T, Colussi P, Kumar S, Richardson H (2003) Buffy, a *Drosophila* Bcl-2 protein, has anti-apoptotic and cell cycle inhibitory functions. *EMBO J* 22: 3568 – 3579

Rech de Laval V, Deleage G, Aouacheria A, Combet C (2014) BCL2DB: database of BCL-2 family members and BH3-only proteins. *Database (Oxford)* 2014: bau013

Riedl SJ, Shi Y (2004) Molecular mechanisms of caspase regulation during apoptosis. *Nat Rev Mol Cell Biol* 5: 897 – 907

Sasaki A, Nishimura T, Takano T, Naito S, Yoo SK (2021) White regulates proliferative homeostasis of intestinal stem cells during ageing in *Drosophila*. *Nat Metab* 3: 546 – 557

Schott S, Ambrosini A, Barbaste A, Benassayag C, Gracia M, Proag A, Rayer M, Monier B, Suzanne M (2017) A fluorescent toolkit for spatiotemporal tracking of apoptotic cells in living *Drosophila* tissues. *Development* 144: 3840 – 3846

Scott RC, Juhasz G, Neufeld TP (2007) Direct induction of autophagy by Atg1 inhibits cell growth and induces apoptotic cell death. *Curr Biol* 17: 1 – 11

Senoo-Matsuda N, Igaki T, Miura M (2005) Bax-like protein Drob-1 protects neurons from expanded polyglutamine-induced toxicity in *Drosophila*. *EMBO J* 24: 2700 – 2713

Sevrioukov EA, Burr J, Huang EW, Assi HH, Monserrate JP, Purves DC, Wu JN, Song EJ, Brachmann CB (2007) *Drosophila* Bcl-2 proteins participate in

© 2023 The Authors

stress-induced apoptosis, but are not required for normal development. *Genesis* 45: 184 – 193

Shamas-Din A, Brahmbhatt H, Leber B, Andrews DW (2011) BH3-only proteins: orchestrators of apoptosis. *Biochim Biophys Acta* 1813: 508 – 520

Shiota T, Mabuchi H, Tanaka-Yamano S, Yamano K, Endo T (2011) *In vivo* protein-interaction mapping of a mitochondrial translocator protein Tom22 at work. *Proc Natl Acad Sci USA* 108: 15179 – 15183

Sinha S, Levine B (2008) The autophagy effector Beclin 1: a novel BH3-only protein. *Oncogene* 27: S137 – S148

Underwood R, Wang B, Carico C, Whitaker RH, Placzek WJ, Yacoubian TA (2020) The GTPase Rab27b regulates the release, autophagic clearance, and toxicity of alpha-synuclein. *J Biol Chem* 295: 8005 – 8016

Yang E, Zha J, Jockel J, Boise LH, Thompson CB, Korsmeyer SJ (1995a) Bad, a heterodimeric partner for Bcl-XL and Bcl-2, displaces Bax and promotes cell death. *Cell* 80: 285 – 291

Yang Z, Kumar S, Nei M (1995b) A new method of inference of ancestral nucleotide and amino acid sequences. *Genetics* 141: 1641 – 1650

Yoo SK, Pascoe HG, Pereira T, Kondo S, Jacinto A, Zhang X, Hariharan IK (2016) Plexins function in epithelial repair in both *Drosophila* and zebrafish. *Nat Commun* 7: 12282

Zhang H, Huang Q, Ke N, Matsuyama S, Hammock B, Godzik A, Reed JC (2000) *Drosophila* pro-apoptotic Bcl-2/Bax homologue reveals evolutionary conservation of cell death mechanisms. *J Biol Chem* 275: 27303 – 27306

License: This is an open access article under the terms of the Creative Commons Attribution License, which permits use, distribution and reproduction in any medium, provided the original work is properly cited.

Expanded View Figures

Figure EV1. Alignment of noncanonical BH3-only proteins and a structure of CG14044.

A Alignment of Beclin in humans, chicken, xenopus, zebrafish, and fly demonstrates that the BH3 motif (the red rectangle is the core sequence of the BH3 motif; the pink rectangle is the sequence surrounding the core BH3 motif) is not conserved in the fly. An * (asterisk) indicates positions which have a single, perfectly conserved residue. (A:) (colon) indicates conservation between groups of strongly similar properties. (A.) (period) indicates conservation between groups of weakly similar properties.

B Alignment of Bnip3 in humans, chicken, xenopus, zebrafish, and fly demonstrates that the BH3 motif (the red rectangle is the core sequence of the BH3 motif; the pink rectangle is the sequence surrounding the core BH3 motif) is not conserved in the fly.

C The BH3 motif (LAYNLGVIGDARK) of CG14044 exists in the interhelix region of the protein structure, which was predicted by alphafold 2.

D A representative image of overlayed adult wings: black, + (control); magenta, Synr WT; blue, Synr ΔBH3.

E Amino acid sequence of the coiled-coil domain substitution mutant. All the hydrophobic and charged amino acids were changed to glycine.

F HA-tagged Synr was expressed in the wing pouch. The BH3 deletion or BH3 amino acid change does not reduce the Synr expression levels compared with Synr WT. Two independent pictures of each condition are shown.

Data information: Scale bars, 500 μm in (D) and 50 μm in (F).
Source data are available online for this figure.

© 2023 The Authors

© 2023 The Authors

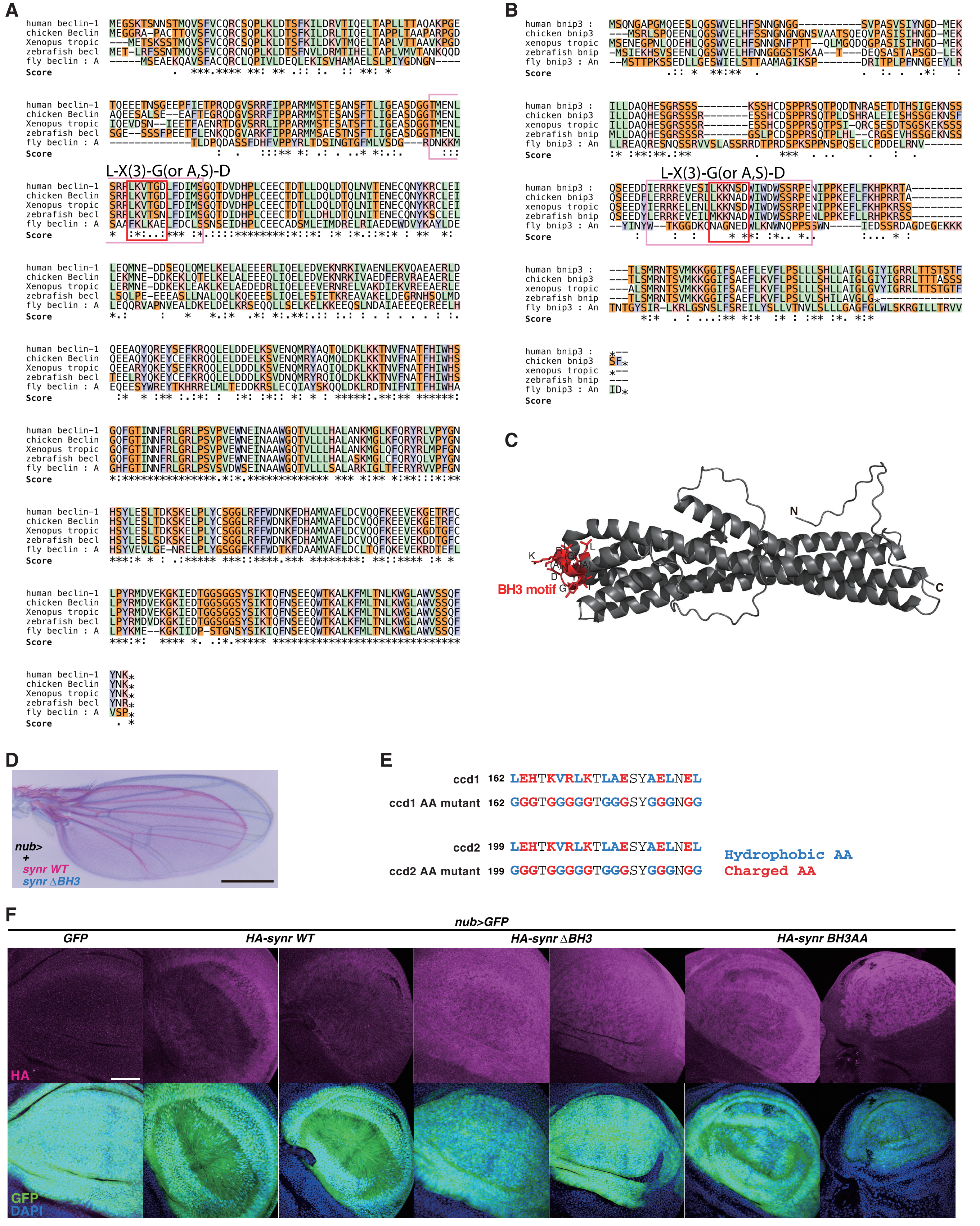

Figure EV1.

 © 2023 The Authors

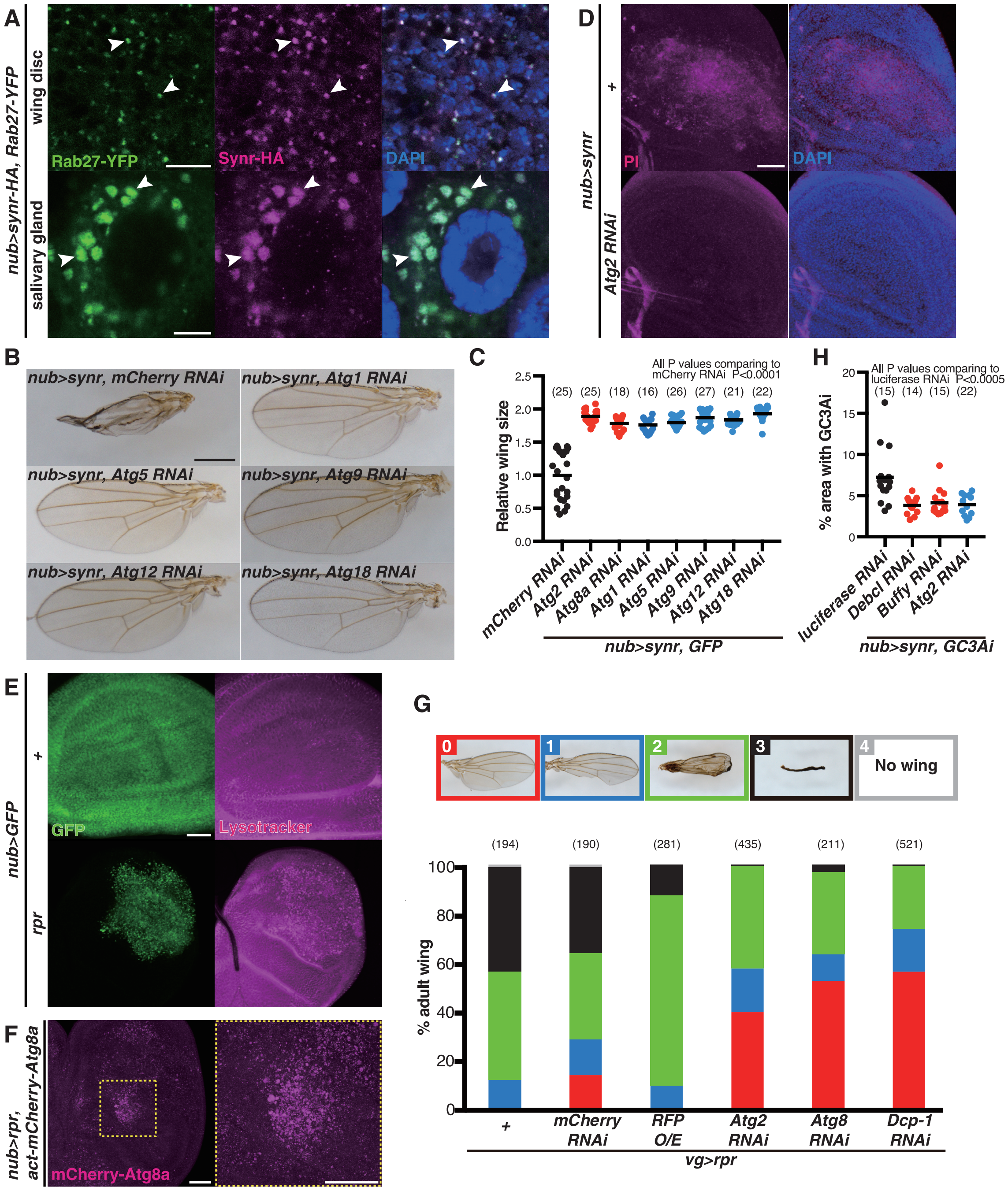

Figure EV2. Synr-induced autophagy.

A Synr is colocalized with Rab27 (arrowheads) in the wing disc and the salivary gland at 112 h AEL.
B The Synr-induced wing structural defect is suppressed by autophagy inhibition.
C Quantification of the wing size with a combination of Synr expression and autophagy inhibition.
D *Atg2* knockdown suppresses Synr-induced cell death, which was detected by propidium iodide (PI).
E A DIAP1 inhibitor, reaper induces autolysosome accumulation in the wing disc.
F Reaper also induces autophagosome accumulation in the wing disc. The yellow-dotted square region is magnified in the right picture.
G Reaper-induced wing defects are suppressed by autophagy inhibition. Wing phenotypes were divided into five classes based on the severity as indicated in pictures.
H Knockdown of *Debcl*, *Buffy*, or *Atg2* significantly suppresses caspase activation compared with the control RNAi for luciferase.

Data information: Statistical significance was determined using one-way ANOVA with Dunnett's *post hoc* test. Scale bars, 10 μm in (A), 500 μm in (B), and 50 μm in (D–F).
Source data are available online for this figure.

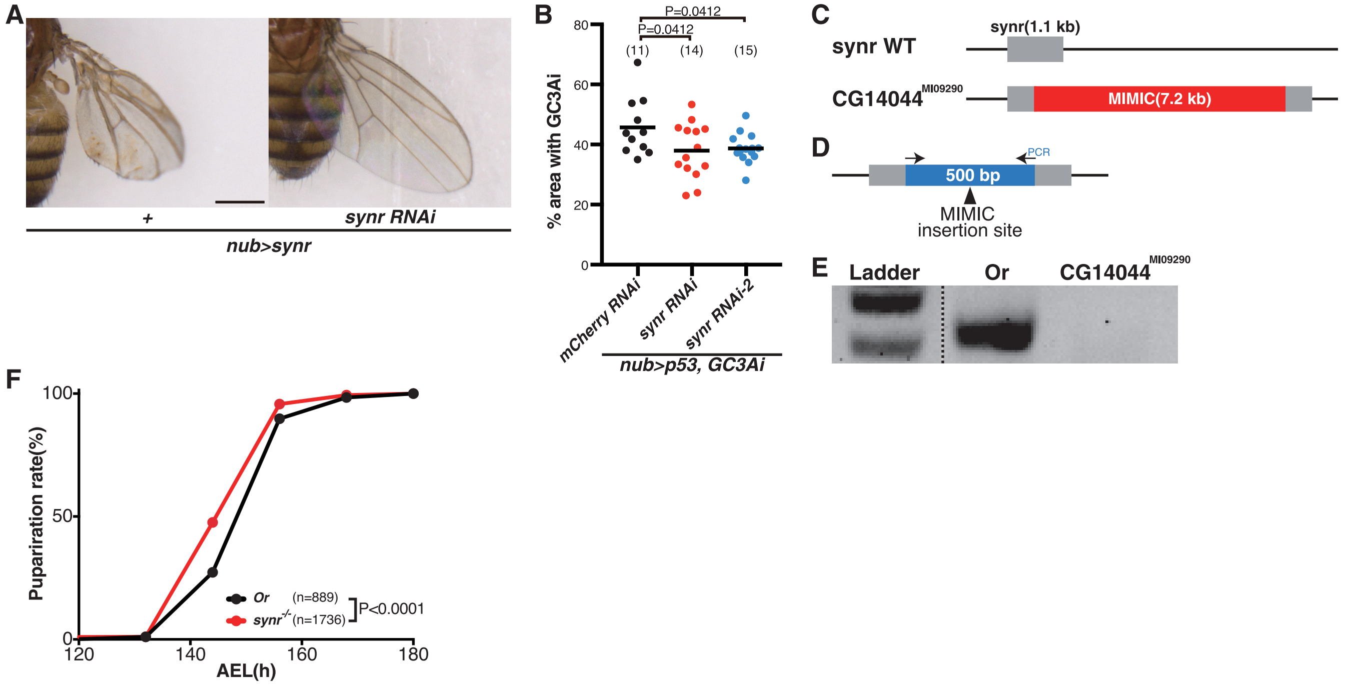

Figure EV3. Knockdown and knockout of *synr*.

A A newly generated *synr* RNAi line can suppress the effect of ectopic *synr* expression on wings.
B Two RNAis for *synr* can suppress p53-mediated caspase activation, which is detected by GC3Ai.
C Schematic of MiMIC (Minos-mediated integration cassette) insertion in *synr* (CGCG14044^{MI09290}).
D A design of primers to detect MiMIC insertion.
E Using the primers, the MiMIC insertion was confirmed by PCR. During extensive outcross of CGCG14044^{MI09290} to OregonR, MiMIC insertion was confirmed by PCR.
F The extensively outcrossed *synr* mutant demonstrates a slight, but reproducible developmental delay during development.

Data information: Statistical significance was determined using one-way ANOVA with Holm-Sidak's multiple comparisons test (B) and a log-rank (Mantel–Cox) test (F). Scale bar, 500 μm in (A).
Source data are available online for this figure.

 © 2023 The Authors

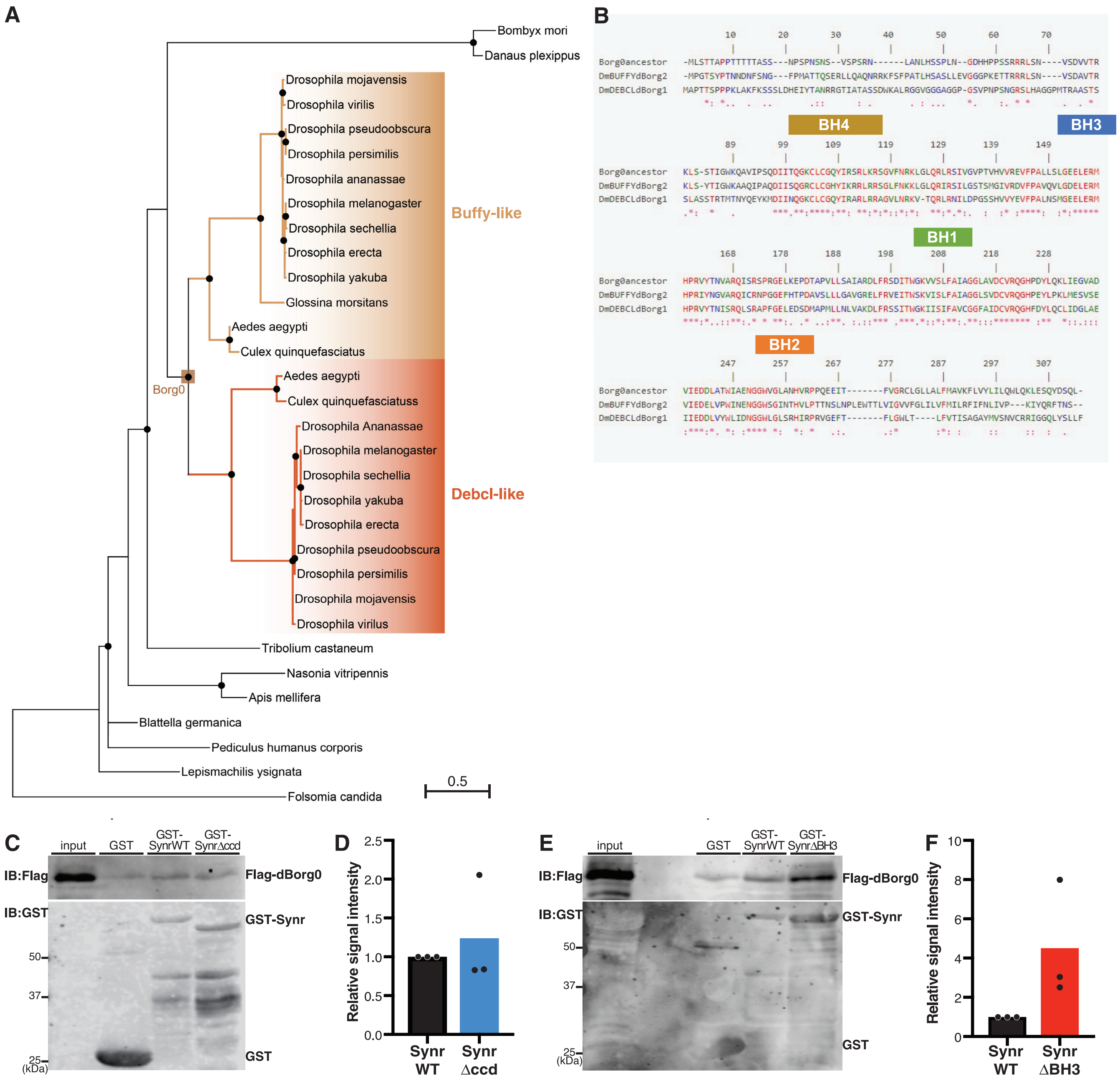

Figure EV4. Evolution of the Buffy–Debcl orthology group in insects.

A Phylogenetic subtree depicting the Buffy–Debcl duplication in Diptera. The Buffy–Debcl ancestral protein before duplication was named dBorg0, since Debcl and Buffy are also known as dBorg-1 and dBorg-2, respectively. The scale bar indicates 0.5 substitution per site.

B The amino acid sequence of dBorg0 was predicted based on ancestral protein reconstruction (see Materials and Methods). Multiple sequence alignment of *Drosophila* Debcl (also known as dBorg-1) and Buffy (dBorg-2) amino acid sequences with that of their putative ancestor dBorg0.

C–F Lysates from HEK293T cells that express Flag-dBorg0 were incubated with GST, GST-Synr WT, GST-Synr ΔBH3, or GST-Synr Δcoiled-coil mutant, which was bound to glutathione Sepharose. Synr WT, Synr BH3 mutant, and Synr coiled-coil domain mutant can pull down dBorg0 in a similar manner, indicating that the coiled-coil region and the BH3 motif are dispensable for Synr's binding to dBorg0, which is similar to Buffy and Debcl.

Source data are available online for this figure.
