# Supplementary figures and images for: Evidence for existence of an apoptosis‐inducing BH3‐only protein, sayonara, in Drosophila (part 2 of 2)
Source: EMBO J. 2023 Feb 2;42(8):e110454. doi: 10.15252/embj.2021110454 (PMC10107002; doi:10.15252/embj.2021110454)

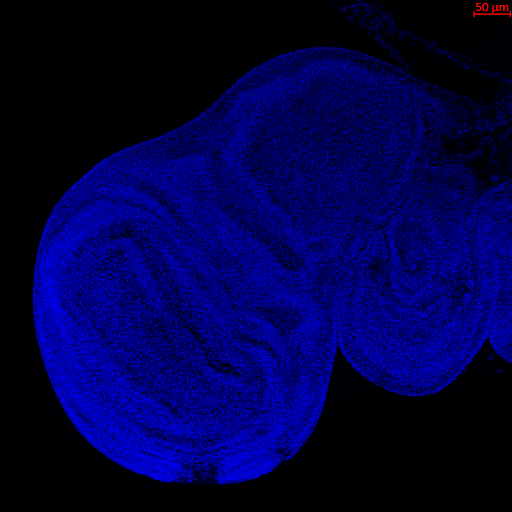

Supplement: Supplementary file 7 — Source Data for Figure 3 [file EMBJ-42-e110454-s007.zip › Fig3/FigB/+-DAPI.tif]

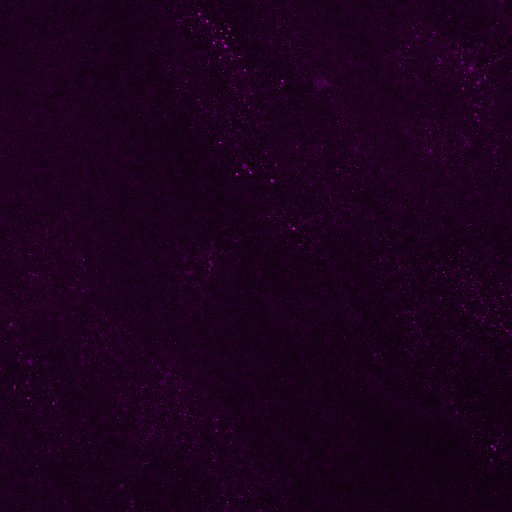

Supplement: Supplementary file 7 — Source Data for Figure 3 [file EMBJ-42-e110454-s007.zip › Fig3/FigB/magnified + mCherry atg8a.tif]

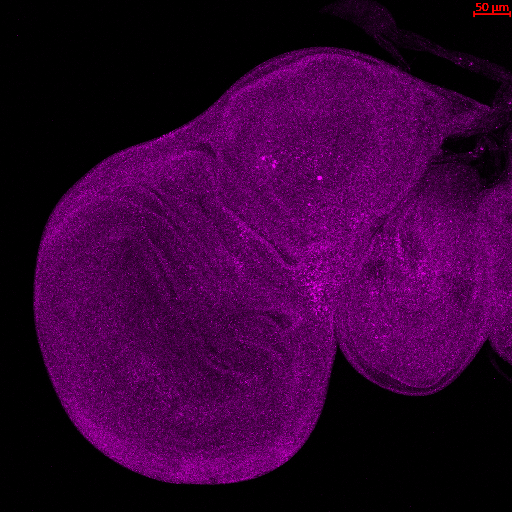

Supplement: Supplementary file 7 — Source Data for Figure 3 [file EMBJ-42-e110454-s007.zip › Fig3/FigB/+-mCherry atg8a.tif]

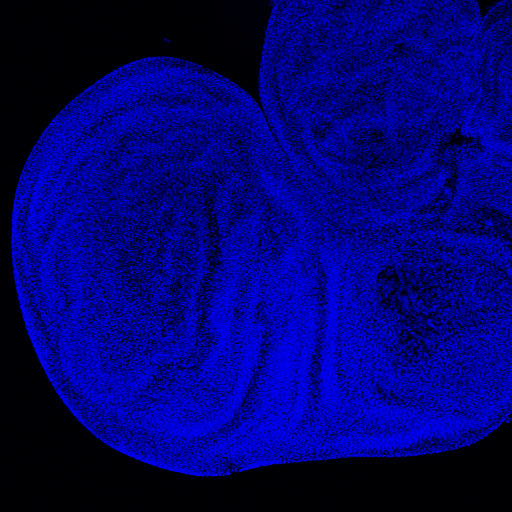

Supplement: Supplementary file 7 — Source Data for Figure 3 [file EMBJ-42-e110454-s007.zip › Fig3/FigB/synr-DAPI.tif]

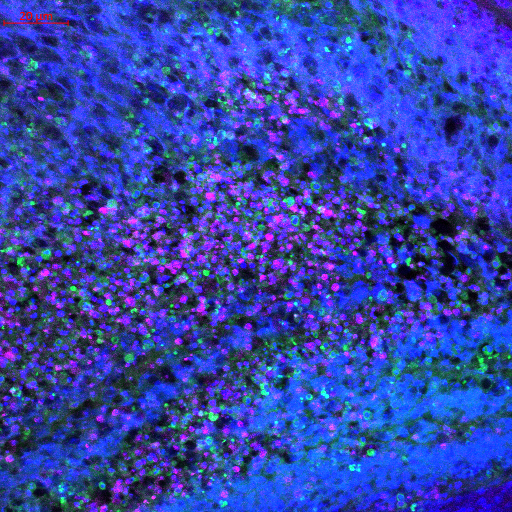

Supplement: Supplementary file 7 — Source Data for Figure 3 [file EMBJ-42-e110454-s007.zip › Fig3/FigG/mCherry RNAi-merge.tif]

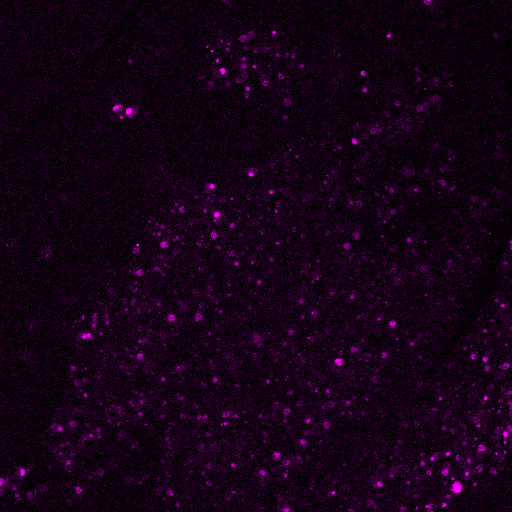

Supplement: Supplementary file 7 — Source Data for Figure 3 [file EMBJ-42-e110454-s007.zip › Fig3/FigG/w--Tunel.tif]

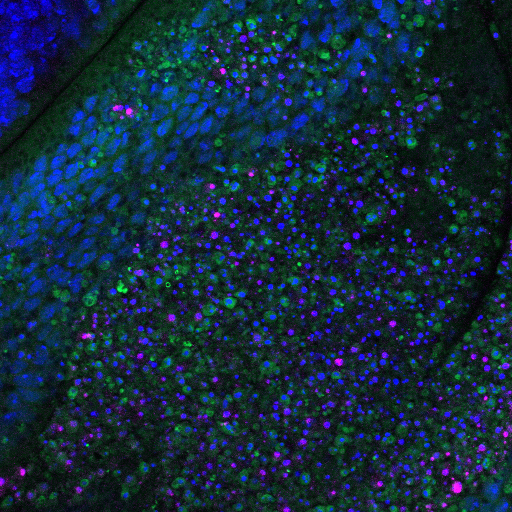

Supplement: Supplementary file 7 — Source Data for Figure 3 [file EMBJ-42-e110454-s007.zip › Fig3/FigG/w--merge.tif]

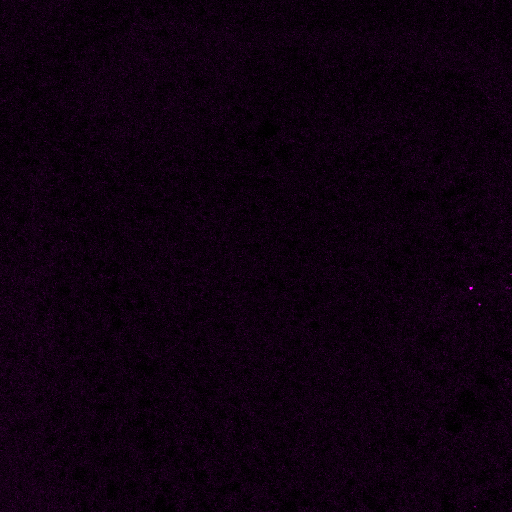

Supplement: Supplementary file 7 — Source Data for Figure 3 [file EMBJ-42-e110454-s007.zip › Fig3/FigG/Atg2 RNAi-tunel.tif]

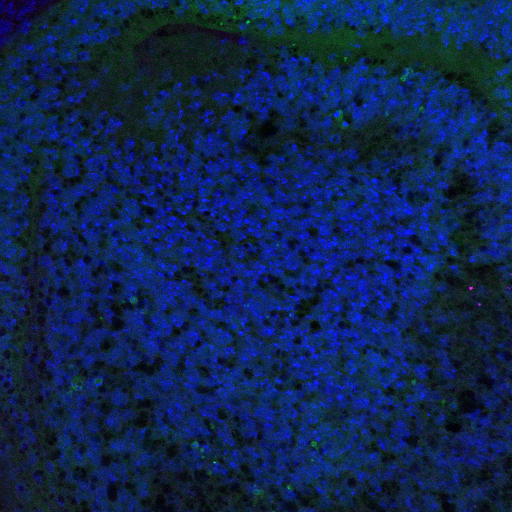

Supplement: Supplementary file 7 — Source Data for Figure 3 [file EMBJ-42-e110454-s007.zip › Fig3/FigG/Atg2 RNAi-merge.tif]

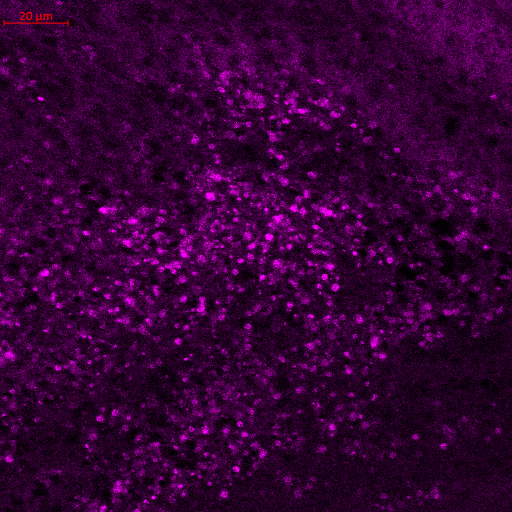

Supplement: Supplementary file 7 — Source Data for Figure 3 [file EMBJ-42-e110454-s007.zip › Fig3/FigG/mCherry RNAi-Tunel.tif]

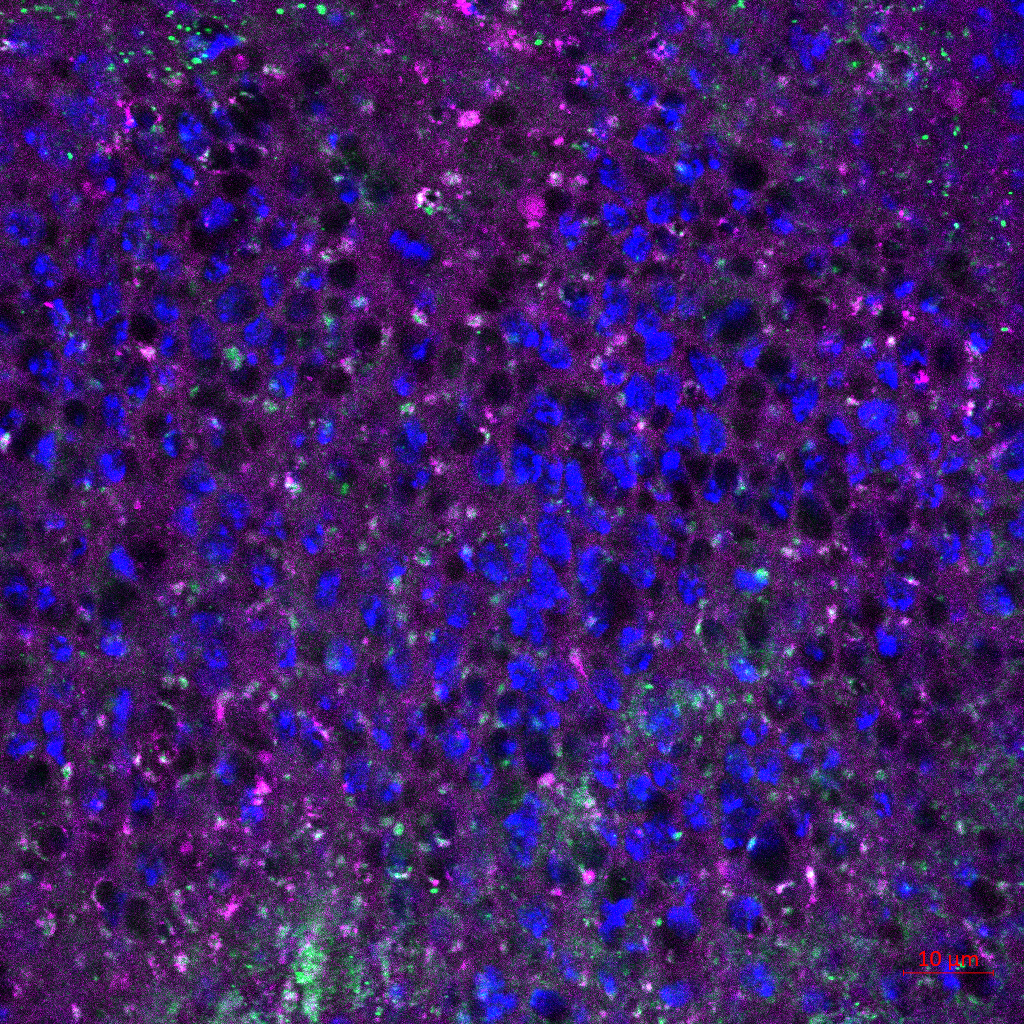

Supplement: Supplementary file 7 — Source Data for Figure 3 [file EMBJ-42-e110454-s007.zip › Fig3/FigC/Wing disc/merge.tif]

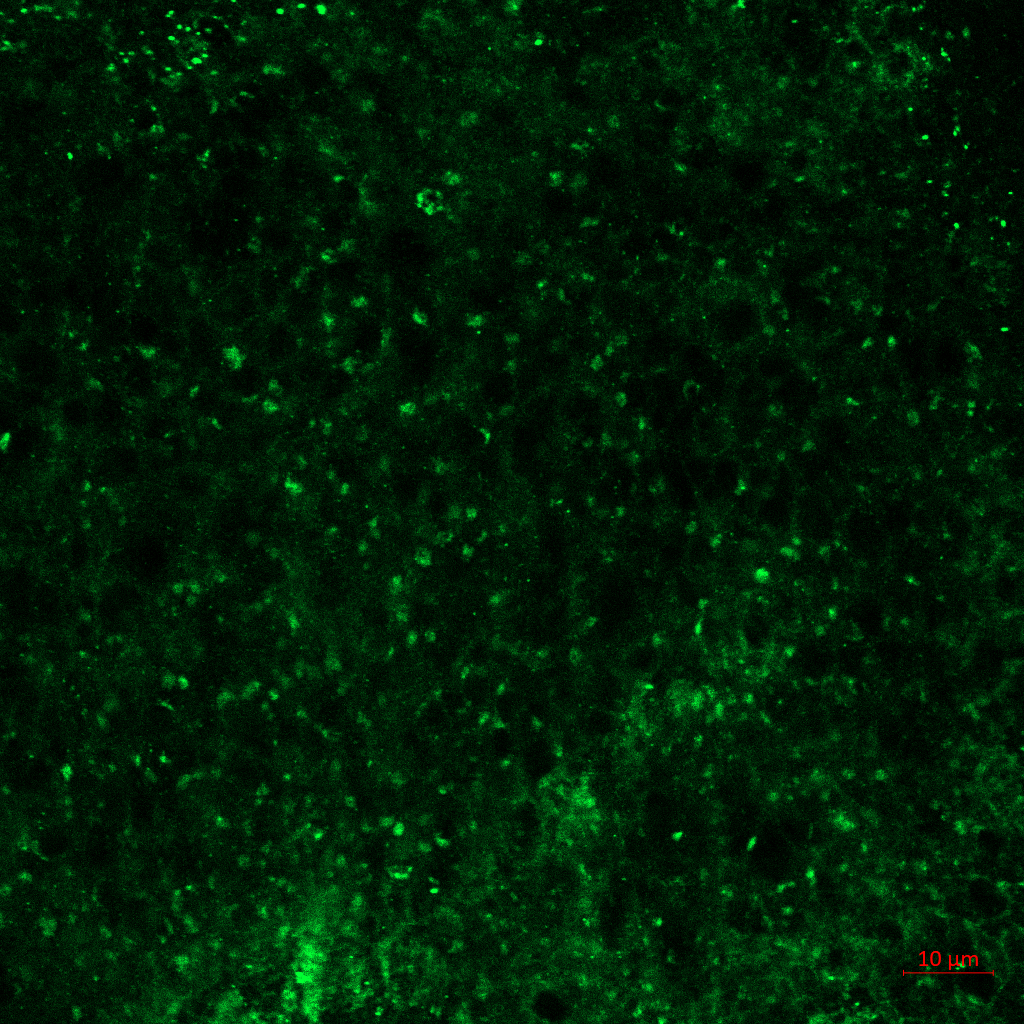

Supplement: Supplementary file 7 — Source Data for Figure 3 [file EMBJ-42-e110454-s007.zip › Fig3/FigC/Wing disc/synr HA.tif]

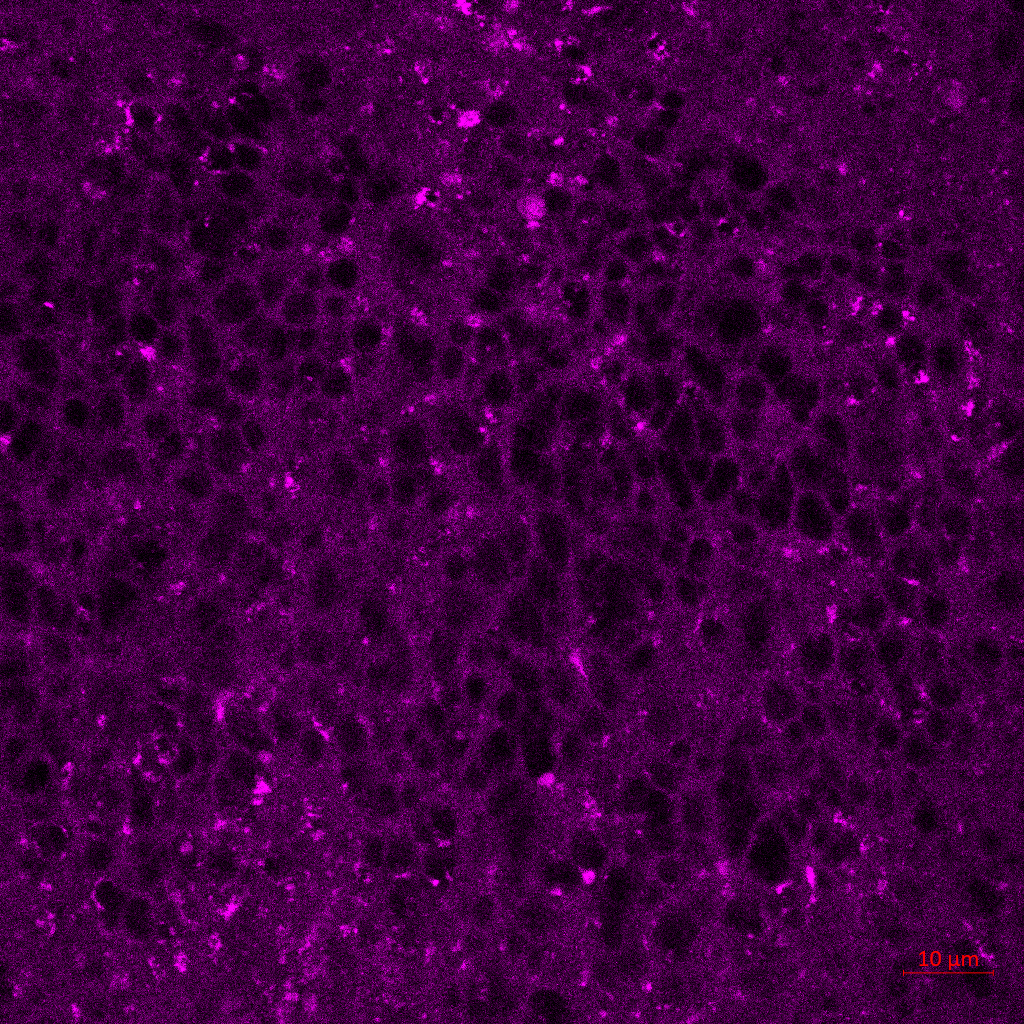

Supplement: Supplementary file 7 — Source Data for Figure 3 [file EMBJ-42-e110454-s007.zip › Fig3/FigC/Wing disc/mCherry atg8a.tif]

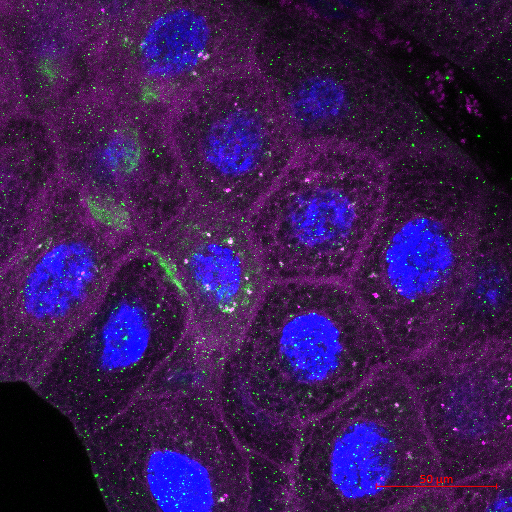

Supplement: Supplementary file 7 — Source Data for Figure 3 [file EMBJ-42-e110454-s007.zip › Fig3/FigC/salivary gland/merge.tif]

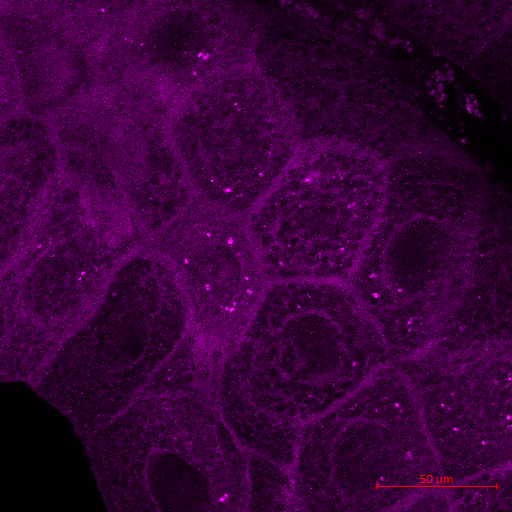

Supplement: Supplementary file 7 — Source Data for Figure 3 [file EMBJ-42-e110454-s007.zip › Fig3/FigC/salivary gland/mCherry atg8a.tif]

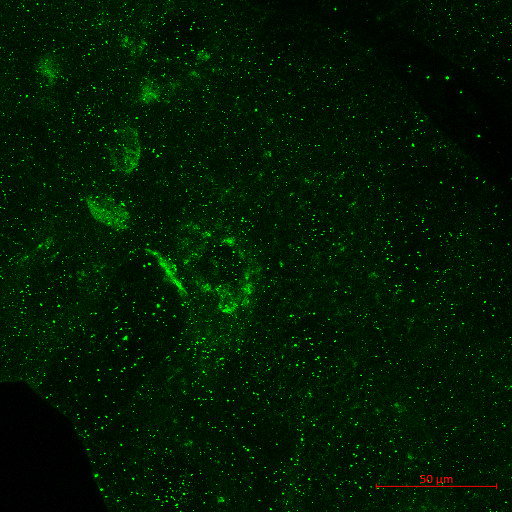

Supplement: Supplementary file 7 — Source Data for Figure 3 [file EMBJ-42-e110454-s007.zip › Fig3/FigC/salivary gland/synr HA.tif]

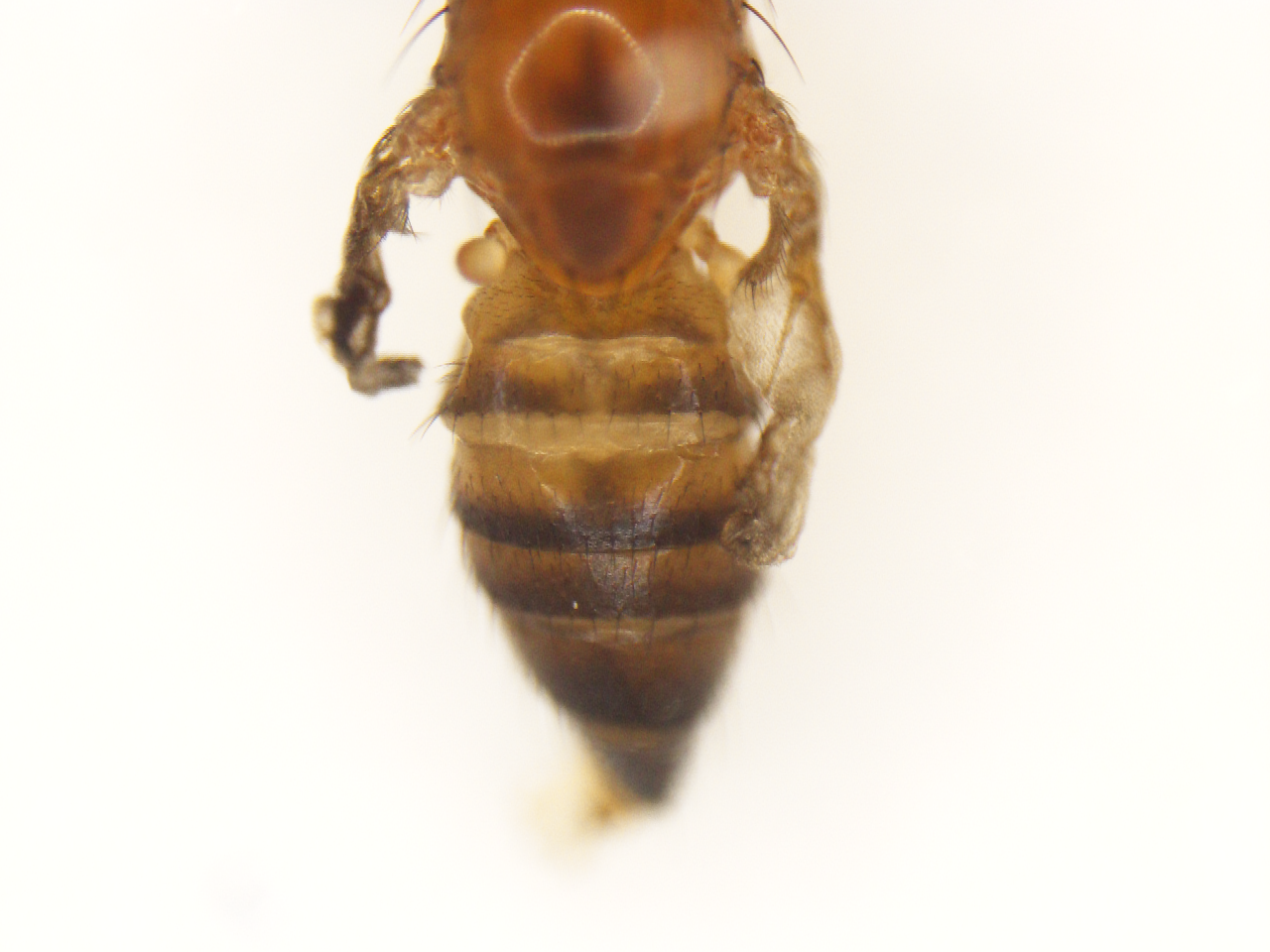

Supplement: Supplementary file 8 — Source Data for Figure 4 [file EMBJ-42-e110454-s002.zip › Fig4/Fig4A/+.png]

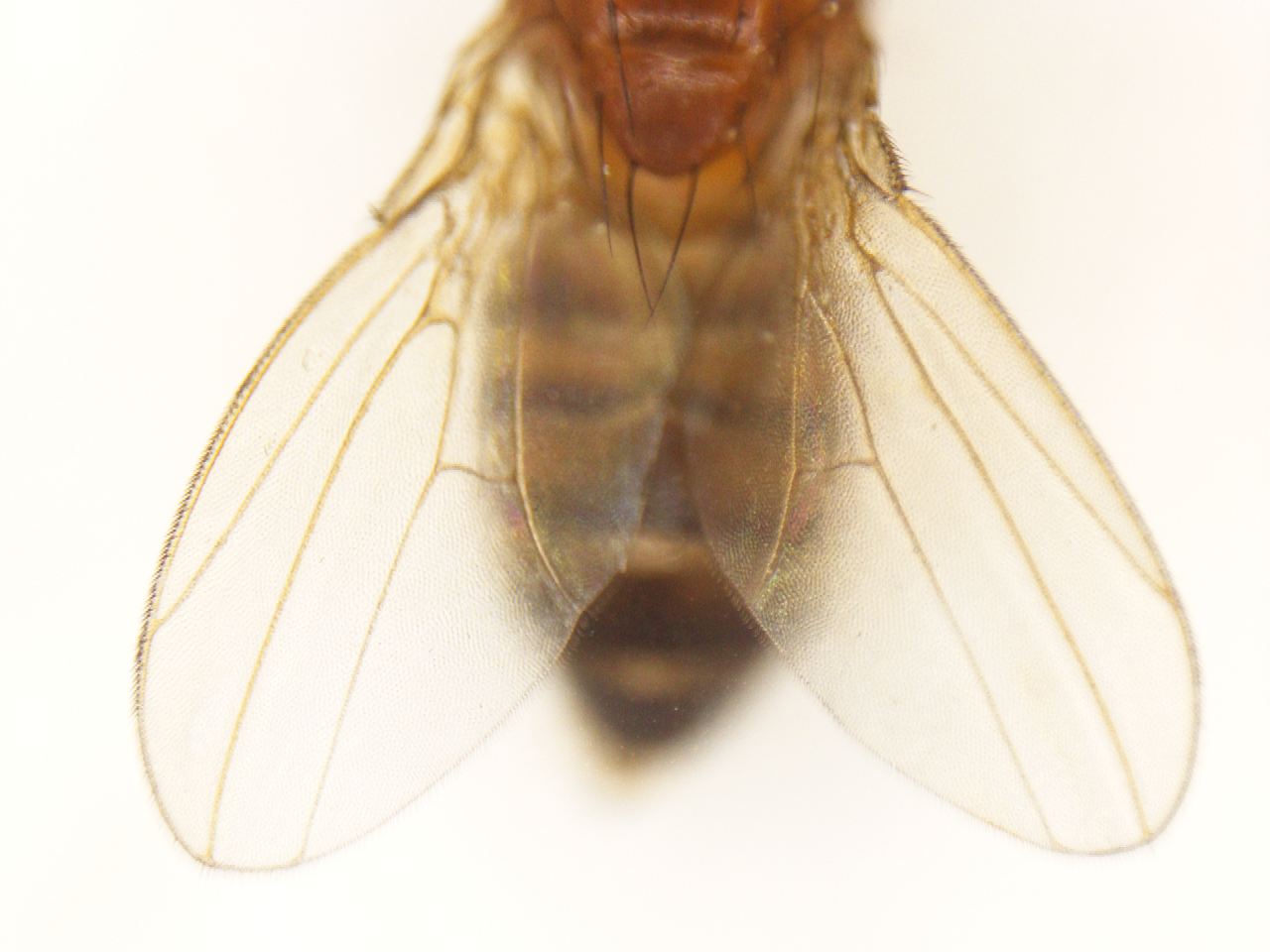

Supplement: Supplementary file 8 — Source Data for Figure 4 [file EMBJ-42-e110454-s002.zip › Fig4/Fig4A/synr RNAi.png]
