# Supplementary material for: Evidence for existence of an apoptosis‐inducing BH3‐only protein, sayonara, in Drosophila
Source: EMBO J. 2023 Feb 2;42(8):e110454. doi: 10.15252/embj.2021110454 (PMC10107002; doi:10.15252/embj.2021110454)
Supplement: Supplementary file 9 [file EMBJ-42-e110454-s009.pdf]

THE EMBO JOURNAL

Evidence for existence of an apoptosis-inducing BH3-only protein, sayonara, in Drosophila

Yuko Ikegawa, Christophe Combet, Mathieu Groussin, Vincent Navratil, Sabrina Safar-Remali, Takuya Shiota, Abdel Aouacheria, and Sa Kan Yoo
DOI: 10.15252/embj.2021110454

Corresponding author(s): Sa Kan Yoo (sakan.yoo@riken.jp)

Review Timeline:

Editorial Decision:	28th Apr 22
Revision Received:	22nd Nov 22
Editorial Decision:	22nd Dec 22
Revision Received:	27th Dec 22
Accepted:	28th Dec 22

Editor: Karin Dumstrei

Transaction Report:

(Note: With the exception of the correction of typographical or spelling errors that could be a source of ambiguity, letters and reports are not edited. Depending on transfer agreements, referee reports obtained elsewhere may or may not be included in this compilation. Referee reports are anonymous unless the Referee chooses to sign their reports.)

1st - Editorial Decision 28th Apr 2022

Dear Sakan,

Thanks for submitting your manuscript to The EMBO Journal. Your study has now been seen by three good experts in the field and their comments are provided below.

As you can see the referees appreciate the analysis and find the discovery of a potential BH3 only protein in Drosophila very interesting. However, as you can see they also raise significant concerns with it that would have to be resolved in order to consider publication here. Should you be able to extend the analysis along the lines suggested by the referees then I would like to consider a revised version.

I think it would be helpful to discuss the raised points further and I am available to do so via email or video.

When preparing your letter of response to the referees' comments, please bear in mind that this will form part of the Review Process File, and will therefore be available online to the community. For more details on our Transparent Editorial Process, please visit our website: https://www.embopress.org/page/journal/14602075/authorguide#transparentprocess

Thank you for the opportunity to consider your work for publication. I look forward to discussing your revisions further

with best wishes

Karin

Karin Dumstrei, PhD
Senior Editor
The EMBO Journal

Instructions for preparing your revised manuscript:

Guide For Authors: https://www.embopress.org/page/journal/14602075/authorguide

I have attached a PDF with helpful tips on how to prepare the revised version.

We realise that it is difficult to revise to a specific deadline. In the interest of protecting the conceptual advance provided by the work, we recommend a revision within 3 months (27th Jul 2022). I can grant an extension if needed and please discuss with me if you need more time.

As a matter of policy, competing manuscripts published during this period will not negatively impact on our assessment of the conceptual advance presented by your study. However, we request that you contact the editor as soon as possible upon publication of any related work, to discuss how to proceed.

Use the link below to submit your revision:

https://emboj.msubmit.net/cgi-bin/main.plex

Referee #1:

Ikegawa et al report the discovery of a potential BH3 only protein in Drosophila. BH3 proteins are induced or activated by stress and interact with pro- and anti- apoptotic multidomain Bcl2 family members to activate apoptosis. Here the authors show that the newly identified Sayonara (Synr) protein can induce cell death in a BH3 dependent manner when overexpressed, and the overexpressed protein can bind to co-expressed multidomain Bcl2 family members Buffy and Debcl. Furthermore, knockdown of synr inhibits stress induced cell death activated by p53 overexpression, and in response to starvation. The authors also demonstrate an interesting relationship between apoptosis and autophagy in response to Synr expression. The absence of a pro-apoptotic BH3 only protein has confused the understanding of how Bcl2 family members contribute to cell death regulation in Drosophila, supporting the value of this work. However, there are a number of issues that decrease enthusiasm.
The most major issue is the mechanism of action of Synr. The domain they identify as a BH3 domain fits with the consensus

BH3 domain structure, and is well conserved between Drosophila species and within the Diptera. This small domain is embedded between the coiled-coil domains of a previously uncharacterized protein. Overexpression of Synr induced caspase activation and tissue loss in the developing wing. Deletion of the BH3 domain completely inhibits caspase activation and tissue loss. Unfortunately, there is no way to judge whether a stable protein is made at all after this domain deletion. The use of a tagged protein would allow the presence and levels of the overexpressed proteins to be assessed. Even more problematic is the analysis of domains required for interactions with the multidomain Bcl2 family members Buffy and Debcl. The canonical mechanism of action of the BH3 proteins is an interaction of the BH3 domain with the surface groove of the multidomain Bcl2 family members. The authors show that knockdown of either Buffy or Debcl inhibits Synr induced tissue loss, indicating a strong genetic interaction. However, deletion of the BH3 domain alone blocks cell death but does not block the detected physical interactions between Synr and Buffy or Debcl. Thus, it is not clear how these interactions, or how the BH3 domain, contribute to cell death.

Cell death induced by overexpressed Synr shows characteristics of both autophagy (punctate Atg8-mCherry) and apoptosis (effector caspase activation). Overexpression of both Synr and the classical apoptosis inducer Reaper result in increased lysotracker staining, indicating acidification (most likely lysosomes), and increased Atg8-mCherry puncta indicating autophagosomes. Caspase and Atg (autophagy) gene knockdown inhibits tissue loss in both cases, suggesting a previously undescribed role for Atg genes in caspase activation. An irrelevant UAS-RNAi would be an important control for all interaction experiments, to account for dilution of the Gal4.

The use of a better control also applies to the nub>p53 experiment shown in Figure 4A, showing that synr knockdown inhibits stress activated cell death. In addition, since this is a unique synr RNAi, it would be best to show that a second line has the same phenotype.

In sum, this work has some problems, however the discovery of a fly BH3 protein would be of significant interest to the cell death community, as it helps to resolve the conserved role and mechanism of action of the Bcl2 family across evolution.

More minor issues:
Page 6 dogma is more appropriate than "dogmatic idea"
Page 7 The caspase sensor GC3A1 needs a reference
Figure S1-it would be helpful to provide a key to the homology score
Figure 2A-D-the wing defects should be quantified to allow significance and variability to be assessed
Figure 2E- the cleaved caspase staining is of poor quality, and the decreased signal is hard to judge
Figure 2N-The detection of both tagged Buffy and Tagged Debcl to GST-Snyr does not mean they bind simultaneously in one complex. A sequential IP would be necessary to show this.
Figure 3C-the colocalization of overexpressed Synr and ATG8-mCherry does not infer a functional relationship.
Figure 3D-E- the wing size should be quantified.
Figure 4E- the graph lacks error bars
Figure S3E-the graph lacks error bars
Figure S4C-please show interactions with the Synr BH3 mutant

Referee #2:

In this paper, the authors describe the identification of a BH3-only protein in Drosophila. These proteins are key regulators of Bcl-2 family proteins during the intrinsic (mitochondrial) pathway of apoptosis. Two proteins of the Bcl-2 family had been identified so far (Buffy and Debcl) in Drosophila but nothing was known concerning a potential BH3-only protein. By an in silico analysis of the database using BLAST searches on UniProt, the authors identified a gene, CG14044 (Q9VMY2), whose product contains a region that meets the definition of the BH3 motif. This gene was named sayonara (synr). Its product, Synr induces caspase activation in a Synr-BH3 motif-dependent manner and interacts genetically and biochemically with Buffy and Debcl. Their results also suggest the existence of a feedback loop between Synr-mediated caspase activation and autophagy. This is a topic of great interest. Indeed, as BH3-only proteins are crucial in the control of apoptosis, at least in mammals and nematodes, the discovery of a Drosophila BH3-only protein is determinant to our understanding of the evolution of the apoptotic machinery. This discovery will help characterize the activities of Bcl-2 proteins. However, I have several concerns about the content and the form of this paper.

Major points:
First, although the authors show that synr over-expression induced caspase activation, they do not clearly demonstrate that Synr induces apoptosis (see points 3, 5).
Second, the authors claim that Synr binds Buffy and Debcl simultaneously, which is not clearly demonstrated and no results are presented in Drosophila (see points 14).
Furthermore, some additional controls are needed (see points 9, 12 and 17).
1- P2 Fig1D: could the authors clarify whether lethality observed under Actin-Gal4 driver occurs at a specific (larval, pupal) or several developmental stages?
2- P2 Fig1E: Gal4 system is temperature-sensitive. When using Nub-Gal4 driver, did the author verify that the strength of wing phenotypes and lethality was varying according to the temperature?
3- The experiments in figure 1E show that overexpression of synr induces caspase activity and abnormal wings. Some caspases functions are not related to apoptosis. Therefore, to conclude that Synr induces apoptosis, it would be necessary to identify that it induces cell death, for example using TUNEL labelling.

4- What does the GFP fluorescence in figure 1E correspond to? What does the dotted line delimit? What does the blue color correspond to in the merge? Could these points be explained in the main text or caption?
5- Figure 1G: Nub>Synr WT wings seem to present a defect in the fusion of the ventral and dorsal epithelial layer, which seems to make wing size difficult to estimate. Furthermore, this kind of phenotype may result from other events than apoptosis induction. Can the author discuss this point and show representative wing phenotype of Nub>SynrΔBH3?
6- Fig1H: the authors use the GC3AI system to quantify caspase activation. Could the authors explain how it works? Are the pictures representative? The labelling is weak and spreads in the whole wing pouch in figure 1H, whereas, using activated-Dcp1 antibodies it is stronger and more centered in the central part of the wing pouch around the dorso-ventral boundary in figure 1E for the same genotype. Could the authors discuss these differences? Could they explain in the materials and methods section how the percent area is calculated when using GC3AI?
7- Fig1K: Since transgene expression depends on the insertion site, are all the different forms of synr inserted at the same site in the Drosophila genome? Are they expressed at the same level? The author should clarify this point.
8- Figure 1K compare wing phenotypes induced by the different forms. To conclude that there is no differences between each form, at least 30 wings in each case seem necessary as in control.
9- Fig2A-D: can genetic suppression of the synr wing-induced phenotype by Buffy RNAi, Debcl RNAi or Dcp-1 RNAi, be seen in all flies at the same level? A UAS RNAi control is absolutely needed to exclude a phenotype suppression due to a problem of Gal4 titration? The valium genetic background can affect apoptosis level. Specific control strains such as UAS-RNAi luciferase are available in Bloomington should be used to perform control experiments. Another control experiment could be to study Synr-induced phenotype in a Debcl or Buffy mutant background.
10- Paragraph 5 of the results section: Concerning the pro-apoptotic activity of Buffy, it should be noted that it has been observed in some peculiar cases, notably in germ cells during spermatogenesis and oogenesis and during the neurodegeneration caused by expanded polyglutamine. Furthermore, in this latter context, Debcl appears anti-apoptotic (Senoo-Matsuda et al. 2005, EMBO J 24:2700-2713). In the wing disc, Debcl and Buffy have been shown to be respectively pro- and anti-apoptotic (Clavier et al. 2015, J Cell Sci 128: 3239-3249). The observation that inhibition of either Buffy, Debcl or Dcp-1 suppresses the adult wing phenotype merits to be discussed in this context.
11- Figure 2E: is caspase activation detected in Nub>synr, Debcl RNAi discs with an anti-activated Dcp1 as in figure 1E? This should be specified in the caption.
12- Similarly, the authors show that inhibition of Debcl suppresses Synr-mediated caspase activation in the wing disc. Does inhibition of Buffy also suppress Synr-mediated caspase activation in the wing disc? This experiment must be added in figure 2E.
13- Fig 2I, K, M: how many experiments have been done? Are there statistical analyses?
14- The fact that Synr can pull down Debcl and Buffy simultaneously does not show that they belong to the same complex. To conclude, the authors need to perform complementary experiments, such as competition experiments. Furthermore, these experiments have been done in mammalian cells which have several Bcl-2 and BH3-only proteins that could bind Debcl, Buffy and/or Synr. Replicating these experiments in Drosophila or using Drosophila extract would be preferable: for example, GST pull-down experiments using Drosophila extract from WT or Buffy mutant strain expressing Debcl-HA protein.
15- Fig 3B: What is the third column for?
16- Fig 3C and Fig S2, the authors look at salivary glands, but it is never explained in the result section. Could the authors clarify this point in the text. Is it the case in wing imaginal disc?
17- Fig 3D-E: same problem as in point 9 above.
18- Fig3F: why do the authors use propidium iodide? A TUNEL experiment would be more suitable.
19- Fig 3G and 3H what are "X" and "+" standing for? A control RNAi strain of the same genetic background as the other RNAi strains should be used as a control (see point 9).
20- Fig3 J-L: What does the GFP labelling correspond to? What is RFP O/E standing for? Is it used as a control?
21- The phenotypic suppression test with Reaper has been done with the vg-Gal4 driver whereas lysotracker and m-cherry Atg8 labelling have been done using nub-Gal4. Could the authors explain why they use different drivers? When induced by the vg-Gal4 driver, Reaper induces notched-wing phenotypes (Figure 3L(1)). Is it the case when Reaper is induced by the nub-Gal4 driver? Could the authors present a picture of the nub>Rpr wing phenotype?
22- I agree that Reaper-induced wing phenotypes may result from autophagy and apoptosis but I think the results presented in 3L do not prove that autophagy and apoptosis activate each other. The authors do not quantify apoptosis or autophagy in the flies in Figure 3L.
23- Did the authors study Reaper-induced apoptosis or phenotype in a synr RNAi context?
24- Fig4F is too speculative (see point 14)
25- It has been clearly reported that Buffy is able to physically interact with the Debcl and that these two proteins often act in an opposite way to control apoptosis. It can be hypothesized that Synr interacts only with Buffy through its BH3 domain and that the interaction with Debcl, that do not involve BH3 and cdd domains, is indirect and occurs through the Buffy protein. This hypothesis could explain the results of the phenotypic interaction tests between synr and buffy RNAi or synr and debcl RNAi, if those results are confirmed. This may be considered in the discussion section.

More general points
1- P2 "...cell death program, which is primarily regulated by the inhibitor of apoptosis (IAP) family of proteins..." should be changed in "...cell death program, which is primarily regulated by the RHG proteins that antagonize inhibitor of apoptosis (IAP) family of proteins..."
2- P2 "BH3-only proteins work as important stress sensors that initiate caspase activation" is ambiguous. BH3 proteins do not

directly activate caspases. "BH3-only proteins work as important stress sensors that initiate events leading to caspase activation" would be better.
3- P2 Similarly, "...BH3-only proteins work as important stress sensors that initiate caspase activation..." could be replaced by "...BH3-only proteins work as important stress sensors that initiate the caspase activation pathway".
4- The legends of the figures are sometimes incomplete. The staining used for each microscopy panel must be precise in the figure caption. Genotypes and statistical tests should be indicated in each figure caption.
5- The imaginal discs orientation (antero-posterior) has to be indicated.
6- The same problem appears recurrently in the text. "BH3-only protein that activates caspase" could be replaced by " BH3-only protein that initiates a caspase activation pathway. " in some parts of the text.
7- In fig 1E: "caspase", not "caspsae"

Referee #3:

Bcl-2 family proteins are critical regulators of caspases in vertebrates and C. elegans, with BH3-only proteins initiating a shift in balance of other Bcl-2 family proteins. However, BH3-only proteins had remained at large in Drosophila. Here, the authors identify a putative BH3-only Drosophila gene, Synr. They show that the encoded protein has signature BH3-only residues. They show evidence that Synr can induce apoptosis and/or autophagy, and this activity requires other Bcl-2 family proteins and caspases. They further show that Synr inhibition can suppress p53-induced phenotypes in Drosophila tissues.

Overall, this study is potentially very interesting. If the authors can support their claims with strong data, it would change the way we think about the Bcl-2 family function in Drosophila. However, there are a number of technical issues that fall short of convincingly supporting their claims. Below are a few specific points that the authors should try to address:

1. As the authors cite, BH3 motifs have very little sequence conservation aside from a few key residues. Because of such poor conservation (as shown in Figure 1A), sequence alignment of the putative BH3-motif does not necessarily make a compelling case. Perhaps with this in mind, the authors show that Synr can help activate caspases in vivo, and that deleting the entire BH3-domain abolishes this Synr function. Still, it will linger in the readers' minds that the putative BH3-motif has poor sequence conservation. It remains possible that this sequence has other functions unrelated to BH3-function, which could have been abolished by the deletion of the entire sequence. To quell such concerns, I suggest generating BH3 point mutants in which the "signature BH3 only residues" are substituted (instead of the entire deletion of the region). That experiment will support the idea that the "BH3-signature sequences" are functionally relevant.
2. In Figure 2, the authors show that nub>synr phenotype is suppressed by adding buffy RNAi in the background - which is potentially very interesting. But it is important to have a control RNAi line in Fig. 2A and E to have the same number of uas-elements in each condition (the authors do this in Figure 3L for rpr suppression experiments). Otherwise, it remains a possibility that the suppression of the nub>synr phenotype is due to the titration of Gal4.
3. Figure 2A-D shows representative images only. Some type of a quantification is needed.
4. The binding experiments in Figure 2H-N is interesting. But to highlight the importance of the putative BH3-motif, the authors should use BH3-point mutants (as suggested for point #1 above) that substitute signature BH3 sequences instead of deleting the entire BH3 region.
5. Figure 3 shows Synr's ability to induce autophagy. The authors should be aware that they are not the first to implicate Drosophila Bcl-2 family proteins in regulating autophagy. Citing previous papers reporting that buffy, debcl, dcp-1 regulates autophagy (e.g., PMID 19242106 and 18794330) would provide a helpful background regarding the Synr-autophagy link.
6. The authors show a number of RNAi experiments throughout the manuscript. Many of those appear to rely on single RNAi lines (indicated in Table S1), leaving open the possibility of off-target effects. The results need to be validated through independent means, such as the use of second RNAi lines (targeting a different region), or the utilization of other loss-of-function strategies.

We thank the editor and all the reviewers for their helpful comments. We addressed almost all comments by experimenting intensely more than half a year for this revision. For example, we newly generated six transgenic lines (three tagged sayonara strains, two amino acid substitution strains and one shRNA strain) to address the reviewers' comments. We also started a new collaboration with a biochemist, Takuya Shiota, who is an expert of UV-mediated cross linking of proteins to demonstrate the BH3 motif of Synr physically and directly binds to Bcl2 proteins Debcl/Buffy. We also re-took appropriate controls. Because of these new experiments that address all of the reviewers' insightful comments, now the manuscript improved tremendously. We hope this extensively revised manuscript will satisfy all reviewers. Following is a point-by-point response.

Referee #1:
Ikegawa et al report the discovery of a potential BH3 only protein in Drosophila. BH3 proteins are induced or activated by stress and interact with pro- and anti- apoptotic multidomain Bcl2 family members to activate apoptosis. Here the authors show that the newly identified Sayonara (Synr) protein can induce cell death in a BH3 dependent manner when overexpressed, and the overexpressed protein can bind to co-expressed multidomain Bcl2 family members Buffy and Debcl. Furthermore, knockdown of synr inhibits stress induced cell death activated by p53 overexpression, and in response to starvation. The authors also demonstrate an interesting relationship between apoptosis and autophagy in response to Synr expression. The absence of a pro-apoptotic BH3 only protein has confused the understanding of how Bcl2 family members contribute to cell death regulation in Drosophila, supporting the value of this work. However, there are a number of issues that decrease enthusiasm.

The most major issue is the mechanism of action of Synr. The domain they identify as a BH3 domain fits with the consensus BH3 domain structure, and is well conserved between Drosophila species and within the Diptera. This small domain is embedded between the coiled-coil domains of a previously uncharacterized protein. Overexpression of Synr induced caspase activation and tissue loss in the developing wing. Deletion of the BH3 domain completely inhibits caspase activation and tissue loss. Unfortunately, there is no way to judge whether a stable protein is made at all after this domain deletion. The use of a tagged protein would allow the presence and levels of the overexpressed proteins to be assessed.

To address this question, we newly performed DNA construction and generated three new transgenic lines with the HA tag: WT, BH3 deletion mutant and amino acid substitution. We observed comparable expression among WT, the deletion mutant and the amino acid change mutant. In fact, we consistently saw slightly more signals in the mutants than WT, likely due to efficient cell death in WT. At least, we never observed lower signals in the mutants. Now we added the data in Fig EV1F.

Even more problematic is the analysis of domains required for interactions with the multidomain Bcl2 family members Buffy and Debcl. The canonical mechanism of action of the BH3 proteins is an interaction of the BH3 domain with the surface groove of the multidomain Bcl2 family members. The authors show that knockdown

of either Buffy or Debcl inhibits Synr induced tissue loss, indicating a strong genetic interaction. However, deletion of the BH3 domain alone blocks cell death but does not block the detected physical interactions between Synr and Buffy or Debcl. Thus, it is not clear how these interactions, or how the BH3 domain, contribute to cell death.

As the reviewer rightly pointed out, we have demonstrated that the BH3-only protein sayonara genetically and biochemically interacts with Buffy/Debcl, and that BH3 deletion or amino acid substitution suppressed the sayonara phenotype in vivo. However, the BH3 mutant still maintains a gross interaction with Buffy/Debcl in a biochemical pulldown assay. Based on our data and literature, we still considered a possibility that BH3 of Sayonara may interact with Buffy/Debcl. To prove this in a definitive manner, we struck a new collaboration with Dr Shiota, an expert of photo-crosslinking by p-benzoyl-L-phenylalanine (BPA) (Chin et al, 2002; Shiota et al, 2011) to examine whether BH3 of Synr interacts with Buffy/Debcl.

We inserted BPA, phenylalanine analog to the BH3 motif of Sayonara by transforming Sayonara with an amber codon at/near the BH3, amber suppressor tRNA and its cognate aminoacyl-tRNA synthetase specific for BPA (Chin et al., 2002). Covalent cross linking at BPA occurs if two proteins are physically interacting (or, exist within 5 Å). This enables detection of direct physical interaction of two proteins. Using this method, we demonstrate that Sayonara's BH3 interacts with Buffy/Debcl (Fig 2N-P). We emphasize that the purpose of the traditional biochemical assay based on pulldown is to prove proteins interact each other at that mutated/deleted region. One problem of this traditional approach is if there is an additional region within the protein that mediates protein interaction, the traditional biochemical pulldown cannot detect interaction at the mutated region. The BPA assay can assess direct interaction of two proteins in a definitive manner.

Our data with photocrosslinking indicate that, although sayonara physically interact with Buffy/Debcl without BH3, there is still a physical and direct interaction between BH3 and Buffy/Debcl. Based on this, we discussed a possibility that this interaction may regulate the complex function at the discussion section.

We'd also like to emphasize a single study like ours cannot prove all biochemical aspects of protein interactions. We view the main, most important message of our study is finding of the BH3-only protein in flies, which has been missing for the last few decades. We admit that, although we demonstrated that BH3 of Sayonara interacts with Buffy/Debcl and that it is important in vivo, we still do not know how exactly Sayonara/Buffy/Debcl work at a molecular level. To reveal how the three proteins work together, we put tremendous effort to try to elucidate the complex structure based on extensive modeling analyses. But, even in the era of alphafold 2 and in spite of our intense efforts, modeling of the complex hasn't been successful so far. To elucidate how a protein complex works is a significant feat itself. For example, although we started to understand functions of channelrhodopsin or CRISPR-Cas9 a decade ago, how exactly they work only started to emerge after detailed protein structure analyses by several labs (Jiang & Doudna, 2017; Kishi et al, 2022). To our view, even how mammalian BH3-only proteins exactly work (direct

activation, indirect activation, or priming-capture displacement model) has not been completely solved a few decades after their initial findings. We believe it takes tremendous time and effort to elucidate how Synr/Buffy/Debcl work together, and it is definitely worthwhile for a future endeavor. We believe that a critical study not only provides answers but also opens up a new avenue of research by providing several questions to pursue in the future. We hope we'll be able to find a molecular mechanism of the Synr/Buffy/Debcl in the coming future. We clarified this point in discussion.

Cell death induced by overexpressed Synr shows characteristics of both autophagy (punctate Atg8-mCherry) and apoptosis (effector caspase activation). Overexpression of both Synr and the classical apoptosis inducer Reaper result in increased lysotracker staining, indicating acidification (most likely lysosomes), and increased Atg8-mCherry puncta indicating autophagosomes. Caspase and Atg (autophagy) gene knockdown inhibits tissue loss in both cases, suggesting a previously undescribed role for Atg genes in caspase activation. An irrelevant UAS-RNAi would be an important control for all interaction experiments, to account for dilution of the Gal4.

We added irrelevant UAS-RNAis as controls (Fig 2A, 2E, 2G, 3D, 3G, 3J, EV2B-C, EV2G-H, EV3B). In case we cannot re-do evrything, we confirmed that UAS-RNAi expression does not affect the phenotype compard to w- control.

The use of a better control also applies to the nub>p53 experiment shown in Figure 4A, showing that synr knockdown inhibits stress activated cell death. In addition, since this is a unique synr RNAi, it would be best to show that a second line has the same phenotype.

To address this question, we newly generated an additional shRNA for synr and quantified GC3Ai signals together with mCherry RNAi and the previous shRNA (Fig EV3B)

In sum, this work has some problems, however the discovery of a fly BH3 protein would be of significant interest to the cell death community, as it helps to resolve the conserved role and mechanism of action of the Bcl2 family across evolution.

We thank this reviewer for his/her potential enthusiasm on our paper.

More minor issues:
Page 6 dogma is more appropriate than "dogmatic idea"

We revised the text.

Page 7 The caspase sensor GC3A1 needs a reference

We inserted the GC3Ai reference (Schott *et al*, 2017) in the revised text.

Figure S1-it would be helpful to provide a key to the homology score

We added the following text in the revised legend.

“An * (asterisk) indicates positions which have a single, perfectly conserved residue. A : (colon) indicates conservation between groups of strongly similar properties. A . (period) indicates conservation between groups of weakly similar properties.”

Figure 2A-D-the wing defects should be quantified to allow significance and variability to be assessed

We re-did all the experiments taking proper controls and quantified the wing size as the reviewer suggested (Fig 2A-E).

Figure 2E- the cleaved caspase staining is of poor quality, and the decreased signal is hard to judge

Since cDCP1 staining consistently gives us noisy signals, we included new data with GC3Ai with proper controls in this revised report (Fig 2G, EV2H).

Figure 2N-The detection of both tagged Buffy and Tagged Debcl to GST-Snyr does not mean they bind simultaneously in one complex. A sequential IP would be necessary to show this.

We thank this reviewer for this extremely helpful comment. As the reviewer helpfully suggested, we performed a sequential IP. We first pulled down flag-Buffy, eluted the protein complex with the flag peptide and did the second pulldown for myc-Synr. We confirmed existence of HA-Debcl in the pulled down sample, strongly indicating that there is a Synr/Debcl/Buffy complex (Fig 2R). Please note that, due to the heavy chain of the antibody used for IP, Myc-Synr couldn’t be visualized so cleanly (If we try to do so, the heavy chain will mask the Myc-Synr signals).

Figure 3C-the colocalization of overexpressed Synr and ATG8-mCherry does not infer a functional relationship.

We agree with the reviewer. We took a great care not to imply any functional causality based on this colocalization data. That is why we pursued mechanistic analyses after this data. In this revised manuscript, we also added data with the wing disc in addition to the original salivary gland data (Fig 3C, EV2A). Again, we made sure that this colocalization does not guarantee a functional causality.

Figure 3D-E- the wing size should be quantified.

We re-did the experiment and quantified all the data with proper controls (Fig 3D-F, EV2B-C).

Figure 4E- the graph lacks error bars
Figure S3E-the graph lacks error bars

This is a log rank test, which does not accommodate error bars.

Figure S4C-please show interactions with the Synr BH3 mutant

To address the reviewer's sprit, we executed this experiment. dBorg0 binds to Synr BH3 mutant (Fig EV4 C-F), which indicates that dBorg0 is similar to Buffy/Debcl.

Referee #2:

In this paper, the authors describe the identification of a BH3-only protein in Drosophila. These proteins are key regulators of Bcl-2 family proteins during the intrinsic (mitochondrial) pathway of apoptosis. Two proteins of the Bcl-2 family had been identified so far (Buffy and Debcl) in Drosophila but nothing was known concerning a potential BH3-only protein. By an in silico analysis of the database using BLAST searches on UniProt, the authors identified a gene, CG14044 (Q9VMY2), whose product contains a region that meets the definition of the BH3 motif. This gene was named sayonara (synr). Its product, Synr induces caspase activation in a Synr-BH3 motif-dependent manner and interacts genetically and biochemically with Buffy and Debcl. Their results also suggest the existence of a feedback loop between Synr-mediated caspase activation and autophagy. This is a topic of great interest. Indeed, as BH3-only proteins are crucial in the control of apoptosis, at least in mammals and nematodes, the discovery of a Drosophila BH3-only protein is determinant to our understanding of the evolution of the apoptotic machinery. This discovery will help characterize the activities of Bcl-2 proteins. However, I have several concerns about the content and the form of this paper.

We thank this reviewer for giving us many feedbacks. We put a serious, tremendous effort to address all his/her comments experimentally.

Major points:
First, although the authors show that synr over-expression induced caspase activation, they do not clearly demonstrate that Synr induces apoptosis (see points 3, 5).

In addition to the original PI data (Now Fig EV2D), we included TUNEL data in the revised paper (Fig 1E, 3G).

Second, the authors claim that Synr binds Buffy and Debcl simultaneously, which is not clearly demonstrated and no results are presented in Drosophila (see points 14).

This was speculation based on biochemical and genetic data in the original manuscript. The reviewer 1 motivated us to perform the sequential pulldown experiment (please see our response to the reviewer 1). Now we demonstrate Synr binds to Buffy and Debcl likely simultaneously in the revised report (Fig 2R). We also clarify that we are using purified Drosophila proteins from either E.coli or culture human cells. Additionally, in this revised manuscript, we demonstrate direct interaction of Synr and Buffy/Debcl using photo-crosslinking based on BPA (Fig 2N-P).

Furthermore, some additional controls are needed (see points 9, 12 and 17).

We added careful control data in this revised report (Fig 2A, 2E, 2G, 3D, 3G, 3J, EV2B-C, EV2G-H, EV3B).

1- P2 Fig1D: could the authors clarify whether lethality observed under Actin-Gal4 driver occurs at a specific (larval, pupal) or several developmental stages?

Animals die during the larval stage. We clarified this point in text.

2- P2 Fig1E: Gal4 system is temperature-sensitive. When using Nub-Gal4 driver, did the author verify that the strength of wing phenotypes and lethality was varying according to the temperature?

We acknowledge that the phenotype with the nub-gal4 is weaker at 18C and stronger at 30C than 25C. 30C is metabolically stressful for flies and 18C is often too weak to drive gene/RNAi expression. Thus, throughout this paper, we used 25 C to express transgenes and RNAis as we previously did (Ciesielski *et al*, 2022; Nishida *et al*, 2021; Sasaki *et al*, 2021; Yoo *et al*, 2016), unless otherwise stated. We clarified this point in the method.

3- The experiments in figure 1E show that overexpression of synr induces caspase activity and abnormal wings. Some caspases functions are not related to apoptosis. Therefore, to conclude that Synr induces apoptosis, it would be necessary to identify that it induces cell death, for example using TUNEL labelling.

We did PI staining in the original manuscript to show that cells are dying, due to secondary necrosis that follows apoptosis (now shown in Fig EV2D). As requested by this reviewer, we added TUNEL labeling here (Fig 1E).

4- What does the GFP fluorescence in figure 1E correspond to? What does the dotted line delimit? What does the blue color correspond to in the merge? Could these points be explained in the main text or caption?

Nubbin-gal4/UAS-GFP was used to promote expression in the wing pouch. This was clarified in the legend.

5- Figure 1G: Nub>Synr WT wings seem to present a defect in the fusion of the ventral and dorsal epithelial layer, which seems to make wing size difficult to estimate. Furthermore, this kind of phenotype may result from other events than apoptosis induction. Can the author discuss this point and show representative wing phenotype of Nub>SynrΔBH3?

This is a good point. In this study we are using two Synr WT stocks. One is from flyORF. The second is the one we made in parallel with other synr mutants using the same insertion site. This was necessary to compare Synr WT and mutants on the identical condition. The flyORF sayonara is stronger than the sayonara WT we

ourselves created. With the one we created, the wing morphology is quantifiable (Fig EV1D). We clarified this in the revised text. To correlate the adult wing phenotype and the wing disc caspase activation, we are also showing caspase activation data (Fig 1E, 1H-I, Fig 2G, Fig 3H-I, Fig EV2H).

6- Fig1H: the authors use the GC3AI system to quantify caspase activation. Could the authors explain how it works? Are the pictures representative? The labelling is weak and spreads in the whole wing pouch in figure 1H, whereas, using activated-Dcp1 antibodies it is stronger and more centered in the central part of the wing pouch around the dorso-ventral boundary in figure 1E for the same genotype. Could the authors discuss these differences? Could they explain in the materials and methods section how the percent area is calculated when using GC3AI?

In our hands, cDCP1 staining gives us consistently variable and noisy signals. Based on conversation with other fly pushers, it seems this is a common observation among researchers. We found that GC3AI gives us clearer and quantifiable signals. We cited an appropriate paper for GC3AI (Schott *et al*, 2017) and quantified GC3Ai signals in the revised paper (Fig 1I, EV2H, 3I, EV3B). How the quantification was done was also explained in methods.

7- Fig1K: Since transgene expression depends on the insertion site, are all the different forms of synr inserted at the same site in the Drosophila genome? Are they expressed at the same level? The author should clarify this point.

We use the same insertion site attp2. We also addressed whether mutations affect expression of Sayonara by making new three transgenic lines with HA tag (see the response to the reviewer 1).

8- Figure 1K compare wing phenotypes induced by the different forms. To conclude that there is no differences between each form, at least 30 wings in each case seem necessary as in control.

We determined sample sizes empirically based on the observed effects. Regarding the assessment of the wing size, which has a relatively small distribution, we determined the minimum number is 10-20 based on our observation. But, for many analyses, more than 10 samples were analyzed in an extensive manner. We clarified that “Sample sizes were determined empirically based on the observed effects” in the method section.

9- Fig2A-D: can genetic suppression of the synr wing-induced phenotype by Buffy RNAi, Debcl RNAi or Dcp-1 RNAi, be seen in all flies at the same level? A UAS RNAi control is absolutely needed to exclude a phenotype suppression due to a problem of Gal4 titration? The valium genetic background can affect apoptosis level. Specific control strains such as UAS-RNAi luciferase are available in Bloomington should be used to perform control experiments. Another control experiment could be to study Synr-induced phenotype in a Debcl or Buffy mutant background.

We used a variety of additional controls in this revised manuscript. Please see our response to the reviewer 1. Although this information was not included in the manuscript, we‘ve been performing an RNAi screening to identify Synr’s downstream. We only got a few hits among hundreds of stocks we used. In general, to reverse the apoptosis phenotype is very difficult compared to inducing cell death by non-specific toxicity of RNAis. Based on this experience, we think the titration effect is minimum, and that is also why we didn’t include irrelevant control RNAis in the original manuscript.

10- Paragraph 5 of the results section: Concerning the pro-apoptotic activity of Buffy, it should be noted that it has been observed in some peculiar cases, notably in germ cells during spermatogenesis and oogenesis and during the neurodegeneration caused by expanded polyglutamine. Furthermore, in this latter context, Debcl appears anti-apoptotic (Senoo-Matsuda et al. 2005, EMBO J 24:2700-2713). In the wing disc, Debcl and Buffy have been shown to be respectively pro- and anti-apoptotic (Clavier et al. 2015, J Cell Sci 128: 3239-3249). The observation that inhibition of either Buffy, Debcl or Dcp-1 suppresses the adult wing phenotype merits to be discussed in this context.

We thank this reviewer for this helpful information. We apologize we failed to cite sufficient numbers of previous literature in the original text. As the reviewer mentioned, Debcl and Buddy can function as both pro and anti-apoptosis depending on contexts. Sharad Kumar also showed Buffy induces cell death in cultured Drosophila SL2 cells (Doumanis *et al*, 2007). We clarified this point and added appropriate, important literature (such as Clavier et al. 2015) in the revised text.

11- Figure 2E: is caspase activation detected in Nub>synr, Debcl RNAi discs with an anti-activated Dcp1 as in figure 1E? This should be specified in the caption.

Yes, that was cDCP1. In our hands cDCP1 tends to be more variable depending on replications, so in this revised manuscript, we also used GC3Ai extensively.

12- Similarly, the authors show that inhibition of Debcl suppresses Synr-mediated caspase activation in the wing disc. Does inhibition of Buffy also suppress Synr-mediated caspase activation in the wing disc? This experiment must be added in figure 2E.

Since GC3Ai is more quantitative and more consistent, we added data of Buffy using GC3Ai (Fig 2G, quantification in Fig EV2H).

13- Fig 2I, K, M: how many experiments have been done? Are there statistical analyses?

Each dot represents one replication: two independent replates in 2I: three replicates in 2K and three replicates in 2M in the original manuscript.

There is a change of the story here, so let us explain a little bit details.

In the original manuscript, in Fig 2I, the effect of delta ccd looked strong based on two independent experiments (original Fig 2 H-I). So, originally we concluded that deletion of the coiled-coil domain suppresses binding of Synr to Buffy. But, this reviewer's comment prompted us to increase the number of replication of GST-Synr and Buffy binding. And to our surprise, this time delta ccd Synr interacted with Buffy (please see the data below). So, we performed three more independent experiments, in total 6 replications. In the end, we had to change our conclusion to the one that deletion of the coiled-coil does not reduce binding of Sayonara and Buffy. That is, both Buffy and Debcl can bind to Sayonara without ccd. This does not change the overall story of this paper, but we are really relieved to have noticed this before publication. We thank this reviewer for his/her careful comment that prompted us to obtain more replications.

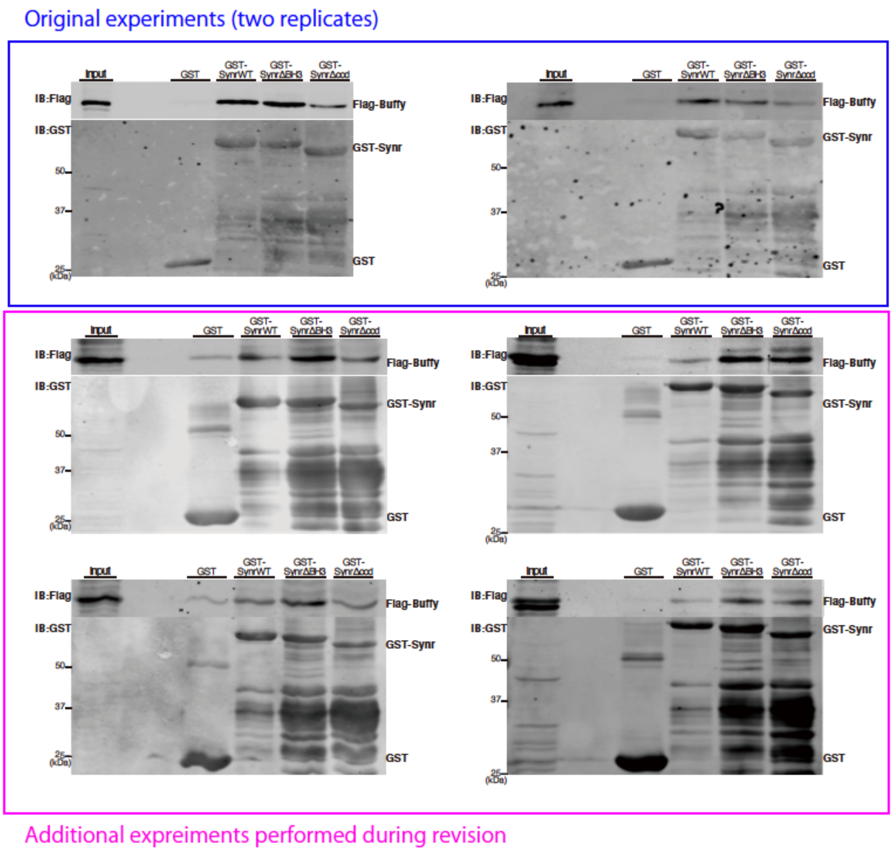

14- The fact that Synr can pull down Debcl and Buffy simultaneously does not show that they belong to the same complex. To conclude, the authors need to perform complementary experiments, such as competition experiments. Furthermore, these experiments have been done in mammalian cells which have several Bcl-2 and BH3-only proteins that could bind Debcl, Buffy and/or Synr. Replicating these experiments in Drosophila or using Drososphila extract would be preferable: for example, GST pull-down experiments using Drosophila extract from WT or Buffy mutant strain expressing Debcl-HA protein.

Regarding the complex formation, it was a speculation based on our genetic and biochemical data. In the revised paper, as the first reviewer suggested, we performed sequential IP and showed that Synr likely binds to Debcl/Buffy simultaneously (Fig 2R, please see our response to the reviewer 1).

Regarding our biochemical assay, we purified Drosophila protein from E.coli. Although we used HEK293T cells as a source of Drosophila Buffy and Debcl, we are using cell culture as a system to express Buffy and Debcl. For example, previous research also used HEK293T cells to demonstrate interaction between Debcl and Buffy (Quinn *et al*, 2003). We also respectfully note that even if we use Drosophila lysates, it does not guarantee that the interaction is direct. In fact, that possibility of indirect interaction might become higher if we use the lysate from the same species with the bait protein. To resolve this issue, in the revised we demonstrate a direct association of Sayonara and Buffy/Debcl based on BPA cross linking experiment (Fig 2N-P). Photoactivation of BPA induces direct covalent crosslinking of two proteins. This demonstrates that there is a direct interaction between Synr and Debc/Buffy.

15- Fig 3B: What is the third column for?

Magnification. This was clarified in the legend.

16- Fig 3C and Fig S2, the authors look at salivary glands, but it is never explained in the result section. Could the authors clarify this point in the text. Is it the case in wing imaginal disc?

We apologize that we failed to explain our rationale to use salivary glands. We just used salivary glands because it was easy to visualize proteins due to their large cell size. In this revised report, we also added data of sayonara int the wing disc (Fig 3C, EV2A).

17- Fig 3D-E: same problem as in point 9 above.

We added additional controls with quantification (Fig 3D-F, EV2B-C).

18- Fig3F: why do the authors use propidium iodide? A TUNEL experiment would be more suitable.

We here used PI as a death marker, that can label cells undergoing necrosis, including secondary necrosis following apoptosis. At this point, before showing data of caspase activation, we wanted to show a phenotype of cell death rather than apoptosis. In the revised manuscript, we also added data of TUNEL (Fig 3G).

19- Fig 3G and 3H what are "X" and "+" standing for? A control RNAi strain of the same genetic background as the other RNAi strains should be used as a control (see point 9).

X or + are control w- control strains we often use in the lab. In the revised manuscript, we added other more proper RNAis. Again, in the original manuscript, based on our RNAis screening, we did not consider random RNAi can affect the phenotype.

20- Fig3 J-L: What does the GFP labelling correspond to? What is RFP O/E standing for? Is it used as a control?

O/E is over expression. RFP was just used a UAS control. GFP was expressed to clarify localization of the nubbin-gal4 expression in the wing pouch.

21- The phenotypic suppression test with Reaper has been done with the vg-Gal4 driver whereas lysotracker and m-cherry Atg8 labelling have been done using nub-Gal4. Could the authors explain why they use different drivers? When induced by the vg-Gal4 driver, Reaper induces notched-wing phenotypes (Figure 3L(1)). Is it the case when Reaper is induced by the nub-Gal4 driver? Could the authors present a picture of the nub>Rpr wing phenotype?

Reaper with nubbin-gal4 was too strong. It is leathal. Vg-gal4 expression is relatively restricted to the DV boundary of the wing disc. For the phenotypic analyses of adult wings, we had to keep animals alive, that's why we are using vg-gal4 in the corresponding figure.

22- I agree that Reaper-induced wing phenotypes may result from autophagy and apoptosis but I think the results presented in 3L do not prove that autophagy and apoptosis activate each other. The authors do not quantify apoptosis or autophagy in the flies in Figure 3L.

We agree with this reviewer's important comment. Although we demonstrate that rpr induces autophagy and that rpr-induced adult wing phenotype is suppressed by autophagy inhibition, we failed to show actual feedback between autophagy and apoptosis with rpr, as we have done in a context of sayonara. We tried to check the existence of such a feedback in a context of rpr, but the results are confusing and need much more time and effort for further clarification. We think that one problem is rpr is such a strong inducer of caspase activation to assess the feedback. Since the reaper part is not a main component of this paper, we toned down this part in the revised report and emphasized the feedback aspect only in a context of sayonara.

23- Did the authors study Reaper-induced apoptosis or phenotype in a synr RNAi context?

We have done this analysis (please see the data below). We didn't see much effect of synr RNAi on the rpr-induced adult wing phenotype, which is consistent with the idea that reaper induces caspase activation in a separate signaling from the Bcl2 pathway.

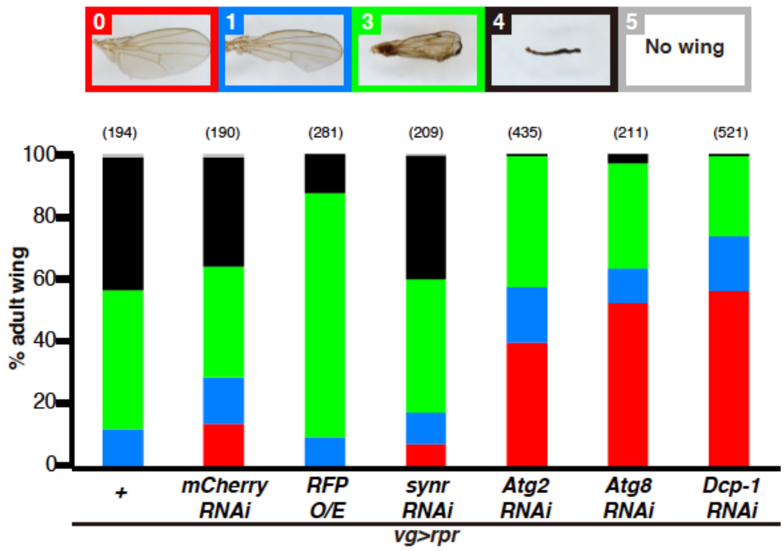

24- Fig4F is too speculative (see point 14)

It's a speculative model reasonably based on our genetic and biochemical data. We humbly believe that this kind of schematic will help readers understand our paper. I hope the review will be okay with it.

25- It has been clearly reported that Buffy is able to physically interact with the Debcl and that these two proteins often act in an opposite way to control apoptosis. It can be hypothesized that Synr interacts only with Buffy through its BH3 domain and that the interaction with Debcl, that do not involve BH3 and cdd domains, is indirect and occurs through the Buffy protein. This hypothesis could explain the results of the phenotypic interaction tests between synr and buffy RNAi or synr and debcl RNAi, if those results are confirmed. This may be considered in the discussion section.

Previously, Sharad Kumar's lab demonstrated that Buffy and Debcl interact using HEK293T cell lysates (Quinn *et al.*, 2003). Isabelle Guenal's lab also demonstrated Buffy can inhibit binding of Debcl and Drp1using fly lysates. We also confirmed that Buffy and Debcl physically interact based on GST-Buffy purified from E. coli and Debcl from HEK293T cells (data not shown). Although these data do not prove that Buffy and Debcl interact directly, most likely, their interaction is direct. As explained above, based on the photocrosslinking with BPA, we demonstrate that sayonara binds to Debcl/Buffy directly.

Let us summarize 4 pieces of data we obtained ourselves.

1. Sayoanra-induced caspase activation and cell death are suppressed by knockdown of either buffy or debcl.
2. The BH3 motif of Synr is important for sayonara-induced cell death.
3. Sayonara binds to Buffy and Debcl. Although the BH3 motif is dispensable for the gross interaction, BH3 directly binds to Buffy/Debcl.
4. Based on GST-pulldown and sequential pulldown, Synr likely makes a complex with Buffy and Debcl.

5. Buffy and Debcl binds each other, likely directly.

Based on these, we are making a plausible model that Buffy and Debcl work in a cooperative manner at least in a context of sayonara. The difference between our results and some of previous literature may come from existence of the BH3-only protein Sayonara. We discussed the coordination of Synr, Buffy and Debcl in both the result and discussion sections, mentioning on the interaction of Buffy and Debcl.

More general points

1- P2 "...cell death program, which is primarily regulated by the inhibitor of apoptosis (IAP) family of proteins..." should be changed in "...cell death program, which is primarily regulated by the RHG proteins that antagonize inhibitor of apoptosis (IAP) family of proteins..."

The text was modified as kindly suggested by the reviewer.

2- P2 "BH3-only proteins work as important stress sensors that initiate caspase activation" is ambiguous. BH3 proteins do not directly activate caspases. "BH3-only proteins work as important stress sensors that initiate events leading to caspase activation" would be better.

The text was modified as kindly suggested by the reviewer.

3- P2 Similarly, "...BH3-only proteins work as important stress sensors that initiate caspase activation..." could be replaced by "...BH3-only proteins work as important stress sensors that initiate the caspase activation pathway".

The text was modified as kindly suggested by the reviewer.

4- The legends of the figures are sometimes incomplete. The staining used for each microscopy panel must be precise in the figure caption. Genotypes and statistical tests should be indicated in each figure caption.

We modified the legends in the revised text.

5- The imaginal discs orientation (antero-posterior) has to be indicated.

In all pictures, we made our best effort to show wing discs making the anterior left, the posterior right, the dorsal bottom and the ventral top.

6- The same problem appears recurrently in the text. "BH3-only protein that activates caspase" could be replaced by " BH3-only protein that initiates a caspase activation pathway. " in some parts of the text.

Although we are not implying the activation is direct, we understand the reviewer’s concern. We modified the corresponding text as much as possible in the revised text.

7- In fig 1E: "caspase", not "caspsae"

We appreciate this reviewer's careful catch!

Referee #3:

Bcl-2 family proteins are critical regulators of caspases in vertebrates and C. elegans, with BH3-only proteins initiating a shift in balance of other Bcl-2 family proteins. However, BH3-only proteins had remained at large in Drosophila. Here, the authors identify a putative BH3-only Drosophila gene, Synr. They show that the encoded protein has signature BH3-only residues. They show evidence that Synr can induce apoptosis and/or autophagy, and this activity requires other Bcl-2 family proteins and caspases. They further show that Synr inhibition can suppress p53-induced phenotypes in Drosophila tissues.

Overall, this study is potentially very interesting. If the authors can support their claims with strong data, it would change the way we think about the Bcl-2 family function in Drosophila. However, there are a number of technical issues that fall short of convincingly supporting their claims. Below are a few specific points that the authors should try to address:

We thank this reviewer for his/her supportive comments.

1. As the authors cite, BH3 motifs have very little sequence conservation aside from a few key residues. Because of such poor conservation (as shown in Figure 1A), sequence alignment of the putative BH3-motif does not necessarily make a compelling case. Perhaps with this in mind, the authors show that Synr can help activate caspases in vivo, and that deleting the entire BH3-domain abolishes this Synr function. Still, it will linger in the readers' minds that the putative BH3-motif has poor sequence conservation. It remains possible that this sequence has other functions unrelated to BH3-function, which could have been abolished by the deletion of the entire sequence. To quell such concerns, I suggest generating BH3 point mutants in which the "signature BH3 only residues" are substituted (instead of the entire deletion of the region). That experiment will support the idea that the "BH3-signature sequences" are functionally relevant.

To address this issue, we newly created a BH3 amino acid substitution mutant by changing the four conserved hydrophobic amino acids to glutamate, as previously performed (Chen *et al*, 2005) during revision (Fig 1L-M). We also verified that neither deletion nor amino acid substitution of BH3 reduces its expression level by newly generating HA-tagged sayonara (Fig EV1F).

2. In Figure 2, the authors show that nub>synr phenotype is suppressed by adding buffy RNAi in the background - which is potentially very interesting. But it is important to have a control RNAi line in Fig. 2A and E to have the same number of uas-elements in each condition (the authors do this in Figure 3L for rpr suppression experiments). Otherwise, it remains a possibility that the suppression of the nub>synr phenotype is due to the titration of Gal4.

We added appropriate controls in this revision. Please see our responses to the reviewers 1 and 2.

3. Figure 2A-D shows representative images only. Some type of a quantification is needed.

We added quantification and took further control (Fig 2A-E).

4. The binding experiments in Figure 2H-N is interesting. But to highlight the importance of the putative BH3-motif, the authors should use BH3-point mutants (as suggested for point #1 above) that substitute signature BH3 sequences instead of deleting the entire BH3 region.

We concluded that even the BH3 deletion does not affect binding of Synr to Buffy or Debcl. So, we expect to see no effect by the BH3 amino acid substitution since the mutation effect should be weaker compared to the deletion. To verify an importance of the BH3 motif in this revised paper, we started a new collaboration with Takuya Shiota, an expert of photo crosslinking. Using BPA-mediated photo crosslinking, we demonstrate a direct binding of the Synr BH3 motif and Bcl2 proteins in a definitive manner (Fig 2N-P).

5. Figure 3 shows Synr's ability to induce autophagy. The authors should be aware that they are not the first to implicate Drosophila Bcl-2 family proteins in regulating autophagy. Citing previous papers reporting that buffy, debcl, dcp-1 regulates autophagy (e.g., PMID 19242106 and 18794330) would provide a helpful background regarding the Synr-autophagy link.

We apologize that we failed to cite sufficient literature in the original manuscript. In the revised manuscript, we emphasized that caspase is known to active autophagy and that autophagy can activate caspase vice versa. But, these have only been demonstrated in separate contexts, never in the same setting. In this report, we show that at least in a context of sayonara, autophagy and caspase activate each other, forming a feedback loop. Again, each pathway was already known, but a novelty of our findings is to combine/integrate previous insights into a feedback loop in a single setting. To the best of our knowledge, this feedback loop between apoptosis and autophagy has not been demonstrated previously: it has always been one way direction.

6. The authors show a number of RNAi experiments throughout the manuscript. Many of those appear to rely on single RNAi lines (indicated in Table S1), leaving open the possibility of off-target effects. The results need to be validated through independent means, such as the use of second RNAi lines (targeting a different region), or the utilization of other loss-of-function strategies.

To address this issue, we made an effort to generate a new shRNA for sayonara. It shows a similar phenotype to the previous synr shRNA (Fig EV3B). We also used multiple RNAis for most genes (Fig 2E). Regarding RNAis for ATG genes, we used only one RNAi for each ATG, because we used previously verified RNAis for ATGs

and also these ATGs work in the same pathway. We believe this strategy would work better than, for example, using two RNAis for only a single ATG gene without examining additional ATG genes.

Let us also share our behind-the scene story. We screened a large number of RNAis for suppressor of the Sayonara-induced phenotype. We only got a very little number of positive hits. In general, it's extremely difficult to make unhealthy (dying) tissues to healthy, compared to inducing non-specific toxicity by RNAi-mediated off-target effects. We also share our observation that we never observed Gal4 dilution or titration by adding another UAS, which is consistent with the fact that each UAS exists as one copy in the genome compared to the astronomical number of Gal4 molecules. That is why in the original manuscript, we abbreviated adding non-specific UAS. We have got so much negative data with tons of RNAis we used.

Chen L, Willis SN, Wei A, Smith BJ, Fletcher JI, Hinds MG, Colman PM, Day CL, Adams JM, Huang DC (2005) Differential targeting of prosurvival Bcl-2 proteins by their BH3-only ligands allows complementary apoptotic function. *Mol Cell* 17: 393-403

Chin JW, Martin AB, King DS, Wang L, Schultz PG (2002) Addition of a photocrosslinking amino acid to the genetic code of Escherichiacoli. *Proc Natl Acad Sci U S A* 99: 11020-11024

Ciesielski HM, Nishida H, Takano T, Fukuhara A, Otani T, Ikegawa Y, Okada M, Nishimura T, Furuse M, Yoo SK (2022) Erebosis, a new cell death mechanism during homeostatic turnover of gut enterocytes. *PLoS Biol* 20: e3001586

Doumanis J, Dorstyn L, Kumar S (2007) Molecular determinants of the subcellular localization of the Drosophila Bcl-2 homologues DEBCL and BUFFY. *Cell Death Differ* 14: 907-915

Jiang F, Doudna JA (2017) CRISPR-Cas9 Structures and Mechanisms. *Annu Rev Biophys* 46: 505-529

Kishi KE, Kim YS, Fukuda M, Inoue M, Kusakizako T, Wang PY, Ramakrishnan C, Byrne EFX, Thadhani E, Paggi JM *et al* (2022) Structural basis for channel conduction in the pump-like channelrhodopsin ChRmine. *Cell* 185: 672-689 e623

Nishida H, Okada M, Yang L, Takano T, Tabata S, Soga T, Ho DM, Chung J, Minami Y, Yoo SK (2021) Methionine restriction breaks obligatory coupling of cell proliferation and death by an oncogene Src in Drosophila. *Elife* 10

Quinn L, Coombe M, Mills K, Daish T, Colussi P, Kumar S, Richardson H (2003) Buffy, a Drosophila Bcl-2 protein, has anti-apoptotic and cell cycle inhibitory functions. *EMBO J* 22: 3568-3579

Sasaki A, Nishimura T, Takano T, Naito S, Yoo SK (2021) white regulates proliferative homeostasis of intestinal stem cells during ageing in Drosophila. *Nat Metab* 3: 546-557

Shiota T, Mabuchi H, Tanaka-Yamano S, Yamano K, Endo T (2011) In vivo protein-interaction mapping of a mitochondrial translocator protein Tom22 at work. *Proc Natl Acad Sci U S A* 108: 15179-15183

Yoo SK, Pascoe HG, Pereira T, Kondo S, Jacinto A, Zhang X, Hariharan IK (2016) Plexins function in epithelial repair in both Drosophila and zebrafish. *Nat Commun* 7: 12282

2nd Revision - Editorial Decision 22nd Dec 2022

Dear Sa Kan,

Thank you for submitting your revised manuscript to The EMBO Journal. Your study has now been re-reviewed by the original referees. As you can see from the comments below, the referees appreciate the introduced changes and support publication here. They have a few remaining queries that should be easy enough to sort out. When you submit the revised version will you also address the following points:

- please add 3-5 keywords to the title page

- Please remove the Authors Contributions from the manuscript. The 'Author Contributions' section is replaced by the CRediT contributor roles taxonomy to specify the contributions of each author in the journal submission system. Please use the free text box in the 'author information' section of the manuscript submisssion system to provide more detailed descriptions (e.g., 'X provided intracellular Ca++ measurements in fig Y')

- Please upload high resolution figure files including for the EV figures

- The manuscript sections need to be arranged in the right order. See also guide to authors.

- "Methods" needs correcting to 'Materials and Methods'.

- The expanded view figure legends should follow-on after the main figure legends. Add the heading 'Expanded View Figure Legends'.

- I see that you have uploaded a Reagent table, but it is not complete and contains "only" the drosophila strains and not all the reagents used. It is OK to keep it like that, but in this case upload it as a Dataset EV1 file and change the callout in the text to Dataset EV1 .

- Our publisher has also done their pre-publication check on your manuscript. When you log into the manuscript submission system you will see the file "Data Edited Manuscript file". Please take a look at the word file and the comments regarding the figure legends and respond to the issues.

- Please upload a synopsis text => a summary statement plus 3-5 bullet points describing the key findings of the

- We also need a synopsis image => 550 wide by [200-400]

- Regarding the source data files:

For main figures upload one file/folder (zipped) per figure labelled Source Data figure 1 etc.

For the EV figures: zip all source data files in one file labelled Source Data EV figures

That should be all! I will accept the revised version once I get it back in.

Congratulations on a nice study

Best Karin

Karin Dumstrei, PhD
Senior Editor
The EMBO Journal

Instructions for preparing your revised manuscript:

Guide For Authors: https://www.embopress.org/page/journal/14602075/authorguide

Use the link below to submit your revision:

https://emboj.msubmit.net/cgi-bin/main.plex

Referee #1:

The authors have put considerable effort into addressing the reviewers' concerns, and the manuscript is significantly improved. There are a few further modifications and clarifications that are needed to solidify this interesting manuscript.
The authors show that knockdown of buffy and debcl inhibit caspase activation and tissue loss induced by synr expression and that Synr interacts with Buffy and Debcl. Do they think that it is this three part complex that is needed for killing? This would imply that synr does not inhibit the anti-apoptotic activity buffy or debcl, but rather activate their pro-apoptotic activity. This conclusion has implications for thinking about the comparison to the mammalian system. The authors should add some discussion about this. Can they discuss they model for how the interactions activate apoptosis and autophagy?
In Figure 2H-L, the break between the anti-Flag and anti-GST immunoblots should be clarified with a line or at least a wider gap, as it is a bit unclear.
On page 6 the synr mutant is introduced. It would be helpful to have more description of the nature of the mutant insertion in the text. Also, viability does not indicate a lack of necessity for apoptosis. It would be easy for the authors to show that apoptosis occurs normally in some tissues such as the developing CNS or eye.
On page 7 there is the statement that rpr-mediated caspase activation induced autophagy (shown in figure EV2 E&F). However, the interesting data showing that inhibition of autophagy suppresses tissue loss, as shown in EV2G is not mentioned. It would be very nice if they showed that rpr-induced caspase activation is also inhibited by ATG knockdown, similar to what is seen with synr.
In Figure 4A, the nub>p53 wing phenotype is so strongly suppressed by synr RNAi, that is surprising that the caspase activation is only partially suppressed. Can the range of the wing phenotype suppression be quantified, similar to what is shown in EV2G?

Referee #2:

The authors answered my questions and have done the requested changes. The additional experiments performed and added to the article significantly improved it and convincingly support the authors' conclusions.
I have a minor comment regarding one of the new figures: the caption of figure EV1 needs to be clarified. Do the two photographs presented for each genotype correspond to two different focal plans of the wing pouch of the same wing imaginal disc or to two different discs?

Referee #3:

While Bcl-2 family proteins play critical roles in apoptosis across phyla, the presence of BH3-only domain proteins in Drosophila had remained unclear. Such absence of a BH3-only factor was puzzling because apoptosis plays critical roles in Drosophila as in other organisms. The authors have addressed all of my previous points in the revised manuscript. I think this is a highly interesting manuscript. I have no further concerns.

We provide point-by-point responses below.

Referee #1:

The authors have put considerable effort into addressing the reviewers' concerns, and the manuscript is significantly improved. There are a few further modifications and clarifications that are needed to solidify this interesting manuscript.
The authors show that knockdown of buffy and debcl inhibit caspase activation and tissue loss induced by synr expression and that Synr interacts with Buffy and Debcl. Do they think that it is this three part complex that is needed for killing? This would imply that synr does not inhibit the anti-apoptotic activity buffy or debcl, but rather activate their pro-apoptotic activity. This conclusion has implications for thinking about the comparison to the mammalian system. The authors should add some discussion about this. Can they discuss they model for how the interactions activate apoptosis and autophagy?

In the result section, we had the following text:

Based on these biochemical data and genetic data, we speculate that Synr makes a complex with Buffy and Debcl, where Synr's BH3 motif could exert a crucial effect on the protein complex's function. It is of note that Buffy and Debcl themselves interact each other (Quinn et al., 2003), indicating that all the three proteins can interact each other. The idea that Synr makes a complex with Debcl and Buffy is consistent with our genetic data that inhibition of either Debcl or Buffy is sufficient to suppress the synr-induced phenotype.

In the discussion section, we modified text as follows:

Based on our genetic and biochemical data, we propose that Synr makes a complex with Debcl and Buffy, which is critical for caspase activation. In this model, both Debcl and Buffy function as pro-apoptotic BCL-2 proteins. This is reminiscent of the direct model rather than the indirect model in mammalian BH3-only proteins (Aouacheria et al., 2015; Bouillet & Strasser, 2002; Doerflinger et al., 2015; Giam et al., 2008; Happo et al., 2012; Lomonosova & Chinnadurai, 2008; Shamas-Din et al., 2011). One unique aspect of the BH3 motif of Synr is, in spite of its importance in Synr proapoptotic activity, being dispensable for Synr overall binding to Debcl/Buffy. At the same time, the BH3 motif binds to Deblcl/Buffy, as demonstrated by the photocrosslinking at the BH3 motif. We speculate that the local binding of Synr BH3 and Debc/Buffy is critical for the function of the protein complex. Future protein structural analyses will reveal the molecular details by which Synr-BH3 motif exerts its function.

We believe the text above addresses the review's comment sufficiently. We also discussed the relationship of apoptosis and autophagy in discussion.

In Figure 2H-L, the break between the anti-Flag and anti-GST immunoblots should be clarified with a line or at least a wider gap, as it is a bit unclear.

Many thanks for this kind notice. We modified the figure accordingly.

On page 6 the synr mutant is introduced. It would be helpful to have more description of the nature of the mutant insertion in the text. Also, viability does not indicate a lack of necessity for apoptosis. It would be easy for the authors to show that apoptosis occurs normally in some tissues such as the developing CNS or eye.

We added detailed information on the mutant in Fig EV3C-E. Regarding apoptosis in flies, if apoptosis does not occur during development as in the case of rpr, hid, grim mutant (H99), normal development does not occur. Since the sayonara mutant develops normally, apoptosis should occur during development, which is consistent with the phenotypes of Buffy and Debcl mutants (Sevrioukov *et al*, 2007). We clarified this point in text.

On page 7 there is the statement that rpr-mediated caspase activation induced autophagy (shown in figure EV2 E&F). However, the interesting data showing that inhibition of autophagy suppresses tissue loss, as shown in EV2G is not mentioned. It would be very nice if they showed that rpr-induced caspase activation is also inhibited by ATG knockdown, similar to what is seen with synr.

We need to humbly indicate that EV2G is already mentioned in the original, revised manuscript.

Regarding whether ATG inhibition can suppress rpr-induced apoptosis activation, we previously discussed it extensively in a response to the reviewer 2 (22nd point). Let us reiterate what we wrote there.

We agree with this reviewer's important comment. Although we demonstrate that rpr induces autophagy and that rpr-induced adult wing phenotype is suppressed by autophagy inhibition, we failed to show actual feedback between autophagy and apoptosis with rpr, as we have done in a context of sayonara. We tried to check the existence of such a feedback in a context of rpr, but the results are confusing and need much more time and effort for further clarification. We think that one problem is rpr is such a strong inducer of caspase activation to assess the feedback. Since the reaper part is not a main component of this paper, we toned down this part in the revised report and emphasized the feedback aspect only in a context of sayonara.

In Figure 4A, the nub>p53 wing phenotype is so strongly suppressed by synr RNAi, that is surprising that the caspase activation is only partially suppressed. Can the range of the wing phenotype suppression be quantified, similar to what is shown in EV2G?

We think that the apparent difference between the wing phenotype and caspase activation in the wing disc comes from the robustness of the wing formation. In general, a little bit apoptosis does not affect the overall wing formation, which is intrinsically a robust process. Since characterization of the wing phenotype based on categorization is not so quantitative and is not a direct readout of caspase activation, we think that detection of caspase activation by GC3Ai is more quantitative and direct. That is why we quantified the caspase activation, which is a more direct indicator of apoptosis. We clarified this point in text.

Referee #2:

The authors answered my questions and have done the requested changes. The additional experiments performed and added to the article significantly improved it and convincingly support the authors' conclusions.

We appreciate this review's encouraging comment.

I have a minor comment regarding one of the new figures: the caption of figure EV1 needs to be clarified. Do the two photographs presented for each genotype correspond to two different focal plans of the wing pouch of the same wing imaginal disc or to two different discs?

We assume that the reviewer is mentioning on Fig EV1F. These are showing two examples of each condition. We clarified this in the corresponding legend.

Referee #3:

While Bcl-2 family proteins play critical roles in apoptosis across phyla, the presence of BH3-only domain proteins in Drosophila had remained unclear. Such absence of a BH3-only factor was puzzling because apoptosis plays critical roles in Drosophila as in other organisms. The authors have addressed all of my previous points in the revised manuscript. I think this is a highly interesting manuscript. I have no further concerns.

We thank this review's supportive comment.

3rd Revision - Editorial Decision **28th Dec 2022**

Dear Sakan,

Thank you for submitting your revised manuscript. I have now looked at everything and all looks good!

I asm therefore very pleased to accept the manuscript for publication here.

Congratulations on a nice study!

with best wishes

Karin

Karin Dumstrei, PhD
Senior Editor
The EMBO Journal

Please note that it is EMBO Journal policy for the transcript of the editorial process (containing referee reports and your response letter) to be published as an online supplement to each paper. If you do NOT want this, you will need to inform the Editorial Office via email immediately. More information is available here:
https://www.embopress.org/page/journal/14602075/authorguide#transparentprocess

Your manuscript will be processed for publication in the journal by EMBO Press. Manuscripts in the PDF and electronic editions of The EMBO Journal will be copy edited, and you will be provided with page proofs prior to publication. Please note that supplementary information is not included in the proofs.

You will be contacted by Wiley Author Services to complete licensing and payment information. The required 'Page Charges Authorization Form' is available here: https://www.embopress.org/pb-assets/embo-site/tej_apc.pdf - please download and complete the form and return to embopressproduction@wiley.com

EMBO Press participates in many Publish and Read agreements that allow authors to publish Open Access with reduced/no publication charges. Check your eligibility: https://authorservices.wiley.com/author-resources/Journal-Authors/open-access/affiliation-policies-payments/index.html

Should you be planning a Press Release on your article, please get in contact with embojournal@wiley.com as early as possible, in order to coordinate publication and release dates.

If you have any questions, please do not hesitate to call or email the Editorial Office. Thank you for your contribution to The EMBO Journal.

** Click here to be directed to your login page: https://emboj.msubmit.net

EMBO Press Author Checklist

Corresponding Author Name: Sa Kan Yoo	
Journal Submitted to: EMBO	
Manuscript Number: EMBOJ-2021-110454R1	

USEFUL LINKS FOR COMPLETING THIS FORM
The EMBO Journal - Author Guidelines
EMBO Reports - Author Guidelines
Molecular Systems Biology - Author Guidelines
EMBO Molecular Medicine - Author Guidelines

Reporting Checklist for Life Science Articles (updated January
This checklist is adapted from Materials Design Analysis Reporting (MDAR) Checklist for Authors. MDAR establishes a minimum set of requirements in transparent reporting in the life sciences (see Statement of Task: 10.31222/osf.io/9sm4x). Please follow the journal's guidelines in preparing your
Please note that a copy of this checklist will be published alongside your article.

Abridged guidelines for figures
1. Data
The data shown in figures should satisfy the following conditions:

- the data were obtained and processed according to the field's best practice and are presented to reflect the results of the experiments in an accurate and unbiased manner.
- ideally, figure panels should include only measurements that are directly comparable to each other and obtained with the same assay.
- plots include clearly labeled error bars for independent experiments and sample sizes. Unless justified, error bars should not be shown for technical
- if n<5, the individual data points from each experiment should be plotted. Any statistical test employed should be justified.
- Source Data should be included to report the data underlying figures according to the guidelines set out in the authorship guidelines on Data

2. Captions
Each figure caption should contain the following information, for each panel where they are relevant:

- a specification of the experimental system investigated (eg cell line, species name).
- the assay(s) and method(s) used to carry out the reported observations and measurements.
- an explicit mention of the biological and chemical entity(ies) that are being measured.
- an explicit mention of the biological and chemical entity(ies) that are altered/varied/perturbed in a controlled manner.
- the exact sample size (n) for each experimental group/condition, given as a number, not a range;
- a description of the sample collection allowing the reader to understand whether the samples represent technical or biological replicates (including how many animals, litters, cultures, etc.).
- a statement of how many times the experiment shown was independently replicated in the laboratory.
- definitions of statistical methods and measures:
  - common tests, such as t-test (please specify whether paired vs. unpaired), simple χ2 tests, Wilcoxon and Mann-Whitney tests, can be unambiguously identified by name only, but more complex techniques should be described in the methods section;
  - are tests one-sided or two-sided?
  - are there adjustments for multiple comparisons?
  - exact statistical test results, e.g., P values = x but not P values < x;
  - definition of 'center values' as median or average;
  - definition of error bars as s.d. or s.e.m.

Please complete ALL of the questions below.
Select "Not Applicable" only when the requested information is not relevant for your study.

Materials

Newly Created Materials	Information included in the manuscript?	In which section is the information available? (Reagents and Tools Table, Materials and Methods, Figures, Data Availability Section)
New materials and reagents need to be available; do any restrictions apply?	Yes	method

Antibodies	Information included in the manuscript?	In which section is the information available? (Reagents and Tools Table, Materials and Methods, Figures, Data Availability Section)
For **antibodies** provide the following information:<br>- Commercial antibodies: RRID (if possible) or supplier name, catalogue number and or/clone number<br>- Non-commercial: RRID or citation	Yes	method

DNA and RNA sequences	Information included in the manuscript?	In which section is the information available? (Reagents and Tools Table, Materials and Methods, Figures, Data Availability Section)
**Short novel DNA or RNA including primers, probes**: provide the sequences.	Yes	method

Cell materials	Information included in the manuscript?	In which section is the information available? (Reagents and Tools Table, Materials and Methods, Figures, Data Availability Section)
**Cell lines:** Provide species information, strain. Provide accession number in repository **OR** supplier name, catalog number, clone number, and/**OR** RRID.	Yes	method
**Primary cultures:** Provide species, strain, sex of origin, genetic modification status.	Not Applicable	
Report if the cell lines were recently **authenticated** (e.g., by STR profiling) and tested for mycoplasma contamination.	Not Applicable	

Experimental animals	Information included in the manuscript?	In which section is the information available? (Reagents and Tools Table, Materials and Methods, Figures, Data Availability Section)
**Laboratory animals or Model organisms:** Provide species, strain, sex, age, genetic modification status. Provide accession number in repository **OR** supplier name, catalog number, clone number, **OR** RRID.	Yes	method
**Animal observed in or captured from the field:** Provide species, sex, and age where possible.	Not Applicable	
Please detail **housing and husbandry conditions**.	Not Applicable	

Plants and microbes	Information included in the manuscript?	In which section is the information available? (Reagents and Tools Table, Materials and Methods, Figures, Data Availability Section)
**Plants:** provide species and strain, ecotype and cultivar where relevant, unique accession number if available, and source (including location for collected wild specimens).	Not Applicable	
**Microbes:** provide species and strain, unique accession number if available, and source.	Not Applicable	

Human research participants	Information included in the manuscript?	In which section is the information available? (Reagents and Tools Table, Materials and Methods, Figures, Data Availability Section)
If collected and within the bounds of privacy constraints report on age, sex and gender or ethnicity for all study participants.	Not Applicable	

Core facilities	Information included in the manuscript?	In which section is the information available? (Reagents and Tools Table, Materials and Methods, Figures, Data Availability Section)
If your work benefited from core facilities, was their service mentioned in the acknowledgments section?	Not Applicable	

Design

Study protocol	Information included in the manuscript?	In which section is the information available? (Reagents and Tools Table, Materials and Methods, Figures, Data Availability Section)
If study protocol has been **pre-registered, provide DOI in the manuscript**. For clinical trials, provide the trial registration number **OR** cite DOI.	Not Applicable	
Report the **clinical trial registration number** (at ClinicalTrials.gov or equivalent), where applicable.	Not Applicable	

Laboratory protocol	Information included in the manuscript?	In which section is the information available? (Reagents and Tools Table, Materials and Methods, Figures, Data Availability Section)
Provide DOI OR other citation details if **external detailed step-by-step protocols** are available.	Not Applicable	

Experimental study design and statistics	Information included in the manuscript?	In which section is the information available? (Reagents and Tools Table, Materials and Methods, Figures, Data Availability Section)
Include a statement about **sample size** estimate even if no statistical methods were used.	Yes	method
Were any steps taken to minimize the effects of subjective bias when allocating animals/samples to treatment (e.g. **randomization procedure**)? If yes, have they been described?	Yes	method
Include a statement about **blinding** even if no blinding was done.	Yes	method
Describe **inclusion/exclusion criteria** if samples or animals were excluded from the analysis. Were the criteria pre-established?<br><br>If sample or data points were omitted from analysis, report if this was due to attrition or intentional exclusion and provide justification.	Not Applicable	
For every figure, are **statistical tests** justified as appropriate? Do the data meet the assumptions of the tests (e.g., normal distribution)? Describe any methods used to assess it. Is there an estimate of variation within each group of data? Is the variance similar between the groups that are being statistically compared?	Yes	method

Sample definition and in-laboratory replication	Information included in the manuscript?	In which section is the information available? (Reagents and Tools Table, Materials and Methods, Figures, Data Availability Section)
In the figure legends: state number of times the experiment was **replicated** in laboratory.	Yes	legend
In the figure legends: define whether data describe **technical or biological replicates**.	Yes	legend

Ethics

Ethics	Information included in the manuscript?	In which section is the information available? (Reagents and Tools Table, Materials and Methods, Figures, Data Availability Section)
Studies involving **human participants**: State details of **authority granting ethics approval** (IRB or equivalent committee(s), provide reference number for approval.	Not Applicable	
Studies involving **human participants**: Include a statement confirming that **informed consent** was obtained from all subjects and that the experiments conformed to the principles set out in the WMA Declaration of Helsinki and the Department of Health and Human Services Belmont Report.	Not Applicable	
Studies involving **human participants:** For publication of **patient photos**, include a statement confirming that consent to publish was obtained.	Not Applicable	
Studies involving experimental **animals**: State details of **authority granting ethics approva**l (IRB or equivalent committee(s), provide reference number for approval. Include a statement of compliance with ethical regulations.	Not Applicable	
Studies involving **specimen and field samples:** State if relevant **permits** obtained, provide details of authority approving study; if none were required, explain why.	Not Applicable	

Dual Use Research of Concern (DURC)	Information included in the manuscript?	In which section is the information available? (Reagents and Tools Table, Materials and Methods, Figures, Data Availability Section)
Could your study fall under dual use research restrictions? Please check biosecurity documents and list of **select agents and toxins** (CDC): https://www.selectagents.gov/sat/list.htm	Not Applicable	
If you used a select agent, is the security level of the lab appropriate and reported in the manuscript?	Not Applicable	
If a study is subject to dual use research of concern regulations, is the name of the **authority granting approval and reference number** for the regulatory approval provided in the manuscript?	Not Applicable	

Reporting

The MDAR framework recommends adoption of discipline-specific guidelines, established and endorsed through community initiatives. Journals have their own policy about requiring specific guidelines and recommendations to complement MDAR.

Adherence to community standards	Information included in the manuscript?	In which section is the information available? (Reagents and Tools Table, Materials and Methods, Figures, Data Availability Section)
State if relevant guidelines or checklists (e.g., **ICMJE, MIBBI, ARRIVE, PRISMA**) have been followed or provided.	Not Applicable	
For **tumor marker prognostic studies,** we recommend that you follow the **REMARK** reporting guidelines (see link list at top right). See author guidelines, under 'Reporting Guidelines'. Please confirm you have followed these guidelines.	Not Applicable	
For **phase II and III randomized controlled trials**, please refer to the **CONSORT** flow diagram (see link list at top right) and submit the CONSORT checklist (see link list at top right) with your submission. See author guidelines, under 'Reporting Guidelines'. Please confirm you have submitted this list.	Not Applicable	

Data Availability

Data availability	Information included in the manuscript?	In which section is the information available? (Reagents and Tools Table, Materials and Methods, Figures, Data Availability Section)
Have **primary datasets** been deposited according to the journal's guidelines (see 'Data Deposition' section) and the respective accession numbers provided in the Data Availability Section?	Not Applicable	
Were **human clinical and genomic datasets** deposited in a public access-controlled repository in accordance to ethical obligations to the patients and to the applicable consent agreement?	Not Applicable	
Are **computational models** that are central and integral to a study available without restrictions in a machine-readable form? Were the relevant accession numbers or links provided?	Not Applicable	
If publicly available data were reused, provide the respective **data citations in the reference list**.	Not Applicable	
